# Microarray and deep sequencing cross-platform analysis of the mirRNome and isomiR variation in response to epidermal growth factor

**DOI:** 10.1186/1471-2164-14-371

**Published:** 2013-06-01

**Authors:** Franc Llorens, Manuela Hummel, Lorena Pantano, Xavier Pastor, Ana Vivancos, Ester Castillo, Heidi Mattlin, Anna Ferrer, Matthew Ingham, Marc Noguera, Robert Kofler, Juliane C Dohm, Raquel Pluvinet, Mònica Bayés, Heinz Himmelbauer, José Antonio del Rio, Eulàlia Martí, Lauro Sumoy

**Affiliations:** 1 Centre for Genomic Regulation (CRG), Barcelona, Spain; 2 Universitat Pompeu Fabra (UPF), Barcelona, Spain; 3 Molecular and Cellular Neurobiotechnology Group, Institut de Bioenginyeria de Catalunya (IBEC), Barcelona, Spain; 4 Department of Cell Biology, University of Barcelona (UB), Barcelona, Spain; 5 Centro de Investigación Biomédica en Red en Enfermedades Neurodegenerativas (CIBERNED), Barcelona, Spain; 6 Centro de Investigación Biomédica en Red en Epidemiología y Salud Pública (CIBERESP), Barcelona, Spain; 7 Institute of Predictive and Personalized Medicine of Cancer (IMPPC), Badalona, Spain; 8 Vall d'Hebron Institute of Oncology (VHIO), Barcelona, Spain; 9 Centro Nacional de Análisis Genómicos (CNAG), Barcelona, Spain; 10 Molecular Epidemiology Group, IRSI-Caixa Foundation, Barcelona, Spain; 11 Max Planck Institute for Molecular Genetics, Berlin, Germany; 12 Institute for Population Genetics, University of Veterinary Medicine, Vienna, Austria

## Abstract

**Background:**

Epidermal Growth Factor (EGF) plays an important function in the regulation of cell growth, proliferation, and differentiation by binding to its receptor (EGFR) and providing cancer cells with increased survival responsiveness. Signal transduction carried out by EGF has been extensively studied at both transcriptional and post-transcriptional levels. Little is known about the involvement of microRNAs (miRNAs) in the EGF signaling pathway. miRNAs have emerged as major players in the complex networks of gene regulation, and cancer miRNA expression studies have evidenced a direct involvement of miRNAs in cancer progression.

**Results:**

In this study, we have used an integrative high content analysis approach to identify the specific miRNAs implicated in EGF signaling in HeLa cells as potential mediators of cancer mediated functions. We have used microarray and deep-sequencing technologies in order to obtain a global view of the EGF miRNA transcriptome with a robust experimental cross-validation. By applying a procedure based on Rankprod tests, we have delimited a solid set of EGF-regulated miRNAs. After validating regulated miRNAs by reverse transcription quantitative PCR, we have derived protein networks and biological functions from the predicted targets of the regulated miRNAs to gain insight into the potential role of miRNAs in EGF-treated cells. In addition, we have analyzed sequence heterogeneity due to editing relative to the reference sequence (isomiRs) among regulated miRNAs.

**Conclusions:**

We propose that the use of global genomic miRNA cross-validation derived from high throughput technologies can be used to generate more reliable datasets inferring more robust networks of co-regulated predicted miRNA target genes.

## Background

EGF signaling activates an extensive network of signal transduction pathways leading to: 1) Activation or inhibition of genes regulating DNA synthesis, cell proliferation and pro- or anti- apoptotic pathways 2) rises in intracellular calcium levels, 3) increases in glycolysis and protein synthesis [1,2]. EGF signaling provides cancer cells with increased pro-survival responses and deregulated activity of this network leads to diverse types of tumors [2,3]. By targeting EGF survival pathways, cancer cells can be forced to undergo apoptosis or become sensitive to radiation or chemotherapy. Hence, targeted therapies that block EGF signaling have been successfully applied towards anti-cancer treatment [4].

Although the EGFR mediated signaling response is one of the best understood, questions such as how the specificity and dynamics of response to EGF is achieved or what are the regulatory feed-back mechanisms have been the subject of intense investigation to reach a global and integrative view of EGF-dependent cellular regulation [5-7]. Indeed, since EGF signaling pathways can be deregulated at many levels during cancer progression, a multi-faceted approach to target these pathways and their regulatory mechanisms for cancer treatment is required.

Over recent years, miRNAs have emerged as major players in the complex networks controlling gene regulation. These small, endogenous non-coding RNA molecules that regulate post-transcriptionally the expression of protein coding genes [8] have been implicated in various spectra of human pathobiology, such as cancer, metabolic disorders and infectious diseases ([9,10] for review). Cancer miRNA expression studies have proven that miRNA expression profiles can classify tumors in a very robust fashion, and mutational and functional analyses have evidenced a direct role of miRNAs in cancer progression [11,12]. Deregulation of miRNAs promotes carcinogenesis; hence these molecules can act as oncogenes or tumor suppressor genes [13]. Moreover, both miRNA inhibition and activation show great promise in the treatment of various types of cancer, as well as viral and metabolic diseases. Aberrant gene expression is the main reason for miRNA dysfunction in cancer, which results in abnormal miRNA levels in tumor samples. miRNA germline and somatic mutations or polymorphisms in the protein-coding mRNAs targeted by miRNAs also contribute to cancer predisposition, initiation or progression [14]. These findings have revealed that, besides considering the role of oncogenes and tumor suppressor protein coding genes, it will be essential to understand how miRNAs affect the responsiveness of cells to signaling molecules involved in such processes in order to advance in the knowledge of the mechanisms underlying malignant transformation [15]. While the EGF pathway has been investigated in depth at the phosphorylation [5], and gene expression levels [6,16] much less is known about how EGF may regulate miRNA expression or the role of such miRNAs in the regulation of the EGF-related cellular functions such as cell growth, proliferation and differentiation. Hayashi and collaborators [17] profiled the EGF-dependent microRNAs of the fetal mouse submandibular gland at embryonic day 13. Avraham and colleagues, [7] described a coordinated transcriptional program between miRNA and transcription factors revealed through an EGF treatment time course in HeLa cells. Similarly, a very recent study in lung cancer cell lines has addressed the miRNA response to EGFR inhibition by shRNA [18].

Optimal exploitation of genomics and bioinformatics technologies have provided many tools that have been successfully applied to the study of EGF signaling (establishing an elaborate model of an EGF-dependent transcriptional modulator network) [6] and to many types of cancers [19]. In addition, ongoing progress in ultra-sequencing technologies has eased and expanded the possibilities to perform measurements of millions of molecules in a single assay, allowing for even more precise modeling.

In this study, we present the complete view of the EGF-induced miRNA transcription in HeLa cells which has been cross-validated using different array and ultra-sequencing technologies, including the analysis of the miRNA variability in the EGF-induced miRNome, a phenomenon that may specifically influence the mechanisms of gene silencing or gene targeting under both physiological and pathological conditions. In parallel, we have developed specific bioinformatics and statistical tools to assist in the handling and analysis of the vast amounts of data generated.

## Methods

### Reagents and antibodies

EGF and anti-Tubulin (1:10000) were purchased from Sigma. Anti phosho-ERK1/2 (1:2000), anti phosho-AKT (1:1000) were from Cell Signaling. U0126, AG1478 and wortmannin were from Calbiochem.

### Cell culture and sample preparation

HeLa cells were cultured at 37°C in a 95/5 Air/CO_2_ water saturated atmosphere in Dulbecco’s modified Eagle’s medium (DMEM) containing 10% heat inactivated fetal bovine serum (FBS), 2 mM L-glutamine and 100U/ml Penicillin/streptomycin. For treatments, the cells were transferred to 60 mm dishes and after 48 hours starved for 24 hours in DMEM containing 0.5% FBS. The cells were incubated (if indicated) with the protein kinase inhibitors U0126 (10 μM) or AG1478 (10 μM) for 30 min, and then stimulated with EGF (150 ng/ml) for the indicated times. Cells were harvested, washed twice with cold phosphate-buffered saline and lysed with either 2x Laemmli sample buffer (Sigma), for protein extraction, or miRVANA lysis/binding buffer (Ambion), for total RNA extraction following manufacturer’s instructions.

For transfections, HeLa cells were cultured as indicated above and transfected with either an empty plasmid (pcDNA), or with the plasmid encoding a constitutively active form of Ras (pcDNA-RasV12) with Lipofectamine 2000 (Invitrogen) following the manufacturer’s instructions. After transfection, cells were cultured for 24 h in DMEM containing 0.5% FBS.

Total RNA was quantified with NanoDrop ND-1000 followed by quality assessment with 2100 Bioanalyzer (Agilent Technologies) Nano 6000 assay according to the manufacturer’s protocol. Acceptable A260/A280 ratios were in the range 1.8-2.2. Acceptable rRNA ratio (28S/18S) had to be >0.9 and RIN (RNA Integrity Number) value has to be >8.0.

### Western blot

For Western blotting 50 μg of cell extracts from HeLa cells were subjected to 8-10% SDS-PAGE. Gels were transferred onto PVDF membranes and processed for specific immunodetection by ECL (Pharmacia) following manufacturer’s instructions using antibodies as indicated above.

### Exiqon microarrays

One μg of total RNA from sample (individual EGF treated or untreated control) and reference (pool of three control samples) were labeled with Hy3™ and Hy5™ fluorescent label, respectively, using the miRCURY™ LNA Array power labeling kit (Exiqon, Denmark) following the procedure described by the manufacturer. The Hy3™-labeled samples and a Hy5™-labeled reference RNA sample were mixed pair-wise and hybridized to the miRCURY™ LNA array version 9.2 (Exiqon, Denmark), which contains capture probes targeting all miRNAs for all species registered in the miRBase version 9.2 at the Sanger Institute. The hybridization was performed according to the miRCURY™ LNA array manual using a Tecan HS4800 hybridization station (Tecan, Austria). After hybridization the microarray slides were scanned and stored in an ozone free environment (ozone level below 2.0 ppb) in order to prevent potential bleaching of the fluorescent dyes. The miRCURY™ LNA array microarray slides were scanned using the Agilent G2565BA Microarray Scanner System (Agilent Technologies, Inc., USA) and the image analysis was carried out using the ImaGene 7.0 software (BioDiscovery, Inc., USA).

### Agilent microarrays

Total RNA (0.5 μg) was dephosphorylated with CIP at 37°C for 30 min, samples were denatured and ligation was carried out for 2 hours at 16°C, where a molecule of Cyanine 3-pCp is incorporated to the 3′ end of RNA molecules. Labeled RNA was dried, resuspended with Hybridization Buffer and Blocking Agent, incubated 10 min at 100°C and transferred to an ice water bath for 5 min. Samples were hybridized in a volume of 45 μl to the Human miRNA V2 Oligo Microarray (Agilent) for 20 hours at 55°C with 20 rpm rotation. Post-hybridization washes were in GE Wash Buffer 1 (Agilent) at RT to remove the cover slip, followed by one wash with GE Wash Buffer 1 (Agilent) at RT for 5 min and one wash with GE Wash Buffer 2 (Agilent) at 37°C for 5 min.

Arrays were scanned on an Agilent G2565BA microarray scanner under default settings recommended by Agilent Technologies for miRNA microarrays at 100% PMT setting and 5 μm resolution. Image derived raw intensity data was extracted using Agilent Feature Extraction Software (Agilent).

### Illumina miRNA sequencing

Starting from 1 μg of total RNA, small RNAs in the range of 18–30nt were separated by 15% Novex TBE-urea PAGE, excised from the gel, and eluted out of the gel slice. 5′ RNA adapters (5′-GUUCAGAGUUCUACAGUCCGACGAUC-3′) were ligated using T4 RNA ligase, and ligated fragments in the range of 40-60nt were separated and recovered as before. Thereafter, 3′ RNA adapters (5′-UCGUAUGCCGUCUUCUGCUUGUidT-3′) were ligated to the RNA. Ligation products were isolated by 10% Novex TBE-urea PAGE, recovering fragments of 70-90nt. SuperScript II reverse transcriptase was used to generate cDNA constructs with the SRA RT primer (5′-CAAGCAGAAGACGGCATACGA-3′), from the small RNA ligated with 5′ and 3′ adapters. Single stranded cDNA with adapters at both ends were selectively amplified by 15-cycle PCR reaction using Phusion DNA polymerase and primers GX1 (5′-CAAGCAGAAGACGGCATACGA-3′) and GX2 (5′-AATGATACGGCGACCACCGACAGGTTCAGAGTTCTACAGTCCGA-3′). Amplified cDNA was resolved by 6% Novex TBE PAGE and amplicon fragments of 92 nt were recovered as before. Library quality was assessed on the Agilent Technologies 2100 Bioanalyzer. DNA was loaded into a lane of a single-read flow cell at a concentration of 3–3.5 pM for cluster generation using a single-read cluster generation kit (Illumina). The sequencing primer (5′-CGACAGGTTCAGAGTTCTACAGTCCGACGATC-3′) was annealed to the clusters and the flow cell was then mounted on a Genome Analyzer (GA) I or GA II instrument for sequencing, and 36–41 sequencing cycles were performed. A PhiX control lane loaded at a concentration of 2 pM was used to monitor run quality. Image processing and base calling was performed using Illumina sequencing analysis pipelines v0.3.0 or v1.3.2. Comment: replicate 1 was sequenced in May 2008; replicates 2 and 3 were sequenced in December 2008. Some of the technicalities had changed (upgrade of GA I to GA II, different pipeline versions, different number of sequencing cycles). Raw sequencing data were further processed to specifically analyze miRNA sequences using the MIRO software (http://seq.crg.es/download/software/Miro/) (Koffler et al., unpublished). 3′ adapters were recognized and trimmed using a perl script that penalizes mismatches to a lesser extent at read ends, following the distribution of sequencing errors along Solexa reads [19]. Where no adaptor could be recognized, sequence was cut down to 22 nt. The compiled collection of reads with removed adapters was aligned against the reduced-complexity set of miRBase version 12.0 entries (including miRBase primary miRNA, mature miRNA and star miRNAs). The mapping was performed by applying Eland iteratively in order to include all possible product sizes. Reads mapping unambiguously were counted for each unique miRBase entry within the reduced-complexity miRBase reference set. The per-miRNA count data from Illumina sequencing was normalized according to estimated effective library sizes [20].

### miRNA RT-qPCR

Quantitative real time PCR was performed using the miRCURY LNA™ microRNA PCR System (Exiqon) on total RNA extracted from HeLa cells treated at different times with EGF (with or without protein kinase inhibitors) with miRVana’s isolation kit (Ambion) following the manufacturer’s instructions. PCR amplification and detection were performed with the ROCHE LightCycler 480 detector, using 2x SYBR GREEN Master Mix. The reaction profile was: Polymerase Activation/Denaturation cycle (95°C for 10 min) followed by 40 amplification cycles (95°C-10 sec, 60°C-20 sec). miRNA levels were calculated using the LightCycler 480 software. The data analysis was carried out using the ∆∆Ct method that provides the target gene expression values as fold changes in the problem sample compared to a calibrator sample. Both problem and calibrator samples were normalized by the relative expression of housekeeping genes (SNORD44 and SNORD48).

### Analysis of miRNA variability (isomiRs)

Deep characterization of miRNA was performed using a stand-alone version of the Seqbuster software (http://estivill_lab.crg.es/seqbuster; [21]). Several filters were applied for miRNA variant analysis. First, the sequences considered in the analysis presented a frequency above 3. Second, 10 was chosen as the ‘Contribution Cut-Off’ parameter, meaning that every isomiR considered in the analysis contributes by more than 10% to the total number of variants annotated in the same miRNA locus. The contribution cutoff was 10% to highlight the most abundant species annotating onto a specific miRNA locus, as previously shown [22]. However, the results did not significantly differ when using a decreased contribution cutoff. Third, we applied the Z-score option to exclude sequencing errors as the possible cause of the nucleotide changes observed in some variants [22].

### Statistical analysis of differential miRNA expression

Log2ratio values were computed for all pairs of control and EGF stimulated samples. Analysis for differential expression on a miRNA-by-miRNA basis was performed using SAM and limma (SAM: [23], limma: [24]), including correction for multiple testing using the False Discovery Rate (FDR) method.

We also aimed at defining a consensus list of regulated genes using information from all platforms simultaneously. Since expression measures are not directly comparable between different platforms we used the Rankprod approach [25] that is based on log2ratio ranks. Only genes present in all the platforms under consideration can be included in each analysis. Therefore we applied the Rankprod analysis for all combinations of platforms as given by the complete merge data matrix. p-value adjustment according to [26] (FDR) was then applied to the union of all genes.

### Network and pathway analysis

Ingenuity pathway analysis 3.1 software (IPA; Ingenuity Systems) was used for evaluating the functional significance of regulated gene profiles. Protein coding genes identified by at least 3 of 4 miRNA target prediction methods (TargetScan 4.0, PicTar, miRBase, and miRanda) as hypothetical targets of the EGF responsive miRNAs were used for network inference and pathway analyses implemented in IPA tools. The list of corresponding HUGO official gene symbols was uploaded into the IPA web tool, and each gene was mapped to the Ingenuity Pathway Knowledge Base. Significant interaction networks were generated by IPA for genes found together with higher likelihood than by random chance. Using a 99% confidence level, IPA network scores ≥2 (reflecting the negative logarithm of *P* values <0.01) were considered significant. Significances for biological functions were then assigned to each network by determining a *P* value for the enrichment of the genes in the network for such functions compared with the whole Ingenuity Pathway Knowledge Base as a reference set.

### Permutation testing for enrichment in experimentally verified miRNA targets

We used the miRTarBase database [27] to call miRNA targets. This database collects all validated miRNA targets that have been published up to November 2012.

We used the list of de-regulated genes after EGF treatment published in our previous work [16]. All mRNA targets from the 6 h EGF-regulated miRNAs were called from the database and crossed with the de-regulated gene list, dividing between up-regulated and down-regulated genes.

To estimate whether the proportions of target genes in these two lists were statistically significant, we compared them to simulated data that was generated with 400 permutations. In this way, we produced a random distribution of expected numbers of genes overlapping down-regulated and up-regulated genes. We used two different strategies to generate random target genes: 1) we randomly picked up as many miRNAs from the miRTarBase list as the real number of de-regulated miRNAs (8), and retrieved their targets; and 2) we randomly picked as many genes as those from the 8 de-regulated miRNAs (168). Then we assigned the p-value to each analysis according to this equation:

$$p=\frac{\sum_{i-1}^{N}e>o}{N}$$

where e is the number of miRNA target genes in the de-regulated (up and down) gene list coming from the simulated data in each permutation, o is the number of miRNA target genes in the de-regulated (up and down) gene list coming from the real data, and N is the number of permutations, in this case 400.

## Results

### Microarray cross-platform comparison

**Figure 1:**
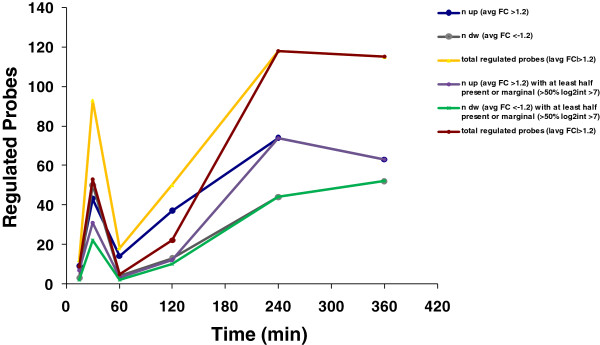
**Time-course miRNA transcriptome profile of EGF-treated HeLa cells using Agilent miRNA arrays.** Agilent miRNA microarrays were used to analyze miRNA expression in HeLa cells treated with EGF at the indicated time points. Average differential miRNA expression between EGF and control was calculated and the number differentially regulated probes (FC > 1.2) was plotted for each time-course point. Different lines show numbers of probes found: up-regulated (blue and purple), down-regulated (gray and green), regulated up or downwards (yellow and brown). Blue-gray-yellow: all regulated probes, without filtering for intensity; purple- green-brown: only regulated probes when at least half of the samples had log2intensity >7 (arbitrary detection limit to consider a miRNA as not absent).

We hypothesized that miRNAs are key to signaling processes such as the growth factor proliferative response to EGF. Serum-starved HeLa cells were EGF stimulated (15 min, 30 min, 1, 2, 4 and 6 hour EGF treatments). Total RNA was extracted and samples were analyzed with Agilent miRNA microarrays. Plotting the number of probes with a Fold Change ≥ 1.2 at for each time point shows a peak in the number of regulated miRNAs at 30 min after EGF stimulation and a second peak at 4 hours maintained at 6 hours. The highest number of miRNA with changes in expression relative to baseline was found to be at 4 and 6 hours after EGF stimulation (Figure 1). We focused on the 6 hour time point to perform a cross-platform validation study in order to establish a robust set of miRNAs regulated upon EGF stimulation of serum-starved HeLa cells. Three independent experiments were performed where cells were serum-starved for 24 hours and then stimulated with EGF for 6 hours. In order to ensure a correct activation of EGF-related pathways in our samples, activation of ERK and AKT was checked with phospho-specific antibodies (see Additional file 1). Total RNA from EGF-treated and control samples were extracted. Using miRNA microarrays we evaluated miRNA expression profiles EGF treated HeLa cells compared to their respective untreated controls in triplicate with two different commercial miRNA platforms (Exiqon and Agilent). All data from this study can be accessed at the National Center for Biotechnology Information, Gene Expression Omnibus with the GEO ID (GSE41360).

**Figure 2:**
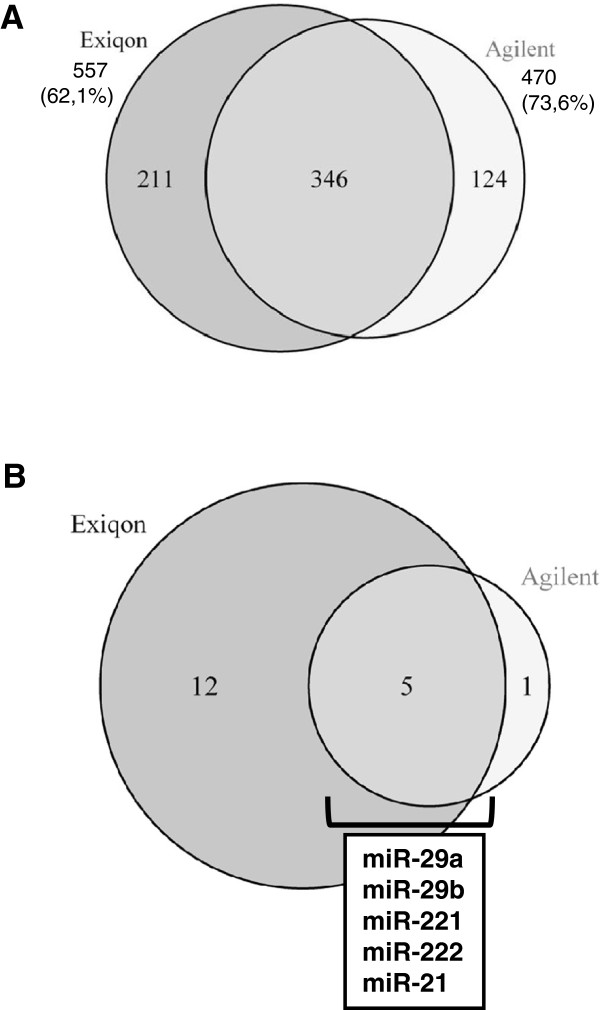
**miRNA transcriptome profiles of EGF-treated HeLa cells using Exiqon and Agilent miRNA arrays. A**. Shared miRNA genes between Exiqon and Agilent microRNA arrays. Venn diagram showing the unique and named miRNAs genes shared between Agilent and Exiqon microarray platforms. The pool of 346 shared genes was used for all subsequent cross-platform analyses. Numbers outside diagrams indicate the total amount of miRNA genes contained in each platform. In brackets, percentage of miRNA genes contained in each platform over the total miRNA genes contained in all platforms. **B**. Regulated miRNAs after 6 hours EGF treatment using Exiqon and Agilent miRNA arrays. HeLa cells were serum-starved for 24 h and treated with EGF for 6 h. Total RNAs prepared from cells lysates were hybridized to Exiqon miRCURY LNA microRNA Array V9.2 and Agilent Human miRNA V2 Oligo Microarray. List shows regulated miRNAs identified by SAM analysis (FDR = 0.05) for miRNAs with a minimal expression change of 1.2 fold.

In order to assure hybridization quality and reproducibility within each microarray platform, we computed correlation coeficients; showing distinct performances for each platform in the range of 0.8 to 0.99 (see Additional files 2 and 3). Agilent and Exiqon platforms have a total of 346 miRNAs profiled in common which represent 62.1% and 73.6% of the total of miRNAs represented on each platform, respectively (Figure 2A). To identify differentially expressed miRNAs on the chips, SAM analysis was applied to generate a list of significantly regulated miRNAs at FDR = 0.05. Exiqon identified 17 regulated miRNAs and 6 regulated miRPlus (proprietary microRNAs sequences not included in miRBase that have been identified by cloning and sequencing in human disease or normal tissues (http://www.exiqon.com/array) that have not been included in further analyses). Instead, Agilent only identified 6 regulated miRNAs. Initial comparison between platforms indicates a good correlation, with 5 common regulated hsa-miRNAs: miR-21, miR-221, miR-222, miR-29a and miR-29b, all of them being up-regulated after EGF stimulation (Figure 2B).

The fact that Agilent platform only showed 6 regulated genes suggests this platform may be less sensitive than the Exiqon one. Indeed the Exiqon platform allowed us to detect double the regulated genes than the Agilent platform (2.8% vs. 1.27%) from the total of mRNAs represented on each platform.

### Illumina deep sequencing

The main limitation of array technologies is that the set of regulated genes that can be found is reduced due to the constrained number of probes included in each platform. In our experimental design, miRNAs not covered by both microarray platforms had a higher likelihood of being false positives or false negatives, with a very small regulated set of miRNAs found shared among both platforms. The only way to extend the validation without being limited by the probe content of each platform was to use of deep sequencing, which could fix the lower resolution associated to microarray measurements and should allow a cross-platform validation using different technologies. For this purpose, we used the small RNA-seq methodology developed by Illumina. We re-analyzed aliquots of total RNA from the exact same three replicate experiments that had been tested on microarrays, for the control (serum-starved) and the EGF treated at the 6 h time point.

On average, 5.5 million raw sequences were obtained per sample, which after running the MIRO analysis pipeline allowed us to monitor the expression of an average of 3.7 million unambiguous tags matching to mature miRNA with less than 3 mismatches, corresponding to 490 different miRNA genes (see Additional file 4). We found a good correlation between replicates with coefficients ranging from 0.85 to 0.96 (see Additional file 5).

### Extended comparison of microarray and small RNA-seq results

**Figure 3:**
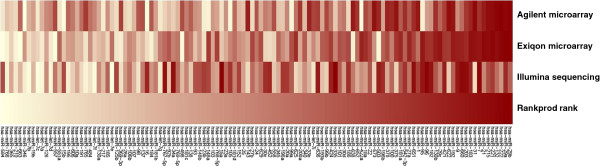
**Correspondence between Microarrays and Illumina sequencing.** In each platform, miRNAs were ranked based on EGF versus Control log ratios. The heatmap shows a color representation of those ranks, ranging from most up-regulated (dark red) to most down-regulated (light yellow) miRNAs. miRNAs are listed in decreasing Rankprod score, grouping genes that are most concordantly regulated across platforms at the top.

343 of the miRNAs measured by microarray could be detected through small RNA-seq (see Additional file 6). 121 miRNAs present in both microarray platforms had no detectable measure by small RNA-seq in any of the three biological replicates, whereas 147 detected by small RNA-seq had not been addressed by any of the microarray platforms. Using the Rankprod test [25], the three data sets were analyzed jointly for differential expression based on per replicate log2ratio rank order for all miRNAs that shared an expression measure. In the case where not all platforms had this measure the same was computed for the subset of platforms that had probes for the same miRNA (see Additional file 7). This global measure of statistical significance was computed in order to get at a consensus set of regulated genes. The results of the Rankprod analysis, for those miRNAs present in all platforms, are shown as a heatmap of color-coded ranks of log ratios within each platform (Figure 3). As would be expected, the concordance is higher between the microarray platforms. The miRNAs with best Rankprod scores are found highly up-regulated with both Agilent and Exiqon arrays. Even though overall there is a relatively low concordance between microarrays and Illumina data in terms of log ratio ranks, with the Rankprod analysis, it is possible to define a statistically significant consensus list of 8 miRNAs that are up-regulated in response to EGF at 6 h (Rankprod test adjusted p-value: p < 0.05, mean absolute fold change of all measurements: |FC| > 1.2) (Table 1).

**Table 1:** Top concordant regulated miRNAs across different platforms

**Index rank**	**hsa-miR**	**Agilent**	**Exiqon**	**Illumina**	**Adj. p-value**	**RT-qPCR**
1	**hsa-miR-29b**	1.54	1.40	2.62	0.00	2.73
2	**hsa-miR-29a**	1.33	1.36	4.90	0.00	3.37
3	**hsa-miR-222**	1.58	1.32	1.85	0.00	2.56
4	**hsa-miR-132**	1.47	1.19	2.46	0.01	2.27
5	**hsa-miR-221**	1.26	1.31	3.94	0.01	3.31
6	**hsa-miR-215**	1.20	1.22	3.09	0.18	2.35
7	**hsa-miR-21**	1.11	1.25	2.02	0.35	2.44
8	**hsa-miR-7**	1.06	1.12	16.75	0.43	1.63

Rankprod analysis identified 8 miRNAs that were found to be concordantly up-regulated in both microarray platforms (Agilent, Exiqon) and by small RNA-seq sequencing (Illumina). The table shows fold changes for each platform and corresponding Rankprod adjusted p-values. Additionally, these miRNAs were validated by RT-qPCR, with relative expression fold changes provided in the last column.

### RT-qPCR Validation

**Figure 4:**
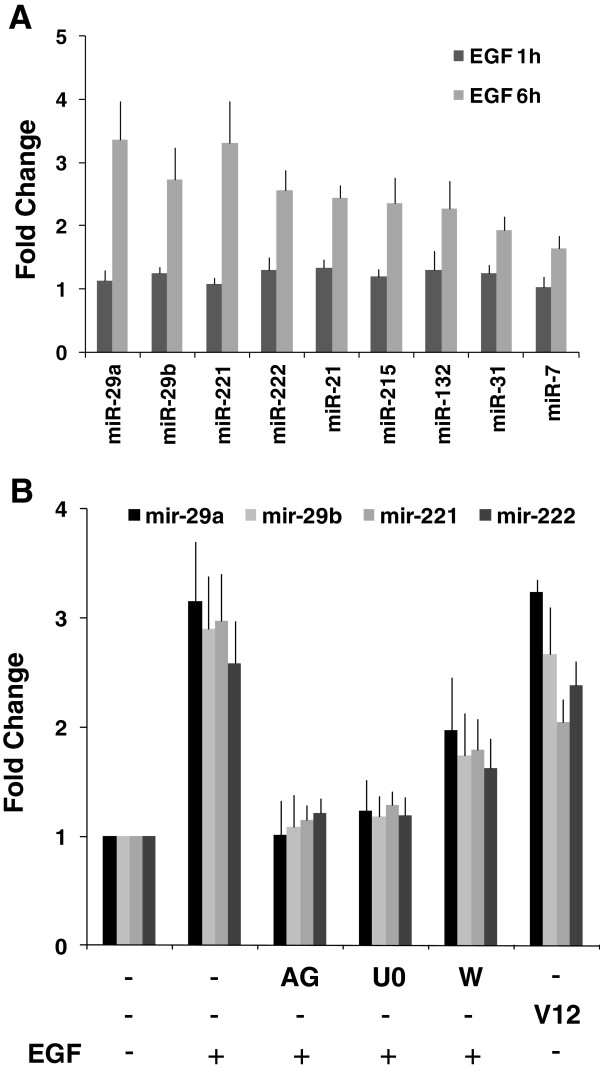
**RT-qPCR validation of EGF-regulated miRNAs.** Total RNA prepared from cells lysates were analyzed by quantitative real time PCR using the miRCURY LNA™ microRNA PCR System (Exiqon) for each of the miRNAs as indicated. **A**. Control treatment without inhibitors: HeLa cells were serum-starved for 24 hours and treated with EGF for 1 and 6 hours. **B**. Inhibitor treatment. HeLa cells were serum-starved for 24 hours and treated with EGF for 6 hours in the presence or absence of protein kinase inhibitors: AG1470 (EGFR inhibitor), U0126 (MEK inhibitor) and Wortmannin (PI3K inhibitor). In addition, HeLa cells were transfected with a constitutively active form of Ras (RasV12). Effective pathway inhibition was verified by western blotting in parallel samples from the same experiment (see Additional file 8).

In order to validate the miRNAs selected by Rankprod analysis we measured the relative expression of the 8 most significant differentially expressed miRNAs at 1 hour and 6 hours after EGF treatment with the miRCURY LNA™ microRNA PCR System (Exiqon). This system uses the LNA technology allowing an accurate and sensitive quantitation of mature microRNAs [28]. In agreement with microarrays, and Illumina ultrasequencing data, all the hsa-miRNAs tested (miR-29a, miR-29b, miR-221, miR-222, miR-21, miR-132, miR-215 and miR-7) were found to be up-regulated at 6 hours but not at 1 hour after EGF treatment (Figure 4A). Interestingly, we also validated miR-31, the next miRNA top hit in the Rankprod analysis. miR-31 is also up-regulated after 6 hours EGF treatment (Figure 4A).

Upon EGF activation, multiple pathways are engaged including Ras/ERK MAPK, PI3K/AKT [1,2]. To evaluate the role of these signaling pathways on EGF-dependent miRNA regulation, selective pharmacological inhibitors were tested for their effect on the expression changes of the four most up-regulated miRNAs in response to EGF treatment. In order to evaluate the effects of protein kinase inhibitors used in this study, as well as the effect of RasV12 transfection on ERK and AKT activation, western-blotting assays against phospho-ERK and phospho-AKT were performed (see Additional file 8). To assess the involvement of transactivation of EGFR in EGF-induced miRNAs expression, we used the specific EGFR tyrosine kinase inhibitor, AG1478. As expected, pre-treatment of HeLa cells with AG1478 abolished EGF-induced tyrosine phosphorylation of EGFR (data not shown) and totally blocked EGF-induced miR-29a, miR-29b, miR-221 and miR-222 expression (Figure 4B). The ERK inhibitor U0126 also effectively blocked overexpression of all 4 miRNAs bringing them to levels equivalent to AG1478 inhibitor treatment. In contrast, the PI3K-AKT pathway inhibitor, wortmannin, only partially blocked miRNA up-regulation provoked by EGF. Furthermore, transfection of cells with RasV12, a constitutively active Ras mutant, which activates the ERK pathway, is sufficient to promote miR-29a, miR-29b, miR-221 and miR-222 overexpression without the addition of EGF.

### Inferred biological function of EGF-regulated miRNAs

To explore the hypothetical biological functions of EGF-regulated miRNAs we looked for the predicted genes targeted by these miRNAs. Since the reliability of the miRNA prediction methods is very discrepant [10] our target prediction was based on a compilation of methods (TargetScan 4.0, PicTar, miRBase, and miRanda) (see Additional file 9). The list of the genes predicted by at least three of the four prediction programs was uploaded onto the IPA (Ingenuity Pathway Assist) software to integrate genomic data with mining techniques, allowing us to infer protein networks and biological functions associated to miRNAs regulated by EGF. IPA analysis indicated that the potential targets for our set of regulated miRNAs are implicated in molecular functions related to EGF signaling such as cellular development, growth and proliferation; cell morphology and cell death and cell-to-cell-signaling and interaction [29], (Table 2). These results are very similar to the ones we found when we analyzed the EGF-dependent mRNA transcriptome using a similar genome wide cross-validation approach [16] reinforcing the idea that the miRNAs regulated by EGF described in the present work play an important role in EGF signaling. In the same direction, most over-represented networks detected through IPA analysis converged on two hub proteins, MAPK and NFK-B, which are two of the most important kinases activated downstream of the EGF signaling pathway after mitogenic stimulation with EGF (Data not shown).

**Table 2:** Ingenuity pathways analysis

**Diseases and disorders**		
**Name**	**p-value**	**Molecules**
Genetic Disorder	4,26E-09 - 1,42E-02	139
Cancer	2,72E-05 - 1,42E-02	91
Connective Tissue Disorders	4,26E-09 - 1,42E-02	57
Developmental Disorder	2,22E-06 - 1,42E-02	32
Dermatological Diseases and Conditions	3,49E-08 - 1,42E-02	29
**Molecular and cellular functions**		
**Name**	**p-value**	**Molecules**
Cellular Growth and Proliferation	5,25E-06 - 1,42E-02	75
Gene Expression	1,28E-08 - 1,42E-02	67
Cellular Development	6,24E-09 - 1,42E-02	63
Cell Cycle	2,80E-06 - 1,42E-02	32
DNA Replication, Recombination, and Repair	2,71E-06 - 1,42E-02	15

List of Ingenuity Networks and Biological Functions from predicted targets of miRNA differentially expressed during EGF treatment.

**Figure 5:**
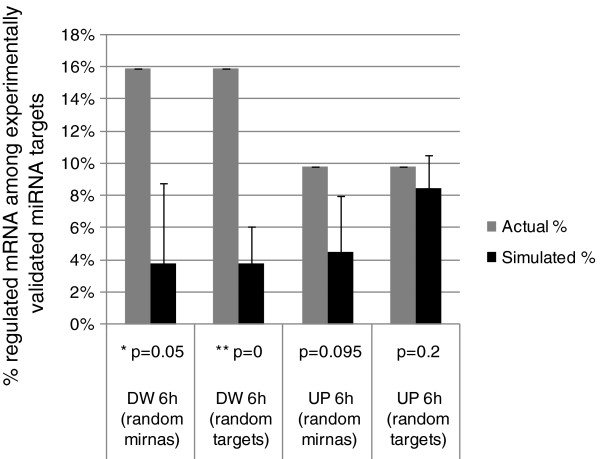
**Significant enrichment in mRNAs down-regulated by EGF among experimentally validated targets of EGF-dependent miRNAs.** Plot comparing the percentage of observed versus randomly expected mRNAs regulated by EGF that are known experimentally validated targets of the nine miRNAs shown to be regulated by EGF in this study. Actual % (gray): experimentally observed; Simulated % (black); result of random permutations; DW 6 h: down regulated mRNAs at 6 h; UP 6 h, up-regulated mRNAs at 6 h. Two randomization tests were performed: choosing as many (n = 8) miRNAs as those found regulated (random miRNAs) and looking at their targets, or choosing as many target mRNAs (n = 168) among the experimentally validated targets of the entire miRTarBase.

To further verify that the processes found regulated were consistent with real data and not just target predictions, we tested for enrichment among the experimentally validated targets (as compiled in the miRTarBase database [27]) for the regulated miRNAs among the robust dataset of differentially expressed mRNA that we had described in prior work [16]. Testing revealed that there was a significant enrichment in experimentally verified miRNA targets of the set of 8 EGF dependent miRNAs described in this work (Table 1) among the mRNA found to be down-regulated in HeLa cells after 6 h EGF treatment (Figure 5). This was not the case among up-regulated mRNAs in agreement with a possible mechanism of action were miRNAs may be acting through destabilization of their target mRNAs leading to decreases in steady state levels of mRNA.

### Sequence heterogeneity in the EGF-dependent miRNome

**Figure 6:**
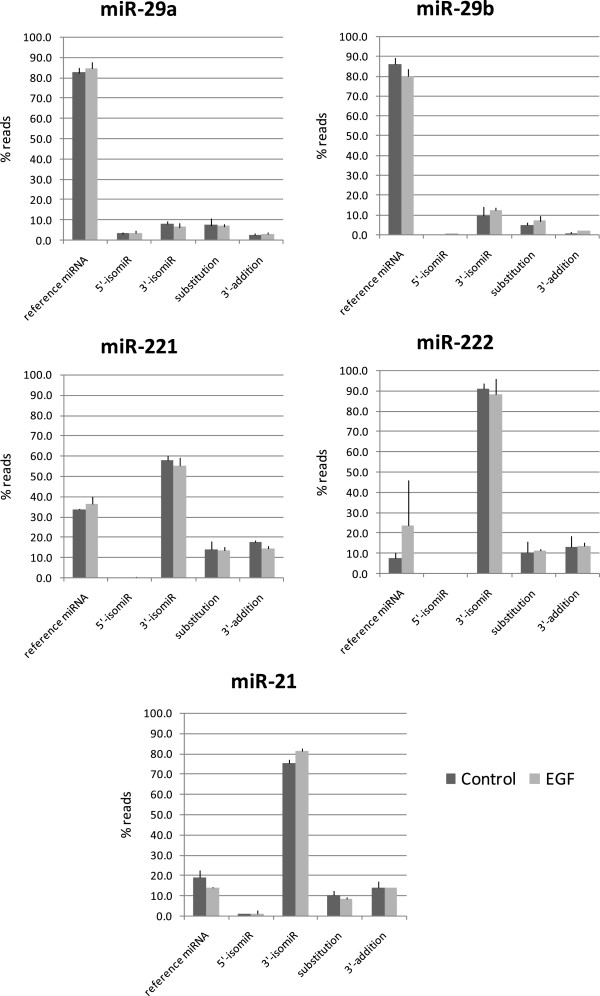
**miRNA heterogeneity in EGF-treated HeLa cells.** Distribution of the different types of sequences mapping to the indicated miRNA loci. The percentage of sequences annotated as the reference-miRNA (Ref), and as the different types of isomiRs is shown in control cells (dark gray) and EGF treated cells (light gray). IsomiRs consist in trimming variants at the 3’ end of the reference miRNA (3’-trim), trimming variants at the 5’-end of the reference miRNA (5’-trim), variants showing changes in the nucleotide composition with respect the reference miRNA (subst) and variants consisting in nucleotide additions to the 3’ end of the reference miRNA (3’-add). Data are presented as the mean and the standard deviation of three independent experiments per condition.

Next we compared the distribution of miRNA sequence variants (isomiRs) in serum-starved and EGF-treated HeLa cells for the top EGF-regulated miRNAs (see Additional files 10 and 11). We observed that all the miRNAs analyzed present sequence variations scored as 5′trimming, 3′trimming and substitutions. For two of the miRNAs (miR-29a and miR-29b) the most abundant sequence matched with the mature sequence listed in miRNA databases such as miRBase (http://www.mirbase.org), with a residual presence of variant sequences with 5′ trimming, 3′ trimming, substitutions and 3′ additions. On the contrary, for miR-221, miR-222 and miR-21 the most abundant sequences where those with 3′ trimming variations (Figure 6).

IsomiR expression profiles between the serum-starved and the EGF-treated cells present a high correlation for all the regulated miRNAs suggesting that EGF is not involved in the molecular mechanisms of biosynthesis leading to sequence heterogeneity of the miRNAs analyzed.

## Discussion

### Cross-platform validation

The results of profiling miRNA using genomic methods have proven to be very platform dependent. This can be explained by differences the probe design methods which at times address all possible isoforms or sometimes just target one very specific isoform.

Some of the differences observed between Agilent and Exiqon may be related to this fact. Agilent arrays use hairpin probes that require a perfect match of the 3′ end of the probe, therefore being very specific for the major reference mature isoform and less likely to detect isomiRs different from the reference isoform. On the other hand, Exiqon probe design in principle could cross-hybridize to pre-miRNA and different isomiRs from a specific class. This could explain why more miRNAs show differential expression in Exiqon. For example, miR-21 was not found to be differentially expressed on the Agilent platform, and this could be explained by the fact that the miRBase reference sequence interrogated by the hairpin loop probes is the minor isoform in HeLa cells.

Small RNA-seq is a powerful methodology that provides a qualitative improvement on the microarray technology. We have shown that it is able to detect changes similar to those detected by microarrays, as well as revealing the underlying isoform complexity that cannot be captured using microarray based assays.

In our hands, both platform approaches, high-throughput sequencing and microarrays, are complementary techniques and not mutually exclusive. We observed high agreement between both strategies for the highest ranked targets using Rankprod, which were further cross-validated using RT-qPCR.

We compared our results with those reported in a recent study describing the temporal response to EGF treatment of HeLa cells and found little overlap (Avraham et al., 2010). We believe this is due to the fact that in our study we compare the treated and untreated transcriptome at the time of interest whereas the experimental design of this other work compares to a baseline at the onset of treatment. Whereas our design would focus on the specific EGF dependent response, the comparison to the baseline may be biased toward temporal rather than treatment specific responses. In agreement with this, subtraction of control 15 minute time point in our dataset showed similar temporal profiles for most genes with or without exposure to EGF, indicating that exposure to reduced serum used in EGF exposure experiments may account for most of the responses reported previously, and therefore the response attributed to EGF in the work of Avraham may be confounded. This would explain why in our hands the number of EGF responsive miRNAs may be reduced relative to theirs.

In agreement with our observations, recent results derived from lung cancer cell lines where EGFR transcripts were targeted by shRNA expression resulted in miRNA targets highly overlapping with our own targets (including miR-221, miR-222, miR-29a,b,c) also found to be targets of MET tyrosine kinase receptor [18]. At the functional level this would identify such targets as core to EGFR (and MET) tyrosine kinase signaling in other cell types.

### EGF-dependent miRNAs in cancer

EGF treatment leads to the activation of a network of signaling pathways which leads to the up-regulation of anti-apoptotic proteins or to the inactivation of pro-apoptotic proteins. The post-transcriptional and expression events implicated in these pathways have been a matter of intense study during the last decade [5-7,16]. A critical question about signal transduction is how a weak or transient activation achieves a robust and long-term switch in gene expression. The discovery of miRNAs opened a new door in the study of the regulation of the downstream events related to these pathways that may help disclose some of these open questions, such us the inhibitory mechanisms leading to attenuation of gene expression [15]. This hypothesis is supported by the fact that Gene Ontology analysis indicates that several of the genes targeted by the up-regulated miRNAs after 6 hours of EGF treatment in our study are involved in the mechanisms that down-regulate the MAP kinase pathway after a mitogenic stimulus.

Recent studies have emphasized causative links between microRNA deregulation and cancer development. In fact, many oncogenes and tumor suppressor genes are regulated by miRNAs ([8,30-33] for review) and significant down-regulation of most of the miRNAs has been reported in various tumors as compared to normal tissues [34].

Few studies so far have focused on the potential link between EGF signaling and miRNA [7,17] although a direct link between EGF signaling and miRNA biosynthesis has been described. Argonaute-2 expression is regulated by epidermal growth factor receptor and mitogen-activated protein kinase signaling and correlates with a transformed phenotype in breast cancer cells [35] and miR-7 has been found to mediate EGF receptor signaling and promote photoreceptor differentiation in the Drosophila eye [36]. In addition, miR-7 down-regulates EGFR mRNA and protein expression in cancer cell lines (lung, breast, and glioblastoma) and the AKT pathway [37,38] inhibiting schwannoma cell growth [39] and inducing cell cycle arrest and cell death, which suggests a negative regulatory mechanism acting on the MAPK pathway [40]. Furthermore, increasing evidence shows that the links between microRNA deregulation and cancer development may be often mediated through the MAPK pathway [41-44]. Interestingly, the 8 miRNAs described to be up-regulated in the present study had already been described to be involved in several processes such as cell growth and proliferation, cell cycle and cellular development among others, besides their implication in several types of cancers.

In vitro studies indicate that miR-21 promotes proliferation and invasion in cultured cells [45]. miR-21 has also been found to be overexpressed under many pathophysiological conditions, especially in cancer ([46] for review). Indeed, miR-21 has been implicated in various aspects of carcinogenesis, including cellular proliferation, apoptosis, and migration [47]. miR-21 is up-regulated in breast cancer [48] and this overexpression is correlated with specific breast cancer biopathological features such as advanced tumor stage, lymph node metastasis and poor survival of the patients. miR-21 is also up-regulated in pancreatic cancer [49] and in hepatocellular carcinoma (HCC) tumors [50]. Furthermore, an aberrant expression of miR-21 can contribute to HCC growth and spreading by modulating Phosphatase and TENsin homolog (PTEN) expression which mediates phenotypic characteristics of cancer cells such as cell growth, migration and invasion [51].

miR-21 has been also found to target tumor suppressor Programmed Cell Death 4 (PDCD4) and stimulates invasion, intravasation and metastasis in colorectal cancer [52] and its inhibition in breast cancer cells causes reduced cell growth [49]. Interestingly PDCD4, suppresses tumor progression in human colon carcinoma cells by down-regulating the MEK kinase kinase 1 (MAP4K1) gene transcription [53], with the consequent inhibition of c-Jun activation and Activator Protein-1 (AP-1)-dependent transcription. Given the role of PDCD4 as a negative regulator of AP-1, the miR-21-mediated down-regulation of PDCD4 seems to be essential for the maximal induction of AP-1 activity in response to RAS transformation [54]. Interestingly we have also found that c-jun expression was down-regulated after 6 h of EGF treatment [16]. Recent studies have implicated the down-regulation of the tumor suppressor gene acidic (leucine-rich) nuclear phosphoprotein 32 family, member A (ANP32A) [55] and Spry2 [56] of miR-21 to its oncogenic function.

miR-29 regulates the anti-apoptotic Bcl-2 family member, Mcl-1 [57] and DNA methyltransferases (DNMT) 3A and 3B [58] and is down-regulated in lung cancer where DNMT is frequently up-regulated. In addition, several studies have implicated miR-29 in several types cancers such as acute myeloid leukemia [59], chronic lymphocytic leukemia [60], hepatocellular carcinoma [61] and cutaneous melanoma [62].

miR-132 is elevated in pancreatic cancer [56] and in the monocytic leukemia cell line THP-1 by Lipopolysaccharide (LPS) treatment. LPS triggers activation of NF-κB and AP-1 transcription factors and results in up-regulation of immune-responsive genes. REST (Re1 silencing transcription factor) and its cofactor complex are targets of miR-132 [63]. Given the role of both REST and miRNA as repressors, this suggests a double-negative feedback loop between REST and the miR-132 for stabilizing and maintaining neuronal gene expression. miR-132 is also induced by photic entrainment cues via a MAPK/CREB-dependent mechanism, modulates clock-gene expression, and attenuates the entraining effects of light [64].

miR-31 is a pleiotropic miRNA up-regulated in colorectal tumors and its expression levels are correlated with the stage of the tumor [65]. On the contrary, it has been found to be down-regulated greater than two-fold in breast cancer [48]. miR-31 also inhibits breast cancer metastasis [66] and over-expression of miR-31 significantly enhanced proliferation and tumorigenicity of lung cancer cells [67]. An interesting recently published study indicates that restoring miR-31 function in already-established metastases induces metastatic regression and opens the possibility of intervention strategies centered in restoring miR-31 function in order to combat metastasis [68].

Distinct up-regulations of miR-222, miR-221, and miR-31 have been observed in HCC [69]. miR-221 and miR-222 are potent regulators of proliferation, mediated by regulation of p27Kip, a cell cycle inhibitor and tumor suppressor [70]. High levels of miR-221 and miR-222 appear in human thyroid papillary carcinomas [71], prostate cancer [72] and in glioblastomas [36,73] and correlate with low levels of p27kip. Indeed, sustained activation of ERK1/2 by NGF induces miR-221 and miR-222 in PC12 cells [74].

Finally, miR-215 has been described to be a p53-responsive miRNA [75] able to induce cell cycle arrest. miR-215 suppresses Denticleless protein homolog expression, inducing a decreased cell proliferation by causing G2-arrest [76] and contributing to enhanced CDKN1A/p21 levels, colony suppression, cell cycle arrest, and cell detachment from a solid support [77].

Blocking of miR-29a, miR-29b, miR-221 and miR-222 expression by the selective MEK inhibitor U0126 in an analogous manner to AG1478 and the overexpression of these miRNAs in RasV12 transfected cells suggest that the MAPK pathway is a critical effector downstream of EGFR for the transmission of the information leading to miRNAs regulation. The same effect but to a lesser extent is observed after PI3K/AKT pathway inhibition with wortmannin. Indeed, both MAPK and PI3K/AKT pathways are activated by EGF-treatment controlling cell fates such as cell proliferation, survival and apoptosis [78,79]. Expression of most of the miRNAs regulated by EGF found in the present work induces a decrease (miR-7, miR-215) or increase in cell proliferation (miR-21, miR-221, miR-222, miR-31).

Further studies are required to identify their in vivo targets and to elucidate the specific regulatory networks where they are involved.

The results obtained from isomiR analysis show the presence of sequence heterogeneity for all the miRNA analyzed, supporting the idea of a novel degree of complexity for the miRNA transcriptome [67,80] as a result of inexact Dicer processing. Furthermore, some of the miRNA species studied have a majority of mature miRNA sequences that differ from that listed on databases. Lee et al. catalogued miRNA end heterogeneity from different biological samples and found that from 34% to 51% of the detectable miRNA species have a predominant sequence differing from that listed in databases [38]. However, the equal proportion of the different types of variants in serum-starved versus EGF-treated indicates that the molecular mechanisms involved in the generation of these variants are not regulated by EGF. Several studies already confirmed the presence of these variants in multiple and diverse kinds of samples, tissues and species, however, up to this date, there is no data regarding the functional role of isomiR diversity as well as their generation mechanisms besides a recent study suggesting that 5′ end variations may produce specificity changes in the type of AGOs with which these miRNA isoforms preferentially associate [81]. In addition a biological role for isomiRs is supported by the finding of regulation of variability in isomiR expression [82].

## Conclusions

In the present study we have used microarrays and deep sequencing analysis to present the EGF-induced miRNome and its integration into functional cellular networks.

Knowing that there are biases in genomic studies that are platform dependent, in our study we have attempted to get around this limitation to increase the confidence in the detected miRNome changes. By applying a procedure for cross-platform data tests we are able to present a dataset, also validated by RT-qPCR, which grants more reliable analyses at the functional genomics level and more robust predictions of networks of co-regulated microRNA target genes.

Specifically, we find that miR-29a, miR-29b, miR-221, miR-222, miR-21, miR-132, miR-215 and miR-7 are all up-regulated 6 hours after EGF treatment in HeLa cells, with an inferred effect on genes with a function in the regulation of cell proliferation, cell cycle, and DNA repair as well as gene expression. This supports a prominent role for these miRNA in modulating the effects of EGF on cancer-related cellular processes.

In addition, we make a comparison of performance between microarray and high throughput sequencing profiling that suggests the two methodologies combined may survey the miRNome in a better way than each on its own.

Finally, we observed the presence of sequence heterogeneity for all the EGF-dependent microRNAs, supporting the idea of a novel degree of complexity for the miRNA transcriptome. There is no apparent effect of EGF on isomiR regulation but we can confirm cell type specific differences in the prevalent edited isoforms relative to the miRBase reference miRNAs.

## Competing interests

The authors declare that they have no competing interests.

## Authors’ contributions

FL and LS conceived and designed experiments. FL and RP performed cell culture experiments and obtained biological samples. FL, AF, AV and EC performed microarray and deep-sequencing experiments. FL, MH, LP, XP, MI, JD, MN, RK, EM and LS analyzed data. MB, JADR and HH contributed reagents, materials and/or analytical/technical expertise, HM assisted with technical expertise. FL and RP performed RT-qPCR experiments. FL and LS wrote the paper. All authors read and approved the final manuscript.

## Supplementary Material

Additional file 1 **EGF activation of the MAPK pathway in HeLa cells.** Serum-starved HeLa cells were stimulated with EGF at the indicated times. Total cell extracts were prepared as indicated in Materials and Methods and samples were subjected to SDS-PAGE and immunoblotting using the indicated antibodies. Click here for file

Additional file 2 **Intensity correlation between replicate samples processed on Exiqon microarrays.** (A,B) Correlation of normalized log2intensities between biological replicates in the Control (A) and the EGF group (B) measured on Exiqon microarrays. Click here for file

Additional file 3 **Intensity correlation between replicate samples processed on Agilent microarrays.** (A,B) Correlation of normalized log2intensities between biological replicates in the Control (A) and the EGF group (B) measured on Agilent microarrays. Sample 1 was processed with two technical replicates ('1' and '1_b'). Click here for file

Additional file 4 Summary of read counts generated by the miRNA-seq MIRO analysis pipeline. Click here for file

Additional file 5 **Read count correlation between replicate samples processed by Illumina sequencing.** (A,B) Correlation of normalized, log2transformed read counts per miRNA between biological replicates in the Control (A) and the EGF group (B) measured by Illumina small RNA-seq. Click here for file

Additional file 6 **Numbers of miRNAs present in the different platforms.** The Venn diagram shows how many miRNAs are present on the two microarray platforms, how many were detected by Illumina sequencing, and the numbers of the respective overlaps. Click here for file

Additional file 7 Agilent, Exiqon and Illumina EGF versus control log2ratios, fold changes, statistical significance nominal and adjusted p-values, and ranks computed with Rankprod. Click here for file

Additional file 8 **Inhibition of the MAPK pathway in HeLa cells.** HeLa cells were serum-starved for 24 hours and treated with EGF for 6 hours in the presence or absence of protein kinase inhibitors: AG1470 (EGFR inhibitor), U0126 (MEK inhibitor) and Wortmannin (PI3K inhibitor). In addition, HeLa cells were transfected with a constitutively active form of Ras (RasV12). Lysates were analysed by Western-Blot against pospho-ERK1/2 and phospho-AKT to ensure protein inhibitors and transfection action over MAPK and AKT pathways. Tubulin was used as sample loading control. Click here for file

Additional file 9 List of 234 genes predicted in common by TS5.0 miRvana 3.0 and PICTar to be targets of the 8 miRNAs found to be regulated by EGF in this work. Click here for file

Additional file 10 Summary of isomiR counts for top EGF regulated miRNAs. Click here for file

Additional file 11 Listing of isomiR sequence variants and their respective read counts for top EGF regulated miRNAs. Click here for file
