# Supplementary figures and images for: Microarray and deep sequencing cross-platform analysis of the mirRNome and isomiR variation in response to epidermal growth factor
Source: BMC Genomics. 2013 Jun 1;14:371. doi: 10.1186/1471-2164-14-371 (PMC3680220; doi:10.1186/1471-2164-14-371)

## Slide 1
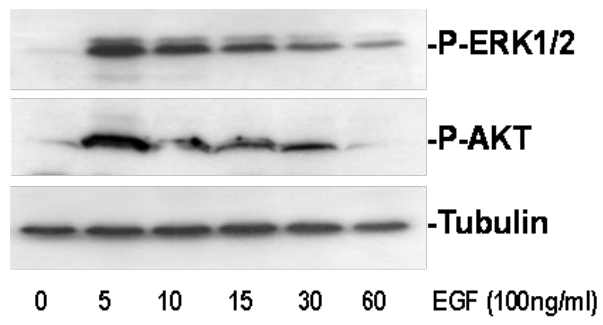

#

Supplement: Additional file 1 — EGF activation of the MAPK pathway in HeLa cells. Serum-starved HeLa cells were stimulated with EGF at the indicated times. Total cell extracts were prepared as indicated in Materials and Methods and samples were subjected to SDS-PAGE and immunoblotting using the indicated antibodies. [file 1471-2164-14-371-S1.pptx]

## Slide 1
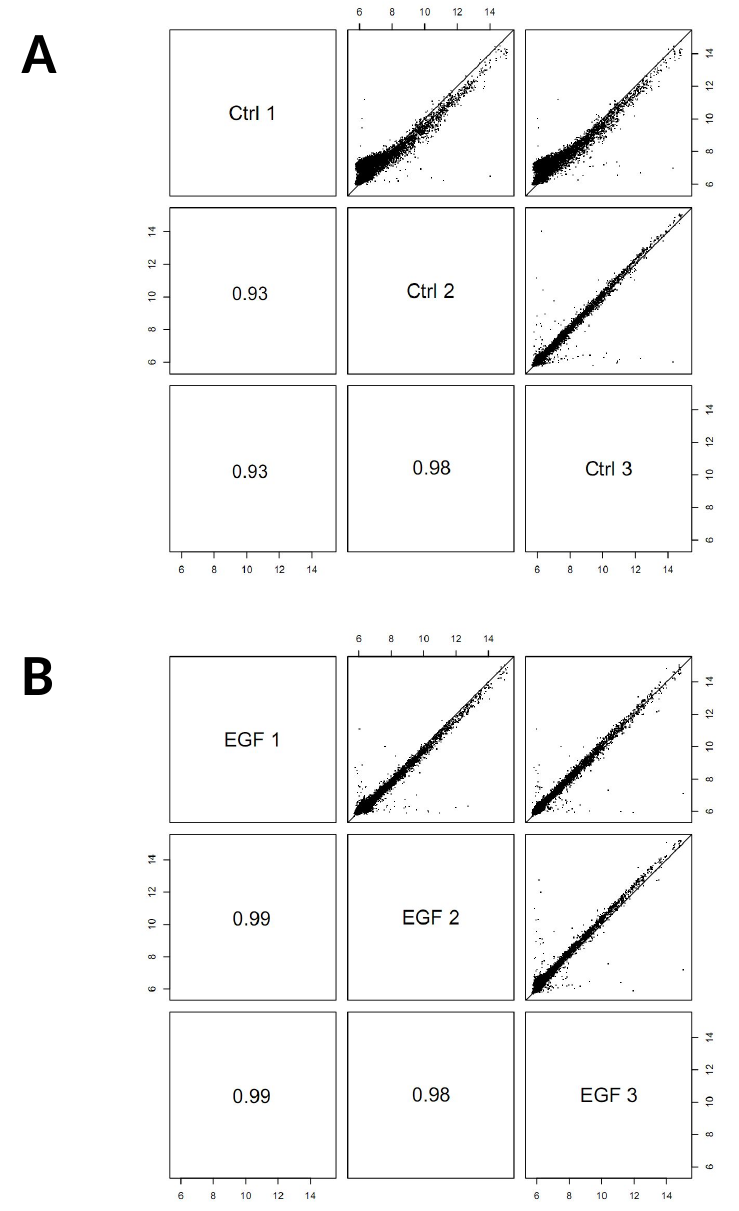

A
#
B

Supplement: Additional file 2 — Intensity correlation between replicate samples processed on Exiqon microarrays. (A,B) Correlation of normalized log2intensities between biological replicates in the Control (A) and the EGF group (B) measured on Exiqon microarrays. [file 1471-2164-14-371-S2.pptx]

## Slide 1
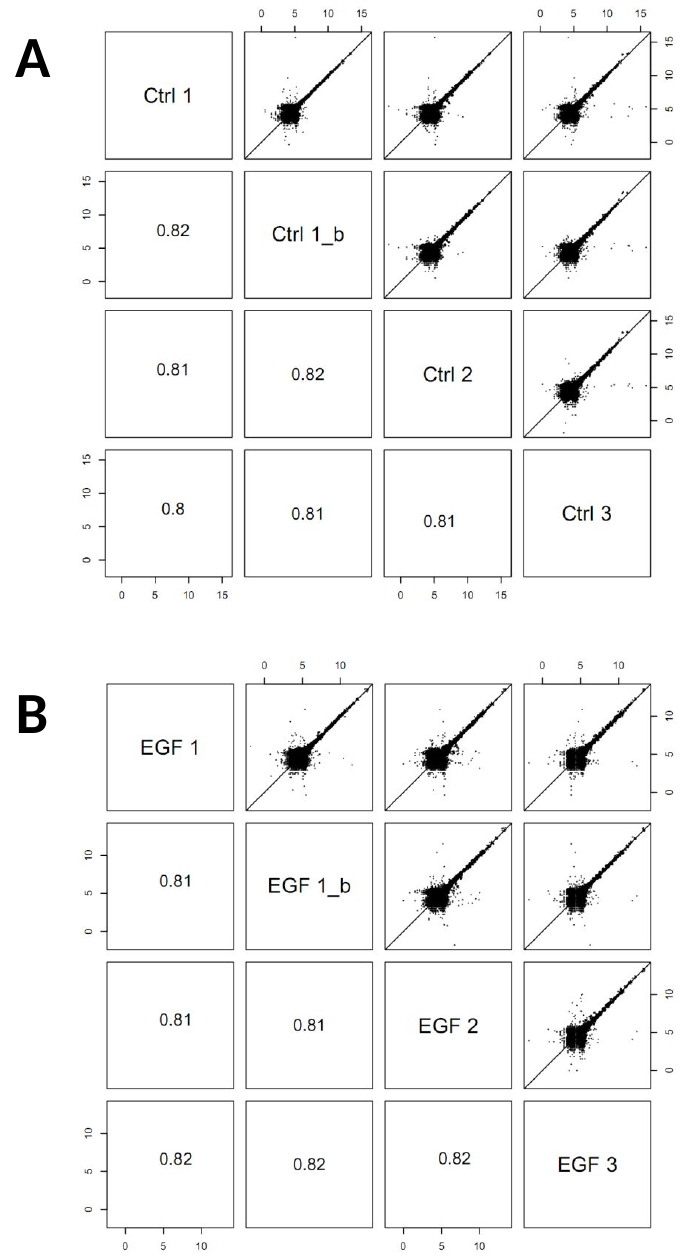

A
#
B

Supplement: Additional file 3 — Intensity correlation between replicate samples processed on Agilent microarrays. (A,B) Correlation of normalized log2intensities between biological replicates in the Control (A) and the EGF group (B) measured on Agilent microarrays. Sample 1 was processed with two technical replicates ('1' and '1_b'). [file 1471-2164-14-371-S3.pptx]

## Slide 1
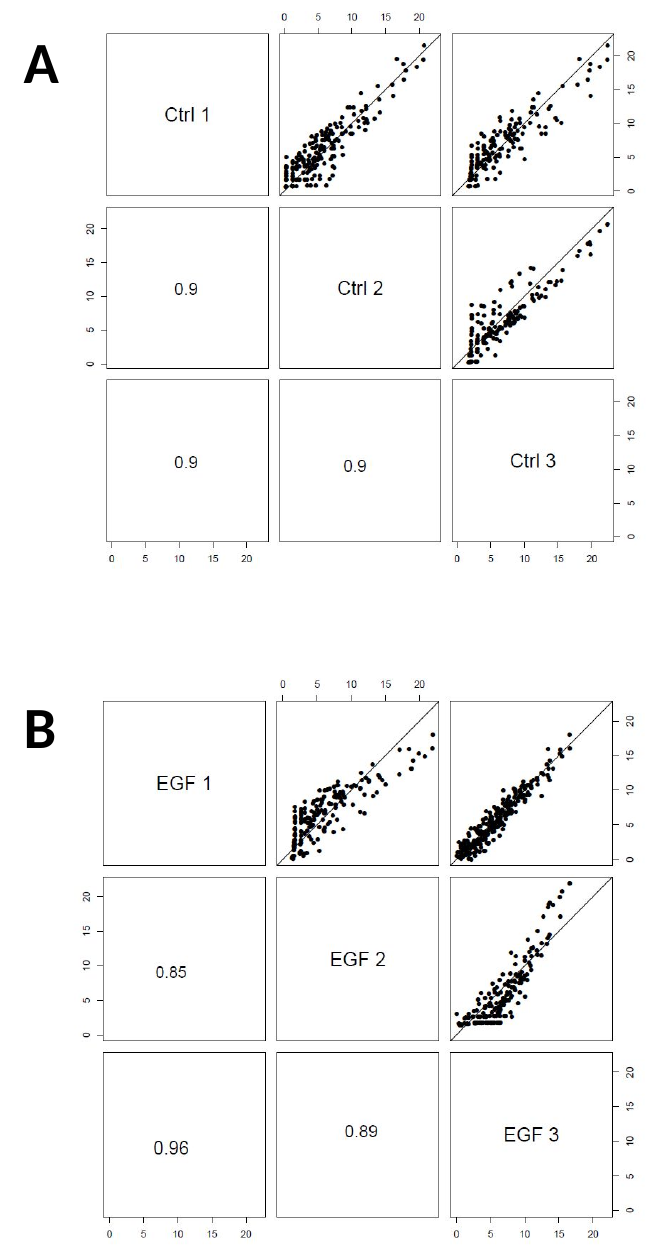

A
#
B

Supplement: Additional file 5 — Read count correlation between replicate samples processed by Illumina sequencing. (A,B) Correlation of normalized, log2transformed read counts per miRNA between biological replicates in the Control (A) and the EGF group (B) measured by Illumina small RNA-seq. [file 1471-2164-14-371-S5.pptx]

## Slide 1
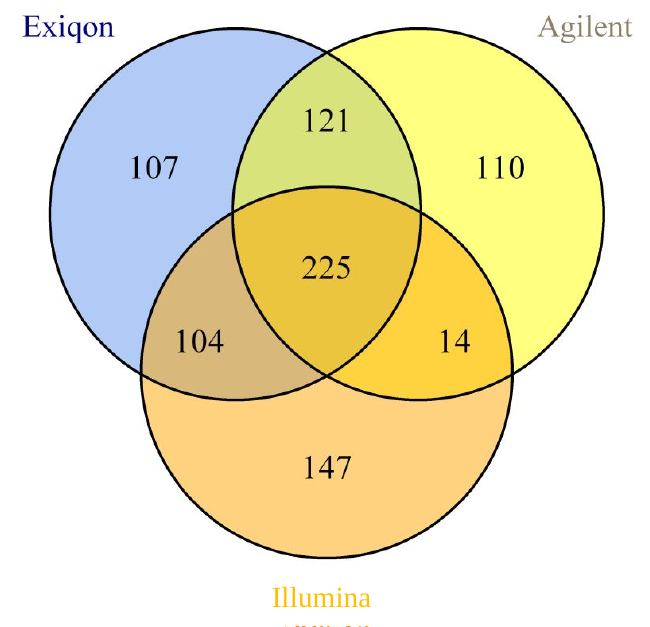

Illumina
#

Supplement: Additional file 6 — Numbers of miRNAs present in the different platforms. The Venn diagram shows how many miRNAs are present on the two microarray platforms, how many were detected by Illumina sequencing, and the numbers of the respective overlaps. [file 1471-2164-14-371-S6.pptx]

## Slide 1
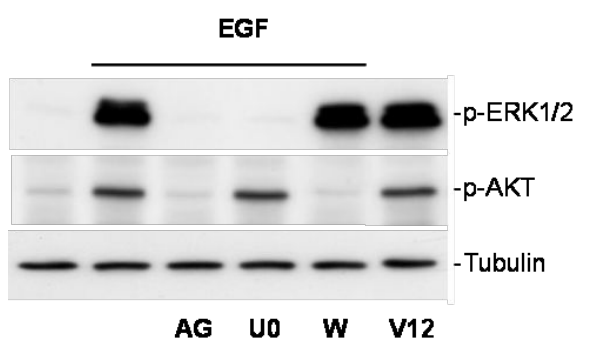

#

Supplement: Additional file 8 — Inhibition of the MAPK pathway in HeLa cells. HeLa cells were serum-starved for 24 hours and treated with EGF for 6 hours in the presence or absence of protein kinase inhibitors: AG1470 (EGFR inhibitor), U0126 (MEK inhibitor) and Wortmannin (PI3K inhibitor). In addition, HeLa cells were transfected with a constitutively active form of Ras (RasV12). Lysates were analysed by Western-Blot against pospho-ERK1/2 and phospho-AKT to ensure protein inhibitors and transfection action over MAPK and AKT pathways. Tubulin was used as sample loading control. [file 1471-2164-14-371-S8.pptx]
