# Supplementary material for: Microarray and deep sequencing cross-platform analysis of the mirRNome and isomiR variation in response to epidermal growth factor
Source: BMC Genomics. 2013 Jun 1;14:371. doi: 10.1186/1471-2164-14-371 (PMC3680220; doi:10.1186/1471-2164-14-371)
Supplement: Additional file 4 — Summary of read counts generated by the miRNA-seq MIRO analysis pipeline. [file 1471-2164-14-371-S4.pdf]

Supplementary Table 1

	Total reads	All matching reads	Unambiguously matching reads	No match reads
**CON1**	4512887	3844571	3708731	668316
**EGF1**	5295154	3491148	3369776	1804006
**CON2**	6566227	4689802	4445651	1876425
**EGF2**	5966700	4706846	4453746	1259854
**CON3**	6190275	4679417	4350224	1510858
**EGF3**	4429466	2127404	2012846	2302062
**average**	5493452	3923198	3723496	1570254

	Total unique sequences	All matching unique sequences	Unambiguously matching unique sequences	No match unique sequences
**CON1**	414305	19027	16970	395278
**EGF1**	927688	37376	34074	890312
**CON2**	776574	18096	16190	758478
**EGF2**	490200	15093	13266	475107
**CON3**	649745	18694	16396	631051
**EGF3**	1273784	41450	37906	1232334
**average**	755383	24956	22467	730427
