# Supplementary material for: Microarray and deep sequencing cross-platform analysis of the mirRNome and isomiR variation in response to epidermal growth factor
Source: BMC Genomics. 2013 Jun 1;14:371. doi: 10.1186/1471-2164-14-371 (PMC3680220; doi:10.1186/1471-2164-14-371)
Supplement: Additional file 7 — Agilent, Exiqon and Illumina EGF versus control log2ratios, fold changes, statistical significance nominal and adjusted p-values, and ranks computed with Rankprod. [file 1471-2164-14-371-S7.pdf]

ID	log2ratio_Agilent_1	log2ratio_Agilent_2	log2ratio_Agilent_3	avg_log2ratio_Agilent	log2ratio_Exiqon_1	log2ratio_Exiqon_2	log2ratio_Exiqon_3	avg_log2ratio_Exiqon	log2ratio_Illumina_1	log2ratio_Illumina_2	log2ratio_Illumina_3	avg_log2ratio_Illumina	avg_log2ratio_all	avg_FoldChange_Agilent	avg_FoldChange_Exiqon	avg_FoldChange_Illumina	avg_FoldChange_all	p.value.up	p.value.down	adjusted.p.value.up	adjusted.p.value.down	Index	Index_Rank	RPrank.1	RPrank.2	RPrank.3	RPrank.4	RPrank.5	RPrank.6	RPrank.7
hsa-miR-29b	0.64	0.61	0.61	0.62	0.51	0.52	0.43	0.49	-0.35	4.18	0.35	1.39	0.83	1.54	1.40	2.62	1.78	0.00	0.94	0.00	1.00	1	1_1	1	NA	NA	NA	NA	NA	NA
hsa-miR-29a	0.43	0.36	0.43	0.41	0.46	0.38	0.47	0.44	1.39	3.40	2.09	2.29	1.05	1.33	1.35	4.90	2.06	0.00	0.99	0.00	1.00	1	1_2	2	NA	NA	NA	NA	NA	NA
hsa-miR-222	0.60	0.74	0.63	0.66	0.40	0.40	0.40	0.40	0.40	2.09	0.18	0.89	0.65	1.58	1.32	1.85	1.57	0.00	0.95	0.00	1.00	1	1_3	3	NA	NA	NA	NA	NA	NA
hsa-miR-132	0.77	0.26	0.63	0.55	0.20	0.21	0.32	0.24	1.30			1.30	0.53	1.47	1.18	2.46	1.44	0.00	1.00	0.01	1.00	1	1_4	4	NA	NA	NA	NA	NA	NA
hsa-miR-221	0.34	0.31	0.33	0.33	0.37	0.43	0.37	0.39	2.49	3.12	0.33	1.98	0.90	1.26	1.31	3.94	1.86	0.00	0.98	0.01	1.00	1	1_5	5	NA	NA	NA	NA	NA	NA
hsa-miR-215	0.14	0.04	0.57	0.25	0.42	0.21	0.22	0.28	1.63			1.63	0.46	1.19	1.22	3.09	1.38	0.00	1.00	0.18	1.00	1	1_6	6	NA	NA	NA	NA	NA	NA
hsa-miR-21	0.14	0.01	0.26	0.14	0.46	0.21	0.30	0.32	0.28	3.22	-0.45	1.02	0.49	1.10	1.25	2.02	1.41	0.00	0.90	0.35	1.00	1	1_7	7	NA	NA	NA	NA	NA	NA
hsa-miR-7	0.03	0.08	0.12	0.08	0.33	0.05	0.12	0.17	5.15	3.21	3.84	4.07	1.44	1.05	1.12	16.75	2.71	0.00	0.98	0.43	1.00	1	1_8	8	NA	NA	NA	NA	NA	NA
hsa-miR-31	0.34	0.52	0.17	0.35	0.26	0.17	0.12	0.18	-3.28	1.20	-1.04	-1.04	-0.17	1.27	1.14	-2.06	-1.13	0.00	0.73	1.00	1.00	1	1_9	9	NA	NA	NA	NA	NA	NA
hsa-miR-675	0.38	0.00	-0.01	0.12	0.24	0.13	0.27	0.21					0.17	1.09	1.16	NA	1.12	0.01	1.00	1.00	1.00	1	1_10	10	NA	NA	NA	NA	NA	NA
hsa-miR-183	0.02	0.12	0.19	0.11	0.24	0.15	0.19	0.19	2.56	1.49	3.36	2.47	0.92	1.08	1.14	5.55	1.90	0.01	0.97	1.00	1.00	1	1_11	11	NA	NA	NA	NA	NA	NA
hsa-miR-203	-0.04	0.11	0.15	0.07	0.09	0.12	0.11	0.10	3.26	2.25	5.09	3.54	1.24	1.05	1.07	11.62	2.36	0.01	0.96	1.00	1.00	1	1_12	12	NA	NA	NA	NA	NA	NA
hsa-miR-660	-0.07	-0.06	0.12	0.00	0.08	0.12	0.17	0.13	5.04	1.44	4.46	3.65	1.26	-1.00	1.09	12.53	2.39	0.01	0.91	1.00	1.00	1	1_13	13	NA	NA	NA	NA	NA	NA
hsa-miR-194	0.06	0.09	0.01	0.05	0.28	0.22	0.16	0.22					0.14	1.04	1.16	NA	1.10	0.01	1.00	1.00	1.00	1	1_14	14	NA	NA	NA	NA	NA	NA
hsa-miR-9	-0.06	0.00	-0.02	-0.02	0.08	0.17	0.13	0.13	3.11	-0.14	5.13	2.70	0.93	-1.02	1.09	6.51	1.91	0.02	0.78	1.00	1.00	1	1_15	15	NA	NA	NA	NA	NA	NA
hsa-miR-192	0.13	0.09	0.25	0.16	0.18	0.09	0.19	0.15	1.82	1.08	1.41	1.44	0.58	1.12	1.11	2.70	1.50	0.03	0.93	1.00	1.00	1	1_16	16	NA	NA	NA	NA	NA	NA
hsa-miR-622	0.24	0.27	0.23	0.25	0.22	-0.06	0.08	0.08	-0.40			-0.40	0.09	1.19	1.06	-1.32	1.06	0.04	0.95	1.00	1.00	1	1_17	17	NA	NA	NA	NA	NA	NA
hsa-miR-421	0.09	-0.07	0.14	0.05	0.21	0.02	0.02	0.08	3.87	-0.45	4.43	2.62	0.92	1.04	1.06	6.13	1.89	0.05	0.79	1.00	1.00	1	1_18	18	NA	NA	NA	NA	NA	NA
hsa-miR-632	0.31	-0.13	0.73	0.30	0.11	-0.03	-0.12	-0.01					0.15	1.23	-1.01	NA	1.11	0.06	0.76	1.00	1.00	1	1_19	19	NA	NA	NA	NA	NA	NA
hsa-miR-29c	0.00	0.00	0.03	0.01	0.22	0.09	0.22	0.18	-0.25	2.38	1.08	1.07	0.42	1.01	1.13	2.10	1.34	0.06	0.82	1.00	1.00	1	1_20	20	NA	NA	NA	NA	NA	NA
hsa-miR-130b	0.08	-0.02	0.18	0.08	0.29	0.02	0.10	0.14	2.82	-0.33	2.10	1.53	0.58	1.05	1.10	2.89	1.50	0.06	0.83	1.00	1.00	1	1_21	21	NA	NA	NA	NA	NA	NA
hsa-miR-182	-0.02	-0.11	0.04	-0.03	0.11	0.06	0.09	0.09	3.37	2.33	2.86	2.85	0.97	-1.02	1.06	7.23	1.96	0.07	0.88	1.00	1.00	1	1_22	22	NA	NA	NA	NA	NA	NA
hsa-miR-608	0.42	0.19	0.20	0.27	0.06	0.04	0.00	0.03					0.15	1.21	1.02	NA	1.11	0.08	1.00	1.00	1.00	1	1_23	23	NA	NA	NA	NA	NA	NA
hsa-miR-769-3p	0.00	0.10	0.41	0.17	0.17	0.06	0.05	0.09					0.13	1.13	1.07	NA	1.10	0.09	1.00	1.00	1.00	1	1_24	24	NA	NA	NA	NA	NA	NA
hsa-miR-96	0.07	-0.29	0.11	-0.04	0.15	0.07	0.07	0.10	4.69			4.69	0.70	-1.03	1.07	25.81	1.62	0.10	0.91	1.00	1.00	1	1_25	25	NA	NA	NA	NA	NA	NA
hsa-miR-95	0.01	-0.40	-0.10	-0.17	0.34	0.05	0.07	0.16	2.61		2.91	2.76	0.69	-1.12	1.11	6.78	1.61	0.12	0.55	1.00	1.00	1	1_26	26	NA	NA	NA	NA	NA	NA
hsa-miR-135b	-0.11	-0.10	0.09	-0.04	0.10	0.29	0.17	0.18					0.07	-1.03	1.14	NA	1.05	0.12	0.95	1.00	1.00	1	1_27	27	NA	NA	NA	NA	NA	NA
hsa-miR-553	-0.43	0.20	0.08	-0.05	0.25	0.11	0.05	0.14					0.04	-1.03	1.10	NA	1.03	0.12	0.64	1.00	1.00	1	1_28	28	NA	NA	NA	NA	NA	NA
hsa-miR-1	0.19	0.17	0.09	0.15	0.23	-0.05	-0.09	0.03	2.67	0.85	2.21	1.91	0.70	1.11	1.02	3.76	1.62	0.12	0.69	1.00	1.00	1	1_29	29	NA	NA	NA	NA	NA	NA
hsa-miR-651	-0.09	0.06	0.31	0.09	0.21	0.01	-0.01	0.07	3.15			3.15	0.52	1.06	1.05	8.88	1.43	0.14	0.95	1.00	1.00	1	1_30	30	NA	NA	NA	NA	NA	NA
hsa-miR-27a	-0.02	0.14	-0.07	0.02	0.20	0.04	0.11	0.12	0.44	1.74	1.54	1.24	0.46	1.01	1.09	2.36	1.37	0.14	0.79	1.00	1.00	1	1_31	31	NA	NA	NA	NA	NA	NA
hsa-miR-448	-0.12	0.31	0.13	0.11	0.15	0.04	0.06	0.09					0.10	1.08	1.06	NA	1.07	0.15	0.99	1.00	1.00	1	1_32	32	NA	NA	NA	NA	NA	NA
hsa-miR-542-3p	0.15	-0.15	0.26	0.09	-0.03	0.09	0.07	0.04	2.10			2.10	0.36	1.06	1.03	4.29	1.28	0.16	0.92	1.00	1.00	1	1_33	33	NA	NA	NA	NA	NA	NA
hsa-miR-181a	-0.02	0.11	-0.05	0.01	0.25	0.06	0.02	0.11	2.89	1.06	0.55	1.50	0.54	1.01	1.08	2.83	1.45	0.17	0.76	1.00	1.00	1	1_34	34	NA	NA	NA	NA	NA	NA
hsa-miR-596	0.10	0.30	-0.06	0.11	-0.04	0.04	0.11	0.04					0.07	1.08	1.03	NA	1.05	0.18	0.96	1.00	1.00	1	1_35	35	NA	NA	NA	NA	NA	NA
hsa-miR-375	-0.06	0.06	0.57	0.19	0.24	-0.02	0.03	0.08	-0.86	-0.50		-0.68	-0.07	1.14	1.06	-1.60	-1.05	0.19	0.58	1.00	1.00	1	1_36	36	NA	NA	NA	NA	NA	NA

**ID**	log2ratio_Agilent_1	log2ratio_Agilent_2	log2ratio_Agilent_3	avg_log2ratio_Agilent	log2ratio_Exiqon_1	log2ratio_Exiqon_2	log2ratio_Exiqon_3	avg_log2ratio_Exiqon	log2ratio_Illumina_1	log2ratio_Illumina_2	log2ratio_Illumina_3	avg_log2ratio_Illumina	avg_log2ratio_all	avg_FoldChange_Agilent	avg_FoldChange_Exiqon	avg_FoldChange_Illumina	**avg_FoldChange_all**	p.value.up	p.value.down	**adjusted.p.value.up**	**adjusted.p.value.down**	Index	Index_Rank	RPrank.1	RPrank.2	RPrank.3	RPrank.4	RPrank.5	RPrank.6	RPrank.7
**hsa-miR-100**	-0.02	0.06	0.27	0.10	-0.02	-0.06	0.14	0.02	1.08	1.44	1.46	1.33	0.48	1.07	1.01	2.51	**1.40**	0.19	0.70	**1.00**	**1.00**	1	1_37	37	NA	NA	NA	NA	NA	NA
**hsa-miR-378**	0.11	0.14	0.08	0.11	0.15	-0.03	0.05	0.06	-0.92	2.14	0.48	0.57	0.24	1.08	1.04	1.48	**1.19**	0.21	0.71	**1.00**	**1.00**	1	1_38	38	NA	NA	NA	NA	NA	NA
**hsa-miR-505**	-0.05	0.20	0.21	0.12	0.16	0.03	0.01	0.07					0.09	1.09	1.05	NA	**1.07**	0.22	0.99	**1.00**	**1.00**	1	1_39	39	NA	NA	NA	NA	NA	NA
**hsa-miR-196b**	-0.16	0.03	0.00	-0.04	0.09	0.01	0.03	0.04	3.09	-0.56	4.15	2.22	0.74	-1.03	1.03	4.67	**1.67**	0.24	0.49	**1.00**	**1.00**	1	1_40	40	NA	NA	NA	NA	NA	NA
**hsa-miR-503**	-0.12	0.08	0.35	0.10	-0.36	-0.21	-0.35	-0.31	3.32	1.14	3.38	2.61	0.80	1.07	-1.24	6.12	**1.74**	0.24	0.07	**1.00**	**1.00**	1	1_41	41	NA	NA	NA	NA	NA	NA
**hsa-miR-573**	0.25	-0.11	0.50	0.21	0.00	-0.03	0.06	0.01	-0.53			-0.53	0.02	1.16	1.00	-1.45	**1.01**	0.24	0.76	**1.00**	**1.00**	1	1_42	42	NA	NA	NA	NA	NA	NA
**hsa-miR-22**	0.07	0.06	-0.09	0.01	0.17	-0.03	0.07	0.07	1.18	0.66	2.85	1.56	0.55	1.01	1.05	2.96	**1.46**	0.26	0.69	**1.00**	**1.00**	1	1_43	43	NA	NA	NA	NA	NA	NA
**hsa-miR-518c**	-0.07	0.60	0.03	0.19	-0.04	0.00	0.06	0.01					0.10	1.14	1.00	NA	**1.07**	0.26	0.92	**1.00**	**1.00**	1	1_44	44	NA	NA	NA	NA	NA	NA
**hsa-miR-512-3p**	-0.07	0.16	0.26	0.12	0.21	-0.06	0.04	0.06					0.09	1.08	1.04	NA	**1.06**	0.26	0.97	**1.00**	**1.00**	1	1_45	45	NA	NA	NA	NA	NA	NA
**hsa-miR-382**	-0.15	0.17	-0.14	-0.04	0.16	0.06	0.13	0.12					0.04	-1.03	1.09	NA	**1.03**	0.26	0.84	**1.00**	**1.00**	1	1_46	46	NA	NA	NA	NA	NA	NA
**hsa-miR-662**	0.08	-0.37	0.49	0.07	0.09	0.04	0.01	0.05					0.06	1.05	1.03	NA	**1.04**	0.28	0.82	**1.00**	**1.00**	1	1_47	47	NA	NA	NA	NA	NA	NA
**hsa-miR-500**	-0.07	-0.05	0.14	0.01	0.22	0.12	0.00	0.11					0.06	1.01	1.08	NA	**1.04**	0.28	0.98	**1.00**	**1.00**	1	1_48	48	NA	NA	NA	NA	NA	NA
**hsa-miR-18a**	0.04	0.03	0.04	0.04	0.18	0.05	0.06	0.10	0.75			0.75	0.17	1.03	1.07	1.68	**1.12**	0.30	0.97	**1.00**	**1.00**	1	1_49	49	NA	NA	NA	NA	NA	NA
**hsa-miR-422a**	-0.06	-0.37	-0.38	-0.27	0.29	0.02	0.05	0.12	0.16	2.46	0.26	0.96	0.27	-1.21	1.09	1.95	**1.21**	0.31	0.17	**1.00**	**1.00**	1	1_50	50	NA	NA	NA	NA	NA	NA
**hsa-miR-570**	0.12	-0.29	0.24	0.03	0.24	-0.06	0.04	0.07					0.05	1.02	1.05	NA	**1.03**	0.31	0.84	**1.00**	**1.00**	1	1_51	51	NA	NA	NA	NA	NA	NA
**hsa-miR-652**	0.03	0.11	0.08	0.07	0.03	-0.03	0.11	0.03		-1.61	2.44	0.41	0.14	1.05	1.02	1.33	**1.10**	0.31	0.65	**1.00**	**1.00**	1	1_52	52	NA	NA	NA	NA	NA	NA
**hsa-miR-190**	0.03	-0.02	0.20	0.07	0.14	0.09	0.01	0.08					0.07	1.05	1.06	NA	**1.05**	0.32	0.99	**1.00**	**1.00**	1	1_53	53	NA	NA	NA	NA	NA	NA
**hsa-miR-638**	0.28	0.54	-0.38	0.15	-0.11	-0.11	-0.17	-0.13		1.18		1.18	0.18	1.11	-1.09	2.27	**1.13**	0.32	0.08	**1.00**	**1.00**	1	1_54	54	NA	NA	NA	NA	NA	NA
**hsa-miR-186**	-0.15	-0.07	-0.08	-0.10	0.09	0.00	0.09	0.06	2.81	-0.17	3.72	2.12	0.69	-1.07	1.04	4.35	**1.62**	0.32	0.43	**1.00**	**1.00**	1	1_55	55	NA	NA	NA	NA	NA	NA
**hsa-miR-657**	-0.10	0.00	0.27	0.06	0.13	0.02	0.08	0.08					0.07	1.04	1.05	NA	**1.05**	0.32	0.98	**1.00**	**1.00**	1	1_56	56	NA	NA	NA	NA	NA	NA
**hsa-miR-650**	-0.05	0.18	0.23	0.12	0.15	0.01	0.01	0.06					0.09	1.09	1.04	NA	**1.06**	0.33	0.99	**1.00**	**1.00**	1	1_57	57	NA	NA	NA	NA	NA	NA
**hsa-miR-30d**	0.00	0.18	-0.20	-0.01	0.05	0.03	0.07	0.05	0.72	1.87	-0.36	0.74	0.26	-1.01	1.03	1.67	**1.20**	0.34	0.50	**1.00**	**1.00**	1	1_58	58	NA	NA	NA	NA	NA	NA
**hsa-miR-571**	-0.12	0.18	0.26	0.11	0.16	0.02	-0.02	0.05					0.08	1.08	1.04	NA	**1.06**	0.34	0.94	**1.00**	**1.00**	1	1_59	59	NA	NA	NA	NA	NA	NA
**hsa-miR-101**	-0.11	-0.03	0.09	-0.02	0.13	0.12	-0.09	0.05	1.32	1.00	2.18	1.50	0.51	-1.01	1.04	2.82	**1.43**	0.35	0.55	**1.00**	**1.00**	1	1_60	60	NA	NA	NA	NA	NA	NA
**hsa-miR-206**	0.18	0.03	0.02	0.08	0.16	0.00	0.05	0.07					0.07	1.05	1.05	NA	**1.05**	0.36	0.99	**1.00**	**1.00**	1	1_61	61	NA	NA	NA	NA	NA	NA
**hsa-miR-335**	-0.10	-0.08	-0.03	-0.07	-0.03	0.07	0.01	0.01	5.36			5.36	0.74	-1.05	1.01	41.03	**1.67**	0.36	0.70	**1.00**	**1.00**	1	1_62	62	NA	NA	NA	NA	NA	NA
**hsa-miR-551b**	0.11	-0.04	0.15	0.07	0.15	0.08	-0.04	0.07					0.07	1.05	1.05	NA	**1.05**	0.36	0.98	**1.00**	**1.00**	1	1_63	63	NA	NA	NA	NA	NA	NA
**hsa-miR-580**	0.11	0.21	0.16	0.16	-0.15	-0.04	0.04	-0.05					0.05	1.12	-1.04	NA	**1.04**	0.37	0.87	**1.00**	**1.00**	1	1_64	64	NA	NA	NA	NA	NA	NA
**hsa-miR-598**	0.08	0.05	-0.01	0.04	0.12	-0.03	0.13	0.07					0.05	1.03	1.05	NA	**1.04**	0.37	0.97	**1.00**	**1.00**	1	1_65	65	NA	NA	NA	NA	NA	NA
**hsa-miR-497**	0.03	0.13	-0.12	0.01	0.18	0.05	0.04	0.09					0.05	1.01	1.06	NA	**1.04**	0.37	0.96	**1.00**	**1.00**	1	1_66	66	NA	NA	NA	NA	NA	NA
**hsa-miR-149**	0.17	-0.03	0.21	0.12	0.07	-0.06	0.07	0.03					0.07	1.08	1.02	NA	**1.05**	0.38	0.97	**1.00**	**1.00**	1	1_67	67	NA	NA	NA	NA	NA	NA
**hsa-miR-424**	-0.08	-0.12	0.18	-0.01	0.14	0.05	0.05	0.08	2.49			2.49	0.39	-1.01	1.06	5.61	**1.31**	0.38	0.92	**1.00**	**1.00**	1	1_68	68	NA	NA	NA	NA	NA	NA
**hsa-miR-99b**	-0.03	0.21	-0.05	0.04	0.06	0.01	0.09	0.05	0.04	-2.93	1.42	-0.49	-0.13	1.03	1.04	-1.40	**-1.10**	0.38	0.33	**1.00**	**1.00**	1	1_69	69	NA	NA	NA	NA	NA	NA
**hsa-miR-370**	0.04	0.09	-0.06	0.02	0.12	0.00	0.11	0.08					0.05	1.02	1.06	NA	**1.04**	0.41	0.97	**1.00**	**1.00**	1	1_70	70	NA	NA	NA	NA	NA	NA
**hsa-miR-554**	-0.19	0.00	0.11	-0.03	-0.38	0.15	0.10	-0.04					-0.03	-1.02	-1.03	NA	**-1.02**	0.41	0.47	**1.00**	**1.00**	1	1_71	71	NA	NA	NA	NA	NA	NA
**hsa-miR-193b**	-0.01	-0.10	-0.02	-0.04	0.06	0.07	0.00	0.04	2.01	-5.40	3.06	-0.11	-0.04	-1.03	1.03	-1.08	**-1.03**	0.41	0.34	**1.00**	**1.00**	1	1_72	72	NA	NA	NA	NA	NA	NA

ID	log2ratio_Agilent_1	log2ratio_Agilent_2	log2ratio_Agilent_3	avg_log2ratio_Agilent	log2ratio_Exiqon_1	log2ratio_Exiqon_2	log2ratio_Exiqon_3	avg_log2ratio_Exiqon	log2ratio_Illumina_1	log2ratio_Illumina_2	log2ratio_Illumina_3	avg_log2ratio_Illumina	avg_log2ratio_all	avg_FoldChange_Agilent	avg_FoldChange_Exiqon	avg_FoldChange_Illumina	avg_FoldChange_all	p.value.up	p.value.down	adjusted.p.value.up	adjusted.p.value.down	Index	Index_Rank	RPrank.1	RPrank.2	RPrank.3	RPrank.4	RPrank.5	RPrank.6	RPrank.7
hsa-miR-636	-0.08	0.24	0.00	0.06	0.03	-0.06	-0.04	-0.02		2.64		2.64	0.39	1.04	-1.02	6.22	1.31	0.41	0.70	1.00	1.00	1	1_73	73	NA	NA	NA	NA	NA	NA
hsa-let-7i	0.12	0.02	-0.01	0.04	0.00	-0.05	0.08	0.01	-3.55	2.28	-4.63	-1.97	-0.64	1.03	1.01	-3.92	-1.56	0.42	0.21	1.00	1.00	1	1_74	74	NA	NA	NA	NA	NA	NA
hsa-miR-33b	0.15	-0.18	0.36	0.11	-0.10	0.02	-0.02	-0.03	-0.22	0.44	0.88	0.37	0.15	1.08	-1.02	1.29	1.11	0.43	0.33	1.00	1.00	1	1_75	75	NA	NA	NA	NA	NA	NA
hsa-miR-433	0.01	0.19	0.35	0.18	-0.10	-0.03	-0.01	-0.05					0.07	1.13	-1.03	NA	1.05	0.43	0.88	1.00	1.00	1	1_76	76	NA	NA	NA	NA	NA	NA
hsa-miR-340	0.15	-0.35	0.01	-0.07	0.15	0.00	-0.01	0.05	1.27	1.68	-0.08	0.95	0.31	-1.05	1.03	1.94	1.24	0.43	0.41	1.00	1.00	1	1_77	77	NA	NA	NA	NA	NA	NA
hsa-miR-299-3p	-0.01	0.15	0.15	0.09	-0.05	0.03	0.07	0.02					0.06	1.07	1.01	NA	1.04	0.43	0.97	1.00	1.00	1	1_78	78	NA	NA	NA	NA	NA	NA
hsa-miR-187	-0.27	-0.03	0.18	-0.04	0.09	0.13	0.03	0.08					0.02	-1.03	1.06	NA	1.01	0.44	0.71	1.00	1.00	1	1_79	79	NA	NA	NA	NA	NA	NA
hsa-miR-489	0.34	0.05	0.12	0.17	0.01	-0.06	-0.04	-0.03					0.07	1.13	-1.02	NA	1.05	0.44	0.88	1.00	1.00	1	1_80	80	NA	NA	NA	NA	NA	NA
hsa-miR-200a	0.19	-0.20	0.21	0.07	-0.13	0.10	-0.01	-0.01	-0.19			-0.19	0.00	1.05	-1.01	-1.14	-1.00	0.44	0.54	1.00	1.00	1	1_81	81	NA	NA	NA	NA	NA	NA
hsa-miR-518e	-0.15	0.10	0.23	0.06	0.05	0.12	-0.05	0.04					0.05	1.04	1.03	NA	1.03	0.44	0.82	1.00	1.00	1	1_82	82	NA	NA	NA	NA	NA	NA
hsa-miR-625	-0.01	-0.07	0.05	-0.01	0.21	0.09	0.01	0.10	-0.75			-0.75	-0.07	-1.01	1.07	-1.68	-1.05	0.44	0.84	1.00	1.00	1	1_83	83	NA	NA	NA	NA	NA	NA
hsa-miR-99a	-0.30	-0.17	-0.07	-0.18	-0.05	0.14	0.07	0.05	1.28	1.21	1.83	1.44	0.44	-1.13	1.04	2.71	1.36	0.45	0.19	1.00	1.00	1	1_84	84	NA	NA	NA	NA	NA	NA
hsa-miR-20a	-0.08	-0.13	0.14	-0.02	0.01	0.00	-0.02	0.00	2.33	1.42	2.28	2.01	0.66	-1.02	-1.00	4.02	1.58	0.45	0.61	1.00	1.00	1	1_85	85	NA	NA	NA	NA	NA	NA
hsa-miR-196a	-0.10	-0.15	0.04	-0.07	0.12	-0.09	0.00	0.01	2.46	0.05	3.73	2.08	0.67	-1.05	1.01	4.24	1.60	0.46	0.36	1.00	1.00	1	1_86	86	NA	NA	NA	NA	NA	NA
hsa-miR-581	0.17	0.17	0.26	0.20	-0.03	-0.11	-0.07	-0.07					0.06	1.15	-1.05	NA	1.05	0.47	0.72	1.00	1.00	1	1_87	87	NA	NA	NA	NA	NA	NA
hsa-miR-345	0.05	-0.29	0.04	-0.07	0.16	-0.05	0.00	0.04	1.31	-2.81	2.96	0.48	0.15	-1.05	1.03	1.40	1.11	0.47	0.24	1.00	1.00	1	1_88	88	NA	NA	NA	NA	NA	NA
hsa-miR-558	-0.06	-0.28	0.42	0.02	-0.32	0.01	0.11	-0.07	-0.28			-0.28	-0.06	1.02	-1.05	-1.21	-1.04	0.48	0.29	1.00	1.00	1	1_89	89	NA	NA	NA	NA	NA	NA
hsa-miR-452	-0.09	-0.04	0.06	-0.02	-0.10	-0.06	-0.06	-0.07	3.63	0.83	2.59	2.35	0.75	-1.02	-1.05	5.10	1.68	0.49	0.33	1.00	1.00	1	1_90	90	NA	NA	NA	NA	NA	NA
hsa-miR-30c	-0.12	0.02	0.05	-0.01	0.05	-0.10	-0.15	-0.07	2.34	1.59	2.67	2.20	0.71	-1.01	-1.05	4.60	1.63	0.49	0.33	1.00	1.00	1	1_91	91	NA	NA	NA	NA	NA	NA
hsa-miR-629	-0.04	0.00	-0.14	-0.06	0.13	0.06	-0.01	0.06	1.40	0.88	1.47	1.25	0.42	-1.04	1.04	2.37	1.33	0.49	0.53	1.00	1.00	1	1_92	92	NA	NA	NA	NA	NA	NA
hsa-miR-487b	0.18	-0.16	0.02	0.01	0.03	0.01	0.10	0.05					0.03	1.01	1.03	NA	1.02	0.50	0.90	1.00	1.00	1	1_93	93	NA	NA	NA	NA	NA	NA
hsa-miR-24	0.03	-0.01	0.05	0.02	0.07	0.02	-0.01	0.03	0.20	1.21	1.33	0.91	0.32	1.02	1.02	1.88	1.25	0.51	0.67	1.00	1.00	1	1_94	94	NA	NA	NA	NA	NA	NA
hsa-miR-326	0.06	0.19	0.08	0.11	-0.29	0.08	-0.10	-0.11					0.00	1.08	-1.08	NA	1.00	0.51	0.63	1.00	1.00	1	1_95	95	NA	NA	NA	NA	NA	NA
hsa-miR-27b	-0.18	-0.15	-0.01	-0.11	0.10	0.04	0.06	0.07	1.18	1.21	1.58	1.33	0.43	-1.08	1.05	2.51	1.34	0.52	0.44	1.00	1.00	1	1_96	96	NA	NA	NA	NA	NA	NA
hsa-miR-143	-0.13	-0.04	0.52	0.11	-0.08	-0.04	0.08	-0.01	-0.92			-0.92	-0.09	1.08	-1.01	-1.90	-1.06	0.52	0.40	1.00	1.00	1	1_97	97	NA	NA	NA	NA	NA	NA
hsa-miR-25	-0.13	-0.04	-0.03	-0.07	-0.10	0.05	0.06	0.00	0.96	2.36	-1.15	0.73	0.22	-1.05	1.00	1.65	1.16	0.52	0.32	1.00	1.00	1	1_98	98	NA	NA	NA	NA	NA	NA
hsa-miR-152	-0.19	-0.01	0.01	-0.06	0.05	0.01	-0.04	0.01	1.94	1.70	2.05	1.90	0.61	-1.05	1.01	3.73	1.53	0.53	0.46	1.00	1.00	1	1_99	99	NA	NA	NA	NA	NA	NA
hsa-miR-379	0.06	0.22	0.00	0.10	0.05	-0.08	0.02	0.00					0.05	1.07	-1.00	NA	1.03	0.53	0.91	1.00	1.00	1	1_100	100	NA	NA	NA	NA	NA	NA
hsa-miR-592	-0.13	-0.26	0.10	-0.10	0.27	0.02	0.04	0.11					0.01	-1.07	1.08	NA	1.01	0.54	0.73	1.00	1.00	1	1_101	101	NA	NA	NA	NA	NA	NA
hsa-miR-181d	0.00	-0.01	0.02	0.00	0.12	0.01	-0.04	0.03	2.00	0.92	0.43	1.11	0.38	1.00	1.02	2.17	1.30	0.55	0.58	1.00	1.00	1	1_102	102	NA	NA	NA	NA	NA	NA
hsa-miR-105	0.04	-0.57	-0.01	-0.18	-0.04	0.04	0.04	0.01	2.32		1.86	2.09	0.46	-1.13	1.01	4.26	1.37	0.55	0.20	1.00	1.00	1	1_103	103	NA	NA	NA	NA	NA	NA
hsa-miR-372	0.06	0.28	0.08	0.14	-0.08	-0.01	-0.07	-0.06					0.04	1.10	-1.04	NA	1.03	0.56	0.78	1.00	1.00	1	1_104	104	NA	NA	NA	NA	NA	NA
hsa-miR-23a	0.04	-0.08	-0.04	-0.03	0.06	-0.11	0.01	-0.01	2.07	1.58	0.47	1.37	0.44	-1.02	-1.01	2.59	1.36	0.56	0.43	1.00	1.00	1	1_105	105	NA	NA	NA	NA	NA	NA
hsa-miR-610	-0.24	0.25	0.13	0.05	0.16	-0.09	-0.03	0.01					0.03	1.03	1.01	NA	1.02	0.56	0.47	1.00	1.00	1	1_106	106	NA	NA	NA	NA	NA	NA
hsa-miR-15a	-0.13	-0.14	-0.09	-0.12	0.06	-0.08	-0.08	-0.04	5.36			5.36	0.70	-1.09	-1.02	41.05	1.62	0.57	0.23	1.00	1.00	1	1_107	107	NA	NA	NA	NA	NA	NA
hsa-miR-200c	-0.23	0.42	0.10	0.10	-0.03	0.00	-0.04	-0.02					0.04	1.07	-1.01	NA	1.03	0.57	0.54	1.00	1.00	1	1_108	108	NA	NA	NA	NA	NA	NA

ID	log2ratio_Agilent_1	log2ratio_Agilent_2	log2ratio_Agilent_3	avg_log2ratio_Agilent	log2ratio_Exiqon_1	log2ratio_Exiqon_2	log2ratio_Exiqon_3	avg_log2ratio_Exiqon	log2ratio_Illumina_1	log2ratio_Illumina_2	log2ratio_Illumina_3	avg_log2ratio_Illumina	avg_log2ratio_all	avg_FoldChange_Agilent	avg_FoldChange_Exiqon	avg_FoldChange_Illumina	**avg_FoldChange_all**	p.value.up	p.value.down	**adjusted.p.value.up**	**adjusted.p.value.down**	Index	Index_Rank	RPrank.1	RPrank.2	RPrank.3	RPrank.4	RPrank.5	RPrank.6	RPrank.7
**hsa-miR-768-5p**	0.05	-0.05	-0.12	-0.04	0.14	0.10	-0.02	0.07	0.70			0.70	0.11	-1.03	1.05	1.63	**1.08**	0.57	0.77	**1.00**	**1.00**	1	1_109	109	NA	NA	NA	NA	NA	NA
**hsa-miR-103**	-0.03	-0.02	-0.05	-0.03	0.02	-0.11	0.05	-0.01	1.31	2.26	-1.24	0.77	0.24	-1.02	-1.01	1.71	**1.18**	0.57	0.33	**1.00**	**1.00**	1	1_110	110	NA	NA	NA	NA	NA	NA
**hsa-miR-148a**	-0.14	-0.25	-0.11	-0.17	0.31	-0.02	-0.04	0.08	1.83		1.84	1.84	0.43	-1.12	1.06	3.57	**1.34**	0.58	0.25	**1.00**	**1.00**	1	1_111	111	NA	NA	NA	NA	NA	NA
**hsa-miR-376b**	0.04	-0.30	0.36	0.03	0.05	-0.01	0.01	0.01					0.02	1.02	1.01	NA	**1.02**	0.58	0.75	**1.00**	**1.00**	1	1_112	112	NA	NA	NA	NA	NA	NA
**hsa-miR-23b**	-0.07	-0.25	-0.09	-0.14	-0.02	-0.13	-0.10	-0.08	2.94	2.66	0.34	1.98	0.59	-1.10	-1.06	3.94	**1.50**	0.61	0.10	**1.00**	**1.00**	1	1_113	113	NA	NA	NA	NA	NA	NA
**hsa-miR-148b**	-0.01	-0.11	0.02	-0.03	0.07	-0.04	-0.03	0.00	1.24	1.12	2.51	1.63	0.53	-1.02	-1.00	3.09	**1.45**	0.61	0.52	**1.00**	**1.00**	1	1_114	114	NA	NA	NA	NA	NA	NA
**hsa-miR-93**	-0.08	0.06	-0.05	-0.03	-0.03	-0.08	0.01	-0.03	2.19	1.36	1.24	1.59	0.51	-1.02	-1.02	3.02	**1.43**	0.61	0.44	**1.00**	**1.00**	1	1_115	115	NA	NA	NA	NA	NA	NA
**hsa-miR-138**	-0.11	0.00	0.15	0.01	0.07	0.02	-0.02	0.02	-0.72	-1.70	1.58	-0.28	-0.08	1.01	1.02	-1.22	**-1.06**	0.62	0.31	**1.00**	**1.00**	1	1_116	116	NA	NA	NA	NA	NA	NA
**hsa-miR-198**	0.02	0.00	0.13	0.05	0.08	-0.01	0.04	0.04					0.04	1.04	1.03	NA	**1.03**	0.62	0.98	**1.00**	**1.00**	1	1_117	117	NA	NA	NA	NA	NA	NA
**hsa-miR-10b**	0.27	0.04	-0.43	-0.04	0.13	-0.11	-0.01	0.00					-0.02	-1.03	1.00	NA	**-1.01**	0.62	0.44	**1.00**	**1.00**	1	1_118	118	NA	NA	NA	NA	NA	NA
**hsa-miR-181c**	-0.09	-0.08	0.13	-0.01	0.07	0.01	0.03	0.04	1.08		1.11	1.10	0.28	-1.01	1.03	2.14	**1.22**	0.62	0.69	**1.00**	**1.00**	1	1_119	119	NA	NA	NA	NA	NA	NA
**hsa-miR-498**	0.00	0.10	0.00	0.03	-0.47	-0.39	-0.49	-0.45	-0.24	2.73	-1.79	0.23	-0.06	1.02	-1.37	1.18	**-1.04**	0.63	0.01	**1.00**	**1.00**	1	1_120	120	NA	NA	NA	NA	NA	NA
**hsa-miR-545**	-0.10	-0.06	0.21	0.01	0.21	-0.11	0.02	0.04					0.03	1.01	1.03	NA	**1.02**	0.63	0.76	**1.00**	**1.00**	1	1_121	121	NA	NA	NA	NA	NA	NA
**hsa-miR-642**	0.02	-0.24	0.09	-0.04	0.11	0.07	0.01	0.06					0.01	-1.03	1.04	NA	**1.01**	0.64	0.89	**1.00**	**1.00**	1	1_122	122	NA	NA	NA	NA	NA	NA
**hsa-miR-769-5p**	-0.12	0.09	-0.16	-0.06	0.10	-0.05	0.02	0.03	0.39		2.26	1.32	0.32	-1.04	1.02	2.50	**1.25**	0.65	0.45	**1.00**	**1.00**	1	1_123	123	NA	NA	NA	NA	NA	NA
**hsa-miR-383**	0.08	-0.21	-0.08	-0.07	0.09	0.07	0.04	0.07					0.00	-1.05	1.05	NA	**-1.00**	0.65	0.81	**1.00**	**1.00**	1	1_124	124	NA	NA	NA	NA	NA	NA
**hsa-miR-34a**	-0.06	-0.23	0.05	-0.08	0.17	-0.04	-0.08	0.02	3.11			3.11	0.42	-1.06	1.01	8.61	**1.34**	0.65	0.55	**1.00**	**1.00**	1	1_125	125	NA	NA	NA	NA	NA	NA
**hsa-miR-621**	0.31	-0.10	0.06	0.09	-0.21	-0.02	0.01	-0.07					0.01	1.06	-1.05	NA	**1.01**	0.65	0.69	**1.00**	**1.00**	1	1_126	126	NA	NA	NA	NA	NA	NA
**hsa-miR-218**	-0.07	0.16	0.13	0.07	0.08	-0.02	0.00	0.02					0.05	1.05	1.01	NA	**1.03**	0.65	0.95	**1.00**	**1.00**	1	1_127	127	NA	NA	NA	NA	NA	NA
**hsa-miR-92b**	-0.13	-0.02	-0.05	-0.07	0.01	0.03	0.10	0.05	-0.72	0.06	-0.15	-0.27	-0.10	-1.05	1.03	-1.21	**-1.07**	0.67	0.24	**1.00**	**1.00**	1	1_128	128	NA	NA	NA	NA	NA	NA
**hsa-miR-767-5p**	-0.08	-0.08	-0.09	-0.08	0.03	0.03	0.05	0.03	2.90			2.90	0.39	-1.06	1.02	7.48	**1.31**	0.67	0.74	**1.00**	**1.00**	1	1_129	129	NA	NA	NA	NA	NA	NA
**hsa-miR-588**	-0.02	0.45	-0.19	0.08	-0.07	-0.04	-0.02	-0.04					0.02	1.06	-1.03	NA	**1.01**	0.68	0.48	**1.00**	**1.00**	1	1_130	130	NA	NA	NA	NA	NA	NA
**hsa-miR-639**	0.17	-0.06	-0.12	0.00	-0.06	0.12	-0.09	-0.01					0.00	-1.00	-1.01	NA	**-1.00**	0.68	0.57	**1.00**	**1.00**	1	1_131	131	NA	NA	NA	NA	NA	NA
**hsa-let-7g**	-0.09	-0.22	-0.08	-0.13	-0.05	0.08	0.04	0.02	-3.63	1.81	-4.70	-2.17	-0.76	-1.09	1.02	-4.50	**-1.69**	0.68	0.03	**1.00**	**1.00**	1	1_132	132	NA	NA	NA	NA	NA	NA
**hsa-miR-181b**	0.00	0.01	-0.13	-0.04	0.00	-0.05	-0.04	-0.03	1.74	1.36	0.38	1.16	0.36	-1.03	-1.02	2.24	**1.29**	0.69	0.36	**1.00**	**1.00**	1	1_133	133	NA	NA	NA	NA	NA	NA
**hsa-miR-603**	-0.01	0.00	-0.09	-0.04	0.21	-0.01	0.04	0.08					0.02	-1.02	1.05	NA	**1.01**	0.69	0.90	**1.00**	**1.00**	1	1_134	134	NA	NA	NA	NA	NA	NA
**hsa-miR-631**	0.03	-0.03	0.21	0.07	-0.04	0.06	-0.13	-0.04					0.02	1.05	-1.03	NA	**1.01**	0.70	0.72	**1.00**	**1.00**	1	1_135	135	NA	NA	NA	NA	NA	NA
**hsa-miR-485-5p**	0.17	-0.41	0.19	-0.02	0.06	-0.01	-0.04	0.00					-0.01	-1.01	1.00	NA	**-1.01**	0.70	0.48	**1.00**	**1.00**	1	1_136	136	NA	NA	NA	NA	NA	NA
**hsa-miR-184**	0.60	-0.08	-0.46	0.02	-0.15	-0.16	-0.12	-0.14	0.90			0.90	0.08	1.01	-1.10	1.87	**1.06**	0.71	0.02	**1.00**	**1.00**	1	1_137	137	NA	NA	NA	NA	NA	NA
**hsa-miR-16**	-0.26	-0.19	-0.13	-0.20	-0.03	-0.12	-0.07	-0.07	2.86	0.97	3.39	2.41	0.71	-1.15	-1.05	5.31	**1.64**	0.71	0.04	**1.00**	**1.00**	1	1_138	138	NA	NA	NA	NA	NA	NA
**hsa-miR-153**	0.22	0.02	-0.14	0.03	0.10	-0.05	-0.05	0.00					0.02	1.02	1.00	NA	**1.01**	0.71	0.69	**1.00**	**1.00**	1	1_139	139	NA	NA	NA	NA	NA	NA
**hsa-miR-643**	-0.01	0.05	-0.01	0.01	0.06	0.08	-0.04	0.03					0.02	1.01	1.02	NA	**1.01**	0.71	0.90	**1.00**	**1.00**	1	1_140	140	NA	NA	NA	NA	NA	NA
**hsa-miR-137**	-0.09	-0.07	0.25	0.03	-0.10	0.04	-0.03	-0.03	1.12			1.12	0.16	1.02	-1.02	2.18	**1.12**	0.72	0.63	**1.00**	**1.00**	1	1_141	141	NA	NA	NA	NA	NA	NA
**hsa-miR-377**	0.02	-0.19	0.45	0.09	-0.06	-0.04	-0.04	-0.05					0.02	1.07	-1.03	NA	**1.02**	0.72	0.58	**1.00**	**1.00**	1	1_142	142	NA	NA	NA	NA	NA	NA
**hsa-miR-98**	0.03	-0.05	0.10	0.02	0.05	-0.09	-0.11	-0.05	-2.96	1.66	-3.40	-1.56	-0.53	1.02	-1.03	-2.96	**-1.44**	0.72	0.14	**1.00**	**1.00**	1	1_143	143	NA	NA	NA	NA	NA	NA
**hsa-miR-107**	-0.09	-0.13	0.03	-0.06	0.09	0.01	-0.07	0.01	0.76	1.54	-1.18	0.37	0.11	-1.05	1.00	1.29	**1.08**	0.73	0.29	**1.00**	**1.00**	1	1_144	144	NA	NA	NA	NA	NA	NA

ID	log2ratio_Agilent_1	log2ratio_Agilent_2	log2ratio_Agilent_3	avg_log2ratio_Agilent	log2ratio_Exiqon_1	log2ratio_Exiqon_2	log2ratio_Exiqon_3	avg_log2ratio_Exiqon	log2ratio_Illumina_1	log2ratio_Illumina_2	log2ratio_Illumina_3	avg_log2ratio_Illumina	avg_log2ratio_all	avg_FoldChange_Agilent	avg_FoldChange_Exiqon	avg_FoldChange_Illumina	avg_FoldChange_all	p.value.up	p.value.down	adjusted.p.value.up	adjusted.p.value.down	Index	Index_Rank	RPrank.1	RPrank.2	RPrank.3	RPrank.4	RPrank.5	RPrank.6	RPrank.7
hsa-miR-595	-0.24	-0.10	-0.14	-0.16	0.41	-0.01	-0.07	0.11					-0.03	-1.12	1.08	NA	-1.02	0.74	0.19	1.00	1.00	1	1_145	145	NA	NA	NA	NA	NA	NA
hsa-miR-212	-0.12	0.00	0.08	-0.01	0.03	-0.04	0.10	0.03					0.01	-1.01	1.02	NA	1.01	0.74	0.86	1.00	1.00	1	1_146	146	NA	NA	NA	NA	NA	NA
hsa-miR-515-5p	0.28	0.04	0.00	0.11	-0.07	-0.08	-0.06	-0.07					0.02	1.08	-1.05	NA	1.01	0.75	0.60	1.00	1.00	1	1_147	147	NA	NA	NA	NA	NA	NA
hsa-miR-431	0.02	-0.11	0.00	-0.03	-0.06	-0.05	0.16	0.02					-0.01	-1.02	1.01	NA	-1.00	0.75	0.72	1.00	1.00	1	1_148	148	NA	NA	NA	NA	NA	NA
hsa-miR-542-5p	-0.10	0.00	0.23	0.04	0.05	-0.01	0.00	0.01					0.03	1.03	1.01	NA	1.02	0.75	0.90	1.00	1.00	1	1_149	149	NA	NA	NA	NA	NA	NA
hsa-miR-142-5p	0.03	-0.07	0.07	0.01	0.03	0.03	0.02	0.03					0.02	1.01	1.02	NA	1.01	0.75	0.95	1.00	1.00	1	1_150	150	NA	NA	NA	NA	NA	NA
hsa-miR-32	-0.18	-0.07	0.01	-0.08	0.15	0.03	0.06	0.08					0.00	-1.06	1.06	NA	-1.00	0.75	0.78	1.00	1.00	1	1_151	151	NA	NA	NA	NA	NA	NA
hsa-miR-106b	-0.15	-0.17	-0.02	-0.11	0.05	0.02	-0.04	0.01	0.84	1.21	1.73	1.26	0.38	-1.08	1.01	2.39	1.31	0.76	0.27	1.00	1.00	1	1_152	152	NA	NA	NA	NA	NA	NA
hsa-miR-26a	-0.05	-0.12	-0.08	-0.09	-0.02	-0.09	0.06	-0.02	1.82	1.06	-0.28	0.87	0.25	-1.06	-1.01	1.82	1.19	0.76	0.25	1.00	1.00	1	1_153	153	NA	NA	NA	NA	NA	NA
hsa-miR-564	-0.24	0.11	0.26	0.04	-0.13	-0.01	-0.03	-0.06					-0.01	1.03	-1.04	NA	-1.00	0.76	0.40	1.00	1.00	1	1_154	154	NA	NA	NA	NA	NA	NA
hsa-miR-369-3p	-0.03	0.03	0.15	0.05	-0.26	-0.03	-0.02	-0.10	-3.01	0.34	-0.61	-1.10	-0.38	1.03	-1.07	-2.14	-1.30	0.77	0.14	1.00	1.00	1	1_155	155	NA	NA	NA	NA	NA	NA
hsa-miR-125b	-0.08	-0.11	-0.15	-0.11	-0.02	-0.13	-0.11	-0.09	1.41	1.33	2.84	1.86	0.55	-1.08	-1.06	3.63	1.47	0.77	0.11	1.00	1.00	1	1_156	156	NA	NA	NA	NA	NA	NA
hsa-let-7e	-0.09	-0.17	0.04	-0.07	0.04	0.06	-0.01	0.03	-3.32	1.14	-5.89	-2.69	-0.91	-1.05	1.02	-6.45	-1.88	0.77	0.08	1.00	1.00	1	1_157	157	NA	NA	NA	NA	NA	NA
hsa-miR-142-3p	-0.13	0.00	-0.09	-0.07	0.18	0.06	-0.03	0.07					0.00	-1.05	1.05	NA	-1.00	0.77	0.72	1.00	1.00	1	1_158	158	NA	NA	NA	NA	NA	NA
hsa-miR-185	-0.08	0.02	-0.01	-0.02	0.01	-0.12	-0.11	-0.07	2.09	0.83	1.24	1.38	0.43	-1.02	-1.05	2.61	1.35	0.78	0.19	1.00	1.00	1	1_159	159	NA	NA	NA	NA	NA	NA
hsa-miR-217	0.20	-0.06	-0.56	-0.14	-0.09	-0.01	0.02	-0.03	-0.01			-0.01	-0.07	-1.10	-1.02	-1.01	-1.05	0.78	0.18	1.00	1.00	1	1_160	160	NA	NA	NA	NA	NA	NA
hsa-miR-130a	-0.13	-0.04	0.04	-0.05	0.03	-0.08	-0.03	-0.03	2.31	-0.28	0.93	0.99	0.30	-1.03	-1.02	1.98	1.23	0.78	0.22	1.00	1.00	1	1_161	161	NA	NA	NA	NA	NA	NA
hsa-miR-519e	-0.34	-0.14	-0.24	-0.24	0.08	0.11	0.07	0.09					-0.08	-1.18	1.06	NA	-1.06	0.79	0.12	1.00	1.00	1	1_162	162	NA	NA	NA	NA	NA	NA
hsa-miR-511	-0.06	-0.14	0.07	-0.04	-0.09	0.14	-0.07	-0.01					-0.02	-1.03	-1.01	NA	-1.02	0.79	0.55	1.00	1.00	1	1_163	163	NA	NA	NA	NA	NA	NA
hsa-miR-611	0.08	-0.37	-0.02	-0.10	0.27	-0.16	-0.08	0.01					-0.05	-1.07	1.01	NA	-1.03	0.79	0.13	1.00	1.00	1	1_164	164	NA	NA	NA	NA	NA	NA
hsa-let-7f	0.07	-0.17	0.04	-0.02	-0.08	-0.09	0.01	-0.05	-3.45	1.22	-5.76	-2.66	-0.91	-1.01	-1.04	-6.34	-1.88	0.79	0.05	1.00	1.00	1	1_165	165	NA	NA	NA	NA	NA	NA
hsa-miR-449b	-0.14	0.20	-0.49	-0.14	0.04	0.00	0.04	0.02					-0.06	-1.10	1.02	NA	-1.04	0.79	0.34	1.00	1.00	1	1_166	166	NA	NA	NA	NA	NA	NA
hsa-miR-484	-0.06	0.00	0.03	-0.01	-0.02	0.04	0.00	0.01		-1.41		-1.41	-0.20	-1.01	1.01	-2.66	-1.15	0.80	0.55	1.00	1.00	1	1_167	167	NA	NA	NA	NA	NA	NA
hsa-miR-765	0.02	0.18	-0.02	0.06	0.09	-0.15	-0.19	-0.09			-2.45	-2.45	-0.36	1.04	-1.06	-5.47	-1.28	0.80	0.12	1.00	1.00	1	1_168	168	NA	NA	NA	NA	NA	NA
hsa-miR-191	-0.12	0.06	0.04	-0.01	-0.02	0.03	-0.09	-0.03	-0.77	0.45	-2.34	-0.88	-0.31	-1.00	-1.02	-1.85	-1.24	0.80	0.13	1.00	1.00	1	1_169	169	NA	NA	NA	NA	NA	NA
hsa-miR-572	0.10	-0.08	-0.17	-0.05	0.16	-0.01	-0.06	0.03					-0.01	-1.03	1.02	NA	-1.01	0.82	0.63	1.00	1.00	1	1_170	170	NA	NA	NA	NA	NA	NA
hsa-miR-429	-0.02	0.20	-0.60	-0.14	-0.12	-0.01	0.01	-0.04					-0.09	-1.10	-1.03	NA	-1.06	0.83	0.11	1.00	1.00	1	1_171	171	NA	NA	NA	NA	NA	NA
hsa-miR-10a	-0.13	-0.12	0.08	-0.06	-0.04	0.00	-0.05	-0.03	0.31	1.01	0.74	0.68	0.20	-1.04	-1.02	1.61	1.15	0.83	0.22	1.00	1.00	1	1_172	172	NA	NA	NA	NA	NA	NA
hsa-miR-606	-0.02	-0.11	0.09	-0.01	-0.01	0.05	-0.04	0.00	-0.40			-0.40	-0.06	-1.01	1.00	-1.32	-1.04	0.84	0.59	1.00	1.00	1	1_173	173	NA	NA	NA	NA	NA	NA
hsa-miR-214	-0.08	0.06	-0.24	-0.09	0.17	-0.03	-0.01	0.04					-0.02	-1.06	1.03	NA	-1.02	0.84	0.60	1.00	1.00	1	1_174	174	NA	NA	NA	NA	NA	NA
hsa-miR-145	0.19	-0.02	-0.26	-0.03	-0.01	-0.02	-0.02	-0.02					-0.02	-1.02	-1.01	NA	-1.02	0.84	0.54	1.00	1.00	1	1_175	175	NA	NA	NA	NA	NA	NA
hsa-miR-614	-0.02	0.17	0.05	0.07	-0.02	-0.02	-0.10	-0.05					0.01	1.05	-1.03	NA	1.01	0.84	0.71	1.00	1.00	1	1_176	176	NA	NA	NA	NA	NA	NA
hsa-miR-597	-0.05	-0.55	-0.04	-0.22	-0.08	0.11	0.04	0.02					-0.10	-1.16	1.02	NA	-1.07	0.85	0.16	1.00	1.00	1	1_177	177	NA	NA	NA	NA	NA	NA
hsa-miR-195	-0.07	-0.10	0.19	0.01	0.06	-0.06	0.00	0.00	-2.26			-2.26	-0.32	1.00	-1.00	-4.78	-1.25	0.86	0.47	1.00	1.00	1	1_178	178	NA	NA	NA	NA	NA	NA
hsa-miR-15b	-0.22	-0.30	-0.08	-0.20	0.09	-0.15	-0.04	-0.03	3.07	0.53		1.80	0.36	-1.15	-1.02	3.49	1.29	0.86	0.04	1.00	1.00	1	1_179	179	NA	NA	NA	NA	NA	NA
hsa-miR-551a	-0.27	-0.14	0.05	-0.12	0.10	0.03	-0.01	0.04	-0.01			-0.01	-0.04	-1.09	1.03	-1.01	-1.03	0.88	0.27	1.00	1.00	1	1_180	180	NA	NA	NA	NA	NA	NA

ID	log2ratio_Agilent_1	log2ratio_Agilent_2	log2ratio_Agilent_3	avg_log2ratio_Agilent	log2ratio_Exiqon_1	log2ratio_Exiqon_2	log2ratio_Exiqon_3	avg_log2ratio_Exiqon	log2ratio_Illumina_1	log2ratio_Illumina_2	log2ratio_Illumina_3	avg_log2ratio_Illumina	avg_log2ratio_all	avg_FoldChange_Agilent	avg_FoldChange_Exiqon	avg_FoldChange_Illumina	**avg_FoldChange_all**	p.value.up	p.value.down	**adjusted.p.value.up**	**adjusted.p.value.down**	Index	Index_Rank	RPrank.1	RPrank.2	RPrank.3	RPrank.4	RPrank.5	RPrank.6	RPrank.7
**hsa-miR-557**	0.04	-0.06	0.14	0.04	-0.99	-0.60	-0.83	-0.81		1.22		1.22	-0.16	1.03	-1.75	2.33	**-1.11**	0.88	0.01	**1.00**	**1.00**	1	1_181	181	NA	NA	NA	NA	NA	NA
**hsa-let-7d**	-0.02	-0.14	0.11	-0.01	-0.06	-0.20	-0.02	-0.09	-3.49	1.08	-5.12	-2.51	-0.87	-1.01	-1.07	-5.70	**-1.83**	0.88	0.03	**1.00**	**1.00**	1	1_182	182	NA	NA	NA	NA	NA	NA
**hsa-miR-200b**	-0.12	0.19	-0.19	-0.04	0.06	-0.03	-0.02	0.00					-0.02	-1.03	1.00	NA	**-1.01**	0.89	0.49	**1.00**	**1.00**	1	1_183	183	NA	NA	NA	NA	NA	NA
**hsa-miR-133a**	-0.22	0.19	0.02	0.00	-0.29	0.00	-0.04	-0.11					-0.05	1.00	-1.08	NA	**-1.04**	0.89	0.26	**1.00**	**1.00**	1	1_184	184	NA	NA	NA	NA	NA	NA
**hsa-miR-602**	0.07	0.19	-0.02	0.08	-0.70	-0.32	-0.45	-0.49					-0.20	1.06	-1.40	NA	**-1.15**	0.89	0.02	**1.00**	**1.00**	1	1_185	185	NA	NA	NA	NA	NA	NA
**hsa-miR-363**	0.03	-0.48	-0.57	-0.34	-0.06	0.12	-0.03	0.01					-0.17	-1.26	1.01	NA	**-1.12**	0.89	0.02	**1.00**	**1.00**	1	1_186	186	NA	NA	NA	NA	NA	NA
**hsa-miR-126**	-0.04	-0.06	0.02	-0.02	0.03	-0.03	-0.02	-0.01	0.97			0.97	0.13	-1.02	-1.00	1.95	**1.09**	0.89	0.69	**1.00**	**1.00**	1	1_187	187	NA	NA	NA	NA	NA	NA
**hsa-miR-618**	0.13	0.00	-0.44	-0.10	0.05	-0.07	-0.03	-0.02					-0.06	-1.07	-1.01	NA	**-1.04**	0.90	0.37	**1.00**	**1.00**	1	1_188	188	NA	NA	NA	NA	NA	NA
**hsa-miR-18b**	-0.12	-0.04	-0.05	-0.07	0.18	-0.08	0.01	0.04					-0.02	-1.05	1.03	NA	**-1.01**	0.90	0.62	**1.00**	**1.00**	1	1_189	189	NA	NA	NA	NA	NA	NA
**hsa-let-7a**	0.01	-0.24	0.04	-0.06	-0.14	-0.09	-0.08	-0.10	-3.34	1.29	-5.70	-2.58	-0.91	-1.04	-1.07	-5.98	**-1.88**	0.90	0.02	**1.00**	**1.00**	1	1_190	190	NA	NA	NA	NA	NA	NA
**hsa-miR-577**	0.03	-0.18	0.02	-0.04	-0.12	0.03	0.02	-0.02					-0.03	-1.03	-1.02	NA	**-1.02**	0.91	0.60	**1.00**	**1.00**	1	1_191	191	NA	NA	NA	NA	NA	NA
**hsa-miR-494**	0.28	-0.08	-0.15	0.02	-0.15	-0.06	-0.07	-0.09					-0.04	1.01	-1.07	NA	**-1.03**	0.91	0.21	**1.00**	**1.00**	1	1_192	192	NA	NA	NA	NA	NA	NA
**hsa-let-7c**	-0.07	-0.28	-0.06	-0.14	-0.18	0.03	-0.04	-0.06	-3.61	1.06	-6.01	-2.85	-1.02	-1.10	-1.04	-7.22	**-2.02**	0.92	0.00	**1.00**	**1.00**	1	1_193	193	NA	NA	NA	NA	NA	NA
**hsa-miR-521**	-0.04	0.02	0.03	0.00	0.03	-0.04	-0.01	-0.01					0.00	1.00	-1.01	NA	**-1.00**	0.92	0.83	**1.00**	**1.00**	1	1_194	194	NA	NA	NA	NA	NA	NA
**hsa-miR-19b**	-0.07	-0.07	0.03	-0.04	-0.02	-0.03	-0.09	-0.05	0.52	-0.04		0.24	0.03	-1.03	-1.03	1.18	**1.02**	0.92	0.26	**1.00**	**1.00**	1	1_195	195	NA	NA	NA	NA	NA	NA
**hsa-miR-612**	0.19	-0.08	-0.55	-0.15	0.05	-0.06	-0.09	-0.03					-0.09	-1.11	-1.02	NA	**-1.06**	0.92	0.14	**1.00**	**1.00**	1	1_196	196	NA	NA	NA	NA	NA	NA
**hsa-let-7b**	-0.12	-0.03	-0.04	-0.06	-0.12	-0.01	-0.10	-0.08	-3.51	1.14	-5.68	-2.68	-0.94	-1.04	-1.05	-6.43	**-1.92**	0.92	0.02	**1.00**	**1.00**	1	1_197	197	NA	NA	NA	NA	NA	NA
**hsa-miR-432**	-0.11	-0.51	0.02	-0.20	0.11	0.05	-0.04	0.04					-0.08	-1.15	1.03	NA	**-1.06**	0.93	0.25	**1.00**	**1.00**	1	1_198	198	NA	NA	NA	NA	NA	NA
**hsa-miR-346**	-0.17	-0.39	-0.50	-0.35	-0.03	0.02	0.03	0.01	-3.16	-2.45	-1.04	-2.22	-0.85	-1.28	1.00	-4.65	**-1.81**	0.93	0.00	**1.00**	**1.00**	1	1_199	199	NA	NA	NA	NA	NA	NA
**hsa-miR-324-5p**	-0.09	-0.16	0.03	-0.07	-0.04	0.03	0.03	0.01					-0.03	-1.05	1.00	NA	**-1.02**	0.93	0.66	**1.00**	**1.00**	1	1_200	200	NA	NA	NA	NA	NA	NA
**hsa-miR-566**	-0.13	-0.29	0.25	-0.06	-0.27	-0.12	0.04	-0.12					-0.09	-1.04	-1.09	NA	**-1.06**	0.94	0.09	**1.00**	**1.00**	1	1_201	201	NA	NA	NA	NA	NA	NA
**hsa-miR-328**	-0.48	0.06	0.04	-0.13	0.10	-0.08	-0.05	-0.01					-0.07	-1.09	-1.01	NA	**-1.05**	0.94	0.09	**1.00**	**1.00**	1	1_202	202	NA	NA	NA	NA	NA	NA
**hsa-miR-663**	0.04	0.09	-0.11	0.01	-1.40	-0.88	-1.33	-1.20		0.33		0.33	-0.46	1.00	-2.30	1.26	**-1.38**	0.95	0.00	**1.00**	**0.21**	1	1_203	203	NA	NA	NA	NA	NA	NA
**hsa-miR-19a**	-0.12	-0.17	-0.06	-0.12	0.00	-0.11	0.09	-0.01					-0.06	-1.08	-1.00	NA	**-1.04**	0.96	0.27	**1.00**	**1.00**	1	1_204	204	NA	NA	NA	NA	NA	NA
**hsa-miR-210**	-0.19	-0.14	0.00	-0.11	-0.30	-0.13	-0.31	-0.25	-2.66	-5.40	1.58	-2.16	-0.84	-1.08	-1.19	-4.48	**-1.79**	0.97	0.00	**1.00**	**0.42**	1	1_205	205	NA	NA	NA	NA	NA	NA
**hsa-miR-106a**	-0.10	-0.06	0.08	-0.03	0.04	-0.06	-0.04	-0.02					-0.02	-1.02	-1.01	NA	**-1.02**	0.97	0.61	**1.00**	**1.00**	1	1_206	206	NA	NA	NA	NA	NA	NA
**hsa-miR-365**	-0.17	-0.11	0.07	-0.07	0.03	-0.06	-0.13	-0.05		0.57		0.57	0.03	-1.05	-1.04	1.48	**1.02**	0.97	0.13	**1.00**	**1.00**	1	1_207	207	NA	NA	NA	NA	NA	NA
**hsa-miR-485-3p**	-0.08	-0.08	-0.16	-0.11	-0.03	-0.01	0.02	-0.01					-0.06	-1.08	-1.00	NA	**-1.04**	0.98	0.48	**1.00**	**1.00**	1	1_208	208	NA	NA	NA	NA	NA	NA
**hsa-miR-641**	-0.08	0.00	-0.01	-0.03	-0.08	-0.09	0.00	-0.06					-0.04	-1.02	-1.04	NA	**-1.03**	0.98	0.49	**1.00**	**1.00**	1	1_209	209	NA	NA	NA	NA	NA	NA
**hsa-miR-492**	0.06	-0.09	0.09	0.02	-1.47	-1.14	-1.47	-1.36					-0.67	1.02	-2.57	NA	**-1.59**	0.98	0.00	**1.00**	**0.03**	1	1_210	210	NA	NA	NA	NA	NA	NA
**hsa-miR-661**	0.09	-0.20	-0.34	-0.15	-0.01	-0.08	-0.04	-0.04					-0.09	-1.11	-1.03	NA	**-1.07**	0.98	0.17	**1.00**	**1.00**	1	1_211	211	NA	NA	NA	NA	NA	NA
**hsa-miR-451**	-0.15	-0.34	0.05	-0.15	0.01	-0.04	0.01	0.00					-0.08	-1.11	-1.00	NA	**-1.05**	0.98	0.27	**1.00**	**1.00**	1	1_212	212	NA	NA	NA	NA	NA	NA
**hsa-miR-381**	-0.07	0.04	-0.01	-0.02	-0.01	-0.11	-0.25	-0.12					-0.07	-1.01	-1.09	NA	**-1.05**	0.98	0.22	**1.00**	**1.00**	1	1_213	213	NA	NA	NA	NA	NA	NA
**hsa-miR-668**	0.13	-0.53	0.01	-0.13	-0.10	-0.13	-0.40	-0.21					-0.17	-1.10	-1.16	NA	**-1.13**	0.98	0.01	**1.00**	**1.00**	1	1_214	214	NA	NA	NA	NA	NA	NA
**hsa-miR-766**	0.02	-0.09	-0.13	-0.07	-0.12	-0.14	-0.31	-0.19		-2.27		-2.27	-0.43	-1.05	-1.14	-4.82	**-1.35**	0.99	0.01	**1.00**	**1.00**	1	1_215	215	NA	NA	NA	NA	NA	NA
**hsa-miR-623**	-0.01	0.07	-0.19	-0.04	-1.02	-0.67	-0.91	-0.87					-0.45	-1.03	-1.82	NA	**-1.37**	0.99	0.00	**1.00**	**0.30**	1	1_216	216	NA	NA	NA	NA	NA	NA

ID	log2ratio_Agilent_1	log2ratio_Agilent_2	log2ratio_Agilent_3	avg_log2ratio_Agilent	log2ratio_Exiqon_1	log2ratio_Exiqon_2	log2ratio_Exiqon_3	avg_log2ratio_Exiqon	log2ratio_Illumina_1	log2ratio_Illumina_2	log2ratio_Illumina_3	avg_log2ratio_Illumina	avg_log2ratio_all	avg_FoldChange_Agilent	avg_FoldChange_Exiqon	avg_FoldChange_Illumina	**avg_FoldChange_all**	p.value.up	p.value.down	**adjusted.p.value.up**	**adjusted.p.value.down**	Index	Index_Rank	RPrank.1	RPrank.2	RPrank.3	RPrank.4	RPrank.5	RPrank.6	RPrank.7
**hsa-miR-584**	-0.08	-0.24	-0.05	-0.13	-1.02	-0.75	-0.85	-0.87	1.83			1.83	-0.16	-1.09	-1.83	3.57	**-1.12**	0.99	0.00	**1.00**	**0.39**	1	1_217	217	NA	NA	NA	NA	NA	NA
**hsa-miR-197**	-0.08	-0.01	-0.26	-0.12	-0.43	-0.10	0.00	-0.18					-0.15	-1.08	-1.13	NA	**-1.11**	0.99	0.06	**1.00**	**1.00**	1	1_218	218	NA	NA	NA	NA	NA	NA
**hsa-miR-601**	0.01	-0.18	-0.23	-0.13	0.00	-0.09	-0.05	-0.05					-0.09	-1.09	-1.03	NA	**-1.06**	1.00	0.13	**1.00**	**1.00**	1	1_219	219	NA	NA	NA	NA	NA	NA
**hsa-miR-30b**	-0.20	-0.01	-0.02	-0.08	0.02	-0.09	-0.15	-0.07					-0.08	-1.06	-1.05	NA	**-1.05**	1.00	0.11	**1.00**	**1.00**	1	1_220	220	NA	NA	NA	NA	NA	NA
**hsa-miR-412**	-0.09	-0.09	-0.02	-0.07	-0.15	-0.03	-0.06	-0.08					-0.07	-1.05	-1.06	NA	**-1.05**	1.00	0.25	**1.00**	**1.00**	1	1_221	221	NA	NA	NA	NA	NA	NA
**hsa-miR-627**	-0.32	-0.09	-0.20	-0.20	0.04	-0.04	-0.06	-0.02					-0.11	-1.15	-1.01	NA	**-1.08**	1.00	0.04	**1.00**	**1.00**	1	1_222	222	NA	NA	NA	NA	NA	NA
**hsa-miR-646**	-0.21	-0.02	-0.19	-0.14	-0.27	-0.06	-0.04	-0.12					-0.13	-1.10	-1.09	NA	**-1.10**	1.00	0.05	**1.00**	**1.00**	1	1_223	223	NA	NA	NA	NA	NA	NA
**hsa-miR-518b**	-0.23	-0.13	-0.19	-0.18	-0.35	-0.06	-0.16	-0.19					-0.19	-1.14	-1.14	NA	**-1.14**	1.00	0.00	**1.00**	**1.00**	1	1_224	224	NA	NA	NA	NA	NA	NA
**hsa-miR-299-5p**	-0.21	-0.11	-0.09	-0.14	-0.32	-0.17	-0.17	-0.22					-0.18	-1.10	-1.17	NA	**-1.13**	1.00	0.00	**1.00**	**1.00**	1	1_225	225	NA	NA	NA	NA	NA	NA
**hsa-miR-9***	-0.01	-0.02	0.23	0.07	0.28	0.14	0.15	0.19					0.13	1.05	1.14	NA	**1.09**	0.00	0.99	**0.76**	**1.00**	2	2_1	NA	1	NA	NA	NA	NA	NA
**hsa-miR-539**	0.22	0.09	0.39	0.24	0.22	0.04	0.09	0.12					0.18	1.18	1.08	NA	**1.13**	0.00	1.00	**1.00**	**1.00**	2	2_2	NA	2	NA	NA	NA	NA	NA
**hsa-miR-302c***	0.30	0.06	0.46	0.27	0.04	0.01	0.10	0.05					0.16	1.21	1.03	NA	**1.12**	0.00	1.00	**1.00**	**1.00**	2	2_3	NA	3	NA	NA	NA	NA	NA
**hsa-miR-518f***	0.06	0.33	-0.06	0.11	0.31	0.07	0.11	0.16					0.14	1.08	1.12	NA	**1.10**	0.01	0.98	**1.00**	**1.00**	2	2_4	NA	4	NA	NA	NA	NA	NA
**hsa-miR-325**	0.25	0.07	-0.07	0.08	0.17	0.11	0.11	0.13					0.11	1.06	1.09	NA	**1.08**	0.02	0.99	**1.00**	**1.00**	2	2_5	NA	5	NA	NA	NA	NA	NA
**hsa-miR-182***	0.17	0.16	-0.11	0.08	0.14	0.12	0.13	0.13					0.10	1.05	1.09	NA	**1.07**	0.03	0.98	**1.00**	**1.00**	2	2_6	NA	6	NA	NA	NA	NA	NA
**hsa-miR-144**	0.01	0.06	0.22	0.10	-0.11	0.05	0.45	0.13					0.11	1.07	1.09	NA	**1.08**	0.04	0.91	**1.00**	**1.00**	2	2_7	NA	7	NA	NA	NA	NA	NA
**hsa-miR-367**	0.33	0.56	0.02	0.31	-0.06	-0.03	0.09	0.00					0.15	1.24	-1.00	NA	**1.11**	0.04	0.94	**1.00**	**1.00**	2	2_8	NA	8	NA	NA	NA	NA	NA
**hsa-miR-18a***	0.10	0.08	-0.01	0.06	0.14	0.03	0.17	0.11					0.09	1.04	1.08	NA	**1.06**	0.05	0.98	**1.00**	**1.00**	2	2_9	NA	9	NA	NA	NA	NA	NA
**hsa-miR-619**	0.46	0.08	-0.01	0.18	0.06	0.09	-0.03	0.04					0.11	1.13	1.03	NA	**1.08**	0.06	0.94	**1.00**	**1.00**	2	2_10	NA	10	NA	NA	NA	NA	NA
**hsa-miR-363***	-0.07	0.68	0.25	0.29	0.19	-0.14	-0.29	-0.08					0.10	1.22	-1.06	NA	**1.07**	0.06	0.14	**1.00**	**1.00**	2	2_11	NA	11	NA	NA	NA	NA	NA
**hsa-miR-555**	0.24	0.61	-0.29	0.19	-0.07	0.05	0.04	0.00					0.10	1.14	1.00	NA	**1.07**	0.07	0.63	**1.00**	**1.00**	2	2_12	NA	12	NA	NA	NA	NA	NA
**hsa-miR-514**	-0.19	0.06	0.38	0.08	0.23	-0.02	0.01	0.07					0.08	1.06	1.05	NA	**1.05**	0.10	0.75	**1.00**	**1.00**	2	2_13	NA	13	NA	NA	NA	NA	NA
**hsa-miR-569**	-0.06	0.59	0.04	0.19	-0.17	0.06	0.08	-0.01					0.09	1.14	-1.01	NA	**1.07**	0.10	0.72	**1.00**	**1.00**	2	2_14	NA	14	NA	NA	NA	NA	NA
**hsa-miR-520e**	0.23	-0.22	0.12	0.04	0.09	0.14	-0.02	0.07					0.06	1.03	1.05	NA	**1.04**	0.11	0.84	**1.00**	**1.00**	2	2_15	NA	15	NA	NA	NA	NA	NA
**hsa-miR-507**	0.09	0.02	0.04	0.05	0.32	-0.02	-0.03	0.09					0.07	1.04	1.07	NA	**1.05**	0.11	0.90	**1.00**	**1.00**	2	2_16	NA	16	NA	NA	NA	NA	NA
**hsa-miR-568**	-0.15	0.06	0.18	0.03	0.04	0.16	-0.04	0.05					0.04	1.02	1.04	NA	**1.03**	0.14	0.68	**1.00**	**1.00**	2	2_17	NA	17	NA	NA	NA	NA	NA
**hsa-miR-410**	-0.07	0.15	0.02	0.04	0.24	0.10	-0.19	0.05					0.04	1.02	1.04	NA	**1.03**	0.15	0.49	**1.00**	**1.00**	2	2_18	NA	18	NA	NA	NA	NA	NA
**hsa-miR-617**	-0.03	0.56	0.15	0.23	0.09	-0.05	0.00	0.02					0.12	1.17	1.01	NA	**1.09**	0.17	0.87	**1.00**	**1.00**	2	2_19	NA	19	NA	NA	NA	NA	NA
**hsa-miR-432***	-0.06	-0.20	0.13	-0.04	0.02	0.09	0.12	0.07					0.02	-1.03	1.05	NA	**1.01**	0.17	0.78	**1.00**	**1.00**	2	2_20	NA	20	NA	NA	NA	NA	NA
**hsa-miR-802**	0.33	0.20	-0.15	0.13	0.09	-0.09	0.03	0.01					0.07	1.09	1.01	NA	**1.05**	0.18	0.69	**1.00**	**1.00**	2	2_21	NA	21	NA	NA	NA	NA	NA
**hsa-miR-586**	0.56	-0.12	0.06	0.17	-0.09	-0.01	-0.02	-0.04					0.07	1.12	-1.03	NA	**1.05**	0.19	0.72	**1.00**	**1.00**	2	2_22	NA	22	NA	NA	NA	NA	NA
**hsa-miR-587**	0.17	0.19	-0.26	0.03	-0.06	0.12	0.01	0.02					0.03	1.02	1.02	NA	**1.02**	0.19	0.63	**1.00**	**1.00**	2	2_23	NA	23	NA	NA	NA	NA	NA
**hsa-miR-561**	0.07	0.15	0.21	0.14	0.11	-0.03	0.01	0.03					0.09	1.10	1.02	NA	**1.06**	0.20	0.96	**1.00**	**1.00**	2	2_24	NA	24	NA	NA	NA	NA	NA
**hsa-miR-758**	0.20	0.26	-0.13	0.11	0.02	0.07	0.00	0.03					0.07	1.08	1.02	NA	**1.05**	0.21	0.88	**1.00**	**1.00**	2	2_25	NA	25	NA	NA	NA	NA	NA
**hsa-miR-373**	0.28	-0.04	0.27	0.17	0.10	-0.15	-0.08	-0.04					0.06	1.13	-1.03	NA	**1.05**	0.22	0.36	**1.00**	**1.00**	2	2_26	NA	26	NA	NA	NA	NA	NA
**hsa-miR-626**	-0.16	-0.07	0.10	-0.04	0.01	0.04	0.15	0.07					0.01	-1.03	1.05	NA	**1.01**	0.23	0.70	**1.00**	**1.00**	2	2_27	NA	27	NA	NA	NA	NA	NA

**ID**	log2ratio_Agilent_1	log2ratio_Agilent_2	log2ratio_Agilent_3	avg_log2ratio_Agilent	log2ratio_Exiqon_1	log2ratio_Exiqon_2	log2ratio_Exiqon_3	avg_log2ratio_Exiqon	log2ratio_Illumina_1	log2ratio_Illumina_2	log2ratio_Illumina_3	avg_log2ratio_Illumina	avg_log2ratio_all	avg_FoldChange_Agilent	avg_FoldChange_Exiqon	avg_FoldChange_Illumina	**avg_FoldChange_all**	p.value.up	p.value.down	**adjusted.p.value.up**	**adjusted.p.value.down**	Index	Index_Rank	RPrank.1	RPrank.2	RPrank.3	RPrank.4	RPrank.5	RPrank.6	RPrank.7
**hsa-miR-411**	0.17	0.46	-0.10	0.18	0.08	0.04	-0.04	0.02					0.10	1.13	1.02	NA	**1.07**	0.23	0.86	**1.00**	**1.00**	2	2_28	NA	28	NA	NA	NA	NA	NA
**hsa-miR-369-5p**	0.15	0.01	0.26	0.14	-0.26	-0.02	0.08	-0.07					0.03	1.10	-1.05	NA	**1.02**	0.24	0.55	**1.00**	**1.00**	2	2_29	NA	29	NA	NA	NA	NA	NA
**hsa-miR-526b**	0.20	0.27	0.05	0.17	-0.04	-0.07	0.07	-0.01					0.08	1.12	-1.01	NA	**1.06**	0.25	0.85	**1.00**	**1.00**	2	2_30	NA	30	NA	NA	NA	NA	NA
**hsa-miR-517***	0.42	0.04	-0.23	0.07	0.17	-0.19	0.01	0.00					0.04	1.05	-1.00	NA	**1.02**	0.25	0.19	**1.00**	**1.00**	2	2_31	NA	31	NA	NA	NA	NA	NA
**hsa-miR-519e***	0.05	0.06	-0.02	0.03	0.00	0.00	0.14	0.05					0.04	1.02	1.03	NA	**1.03**	0.26	0.90	**1.00**	**1.00**	2	2_32	NA	32	NA	NA	NA	NA	NA
**hsa-miR-659**	0.18	-0.16	0.25	0.09	-0.01	0.04	0.00	0.01					0.05	1.06	1.01	NA	**1.03**	0.29	0.85	**1.00**	**1.00**	2	2_33	NA	33	NA	NA	NA	NA	NA
**hsa-miR-409-3p**	0.05	0.06	0.13	0.08	0.01	0.04	0.03	0.03					0.06	1.06	1.02	NA	**1.04**	0.30	0.96	**1.00**	**1.00**	2	2_34	NA	34	NA	NA	NA	NA	NA
**hsa-miR-134**	0.19	0.00	0.10	0.09	0.12	0.00	-0.01	0.03					0.06	1.07	1.02	NA	**1.05**	0.31	0.94	**1.00**	**1.00**	2	2_35	NA	35	NA	NA	NA	NA	NA
**hsa-miR-320**	0.08	0.22	-0.21	0.03	0.09	0.04	0.01	0.04					0.04	1.02	1.03	NA	**1.03**	0.31	0.79	**1.00**	**1.00**	2	2_36	NA	36	NA	NA	NA	NA	NA
**hsa-miR-630**	-0.23	0.03	0.01	-0.07	0.30	0.03	-0.06	0.09					0.01	-1.05	1.07	NA	**1.01**	0.32	0.40	**1.00**	**1.00**	2	2_37	NA	37	NA	NA	NA	NA	NA
**hsa-miR-767-3p**	0.18	0.04	0.12	0.11	0.16	-0.06	-0.01	0.03					0.07	1.08	1.02	NA	**1.05**	0.32	0.88	**1.00**	**1.00**	2	2_38	NA	38	NA	NA	NA	NA	NA
**hsa-miR-544**	0.07	-0.05	0.29	0.10	-0.19	0.05	-0.04	-0.06					0.02	1.08	-1.04	NA	**1.02**	0.32	0.55	**1.00**	**1.00**	2	2_39	NA	39	NA	NA	NA	NA	NA
**hsa-miR-200a***	0.22	0.11	0.28	0.20	-0.12	-0.09	-0.12	-0.11					0.05	1.15	-1.08	NA	**1.03**	0.33	0.35	**1.00**	**1.00**	2	2_40	NA	40	NA	NA	NA	NA	NA
**hsa-miR-567**	-0.03	0.59	0.09	0.22	0.03	-0.02	-0.11	-0.03					0.09	1.16	-1.02	NA	**1.06**	0.33	0.54	**1.00**	**1.00**	2	2_41	NA	41	NA	NA	NA	NA	NA
**hsa-miR-133b**	-0.02	-0.07	0.25	0.05	-0.20	0.01	0.07	-0.04					0.01	1.04	-1.03	NA	**1.01**	0.35	0.56	**1.00**	**1.00**	2	2_42	NA	42	NA	NA	NA	NA	NA
**hsa-miR-649**	-0.05	-0.04	-0.18	-0.09	-0.13	0.18	-0.04	0.00					-0.04	-1.06	1.00	NA	**-1.03**	0.35	0.30	**1.00**	**1.00**	2	2_43	NA	43	NA	NA	NA	NA	NA
**hsa-miR-510**	0.18	0.19	0.14	0.17	-0.09	0.00	-0.04	-0.04					0.06	1.13	-1.03	NA	**1.04**	0.36	0.81	**1.00**	**1.00**	2	2_44	NA	44	NA	NA	NA	NA	NA
**hsa-miR-20b**	0.00	-0.03	0.12	0.03	0.12	-0.02	0.04	0.05					0.04	1.02	1.03	NA	**1.03**	0.37	0.90	**1.00**	**1.00**	2	2_45	NA	45	NA	NA	NA	NA	NA
**hsa-miR-656**	0.12	-0.07	-0.01	0.01	0.05	0.02	0.06	0.04					0.03	1.01	1.03	NA	**1.02**	0.40	0.91	**1.00**	**1.00**	2	2_46	NA	46	NA	NA	NA	NA	NA
**hsa-miR-204**	0.16	0.46	0.00	0.20	0.01	-0.06	-0.02	-0.02					0.09	1.15	-1.02	NA	**1.06**	0.41	0.75	**1.00**	**1.00**	2	2_47	NA	47	NA	NA	NA	NA	NA
**hsa-miR-600**	-0.08	-0.35	0.39	-0.01	0.06	-0.06	0.00	0.00					-0.01	-1.01	-1.00	NA	**-1.01**	0.41	0.25	**1.00**	**1.00**	2	2_48	NA	48	NA	NA	NA	NA	NA
**hsa-miR-515-3p**	-0.36	0.19	-0.02	-0.06	-0.06	0.01	0.09	0.01					-0.02	-1.04	1.01	NA	**-1.02**	0.41	0.33	**1.00**	**1.00**	2	2_49	NA	49	NA	NA	NA	NA	NA
**hsa-miR-512-5p**	0.04	0.05	0.19	0.09	-0.10	0.05	-0.01	-0.02					0.04	1.06	-1.01	NA	**1.02**	0.41	0.84	**1.00**	**1.00**	2	2_50	NA	50	NA	NA	NA	NA	NA
**hsa-miR-504**	-0.27	-0.28	0.07	-0.16	0.24	-0.04	0.03	0.08					-0.04	-1.12	1.06	NA	**-1.03**	0.41	0.28	**1.00**	**1.00**	2	2_51	NA	51	NA	NA	NA	NA	NA
**hsa-miR-605**	0.42	-0.06	-0.70	-0.11	-0.02	-0.01	-0.02	-0.02					-0.06	-1.08	-1.01	NA	**-1.05**	0.42	0.17	**1.00**	**1.00**	2	2_52	NA	52	NA	NA	NA	NA	NA
**hsa-miR-647**	0.28	-0.09	0.08	0.09	-0.05	0.04	-0.07	-0.03					0.03	1.06	-1.02	NA	**1.02**	0.44	0.65	**1.00**	**1.00**	2	2_53	NA	53	NA	NA	NA	NA	NA
**hsa-miR-506**	0.03	-0.18	0.27	0.04	0.04	-0.06	0.02	0.00					0.02	1.03	1.00	NA	**1.01**	0.45	0.67	**1.00**	**1.00**	2	2_54	NA	54	NA	NA	NA	NA	NA
**hsa-miR-141**	0.02	0.42	-0.09	0.12	0.05	-0.20	0.03	-0.04					0.04	1.09	-1.03	NA	**1.03**	0.45	0.21	**1.00**	**1.00**	2	2_55	NA	55	NA	NA	NA	NA	NA
**hsa-miR-302a**	0.24	-0.15	0.19	0.09	0.08	-0.09	-0.09	-0.03					0.03	1.07	-1.02	NA	**1.02**	0.45	0.41	**1.00**	**1.00**	2	2_56	NA	56	NA	NA	NA	NA	NA
**hsa-miR-604**	0.06	0.29	0.01	0.12	0.03	-0.03	-0.02	-0.01					0.06	1.09	-1.00	NA	**1.04**	0.47	0.84	**1.00**	**1.00**	2	2_57	NA	57	NA	NA	NA	NA	NA
**hsa-miR-302c**	0.26	0.16	-0.13	0.09	-0.24	-0.10	0.04	-0.10					0.00	1.07	-1.07	NA	**-1.00**	0.49	0.23	**1.00**	**1.00**	2	2_58	NA	58	NA	NA	NA	NA	NA
**hsa-miR-578**	0.17	-0.20	-0.05	-0.03	-0.05	0.08	0.02	0.02					0.00	-1.02	1.01	NA	**-1.00**	0.49	0.68	**1.00**	**1.00**	2	2_59	NA	59	NA	NA	NA	NA	NA
**hsa-miR-563**	0.10	0.05	-0.11	0.01	-0.14	0.12	-0.09	-0.04					-0.01	1.01	-1.03	NA	**-1.01**	0.49	0.37	**1.00**	**1.00**	2	2_60	NA	60	NA	NA	NA	NA	NA
**hsa-miR-135a**	0.02	-0.09	-0.14	-0.07	0.20	0.06	-0.06	0.07					0.00	-1.05	1.05	NA	**-1.00**	0.50	0.50	**1.00**	**1.00**	2	2_61	NA	61	NA	NA	NA	NA	NA
**hsa-miR-562**	0.11	0.15	-0.27	-0.01	-0.46	0.10	-0.05	-0.14					-0.07	-1.00	-1.10	NA	**-1.05**	0.51	0.05	**1.00**	**1.00**	2	2_62	NA	62	NA	NA	NA	NA	NA
**hsa-miR-633**	0.07	-0.15	0.15	0.02	-0.24	0.06	-0.01	-0.07					-0.02	1.02	-1.05	NA	**-1.02**	0.53	0.41	**1.00**	**1.00**	2	2_63	NA	63	NA	NA	NA	NA	NA

**ID**	log2ratio_Agilent_1	log2ratio_Agilent_2	log2ratio_Agilent_3	avg_log2ratio_Agilent	log2ratio_Exiqon_1	log2ratio_Exiqon_2	log2ratio_Exiqon_3	avg_log2ratio_Exiqon	log2ratio_Illumina_1	log2ratio_Illumina_2	log2ratio_Illumina_3	avg_log2ratio_Illumina	avg_log2ratio_all	avg_FoldChange_Agilent	avg_FoldChange_Exiqon	avg_FoldChange_Illumina	**avg_FoldChange_all**	p.value.up	p.value.down	**adjusted.p.value.up**	**adjusted.p.value.down**	Index	Index_Rank	RPrank.1	RPrank.2	RPrank.3	RPrank.4	RPrank.5	RPrank.6	RPrank.7
**hsa-miR-496**	-0.08	0.06	0.11	0.03	-0.14	0.00	0.06	-0.03					0.00	1.02	-1.02	NA	**1.00**	0.53	0.68	**1.00**	**1.00**	2	2_64	NA	64	NA	NA	NA	NA	NA
**hsa-miR-302b**	0.01	0.01	-0.55	-0.18	0.05	-0.02	0.09	0.04					-0.07	-1.13	1.03	NA	**-1.05**	0.53	0.36	**1.00**	**1.00**	2	2_65	NA	65	NA	NA	NA	NA	NA
**hsa-miR-453**	0.30	-0.14	0.03	0.06	-0.17	-0.08	0.02	-0.07					-0.01	1.04	-1.05	NA	**-1.00**	0.54	0.44	**1.00**	**1.00**	2	2_66	NA	66	NA	NA	NA	NA	NA
**hsa-miR-613**	0.21	-0.09	-0.05	0.02	0.02	-0.07	0.06	0.00					0.01	1.02	1.00	NA	**1.01**	0.54	0.68	**1.00**	**1.00**	2	2_67	NA	67	NA	NA	NA	NA	NA
**hsa-miR-560**	0.06	-0.37	0.13	-0.06	0.20	-0.05	-0.04	0.03					-0.01	-1.04	1.02	NA	**-1.01**	0.56	0.28	**1.00**	**1.00**	2	2_68	NA	68	NA	NA	NA	NA	NA
**hsa-miR-523**	0.09	0.12	-0.04	0.06	0.11	-0.05	-0.06	0.00					0.03	1.04	1.00	NA	**1.02**	0.57	0.66	**1.00**	**1.00**	2	2_69	NA	69	NA	NA	NA	NA	NA
**hsa-miR-191***	0.08	-0.01	-0.07	0.00	0.19	-0.06	-0.01	0.04					0.02	-1.00	1.03	NA	**1.01**	0.57	0.69	**1.00**	**1.00**	2	2_70	NA	70	NA	NA	NA	NA	NA
**hsa-miR-648**	0.02	0.27	-0.08	0.07	-0.08	-0.05	0.04	-0.03					0.02	1.05	-1.02	NA	**1.01**	0.57	0.62	**1.00**	**1.00**	2	2_71	NA	71	NA	NA	NA	NA	NA
**hsa-miR-591**	-0.30	0.17	-0.13	-0.09	0.23	-0.14	-0.05	0.01					-0.04	-1.06	1.01	NA	**-1.03**	0.59	0.07	**1.00**	**1.00**	2	2_72	NA	72	NA	NA	NA	NA	NA
**hsa-miR-655**	-0.11	-0.56	0.07	-0.20	-0.01	0.06	0.03	0.03					-0.09	-1.15	1.02	NA	**-1.06**	0.60	0.15	**1.00**	**1.00**	2	2_73	NA	73	NA	NA	NA	NA	NA
**hsa-miR-220**	0.29	-0.33	-0.12	-0.05	0.05	-0.05	-0.01	0.00					-0.03	-1.04	-1.00	NA	**-1.02**	0.62	0.33	**1.00**	**1.00**	2	2_74	NA	74	NA	NA	NA	NA	NA
**hsa-miR-609**	0.05	-0.28	0.07	-0.05	-0.01	-0.04	0.07	0.01					-0.02	-1.04	1.00	NA	**-1.02**	0.63	0.53	**1.00**	**1.00**	2	2_75	NA	75	NA	NA	NA	NA	NA
**hsa-miR-801**	0.03	-0.18	0.08	-0.02	0.10	0.03	-0.17	-0.01					-0.02	-1.02	-1.01	NA	**-1.01**	0.63	0.40	**1.00**	**1.00**	2	2_76	NA	76	NA	NA	NA	NA	NA
**hsa-miR-637**	0.23	-0.16	0.13	0.07	-0.44	0.01	-0.32	-0.25					-0.09	1.05	-1.19	NA	**-1.06**	0.65	0.02	**1.00**	**1.00**	2	2_77	NA	77	NA	NA	NA	NA	NA
**hsa-miR-136**	-0.04	0.06	0.01	0.01	0.01	-0.04	0.05	0.01					0.01	1.01	1.00	NA	**1.01**	0.65	0.74	**1.00**	**1.00**	2	2_78	NA	78	NA	NA	NA	NA	NA
**hsa-miR-302d**	0.06	-0.12	0.13	0.03	-0.16	0.05	-0.11	-0.07					-0.02	1.02	-1.05	NA	**-1.02**	0.67	0.34	**1.00**	**1.00**	2	2_79	NA	79	NA	NA	NA	NA	NA
**hsa-miR-384**	0.11	-0.16	0.02	-0.01	-0.23	-0.04	0.07	-0.07					-0.04	-1.01	-1.05	NA	**-1.03**	0.67	0.38	**1.00**	**1.00**	2	2_80	NA	80	NA	NA	NA	NA	NA
**hsa-miR-495**	0.30	-0.09	-0.20	0.00	0.04	-0.05	-0.22	-0.08					-0.04	1.00	-1.05	NA	**-1.03**	0.69	0.12	**1.00**	**1.00**	2	2_81	NA	81	NA	NA	NA	NA	NA
**hsa-miR-559**	0.02	0.09	-0.11	0.00	-0.06	-0.04	0.04	-0.02					-0.01	-1.00	-1.01	NA	**-1.01**	0.70	0.63	**1.00**	**1.00**	2	2_82	NA	82	NA	NA	NA	NA	NA
**hsa-miR-565**	-0.07	0.04	-0.11	-0.04	-0.17	0.09	-0.01	-0.03					-0.04	-1.03	-1.02	NA	**-1.03**	0.72	0.41	**1.00**	**1.00**	2	2_83	NA	83	NA	NA	NA	NA	NA
**hsa-miR-585**	-0.47	0.19	-0.03	-0.10	-0.01	-0.04	-0.01	-0.02					-0.06	-1.08	-1.01	NA	**-1.04**	0.74	0.15	**1.00**	**1.00**	2	2_84	NA	84	NA	NA	NA	NA	NA
**hsa-miR-575**	-0.22	0.03	-0.04	-0.08	0.20	-0.17	-0.05	-0.01					-0.04	-1.05	-1.00	NA	**-1.03**	0.75	0.07	**1.00**	**1.00**	2	2_85	NA	85	NA	NA	NA	NA	NA
**hsa-miR-205**	0.01	-0.05	-0.12	-0.05	-0.10	0.09	-0.12	-0.04					-0.05	-1.04	-1.03	NA	**-1.03**	0.76	0.23	**1.00**	**1.00**	2	2_86	NA	86	NA	NA	NA	NA	NA
**hsa-miR-658**	-0.07	0.37	0.02	0.10	-0.14	-0.05	-0.10	-0.10					0.00	1.08	-1.07	NA	**1.00**	0.76	0.21	**1.00**	**1.00**	2	2_87	NA	87	NA	NA	NA	NA	NA
**hsa-miR-770-5p**	-0.35	-0.24	0.10	-0.16	-0.01	-0.09	0.08	-0.01					-0.08	-1.12	-1.01	NA	**-1.06**	0.76	0.08	**1.00**	**1.00**	2	2_88	NA	88	NA	NA	NA	NA	NA
**hsa-miR-620**	0.00	0.09	-0.16	-0.02	0.08	-0.02	-0.05	0.00					-0.01	-1.02	1.00	NA	**-1.01**	0.77	0.44	**1.00**	**1.00**	2	2_89	NA	89	NA	NA	NA	NA	NA
**hsa-miR-645**	0.04	-0.10	-0.06	-0.04	-0.01	0.01	0.00	0.00					-0.02	-1.03	1.00	NA	**-1.01**	0.77	0.68	**1.00**	**1.00**	2	2_90	NA	90	NA	NA	NA	NA	NA
**hsa-miR-223**	0.00	0.04	-0.04	0.00	-0.06	-0.10	0.05	-0.04					-0.02	1.00	-1.02	NA	**-1.01**	0.77	0.44	**1.00**	**1.00**	2	2_91	NA	91	NA	NA	NA	NA	NA
**hsa-miR-181a***	-0.08	-0.10	0.02	-0.05	0.17	-0.05	-0.06	0.02					-0.02	-1.04	1.02	NA	**-1.01**	0.78	0.37	**1.00**	**1.00**	2	2_92	NA	92	NA	NA	NA	NA	NA
**hsa-miR-409-5p**	0.07	-0.10	-0.19	-0.07	-0.35	0.01	0.05	-0.10					-0.09	-1.05	-1.07	NA	**-1.06**	0.78	0.16	**1.00**	**1.00**	2	2_93	NA	93	NA	NA	NA	NA	NA
**hsa-miR-634**	0.02	-0.20	-0.02	-0.07	0.01	0.04	-0.02	0.01					-0.03	-1.05	1.01	NA	**-1.02**	0.79	0.55	**1.00**	**1.00**	2	2_94	NA	94	NA	NA	NA	NA	NA
**hsa-miR-640**	-0.04	-0.15	0.07	-0.04	-0.17	-0.01	0.03	-0.05					-0.04	-1.03	-1.04	NA	**-1.03**	0.80	0.43	**1.00**	**1.00**	2	2_95	NA	95	NA	NA	NA	NA	NA
**hsa-miR-552**	-0.09	0.12	0.12	0.05	-0.17	-0.10	-0.01	-0.09					-0.02	1.04	-1.07	NA	**-1.02**	0.80	0.24	**1.00**	**1.00**	2	2_96	NA	96	NA	NA	NA	NA	NA
**hsa-miR-520g**	0.07	-0.29	0.05	-0.06	-0.06	-0.03	0.01	-0.03					-0.04	-1.04	-1.02	NA	**-1.03**	0.80	0.45	**1.00**	**1.00**	2	2_97	NA	97	NA	NA	NA	NA	NA
**hsa-miR-211**	-0.20	-0.34	0.21	-0.11	-0.04	-0.02	-0.02	-0.03					-0.07	-1.08	-1.02	NA	**-1.05**	0.80	0.15	**1.00**	**1.00**	2	2_98	NA	98	NA	NA	NA	NA	NA
**hsa-miR-607**	0.17	-0.13	-0.48	-0.15	-0.11	0.01	-0.01	-0.04					-0.09	-1.11	-1.03	NA	**-1.07**	0.83	0.23	**1.00**	**1.00**	2	2_99	NA	99	NA	NA	NA	NA	NA

ID	log2ratio_Agilent_1	log2ratio_Agilent_2	log2ratio_Agilent_3	avg_log2ratio_Agilent	log2ratio_Exiqon_1	log2ratio_Exiqon_2	log2ratio_Exiqon_3	avg_log2ratio_Exiqon	log2ratio_Illumina_1	log2ratio_Illumina_2	log2ratio_Illumina_3	avg_log2ratio_Illumina	avg_log2ratio_all	avg_FoldChange_Agilent	avg_FoldChange_Exiqon	avg_FoldChange_Illumina	avg_FoldChange_all	p.value.up	p.value.down	adjusted.p.value.up	adjusted.p.value.down	Index	Index_Rank	RPrank.1	RPrank.2	RPrank.3	RPrank.4	RPrank.5	RPrank.6	RPrank.7
hsa-miR-155	-0.15	-0.06	-0.04	-0.08	0.06	-0.03	0.00	0.01					-0.04	-1.06	1.01	NA	-1.03	0.83	0.50	1.00	1.00	2	2_100	NA	100	NA	NA	NA	NA	NA
hsa-miR-376a	0.07	-0.14	-0.13	-0.07	-0.13	0.00	0.01	-0.04					-0.05	-1.05	-1.03	NA	-1.04	0.85	0.45	1.00	1.00	2	2_101	NA	101	NA	NA	NA	NA	NA
hsa-miR-154	0.00	-0.16	-0.18	-0.11	0.04	-0.02	0.02	0.01					-0.05	-1.08	1.01	NA	-1.04	0.85	0.42	1.00	1.00	2	2_102	NA	102	NA	NA	NA	NA	NA
hsa-miR-487a	-0.16	-0.02	-0.06	-0.08	-0.14	0.03	0.00	-0.04					-0.06	-1.06	-1.02	NA	-1.04	0.87	0.32	1.00	1.00	2	2_103	NA	103	NA	NA	NA	NA	NA
hsa-miR-599	-0.01	-0.30	-0.10	-0.14	-0.12	0.07	-0.05	-0.03					-0.09	-1.10	-1.02	NA	-1.06	0.89	0.16	1.00	1.00	2	2_104	NA	104	NA	NA	NA	NA	NA
hsa-miR-126*	-0.06	-0.23	-0.03	-0.10	-0.06	-0.04	0.02	-0.02					-0.06	-1.08	-1.02	NA	-1.05	0.91	0.37	1.00	1.00	2	2_105	NA	105	NA	NA	NA	NA	NA
hsa-miR-768-3p	-0.11	-0.19	0.02	-0.09	0.05	-0.06	-0.03	-0.01					-0.05	-1.07	-1.01	NA	-1.04	0.92	0.29	1.00	1.00	2	2_106	NA	106	NA	NA	NA	NA	NA
hsa-miR-635	-0.05	-0.24	-0.08	-0.12	0.03	0.00	-0.04	0.00					-0.06	-1.09	-1.00	NA	-1.04	0.92	0.31	1.00	1.00	2	2_107	NA	107	NA	NA	NA	NA	NA
hsa-miR-583	0.09	-0.06	-0.05	-0.01	-0.15	-0.15	-0.01	-0.10					-0.06	-1.00	-1.08	NA	-1.04	0.92	0.18	1.00	1.00	2	2_108	NA	108	NA	NA	NA	NA	NA
hsa-miR-147	-0.09	-0.25	-0.04	-0.12	0.09	-0.09	-0.03	-0.01					-0.07	-1.09	-1.01	NA	-1.05	0.93	0.16	1.00	1.00	2	2_109	NA	109	NA	NA	NA	NA	NA
hsa-miR-208	-0.15	0.13	-0.33	-0.11	0.01	-0.11	-0.03	-0.04					-0.08	-1.08	-1.03	NA	-1.06	0.93	0.06	1.00	1.00	2	2_110	NA	110	NA	NA	NA	NA	NA
hsa-miR-329	0.15	-0.18	-0.13	-0.05	-0.05	-0.05	-0.04	-0.05					-0.05	-1.04	-1.03	NA	-1.03	0.93	0.27	1.00	1.00	2	2_111	NA	111	NA	NA	NA	NA	NA
hsa-miR-154*	-0.13	-0.11	-0.58	-0.27	0.07	-0.02	-0.04	0.00					-0.13	-1.21	1.00	NA	-1.10	0.94	0.06	1.00	1.00	2	2_112	NA	112	NA	NA	NA	NA	NA
hsa-miR-644	-0.08	-0.05	-0.16	-0.09	0.00	0.00	-0.07	-0.02					-0.06	-1.07	-1.02	NA	-1.04	0.95	0.24	1.00	1.00	2	2_113	NA	113	NA	NA	NA	NA	NA
hsa-miR-624	-0.15	-0.33	0.06	-0.14	0.02	-0.08	-0.03	-0.03					-0.08	-1.10	-1.02	NA	-1.06	0.95	0.11	1.00	1.00	2	2_114	NA	114	NA	NA	NA	NA	NA
hsa-miR-517c	-0.12	-0.39	-0.06	-0.19	0.01	-0.01	-0.04	-0.01					-0.10	-1.14	-1.01	NA	-1.07	0.97	0.08	1.00	1.00	2	2_115	NA	115	NA	NA	NA	NA	NA
hsa-miR-526b*	0.00	-0.53	-0.25	-0.26	0.08	-0.15	-0.03	-0.04					-0.15	-1.19	-1.02	NA	-1.11	0.97	0.01	1.00	1.00	2	2_116	NA	116	NA	NA	NA	NA	NA
hsa-miR-549	-0.31	-0.15	-0.11	-0.19	-0.09	-0.06	0.02	-0.04					-0.12	-1.14	-1.03	NA	-1.08	0.97	0.08	1.00	1.00	2	2_117	NA	117	NA	NA	NA	NA	NA
hsa-miR-146a	-0.08	-0.19	0.05	-0.07	-0.17	-0.11	-0.03	-0.10					-0.09	-1.05	-1.07	NA	-1.06	0.98	0.07	1.00	1.00	2	2_118	NA	118	NA	NA	NA	NA	NA
hsa-miR-519d	-0.14	0.02	-0.22	-0.11	-0.18	-0.04	-0.06	-0.09					-0.10	-1.08	-1.07	NA	-1.08	0.99	0.04	1.00	1.00	2	2_119	NA	119	NA	NA	NA	NA	NA
hsa-miR-373*	-0.12	-0.11	0.00	-0.07	-0.13	-0.12	-0.18	-0.14					-0.11	-1.05	-1.10	NA	-1.08	0.99	0.03	1.00	1.00	2	2_120	NA	120	NA	NA	NA	NA	NA
hsa-miR-518c*	0.05	-0.24	-0.16	-0.11	-0.17	-0.08	-0.07	-0.11					-0.11	-1.08	-1.08	NA	-1.08	1.00	0.04	1.00	1.00	2	2_121	NA	121	NA	NA	NA	NA	NA
hsa-miR-593	-0.04	0.31	0.48	0.25									0.25	1.19	NA	NA	1.19	0.01	0.98	1.00	1.00	3	3_1	NA	NA	1	NA	NA	NA	NA
hsa-miR-579	0.28	0.12	0.09	0.16									0.16	1.12	NA	NA	1.12	0.03	0.99	1.00	1.00	3	3_2	NA	NA	2	NA	NA	NA	NA
hsa-miR-589	0.13	-0.06	0.23	0.10					0.39	2.38	1.30	1.36	0.73	1.07	NA	2.56	1.66	0.04	0.39	1.00	1.00	3	3_3	NA	NA	3	NA	NA	NA	NA
hsa-miR-519a	0.22	0.04	0.09	0.12									0.12	1.08	NA	NA	1.08	0.09	0.99	1.00	1.00	3	3_4	NA	NA	4	NA	NA	NA	NA
hsa-miR-224	-0.04	-0.20	-0.01	-0.08					2.59	1.58	0.82	1.66	0.79	-1.06	NA	3.16	1.73	0.29	0.14	1.00	1.00	3	3_5	NA	NA	5	NA	NA	NA	NA
hsa-miR-202	0.00	0.15	-0.06	0.03									0.03	1.02	NA	NA	1.02	0.35	0.90	1.00	1.00	3	3_6	NA	NA	6	NA	NA	NA	NA
hsa-miR-26b	-0.03	-0.22	-0.10	-0.12					1.90	1.22	1.20	1.44	0.66	-1.09	NA	2.72	1.58	0.42	0.06	1.00	1.00	3	3_7	NA	NA	7	NA	NA	NA	NA
hsa-miR-34b	-0.01	0.02	0.06	0.03									0.03	1.02	NA	NA	1.02	0.48	0.94	1.00	1.00	3	3_8	NA	NA	8	NA	NA	NA	NA
hsa-miR-616	-0.19	-0.04	0.00	-0.08									-0.08	-1.05	NA	NA	-1.05	0.85	0.68	1.00	1.00	3	3_9	NA	NA	9	NA	NA	NA	NA
hsa-miR-527	-0.22	-0.01	-0.05	-0.09									-0.09	-1.07	NA	NA	-1.07	0.91	0.53	1.00	1.00	3	3_10	NA	NA	10	NA	NA	NA	NA
hsa-miR-522	-0.28	0.02	-0.29	-0.18									-0.18	-1.14	NA	NA	-1.14	0.96	0.07	1.00	1.00	3	3_11	NA	NA	11	NA	NA	NA	NA
hsa-miR-550	-0.19	-0.23	-0.02	-0.15									-0.15	-1.11	NA	NA	-1.11	0.96	0.15	1.00	1.00	3	3_12	NA	NA	12	NA	NA	NA	NA
hsa-miR-324-3p	-0.12	-0.11	-0.12	-0.11									-0.11	-1.08	NA	NA	-1.08	0.96	0.32	1.00	1.00	3	3_13	NA	NA	13	NA	NA	NA	NA
hsa-miR-150	-0.25	-0.08	-0.38	-0.24									-0.24	-1.18	NA	NA	-1.18	0.99	0.02	1.00	1.00	3	3_14	NA	NA	14	NA	NA	NA	NA

**ID**	log2ratio_Agilent_1	log2ratio_Agilent_2	log2ratio_Agilent_3	avg_log2ratio_Agilent	log2ratio_Exiqon_1	log2ratio_Exiqon_2	log2ratio_Exiqon_3	avg_log2ratio_Exiqon	log2ratio_Illumina_1	log2ratio_Illumina_2	log2ratio_Illumina_3	avg_log2ratio_Illumina	avg_log2ratio_all	avg_FoldChange_Agilent	avg_FoldChange_Exiqon	avg_FoldChange_Illumina	**avg_FoldChange_all**	p.value.up	p.value.down	**adjusted.p.value.up**	**adjusted.p.value.down**	Index	Index_Rank	RPrank.1	RPrank.2	RPrank.3	RPrank.4	RPrank.5	RPrank.6	RPrank.7
**hsa-miR-886-3p**					0.38	0.27	0.24	0.30	-0.01	-3.53	3.40	-0.05	0.13	NA	1.23	-1.03	**1.09**	0.00	0.61	**0.43**	**1.00**	4	4_1	NA	NA	NA	1	NA	NA	NA
**hsa-miR-582-3p**					0.20	0.10	0.17	0.16	4.16			4.16	1.16	NA	1.11	17.91	**2.23**	0.00	0.99	**0.69**	**1.00**	4	4_2	NA	NA	NA	2	NA	NA	NA
**hsa-miR-886-5p**					0.32	0.32	0.32	0.32	-1.09	-3.47	-0.94	-1.83	-0.76	NA	1.25	-3.56	**-1.69**	0.00	0.36	**1.00**	**1.00**	4	4_3	NA	NA	NA	3	NA	NA	NA
**hsa-miR-146b-5p**					0.21	0.15	0.14	0.16					0.16	NA	1.12	NA	**1.12**	0.01	1.00	**1.00**	**1.00**	4	4_4	NA	NA	NA	4	NA	NA	NA
**hsa-miR-582-5p**					0.27	0.14	0.13	0.18	1.12			1.12	0.42	NA	1.13	2.18	**1.33**	0.01	0.97	**1.00**	**1.00**	4	4_5	NA	NA	NA	5	NA	NA	NA
**hsa-miR-574-5p**					0.00	-0.06	0.03	-0.01	4.87	2.94	3.26	3.69	1.84	NA	-1.01	12.91	**3.58**	0.03	0.68	**1.00**	**1.00**	4	4_6	NA	NA	NA	6	NA	NA	NA
**hsa-miR-590-5p**					0.31	0.09	0.03	0.14					0.14	NA	1.10	NA	**1.10**	0.04	0.99	**1.00**	**1.00**	4	4_7	NA	NA	NA	7	NA	NA	NA
**hsa-miR-502-3p**					0.06	-0.01	-0.03	0.01	3.65	3.65	3.21	3.50	1.75	NA	1.01	11.33	**3.38**	0.04	0.68	**1.00**	**1.00**	4	4_8	NA	NA	NA	8	NA	NA	NA
**hsa-miR-615-3p**					0.20	0.21	0.10	0.17	0.08	-1.44		-0.68	-0.17	NA	1.12	-1.60	**-1.13**	0.05	0.81	**1.00**	**1.00**	4	4_9	NA	NA	NA	9	NA	NA	NA
**hsa-miR-362-5p**					0.29	0.10	0.03	0.14					0.14	NA	1.10	NA	**1.10**	0.05	0.99	**1.00**	**1.00**	4	4_10	NA	NA	NA	10	NA	NA	NA
**hsa-miR-342-3p**					0.22	0.09	0.16	0.16	-2.36	0.54	0.93	-0.30	-0.07	NA	1.11	-1.23	**-1.05**	0.07	0.52	**1.00**	**1.00**	4	4_11	NA	NA	NA	11	NA	NA	NA
**hsa-miR-887**					0.13	0.08	0.13	0.12					0.12	NA	1.08	NA	**1.08**	0.08	1.00	**1.00**	**1.00**	4	4_12	NA	NA	NA	12	NA	NA	NA
**hsa-miR-362-3p**					0.17	0.13	0.06	0.12					0.12	NA	1.09	NA	**1.09**	0.09	0.99	**1.00**	**1.00**	4	4_13	NA	NA	NA	13	NA	NA	NA
**hsa-miR-151-5p**					0.07	0.04	0.04	0.05	1.39	0.84	3.99	2.07	1.06	NA	1.04	4.21	**2.09**	0.10	0.76	**1.00**	**1.00**	4	4_14	NA	NA	NA	14	NA	NA	NA
**hsa-miR-125a-5p**					-0.01	0.00	-0.06	-0.02	3.45	2.05	3.46	2.98	1.48	NA	-1.02	7.92	**2.79**	0.10	0.59	**1.00**	**1.00**	4	4_15	NA	NA	NA	15	NA	NA	NA
**hsa-miR-455-3p**					0.28	0.08	0.09	0.15	-1.50	-0.19		-0.84	-0.25	NA	1.11	-1.79	**-1.19**	0.11	0.71	**1.00**	**1.00**	4	4_16	NA	NA	NA	16	NA	NA	NA
**hsa-miR-532-5p**					0.01	0.13	0.07	0.07	1.94	1.34	1.81	1.70	0.89	NA	1.05	3.25	**1.85**	0.11	0.73	**1.00**	**1.00**	4	4_17	NA	NA	NA	17	NA	NA	NA
**hsa-miR-28-5p**					0.08	0.05	-0.01	0.04	3.83	1.54		2.69	1.10	NA	1.03	6.44	**2.14**	0.11	0.84	**1.00**	**1.00**	4	4_18	NA	NA	NA	18	NA	NA	NA
**hsa-miR-374a**					0.09	-0.07	-0.02	0.00	2.74		4.56	3.65	1.46	NA	-1.00	12.54	**2.75**	0.12	0.62	**1.00**	**1.00**	4	4_19	NA	NA	NA	19	NA	NA	NA
**hsa-miR-590-3p**					0.24	0.04	0.08	0.12					0.12	NA	1.09	NA	**1.09**	0.13	0.99	**1.00**	**1.00**	4	4_20	NA	NA	NA	20	NA	NA	NA
**hsa-miR-760**					0.26	0.20	0.03	0.16	-2.00	-4.79	2.00	-1.60	-0.72	NA	1.12	-3.03	**-1.64**	0.13	0.19	**1.00**	**1.00**	4	4_21	NA	NA	NA	21	NA	NA	NA
**hsa-miR-151-3p**					0.11	0.00	0.09	0.07	1.88	1.18	2.33	1.80	0.93	NA	1.05	3.48	**1.91**	0.13	0.76	**1.00**	**1.00**	4	4_22	NA	NA	NA	22	NA	NA	NA
**hsa-miR-28-3p**					0.08	-0.02	0.08	0.05	2.62	0.53	2.99	2.05	1.05	NA	1.03	4.13	**2.07**	0.16	0.72	**1.00**	**1.00**	4	4_23	NA	NA	NA	23	NA	NA	NA
**hsa-miR-92a**					0.10	-0.01	0.06	0.05	0.73	3.15	-0.01	1.29	0.67	NA	1.03	2.44	**1.59**	0.16	0.57	**1.00**	**1.00**	4	4_24	NA	NA	NA	24	NA	NA	NA
**hsa-miR-122**					0.15	0.20	-0.01	0.11					0.11	NA	1.08	NA	**1.08**	0.16	0.97	**1.00**	**1.00**	4	4_25	NA	NA	NA	25	NA	NA	NA
**hsa-miR-876-3p**					0.29	0.00	0.06	0.11	0.89			0.89	0.31	NA	1.08	1.85	**1.24**	0.18	0.92	**1.00**	**1.00**	4	4_26	NA	NA	NA	26	NA	NA	NA
**hsa-miR-885-5p**					0.08	0.04	0.10	0.08					0.08	NA	1.05	NA	**1.05**	0.21	0.99	**1.00**	**1.00**	4	4_27	NA	NA	NA	27	NA	NA	NA
**hsa-miR-449a**					0.37	-0.02	-0.10	0.08					0.08	NA	1.06	NA	**1.06**	0.22	0.70	**1.00**	**1.00**	4	4_28	NA	NA	NA	28	NA	NA	NA
**hsa-miR-127-3p**					0.30	-0.09	0.05	0.09					0.09	NA	1.06	NA	**1.06**	0.25	0.89	**1.00**	**1.00**	4	4_29	NA	NA	NA	29	NA	NA	NA
**hsa-miR-934**					0.00	0.08	0.08	0.06					0.06	NA	1.04	NA	**1.04**	0.27	0.97	**1.00**	**1.00**	4	4_30	NA	NA	NA	30	NA	NA	NA
**hsa-miR-455-5p**					0.27	0.08	0.01	0.12	-1.16			-1.16	-0.20	NA	1.09	-2.24	**-1.15**	0.27	0.82	**1.00**	**1.00**	4	4_31	NA	NA	NA	31	NA	NA	NA
**hsa-miR-17**					0.00	-0.02	0.01	0.00	2.31	1.35	2.50	2.06	1.03	NA	-1.00	4.16	**2.04**	0.28	0.61	**1.00**	**1.00**	4	4_32	NA	NA	NA	32	NA	NA	NA
**hsa-miR-574-3p**					0.19	-0.04	0.05	0.07	1.14	-2.61	2.10	0.21	0.14	NA	1.05	1.16	**1.10**	0.31	0.52	**1.00**	**1.00**	4	4_33	NA	NA	NA	33	NA	NA	NA
**hsa-miR-374b**					0.06	0.00	0.00	0.02	2.51	1.39		1.95	0.79	NA	1.01	3.87	**1.73**	0.32	0.78	**1.00**	**1.00**	4	4_34	NA	NA	NA	34	NA	NA	NA
**hsa-miR-486-3p**					-0.03	0.04	0.11	0.04		-2.10		-2.10	-0.49	NA	1.03	-4.27	**-1.41**	0.34	0.69	**1.00**	**1.00**	4	4_35	NA	NA	NA	35	NA	NA	NA
**hsa-miR-331-3p**					0.06	0.08	0.05	0.06		-2.64	0.58	-1.03	-0.37	NA	1.05	-2.04	**-1.29**	0.35	0.50	**1.00**	**1.00**	4	4_36	NA	NA	NA	36	NA	NA	NA

ID	log2ratio_Agilent_1	log2ratio_Agilent_2	log2ratio_Agilent_3	avg_log2ratio_Agilent	log2ratio_Exiqon_1	log2ratio_Exiqon_2	log2ratio_Exiqon_3	avg_log2ratio_Exiqon	log2ratio_Illumina_1	log2ratio_Illumina_2	log2ratio_Illumina_3	avg_log2ratio_Illumina	avg_log2ratio_all	avg_FoldChange_Agilent	avg_FoldChange_Exiqon	avg_FoldChange_Illumina	avg_FoldChange_all	p.value.up	p.value.down	adjusted.p.value.up	adjusted.p.value.down	Index	Index_Rank	RPrank.1	RPrank.2	RPrank.3	RPrank.4	RPrank.5	RPrank.6	RPrank.7
hsa-miR-33a					0.19	0.05	0.01	0.08	-0.54	-0.06	-1.07	-0.56	-0.24	NA	1.06	-1.47	-1.18	0.36	0.37	1.00	1.00	4	4_37	NA	NA	NA	37	NA	NA	NA
hsa-miR-423-5p					-0.01	-0.01	-0.03	-0.02	0.79	2.69	-3.57	-0.03	-0.02	NA	-1.01	-1.02	-1.02	0.42	0.17	1.00	1.00	4	4_38	NA	NA	NA	38	NA	NA	NA
hsa-miR-219-5p					0.02	0.04	0.09	0.05	-0.68		-2.01	-1.35	-0.51	NA	1.03	-2.54	-1.42	0.42	0.28	1.00	1.00	4	4_39	NA	NA	NA	39	NA	NA	NA
hsa-miR-338-3p					0.11	0.06	0.03	0.07					0.07	NA	1.05	NA	1.05	0.43	0.98	1.00	1.00	4	4_40	NA	NA	NA	40	NA	NA	NA
hsa-miR-147b					-0.12	0.08	0.07	0.01					0.01	NA	1.01	NA	1.01	0.43	0.79	1.00	1.00	4	4_41	NA	NA	NA	41	NA	NA	NA
hsa-miR-876-5p					0.24	0.00	0.01	0.08					0.08	NA	1.06	NA	1.06	0.44	0.96	1.00	1.00	4	4_42	NA	NA	NA	42	NA	NA	NA
hsa-miR-744					0.14	0.00	0.01	0.05	-0.06	-2.94	1.60	-0.47	-0.21	NA	1.03	-1.38	-1.16	0.47	0.38	1.00	1.00	4	4_43	NA	NA	NA	43	NA	NA	NA
hsa-miR-330-3p					0.12	0.06	0.04	0.07	-3.34	-2.76	-1.31	-2.47	-1.20	NA	1.05	-5.53	-2.29	0.47	0.10	1.00	1.00	4	4_44	NA	NA	NA	44	NA	NA	NA
hsa-miR-193a-3p					0.08	-0.01	-0.13	-0.02	0.66	0.44	2.48	1.19	0.59	NA	-1.02	2.29	1.50	0.47	0.36	1.00	1.00	4	4_45	NA	NA	NA	45	NA	NA	NA
hsa-miR-361-5p					0.13	-0.10	0.07	0.03		-0.01		-0.01	0.02	NA	1.02	-1.01	1.02	0.48	0.68	1.00	1.00	4	4_46	NA	NA	NA	46	NA	NA	NA
hsa-miR-30a					0.04	-0.01	-0.15	-0.04	0.41	1.87	-0.03	0.75	0.36	NA	-1.03	1.68	1.28	0.49	0.24	1.00	1.00	4	4_47	NA	NA	NA	47	NA	NA	NA
hsa-miR-140-5p					0.11	-0.01	0.07	0.06					0.06	NA	1.04	NA	1.04	0.52	0.96	1.00	1.00	4	4_48	NA	NA	NA	48	NA	NA	NA
hsa-miR-30e					-0.03	-0.15	-0.01	-0.06	1.39	1.48	0.23	1.03	0.48	NA	-1.05	2.05	1.40	0.53	0.22	1.00	1.00	4	4_49	NA	NA	NA	49	NA	NA	NA
hsa-miR-339-5p					-0.01	0.03	-0.01	0.00	-1.37	0.45	1.19	0.09	0.05	NA	1.00	1.06	1.03	0.55	0.33	1.00	1.00	4	4_50	NA	NA	NA	50	NA	NA	NA
hsa-miR-499-5p					0.22	-0.07	-0.04	0.04	1.69			1.69	0.45	NA	1.03	3.22	1.37	0.56	0.65	1.00	1.00	4	4_51	NA	NA	NA	51	NA	NA	NA
hsa-miR-129-5p					-0.34	-0.14	-0.22	-0.24	2.60	-4.44	3.24	0.47	0.11	NA	-1.18	1.38	1.08	0.57	0.02	1.00	1.00	4	4_52	NA	NA	NA	52	NA	NA	NA
hsa-miR-193a-5p					-0.42	-0.19	-0.39	-0.33	3.32	-2.16	1.64	0.93	0.30	NA	-1.26	1.91	1.23	0.61	0.02	1.00	1.00	4	4_53	NA	NA	NA	53	NA	NA	NA
hsa-miR-125a-3p					-0.05	-0.07	-0.07	-0.06	0.45	2.36		1.40	0.52	NA	-1.05	2.65	1.44	0.61	0.30	1.00	1.00	4	4_54	NA	NA	NA	54	NA	NA	NA
hsa-miR-361-3p					0.03	0.05	0.02	0.03					0.03	NA	1.02	NA	1.02	0.63	0.94	1.00	1.00	4	4_55	NA	NA	NA	55	NA	NA	NA
hsa-miR-380					-0.04	0.06	0.04	0.02					0.02	NA	1.01	NA	1.01	0.65	0.86	1.00	1.00	4	4_56	NA	NA	NA	56	NA	NA	NA
hsa-miR-301a					0.18	-0.03	0.01	0.05					0.05	NA	1.04	NA	1.04	0.66	0.92	1.00	1.00	4	4_57	NA	NA	NA	57	NA	NA	NA
hsa-miR-216b					0.13	-0.07	-0.06	0.00	2.08			2.08	0.52	NA	1.00	4.23	1.43	0.66	0.63	1.00	1.00	4	4_58	NA	NA	NA	58	NA	NA	NA
hsa-miR-425					0.06	-0.12	-0.06	-0.04	0.54	-1.14	1.60	0.33	0.15	NA	-1.03	1.26	1.11	0.66	0.23	1.00	1.00	4	4_59	NA	NA	NA	59	NA	NA	NA
hsa-miR-548d-3p					0.06	-0.09	0.05	0.01		-0.09		-0.09	-0.02	NA	1.01	-1.06	-1.01	0.67	0.60	1.00	1.00	4	4_60	NA	NA	NA	60	NA	NA	NA
hsa-miR-450a					-0.04	-0.09	0.10	-0.01					-0.01	NA	-1.01	NA	-1.01	0.67	0.63	1.00	1.00	4	4_61	NA	NA	NA	61	NA	NA	NA
hsa-miR-330-5p					0.13	0.03	-0.04	0.04					0.04	NA	1.03	NA	1.03	0.67	0.90	1.00	1.00	4	4_62	NA	NA	NA	62	NA	NA	NA
hsa-miR-486-5p					-0.09	-0.01	0.02	-0.03	1.12	-2.04		-0.46	-0.20	NA	-1.02	-1.38	-1.15	0.68	0.42	1.00	1.00	4	4_63	NA	NA	NA	63	NA	NA	NA
hsa-miR-338-5p					0.27	-0.07	-0.10	0.03					0.03	NA	1.02	NA	1.02	0.68	0.54	1.00	1.00	4	4_64	NA	NA	NA	64	NA	NA	NA
hsa-miR-331-5p					0.02	0.02	0.03	0.02					0.02	NA	1.02	NA	1.02	0.70	0.93	1.00	1.00	4	4_65	NA	NA	NA	65	NA	NA	NA
hsa-miR-423-3p					-0.08	0.01	-0.01	-0.03	-1.56	-3.92	1.50	-1.33	-0.68	NA	-1.02	-2.51	-1.60	0.70	0.09	1.00	1.00	4	4_66	NA	NA	NA	66	NA	NA	NA
hsa-miR-454					-0.11	-0.01	0.08	-0.01					-0.01	NA	-1.01	NA	-1.01	0.70	0.71	1.00	1.00	4	4_67	NA	NA	NA	67	NA	NA	NA
hsa-miR-34c-5p					0.18	-0.12	0.01	0.02	-0.47			-0.47	-0.10	NA	1.02	-1.38	-1.07	0.71	0.50	1.00	1.00	4	4_68	NA	NA	NA	68	NA	NA	NA
hsa-miR-576-3p					-0.10	-0.03	-0.07	-0.07	2.82			2.82	0.65	NA	-1.05	7.05	1.57	0.72	0.36	1.00	1.00	4	4_69	NA	NA	NA	69	NA	NA	NA
hsa-miR-524-5p					-0.02	0.00	0.05	0.01					0.01	NA	1.01	NA	1.01	0.72	0.88	1.00	1.00	4	4_70	NA	NA	NA	70	NA	NA	NA
hsa-miR-501-3p					-0.29	-0.12	-0.25	-0.22	2.02	0.97		1.49	0.47	NA	-1.16	2.82	1.38	0.74	0.07	1.00	1.00	4	4_71	NA	NA	NA	71	NA	NA	NA
hsa-miR-519c-3p					0.15	-0.06	0.00	0.03					0.03	NA	1.02	NA	1.02	0.78	0.84	1.00	1.00	4	4_72	NA	NA	NA	72	NA	NA	NA

ID	log2ratio_Agilent_1	log2ratio_Agilent_2	log2ratio_Agilent_3	avg_log2ratio_Agilent	log2ratio_Exiqon_1	log2ratio_Exiqon_2	log2ratio_Exiqon_3	avg_log2ratio_Exiqon	log2ratio_Illumina_1	log2ratio_Illumina_2	log2ratio_Illumina_3	avg_log2ratio_Illumina	avg_log2ratio_all	avg_FoldChange_Agilent	avg_FoldChange_Exiqon	avg_FoldChange_Illumina	avg_FoldChange_all	p.value.up	p.value.down	adjusted.p.value.up	adjusted.p.value.down	Index	Index_Rank	RPrank.1	RPrank.2	RPrank.3	RPrank.4	RPrank.5	RPrank.6	RPrank.7
hsa-miR-339-3p					-0.08	-0.03	-0.03	-0.04	-4.92	-1.70	-0.25	-2.29	-1.17	NA	-1.03	-4.89	-2.24	0.79	0.03	1.00	1.00	4	4_73	NA	NA	NA	73	NA	NA	NA
hsa-miR-139-5p					0.06	0.03	-0.05	0.01					0.01	NA	1.01	NA	1.01	0.82	0.82	1.00	1.00	4	4_74	NA	NA	NA	74	NA	NA	NA
hsa-miR-301b					-0.07	-0.01	0.05	-0.01					-0.01	NA	-1.01	NA	-1.01	0.84	0.75	1.00	1.00	4	4_75	NA	NA	NA	75	NA	NA	NA
hsa-miR-875-3p					-0.02	0.01	0.00	0.00					0.00	NA	-1.00	NA	-1.00	0.86	0.81	1.00	1.00	4	4_76	NA	NA	NA	76	NA	NA	NA
hsa-miR-491-5p					0.03	-0.04	0.01	0.00					0.00	NA	1.00	NA	1.00	0.88	0.82	1.00	1.00	4	4_77	NA	NA	NA	77	NA	NA	NA
hsa-miR-376c					-0.04	0.02	-0.02	-0.01					-0.01	NA	-1.01	NA	-1.01	0.89	0.72	1.00	1.00	4	4_78	NA	NA	NA	78	NA	NA	NA
hsa-miR-188-5p					0.07	-0.05	0.00	0.01					0.01	NA	1.00	NA	1.00	0.90	0.78	1.00	1.00	4	4_79	NA	NA	NA	79	NA	NA	NA
hsa-miR-501-5p					0.06	-0.01	-0.05	0.00					0.00	NA	-1.00	NA	-1.00	0.90	0.75	1.00	1.00	4	4_80	NA	NA	NA	80	NA	NA	NA
hsa-miR-888					0.14	-0.14	-0.09	-0.03					-0.03	NA	-1.02	NA	-1.02	0.92	0.33	1.00	1.00	4	4_81	NA	NA	NA	81	NA	NA	NA
hsa-miR-548c-3p					0.11	-0.07	-0.07	-0.01					-0.01	NA	-1.01	NA	-1.01	0.93	0.54	1.00	1.00	4	4_82	NA	NA	NA	82	NA	NA	NA
hsa-miR-190b					-0.01	-0.03	0.00	-0.01					-0.01	NA	-1.01	NA	-1.01	0.94	0.71	1.00	1.00	4	4_83	NA	NA	NA	83	NA	NA	NA
hsa-miR-296-5p					-0.25	-0.19	-0.38	-0.28	-1.28	-1.97		-1.62	-0.81	NA	-1.21	-3.08	-1.76	0.94	0.01	1.00	1.00	4	4_84	NA	NA	NA	84	NA	NA	NA
hsa-miR-519b-3p					-0.06	-0.02	0.00	-0.03					-0.03	NA	-1.02	NA	-1.02	0.95	0.62	1.00	1.00	4	4_85	NA	NA	NA	85	NA	NA	NA
hsa-miR-518a-3p					-0.04	-0.04	0.01	-0.02					-0.02	NA	-1.02	NA	-1.02	0.95	0.63	1.00	1.00	4	4_86	NA	NA	NA	86	NA	NA	NA
hsa-miR-371-3p					-0.02	-0.01	-0.02	-0.02					-0.02	NA	-1.01	NA	-1.01	0.95	0.65	1.00	1.00	4	4_87	NA	NA	NA	87	NA	NA	NA
hsa-miR-548a-3p					0.04	-0.06	-0.03	-0.02					-0.02	NA	-1.01	NA	-1.01	0.95	0.65	1.00	1.00	4	4_88	NA	NA	NA	88	NA	NA	NA
hsa-miR-548b-3p					0.01	-0.07	-0.02	-0.03					-0.03	NA	-1.02	NA	-1.02	0.96	0.60	1.00	1.00	4	4_89	NA	NA	NA	89	NA	NA	NA
hsa-miR-628-5p					0.02	-0.05	-0.06	-0.03					-0.03	NA	-1.02	NA	-1.02	0.96	0.55	1.00	1.00	4	4_90	NA	NA	NA	90	NA	NA	NA
hsa-miR-890					0.00	-0.02	-0.09	-0.04					-0.04	NA	-1.03	NA	-1.03	0.97	0.47	1.00	1.00	4	4_91	NA	NA	NA	91	NA	NA	NA
hsa-miR-628-3p					-0.16	0.00	-0.08	-0.08					-0.08	NA	-1.06	NA	-1.06	0.97	0.28	1.00	1.00	4	4_92	NA	NA	NA	92	NA	NA	NA
hsa-miR-124					-0.08	-0.05	-0.11	-0.08	-2.84			-2.84	-0.77	NA	-1.06	-7.16	-1.71	0.97	0.05	1.00	1.00	4	4_93	NA	NA	NA	93	NA	NA	NA
hsa-miR-671-5p					-0.50	-0.27	-0.52	-0.43	-2.68	-3.76		-3.22	-1.54	NA	-1.35	-9.32	-2.92	0.97	0.00	1.00	0.09	4	4_94	NA	NA	NA	94	NA	NA	NA
hsa-miR-543					-0.02	-0.12	-0.04	-0.06					-0.06	NA	-1.04	NA	-1.04	0.98	0.31	1.00	1.00	4	4_95	NA	NA	NA	95	NA	NA	NA
hsa-miR-940					-0.88	-0.68	-0.76	-0.77		-2.87		-2.87	-1.30	NA	-1.71	-7.33	-2.46	0.99	0.00	1.00	0.00	4	4_96	NA	NA	NA	96	NA	NA	NA
hsa-miR-490-3p					-0.01	-0.11	-0.08	-0.07					-0.07	NA	-1.05	NA	-1.05	0.99	0.26	1.00	1.00	4	4_97	NA	NA	NA	97	NA	NA	NA
hsa-miR-665					-0.21	-0.20	-0.29	-0.23	-3.21			-3.21	-0.98	NA	-1.17	-9.22	-1.97	0.99	0.00	1.00	1.00	4	4_98	NA	NA	NA	98	NA	NA	NA
hsa-miR-509-3p					-0.08	-0.13	-0.05	-0.09					-0.09	NA	-1.06	NA	-1.06	0.99	0.16	1.00	1.00	4	4_99	NA	NA	NA	99	NA	NA	NA
hsa-miR-891b					-0.22	-0.22	-0.02	-0.15					-0.15	NA	-1.11	NA	-1.11	1.00	0.04	1.00	1.00	4	4_100	NA	NA	NA	100	NA	NA	NA
hsa-miR-483-3p					-0.22	-0.14	-0.15	-0.17					-0.17	NA	-1.13	NA	-1.13	1.00	0.02	1.00	1.00	4	4_101	NA	NA	NA	101	NA	NA	NA
hsa-miR-483-5p					-0.27	-0.23	-0.11	-0.20					-0.20	NA	-1.15	NA	-1.15	1.00	0.01	1.00	1.00	4	4_102	NA	NA	NA	102	NA	NA	NA
hsa-miR-654-5p					-0.13	-0.26	-0.25	-0.21					-0.21	NA	-1.16	NA	-1.16	1.00	0.00	1.00	1.00	4	4_103	NA	NA	NA	103	NA	NA	NA
hsa-miR-371-5p					-0.66	-0.40	-0.49	-0.52					-0.52	NA	-1.43	NA	-1.43	1.00	0.00	1.00	0.04	4	4_104	NA	NA	NA	104	NA	NA	NA
dmr_3	0.61	0.96	-0.21	0.45									0.45	1.37	NA	NA	1.37	0.00	0.64	0.25	1.00	5	5_1	NA	NA	NA	NA	1	NA	NA
hsv1-miR-H1	0.34	0.33	0.54	0.40									0.40	1.32	NA	NA	1.32	0.00	1.00	0.36	1.00	5	5_2	NA	NA	NA	NA	2	NA	NA
SCorner3	0.29	0.12	0.77	0.39									0.39	1.31	NA	NA	1.31	0.00	0.99	1.00	1.00	5	5_3	NA	NA	NA	NA	3	NA	NA
hsa-miR-576	0.28	0.34	0.29	0.30									0.30	1.23	NA	NA	1.23	0.01	1.00	1.00	1.00	5	5_4	NA	NA	NA	NA	4	NA	NA

ID	log2ratio_Agilent_1	log2ratio_Agilent_2	log2ratio_Agilent_3	avg_log2ratio_Agilent	log2ratio_Exiqon_1	log2ratio_Exiqon_2	log2ratio_Exiqon_3	avg_log2ratio_Exiqon	log2ratio_Illumina_1	log2ratio_Illumina_2	log2ratio_Illumina_3	avg_log2ratio_Illumina	avg_log2ratio_all	avg_FoldChange_Agilent	avg_FoldChange_Exiqon	avg_FoldChange_Illumina	avg_FoldChange_all	p.value.up	p.value.down	adjusted.p.value.up	adjusted.p.value.down	Index	Index_Rank	RPrank.1	RPrank.2	RPrank.3	RPrank.4	RPrank.5	RPrank.6	RPrank.7
ebv-miR-BART17-3p	0.23	0.95	0.07	0.42									0.42	1.34	NA	NA	1.34	0.01	0.98	1.00	1.00	5	5_5	NA	NA	NA	NA	5	NA	NA
hsa-miR-654	0.13	0.65	0.29	0.36									0.36	1.28	NA	NA	1.28	0.01	0.99	1.00	1.00	5	5_6	NA	NA	NA	NA	6	NA	NA
hcmv-miR-UL22A*	0.16	-0.28	1.18	0.35									0.35	1.28	NA	NA	1.28	0.02	0.49	1.00	1.00	5	5_7	NA	NA	NA	NA	7	NA	NA
hcmv-miR-UL70-3p	0.33	-0.01	0.37	0.23									0.23	1.17	NA	NA	1.17	0.02	0.95	1.00	1.00	5	5_8	NA	NA	NA	NA	8	NA	NA
hsa-miR-548d	0.21	-0.04	0.44	0.21									0.21	1.15	NA	NA	1.15	0.05	0.91	1.00	1.00	5	5_9	NA	NA	NA	NA	9	NA	NA
hsa-miR-517a	0.14	0.15	0.36	0.22									0.22	1.16	NA	NA	1.16	0.05	0.99	1.00	1.00	5	5_10	NA	NA	NA	NA	10	NA	NA
hsa-miR-454-5p	0.21	-0.12	0.46	0.18									0.18	1.13	NA	NA	1.13	0.05	0.81	1.00	1.00	5	5_11	NA	NA	NA	NA	11	NA	NA
hsa-miR-199a	0.07	0.53	0.14	0.25									0.25	1.19	NA	NA	1.19	0.06	0.97	1.00	1.00	5	5_12	NA	NA	NA	NA	12	NA	NA
hsa-miR-146b	0.19	0.62	-0.30	0.17									0.17	1.13	NA	NA	1.13	0.06	0.45	1.00	1.00	5	5_13	NA	NA	NA	NA	13	NA	NA
hsa-miR-526a	0.07	0.45	0.15	0.22									0.22	1.17	NA	NA	1.17	0.06	0.97	1.00	1.00	5	5_14	NA	NA	NA	NA	14	NA	NA
hsa-miR-376a*	0.58	-0.09	0.00	0.17									0.17	1.12	NA	NA	1.12	0.07	0.72	1.00	1.00	5	5_15	NA	NA	NA	NA	15	NA	NA
kshv-miR-K12-3*	0.12	0.40	0.12	0.21									0.21	1.16	NA	NA	1.16	0.07	0.97	1.00	1.00	5	5_16	NA	NA	NA	NA	16	NA	NA
hsa-miR-548a	0.04	0.13	0.46	0.21									0.21	1.16	NA	NA	1.16	0.08	0.95	1.00	1.00	5	5_17	NA	NA	NA	NA	17	NA	NA
ebv-miR-BART1-3p	0.19	0.46	-0.18	0.16									0.16	1.11	NA	NA	1.11	0.09	0.65	1.00	1.00	5	5_18	NA	NA	NA	NA	18	NA	NA
hsa-miR-425-3p	0.05	0.89	-0.32	0.20									0.20	1.15	NA	NA	1.15	0.10	0.35	1.00	1.00	5	5_19	NA	NA	NA	NA	19	NA	NA
ebv-miR-BART11-3p	0.17	0.15	0.17	0.16									0.16	1.12	NA	NA	1.12	0.11	0.98	1.00	1.00	5	5_20	NA	NA	NA	NA	20	NA	NA
kshv-miR-K12-4-5p	0.54	0.00	-0.17	0.12									0.12	1.09	NA	NA	1.09	0.11	0.56	1.00	1.00	5	5_21	NA	NA	NA	NA	21	NA	NA
ebv-miR-BART20-3p	-0.08	0.14	0.37	0.14									0.14	1.11	NA	NA	1.11	0.13	0.83	1.00	1.00	5	5_22	NA	NA	NA	NA	22	NA	NA
miRNABrightCorner30	0.10	0.55	-0.60	0.02									0.02	1.01	NA	NA	1.01	0.13	0.08	1.00	1.00	5	5_23	NA	NA	NA	NA	23	NA	NA
ebv-miR-BART1-5p	0.13	0.29	0.07	0.16									0.16	1.12	NA	NA	1.12	0.13	0.95	1.00	1.00	5	5_24	NA	NA	NA	NA	24	NA	NA
ebv-miR-BART17-5p	0.16	0.15	0.15	0.15									0.15	1.11	NA	NA	1.11	0.13	0.97	1.00	1.00	5	5_25	NA	NA	NA	NA	25	NA	NA
hsa-miR-493-5p	0.47	-0.30	0.03	0.07									0.07	1.05	NA	NA	1.05	0.14	0.37	1.00	1.00	5	5_26	NA	NA	NA	NA	26	NA	NA
hcmv-miR-US25-1	-0.18	-0.08	0.71	0.15									0.15	1.11	NA	NA	1.11	0.14	0.40	1.00	1.00	5	5_27	NA	NA	NA	NA	27	NA	NA
hcmv-miR-UL148D	0.34	0.04	-0.14	0.08									0.08	1.06	NA	NA	1.06	0.15	0.65	1.00	1.00	5	5_28	NA	NA	NA	NA	28	NA	NA
hsa-miR-556	0.21	0.23	-0.14	0.10									0.10	1.07	NA	NA	1.07	0.16	0.72	1.00	1.00	5	5_29	NA	NA	NA	NA	29	NA	NA
ebv-miR-BHRF1-2	0.18	0.28	-0.14	0.11									0.11	1.08	NA	NA	1.08	0.17	0.71	1.00	1.00	5	5_30	NA	NA	NA	NA	30	NA	NA
kshv-miR-K12-4-3p	0.22	0.17	-0.14	0.08									0.08	1.06	NA	NA	1.06	0.17	0.70	1.00	1.00	5	5_31	NA	NA	NA	NA	31	NA	NA
kshv-miR-K12-5	0.00	-0.31	0.47	0.06									0.06	1.04	NA	NA	1.04	0.17	0.34	1.00	1.00	5	5_32	NA	NA	NA	NA	32	NA	NA
dmr_308	-0.05	0.26	0.18	0.13									0.13	1.09	NA	NA	1.09	0.17	0.87	1.00	1.00	5	5_33	NA	NA	NA	NA	33	NA	NA
dmr_316	0.02	0.32	0.08	0.14									0.14	1.10	NA	NA	1.10	0.18	0.91	1.00	1.00	5	5_34	NA	NA	NA	NA	34	NA	NA
hcmv-miR-US25-2-5p	0.17	0.17	0.01	0.12									0.12	1.09	NA	NA	1.09	0.18	0.92	1.00	1.00	5	5_35	NA	NA	NA	NA	35	NA	NA
hsa-miR-548b	-0.34	0.19	0.29	0.05									0.05	1.03	NA	NA	1.03	0.18	0.27	1.00	1.00	5	5_36	NA	NA	NA	NA	36	NA	NA
hsa-miR-219	0.05	-0.01	0.34	0.12									0.12	1.09	NA	NA	1.09	0.19	0.86	1.00	1.00	5	5_37	NA	NA	NA	NA	37	NA	NA
ebv-miR-BART4	0.22	0.06	0.01	0.09									0.09	1.07	NA	NA	1.07	0.21	0.87	1.00	1.00	5	5_38	NA	NA	NA	NA	38	NA	NA
hsa-miR-519c	0.03	0.33	-0.01	0.12									0.12	1.08	NA	NA	1.08	0.24	0.81	1.00	1.00	5	5_39	NA	NA	NA	NA	39	NA	NA
ebv-miR-BART14-5p	0.12	0.11	0.12	0.12									0.12	1.09	NA	NA	1.09	0.24	0.93	1.00	1.00	5	5_40	NA	NA	NA	NA	40	NA	NA

ID	log2ratio_Agilent_1	log2ratio_Agilent_2	log2ratio_Agilent_3	avg_log2ratio_Agilent	log2ratio_Exiqon_1	log2ratio_Exiqon_2	log2ratio_Exiqon_3	avg_log2ratio_Exiqon	log2ratio_Illumina_1	log2ratio_Illumina_2	log2ratio_Illumina_3	avg_log2ratio_Illumina	avg_log2ratio_all	avg_FoldChange_Agilent	avg_FoldChange_Exiqon	avg_FoldChange_Illumina	avg_FoldChange_all	p.value.up	p.value.down	adjusted.p.value.up	adjusted.p.value.down	Index	Index_Rank	RPrank.1	RPrank.2	RPrank.3	RPrank.4	RPrank.5	RPrank.6	RPrank.7
hsa-miR-513	0.09	0.22	-0.01	0.10									0.10	1.07	NA	NA	1.07	0.25	0.87	1.00	1.00	5	5_41	NA	NA	NA	NA	41	NA	NA
mr_1	0.05	0.16	0.10	0.10									0.10	1.07	NA	NA	1.07	0.25	0.92	1.00	1.00	5	5_42	NA	NA	NA	NA	42	NA	NA
ebv-miR-BART5	0.07	0.25	-0.04	0.09									0.09	1.07	NA	NA	1.07	0.26	0.82	1.00	1.00	5	5_43	NA	NA	NA	NA	43	NA	NA
NC2_00122731	0.05	0.14	0.12	0.10									0.10	1.07	NA	NA	1.07	0.26	0.91	1.00	1.00	5	5_44	NA	NA	NA	NA	44	NA	NA
hsa-miR-499	-0.10	0.11	0.25	0.09									0.09	1.06	NA	NA	1.06	0.27	0.74	1.00	1.00	5	5_45	NA	NA	NA	NA	45	NA	NA
ebv-miR-BART8-3p	-0.17	0.12	0.28	0.08									0.08	1.06	NA	NA	1.06	0.27	0.59	1.00	1.00	5	5_46	NA	NA	NA	NA	46	NA	NA
DarkCorner	0.17	0.13	-0.03	0.09									0.09	1.06	NA	NA	1.06	0.28	0.83	1.00	1.00	5	5_47	NA	NA	NA	NA	47	NA	NA
hsa-miR-520a*	0.00	0.25	0.04	0.10									0.10	1.07	NA	NA	1.07	0.28	0.83	1.00	1.00	5	5_48	NA	NA	NA	NA	48	NA	NA
ebv-miR-BART3-3p	-0.06	0.31	0.04	0.10									0.10	1.07	NA	NA	1.07	0.28	0.76	1.00	1.00	5	5_49	NA	NA	NA	NA	49	NA	NA
hcmv-miR-UL70-5p	0.21	0.04	-0.10	0.05									0.05	1.04	NA	NA	1.04	0.29	0.70	1.00	1.00	5	5_50	NA	NA	NA	NA	50	NA	NA
hsa-miR-520d	0.13	0.09	0.05	0.09									0.09	1.07	NA	NA	1.07	0.30	0.90	1.00	1.00	5	5_51	NA	NA	NA	NA	51	NA	NA
hsa-miR-653	0.31	-0.14	-0.22	-0.01									-0.01	-1.01	NA	NA	-1.01	0.30	0.26	1.00	1.00	5	5_52	NA	NA	NA	NA	52	NA	NA
kshv-miR-K12-10a	-0.41	0.02	0.35	-0.01									-0.01	-1.01	NA	NA	-1.01	0.31	0.10	1.00	1.00	5	5_53	NA	NA	NA	NA	53	NA	NA
hsa-miR-423	0.16	-0.27	0.17	0.02									0.02	1.01	NA	NA	1.01	0.32	0.45	1.00	1.00	5	5_54	NA	NA	NA	NA	54	NA	NA
hsa-miR-520d*	-0.28	0.45	-0.05	0.04									0.04	1.03	NA	NA	1.03	0.33	0.21	1.00	1.00	5	5_55	NA	NA	NA	NA	55	NA	NA
hcmv-miR-US33	-0.13	0.15	0.15	0.06									0.06	1.04	NA	NA	1.04	0.33	0.69	1.00	1.00	5	5_56	NA	NA	NA	NA	56	NA	NA
ebv-miR-BART7	-0.04	-0.24	0.36	0.02									0.02	1.02	NA	NA	1.02	0.33	0.40	1.00	1.00	5	5_57	NA	NA	NA	NA	57	NA	NA
hsa-miR-525	0.07	-0.42	0.24	-0.04									-0.04	-1.03	NA	NA	-1.03	0.33	0.24	1.00	1.00	5	5_58	NA	NA	NA	NA	58	NA	NA
ebv-miR-BART6-3p	0.15	-0.02	0.10	0.08									0.08	1.05	NA	NA	1.05	0.33	0.83	1.00	1.00	5	5_59	NA	NA	NA	NA	59	NA	NA
hsa-miR-342	0.00	0.18	0.04	0.07									0.07	1.05	NA	NA	1.05	0.33	0.84	1.00	1.00	5	5_60	NA	NA	NA	NA	60	NA	NA
hsa-miR-524*	0.27	-0.25	-0.06	-0.01									-0.01	-1.01	NA	NA	-1.01	0.34	0.34	1.00	1.00	5	5_61	NA	NA	NA	NA	61	NA	NA
kshv-miR-K12-7	0.04	-0.11	0.22	0.05									0.05	1.04	NA	NA	1.04	0.34	0.70	1.00	1.00	5	5_62	NA	NA	NA	NA	62	NA	NA
hsa-miR-331	0.08	-0.13	0.19	0.05									0.05	1.03	NA	NA	1.03	0.34	0.69	1.00	1.00	5	5_63	NA	NA	NA	NA	63	NA	NA
hsa-miR-493-3p	0.15	0.14	-0.20	0.03									0.03	1.02	NA	NA	1.02	0.35	0.56	1.00	1.00	5	5_64	NA	NA	NA	NA	64	NA	NA
hsa-miR-452*	-0.17	0.11	0.19	0.04									0.04	1.03	NA	NA	1.03	0.35	0.55	1.00	1.00	5	5_65	NA	NA	NA	NA	65	NA	NA
hcmv-miR-UL112	0.24	-0.04	-0.26	-0.02									-0.02	-1.01	NA	NA	-1.01	0.35	0.33	1.00	1.00	5	5_66	NA	NA	NA	NA	66	NA	NA
kshv-miR-K12-6-5p	-0.08	0.40	-0.23	0.03									0.03	1.02	NA	NA	1.02	0.36	0.32	1.00	1.00	5	5_67	NA	NA	NA	NA	67	NA	NA
hsa-miR-339	0.05	0.15	0.00	0.07									0.07	1.05	NA	NA	1.05	0.36	0.84	1.00	1.00	5	5_68	NA	NA	NA	NA	68	NA	NA
hsa-miR-486	0.05	-0.19	0.19	0.01									0.01	1.01	NA	NA	1.01	0.38	0.59	1.00	1.00	5	5_69	NA	NA	NA	NA	69	NA	NA
ebv-miR-BART6-5p	0.20	0.00	-0.16	0.01									0.01	1.01	NA	NA	1.01	0.38	0.56	1.00	1.00	5	5_70	NA	NA	NA	NA	70	NA	NA
dmr_31a	-0.06	-0.08	0.26	0.04									0.04	1.03	NA	NA	1.03	0.39	0.62	1.00	1.00	5	5_71	NA	NA	NA	NA	71	NA	NA
hsa-miR-518f	-0.06	0.24	-0.01	0.06									0.06	1.04	NA	NA	1.04	0.39	0.69	1.00	1.00	5	5_72	NA	NA	NA	NA	72	NA	NA
hsa-miR-548c	0.25	-0.45	-0.11	-0.10									-0.10	-1.07	NA	NA	-1.07	0.40	0.09	1.00	1.00	5	5_73	NA	NA	NA	NA	73	NA	NA
hsa-miR-17-3p	-0.01	0.08	0.13	0.07									0.07	1.05	NA	NA	1.05	0.40	0.82	1.00	1.00	5	5_74	NA	NA	NA	NA	74	NA	NA
hsa-miR-491	-0.15	0.19	0.06	0.03									0.03	1.02	NA	NA	1.02	0.40	0.59	1.00	1.00	5	5_75	NA	NA	NA	NA	75	NA	NA
hsa-miR-525*	0.21	-0.04	-0.16	0.00									0.00	1.00	NA	NA	1.00	0.41	0.48	1.00	1.00	5	5_76	NA	NA	NA	NA	76	NA	NA

**ID**	log2ratio_Agilent_1	log2ratio_Agilent_2	log2ratio_Agilent_3	avg_log2ratio_Agilent	log2ratio_Exiqon_1	log2ratio_Exiqon_2	log2ratio_Exiqon_3	avg_log2ratio_Exiqon	log2ratio_Illumina_1	log2ratio_Illumina_2	log2ratio_Illumina_3	avg_log2ratio_Illumina	avg_log2ratio_all	avg_FoldChange_Agilent	avg_FoldChange_Exiqon	avg_FoldChange_Illumina	**avg_FoldChange_all**	p.value.up	p.value.down	**adjusted.p.value.up**	**adjusted.p.value.down**	Index	Index_Rank	RPrank.1	RPrank.2	RPrank.3	RPrank.4	RPrank.5	RPrank.6	RPrank.7
**hsa-miR-509**	-0.02	-0.08	0.20	0.03									0.03	1.02	NA	NA	**1.02**	0.41	0.67	**1.00**	**1.00**	5	5_77	NA	NA	NA	NA	77	NA	NA
**hsa-miR-323**	-0.21	0.04	0.21	0.01									0.01	1.01	NA	NA	**1.01**	0.41	0.37	**1.00**	**1.00**	5	5_78	NA	NA	NA	NA	78	NA	NA
**hsa-miR-202***	-0.01	-0.07	0.19	0.04									0.04	1.02	NA	NA	**1.02**	0.42	0.69	**1.00**	**1.00**	5	5_79	NA	NA	NA	NA	79	NA	NA
**hsa-miR-671**	0.09	0.09	-0.01	0.06									0.06	1.04	NA	NA	**1.04**	0.42	0.80	**1.00**	**1.00**	5	5_80	NA	NA	NA	NA	80	NA	NA
**ebv-miR-BART14-3p**	0.06	0.18	-0.55	-0.11									-0.11	-1.08	NA	NA	**-1.08**	0.42	0.17	**1.00**	**1.00**	5	5_81	NA	NA	NA	NA	81	NA	NA
**hsa-miR-188**	0.01	0.03	0.12	0.05									0.05	1.04	NA	NA	**1.04**	0.43	0.81	**1.00**	**1.00**	5	5_82	NA	NA	NA	NA	82	NA	NA
**NC2_00092197**	0.04	0.13	0.00	0.06									0.06	1.04	NA	NA	**1.04**	0.43	0.79	**1.00**	**1.00**	5	5_83	NA	NA	NA	NA	83	NA	NA
**hsa-miR-122a**	-0.01	0.21	-0.08	0.04									0.04	1.03	NA	NA	**1.03**	0.44	0.64	**1.00**	**1.00**	5	5_84	NA	NA	NA	NA	84	NA	NA
**hsa-miR-124a**	-0.25	0.31	-0.05	0.00									0.00	1.00	NA	NA	**1.00**	0.44	0.23	**1.00**	**1.00**	5	5_85	NA	NA	NA	NA	85	NA	NA
**hsa-miR-508**	-0.34	0.12	0.16	-0.02									-0.02	-1.01	NA	NA	**-1.01**	0.44	0.20	**1.00**	**1.00**	5	5_86	NA	NA	NA	NA	86	NA	NA
**kshv-miR-K12-9**	-0.16	-0.03	0.22	0.01									0.01	1.01	NA	NA	**1.01**	0.45	0.47	**1.00**	**1.00**	5	5_87	NA	NA	NA	NA	87	NA	NA
**hsa-miR-330**	-0.02	-0.04	0.19	0.04									0.04	1.03	NA	NA	**1.03**	0.45	0.70	**1.00**	**1.00**	5	5_88	NA	NA	NA	NA	88	NA	NA
**hsa-miR-380-3p**	-0.07	0.14	0.05	0.04									0.04	1.03	NA	NA	**1.03**	0.46	0.72	**1.00**	**1.00**	5	5_89	NA	NA	NA	NA	89	NA	NA
**NegativeControl**	0.10	0.00	0.00	0.03									0.03	1.02	NA	NA	**1.02**	0.47	0.76	**1.00**	**1.00**	5	5_90	NA	NA	NA	NA	90	NA	NA
**hsa-miR-520f**	0.03	0.00	0.12	0.05									0.05	1.03	NA	NA	**1.03**	0.48	0.79	**1.00**	**1.00**	5	5_91	NA	NA	NA	NA	91	NA	NA
**NC2_00079215**	0.04	0.13	-0.04	0.04									0.04	1.03	NA	NA	**1.03**	0.48	0.73	**1.00**	**1.00**	5	5_92	NA	NA	NA	NA	92	NA	NA
**kshv-miR-K12-6-3p**	0.18	-0.01	-0.29	-0.04									-0.04	-1.03	NA	NA	**-1.03**	0.48	0.31	**1.00**	**1.00**	5	5_93	NA	NA	NA	NA	93	NA	NA
**ebv-miR-BART9**	-0.17	-0.32	0.29	-0.06									-0.06	-1.05	NA	NA	**-1.05**	0.48	0.14	**1.00**	**1.00**	5	5_94	NA	NA	NA	NA	94	NA	NA
**hsa-miR-483**	0.01	-0.02	0.12	0.04									0.04	1.03	NA	NA	**1.03**	0.49	0.75	**1.00**	**1.00**	5	5_95	NA	NA	NA	NA	95	NA	NA
**hcmv-miR-US25-2-3p**	0.19	-0.41	0.00	-0.08									-0.08	-1.05	NA	NA	**-1.05**	0.49	0.20	**1.00**	**1.00**	5	5_96	NA	NA	NA	NA	96	NA	NA
**kshv-miR-K12-11**	0.03	-0.10	0.14	0.03									0.03	1.02	NA	NA	**1.02**	0.50	0.66	**1.00**	**1.00**	5	5_97	NA	NA	NA	NA	97	NA	NA
**ebv-miR-BART13**	-0.03	0.11	0.06	0.04									0.04	1.03	NA	NA	**1.03**	0.50	0.75	**1.00**	**1.00**	5	5_98	NA	NA	NA	NA	98	NA	NA
**hsa-miR-380-5p**	-0.16	0.02	0.17	0.01									0.01	1.01	NA	NA	**1.01**	0.50	0.53	**1.00**	**1.00**	5	5_99	NA	NA	NA	NA	99	NA	NA
**ebv-miR-BHRF1-1**	0.04	0.13	-0.12	0.02									0.02	1.01	NA	NA	**1.01**	0.50	0.62	**1.00**	**1.00**	5	5_100	NA	NA	NA	NA	100	NA	NA
**hsa-miR-516-5p**	0.15	-0.04	-0.04	0.03									0.03	1.02	NA	NA	**1.02**	0.51	0.64	**1.00**	**1.00**	5	5_101	NA	NA	NA	NA	101	NA	NA
**ebv-miR-BART20-5p**	-0.11	-0.05	0.19	0.01									0.01	1.01	NA	NA	**1.01**	0.51	0.54	**1.00**	**1.00**	5	5_102	NA	NA	NA	NA	102	NA	NA
**hsa-miR-526c**	0.08	0.12	-0.42	-0.08									-0.08	-1.05	NA	NA	**-1.05**	0.53	0.21	**1.00**	**1.00**	5	5_103	NA	NA	NA	NA	103	NA	NA
**hsa-miR-337**	0.15	-0.07	-0.04	0.01									0.01	1.01	NA	NA	**1.01**	0.53	0.60	**1.00**	**1.00**	5	5_104	NA	NA	NA	NA	104	NA	NA
**hsa-miR-532**	-0.06	0.10	0.05	0.03									0.03	1.02	NA	NA	**1.02**	0.54	0.70	**1.00**	**1.00**	5	5_105	NA	NA	NA	NA	105	NA	NA
**ebv-miR-BART18**	-0.20	0.10	0.12	0.01									0.01	1.01	NA	NA	**1.01**	0.55	0.43	**1.00**	**1.00**	5	5_106	NA	NA	NA	NA	106	NA	NA
**hsa-miR-17-5p**	-0.02	-0.09	0.15	0.01									0.01	1.01	NA	NA	**1.01**	0.55	0.62	**1.00**	**1.00**	5	5_107	NA	NA	NA	NA	107	NA	NA
**hsa-miR-139**	-0.03	0.18	-0.27	-0.04									-0.04	-1.03	NA	NA	**-1.03**	0.55	0.29	**1.00**	**1.00**	5	5_108	NA	NA	NA	NA	108	NA	NA
**hsa-miR-30e-5p**	0.11	-0.13	0.02	0.00									0.00	-1.00	NA	NA	**-1.00**	0.56	0.58	**1.00**	**1.00**	5	5_109	NA	NA	NA	NA	109	NA	NA
**kshv-miR-K12-9***	-0.03	0.15	-0.08	0.01									0.01	1.01	NA	NA	**1.01**	0.56	0.58	**1.00**	**1.00**	5	5_110	NA	NA	NA	NA	110	NA	NA
**hsa-miR-449**	0.00	0.00	0.08	0.02									0.02	1.02	NA	NA	**1.02**	0.56	0.72	**1.00**	**1.00**	5	5_111	NA	NA	NA	NA	111	NA	NA
**ebv-miR-BART8-5p**	0.06	-0.10	0.04	0.00									0.00	1.00	NA	NA	**1.00**	0.57	0.62	**1.00**	**1.00**	5	5_112	NA	NA	NA	NA	112	NA	NA

ID	log2ratio_Agilent_1	log2ratio_Agilent_2	log2ratio_Agilent_3	avg_log2ratio_Agilent	log2ratio_Exiqon_1	log2ratio_Exiqon_2	log2ratio_Exiqon_3	avg_log2ratio_Exiqon	log2ratio_Illumina_1	log2ratio_Illumina_2	log2ratio_Illumina_3	avg_log2ratio_Illumina	avg_log2ratio_all	avg_FoldChange_Agilent	avg_FoldChange_Exiqon	avg_FoldChange_Illumina	avg_FoldChange_all	p.value.up	p.value.down	adjusted.p.value.up	adjusted.p.value.down	Index	Index_Rank	RPrank.1	RPrank.2	RPrank.3	RPrank.4	RPrank.5	RPrank.6	RPrank.7
hsa-miR-199a*	0.16	-0.10	-0.17	-0.04									-0.04	-1.03	NA	NA	-1.03	0.58	0.35	1.00	1.00	5	5_113	NA	NA	NA	NA	113	NA	NA
kshv-miR-K12-8	-0.27	0.23	-0.12	-0.05									-0.05	-1.04	NA	NA	-1.04	0.59	0.16	1.00	1.00	5	5_114	NA	NA	NA	NA	114	NA	NA
hsa-miR-518a	0.06	-0.03	0.00	0.01									0.01	1.01	NA	NA	1.01	0.59	0.67	1.00	1.00	5	5_115	NA	NA	NA	NA	115	NA	NA
hsa-miR-422b	-0.09	0.03	0.09	0.01									0.01	1.00	NA	NA	1.00	0.59	0.64	1.00	1.00	5	5_116	NA	NA	NA	NA	116	NA	NA
NC1_00000215	0.05	-0.01	-0.02	0.01									0.01	1.01	NA	NA	1.01	0.59	0.67	1.00	1.00	5	5_117	NA	NA	NA	NA	117	NA	NA
hcmv-miR-US4	0.03	0.07	-0.06	0.02									0.02	1.01	NA	NA	1.01	0.61	0.64	1.00	1.00	5	5_118	NA	NA	NA	NA	118	NA	NA
hsa-miR-151	-0.14	0.04	0.09	-0.01									-0.01	-1.00	NA	NA	-1.00	0.61	0.53	1.00	1.00	5	5_119	NA	NA	NA	NA	119	NA	NA
dmr_6	-0.10	-0.17	0.18	-0.03									-0.03	-1.02	NA	NA	-1.02	0.61	0.39	1.00	1.00	5	5_120	NA	NA	NA	NA	120	NA	NA
kshv-miR-K12-3	-0.02	0.03	0.01	0.01									0.01	1.00	NA	NA	1.00	0.62	0.68	1.00	1.00	5	5_121	NA	NA	NA	NA	121	NA	NA
hur_6	0.01	-0.03	0.04	0.01									0.01	1.00	NA	NA	1.00	0.62	0.68	1.00	1.00	5	5_122	NA	NA	NA	NA	122	NA	NA
hsa-miR-189	-0.03	-0.10	0.12	0.00									0.00	-1.00	NA	NA	-1.00	0.62	0.58	1.00	1.00	5	5_123	NA	NA	NA	NA	123	NA	NA
ebv-miR-BART12	-0.13	0.08	0.04	0.00									0.00	-1.00	NA	NA	-1.00	0.63	0.56	1.00	1.00	5	5_124	NA	NA	NA	NA	124	NA	NA
hsa-miR-28	-0.14	0.02	0.09	-0.01									-0.01	-1.01	NA	NA	-1.01	0.63	0.53	1.00	1.00	5	5_125	NA	NA	NA	NA	125	NA	NA
ebv-miR-BART2	0.14	-0.20	-0.06	-0.04									-0.04	-1.03	NA	NA	-1.03	0.64	0.37	1.00	1.00	5	5_126	NA	NA	NA	NA	126	NA	NA
kshv-miR-K12-12	-0.15	0.17	-0.17	-0.05									-0.05	-1.04	NA	NA	-1.04	0.64	0.28	1.00	1.00	5	5_127	NA	NA	NA	NA	127	NA	NA
hur_2	0.00	0.00	0.00	0.00									0.00	1.00	NA	NA	1.00	0.65	0.66	1.00	1.00	5	5_128	NA	NA	NA	NA	128	NA	NA
hur_1	0.00	0.00	0.00	0.00									0.00	-1.00	NA	NA	-1.00	0.66	0.64	1.00	1.00	5	5_129	NA	NA	NA	NA	129	NA	NA
ebv-miR-BHRF1-3	-0.11	0.13	-0.07	-0.02									-0.02	-1.01	NA	NA	-1.01	0.67	0.46	1.00	1.00	5	5_130	NA	NA	NA	NA	130	NA	NA
hsa-miR-518d	-0.35	-0.26	0.19	-0.14									-0.14	-1.10	NA	NA	-1.10	0.68	0.04	1.00	1.00	5	5_131	NA	NA	NA	NA	131	NA	NA
hsa-miR-193a	0.01	-0.05	0.03	-0.01									-0.01	-1.01	NA	NA	-1.01	0.68	0.61	1.00	1.00	5	5_132	NA	NA	NA	NA	132	NA	NA
hsa-miR-30e-3p	-0.12	-0.23	0.15	-0.07									-0.07	-1.05	NA	NA	-1.05	0.68	0.29	1.00	1.00	5	5_133	NA	NA	NA	NA	133	NA	NA
dmr_285	0.12	-0.09	-0.21	-0.06									-0.06	-1.04	NA	NA	-1.04	0.68	0.31	1.00	1.00	5	5_134	NA	NA	NA	NA	134	NA	NA
hsa-miR-519b	0.08	-0.01	-0.58	-0.17									-0.17	-1.12	NA	NA	-1.12	0.68	0.07	1.00	1.00	5	5_135	NA	NA	NA	NA	135	NA	NA
kshv-miR-K12-1	0.01	0.06	-0.16	-0.03									-0.03	-1.02	NA	NA	-1.02	0.69	0.46	1.00	1.00	5	5_136	NA	NA	NA	NA	136	NA	NA
hsa-miR-216	0.13	-0.14	-0.24	-0.08									-0.08	-1.06	NA	NA	-1.06	0.71	0.22	1.00	1.00	5	5_137	NA	NA	NA	NA	137	NA	NA
ebv-miR-BART10	-0.21	0.00	0.06	-0.05									-0.05	-1.03	NA	NA	-1.03	0.72	0.32	1.00	1.00	5	5_138	NA	NA	NA	NA	138	NA	NA
hsa-miR-338	-0.09	-0.01	0.04	-0.02									-0.02	-1.01	NA	NA	-1.01	0.72	0.54	1.00	1.00	5	5_139	NA	NA	NA	NA	139	NA	NA
hsa-miR-127	-0.42	-0.51	0.19	-0.25									-0.25	-1.19	NA	NA	-1.19	0.72	0.00	1.00	0.69	5	5_140	NA	NA	NA	NA	140	NA	NA
hsa-miR-454-3p	-0.12	-0.14	0.12	-0.05									-0.05	-1.03	NA	NA	-1.03	0.73	0.37	1.00	1.00	5	5_141	NA	NA	NA	NA	141	NA	NA
hsa-miR-361	-0.14	-0.05	0.08	-0.04									-0.04	-1.03	NA	NA	-1.03	0.74	0.43	1.00	1.00	5	5_142	NA	NA	NA	NA	142	NA	NA
hsa-miR-590	-0.12	-0.12	0.11	-0.05									-0.05	-1.03	NA	NA	-1.03	0.74	0.39	1.00	1.00	5	5_143	NA	NA	NA	NA	143	NA	NA
hsa-miR-128a	0.02	-0.03	-0.05	-0.02									-0.02	-1.02	NA	NA	-1.02	0.74	0.52	1.00	1.00	5	5_144	NA	NA	NA	NA	144	NA	NA
hsa-miR-296	0.05	-0.19	-0.01	-0.05									-0.05	-1.04	NA	NA	-1.04	0.75	0.40	1.00	1.00	5	5_145	NA	NA	NA	NA	145	NA	NA
hsa-miR-490	0.04	-0.05	-0.11	-0.04									-0.04	-1.03	NA	NA	-1.03	0.76	0.46	1.00	1.00	5	5_146	NA	NA	NA	NA	146	NA	NA
hsa-miR-368	-0.19	-0.20	0.14	-0.08									-0.08	-1.06	NA	NA	-1.06	0.76	0.19	1.00	1.00	5	5_147	NA	NA	NA	NA	147	NA	NA
ebv-miR-BART19	0.00	-0.07	-0.02	-0.03									-0.03	-1.02	NA	NA	-1.02	0.76	0.52	1.00	1.00	5	5_148	NA	NA	NA	NA	148	NA	NA

ID	log2ratio_Agilent_1	log2ratio_Agilent_2	log2ratio_Agilent_3	avg_log2ratio_Agilent	log2ratio_Exiqon_1	log2ratio_Exiqon_2	log2ratio_Exiqon_3	avg_log2ratio_Exiqon	log2ratio_Illumina_1	log2ratio_Illumina_2	log2ratio_Illumina_3	avg_log2ratio_Illumina	avg_log2ratio_all	avg_FoldChange_Agilent	avg_FoldChange_Exiqon	avg_FoldChange_Illumina	avg_FoldChange_all	p.value.up	p.value.down	adjusted.p.value.up	adjusted.p.value.down	Index	Index_Rank	RPrank.1	RPrank.2	RPrank.3	RPrank.4	RPrank.5	RPrank.6	RPrank.7
hsa-miR-92	0.00	0.00	-0.10	-0.04									-0.04	-1.02	NA	NA	-1.02	0.76	0.48	1.00	1.00	5	5_149	NA	NA	NA	NA	149	NA	NA
NC1_00000197	-0.08	0.07	-0.12	-0.04									-0.04	-1.03	NA	NA	-1.03	0.77	0.41	1.00	1.00	5	5_150	NA	NA	NA	NA	150	NA	NA
hsa-miR-520a	-0.03	-0.01	-0.04	-0.03									-0.03	-1.02	NA	NA	-1.02	0.77	0.51	1.00	1.00	5	5_151	NA	NA	NA	NA	151	NA	NA
hsa-miR-517b	0.04	0.00	-0.56	-0.17									-0.17	-1.13	NA	NA	-1.13	0.78	0.09	1.00	1.00	5	5_152	NA	NA	NA	NA	152	NA	NA
hsa-miR-30a-5p	-0.08	0.09	-0.20	-0.06									-0.06	-1.05	NA	NA	-1.05	0.78	0.30	1.00	1.00	5	5_153	NA	NA	NA	NA	153	NA	NA
ebv-miR-BART15	-0.03	-0.26	0.04	-0.08									-0.08	-1.06	NA	NA	-1.06	0.78	0.29	1.00	1.00	5	5_154	NA	NA	NA	NA	154	NA	NA
hsa-miR-520h	-0.07	0.09	-0.26	-0.08									-0.08	-1.06	NA	NA	-1.06	0.78	0.23	1.00	1.00	5	5_155	NA	NA	NA	NA	155	NA	NA
hcmv-miR-US5-1	0.06	-0.61	-0.04	-0.20									-0.20	-1.15	NA	NA	-1.15	0.79	0.05	1.00	1.00	5	5_156	NA	NA	NA	NA	156	NA	NA
hsa-miR-140	-0.14	-0.10	0.07	-0.06									-0.06	-1.04	NA	NA	-1.04	0.79	0.35	1.00	1.00	5	5_157	NA	NA	NA	NA	157	NA	NA
hsa-miR-520c	-0.11	0.09	-0.24	-0.09									-0.09	-1.06	NA	NA	-1.06	0.80	0.22	1.00	1.00	5	5_158	NA	NA	NA	NA	158	NA	NA
hsa-miR-582	0.05	-0.13	-0.21	-0.10									-0.10	-1.07	NA	NA	-1.07	0.82	0.23	1.00	1.00	5	5_159	NA	NA	NA	NA	159	NA	NA
kshv-miR-K12-2	-0.22	-0.61	0.12	-0.23									-0.23	-1.18	NA	NA	-1.18	0.82	0.01	1.00	1.00	5	5_160	NA	NA	NA	NA	160	NA	NA
hcmv-miR-UL22A	-0.18	-0.21	0.10	-0.10									-0.10	-1.07	NA	NA	-1.07	0.82	0.18	1.00	1.00	5	5_161	NA	NA	NA	NA	161	NA	NA
hsa-miR-450	-0.19	0.06	-0.10	-0.07									-0.07	-1.05	NA	NA	-1.05	0.83	0.25	1.00	1.00	5	5_162	NA	NA	NA	NA	162	NA	NA
hsa-miR-33	-0.05	-0.15	0.00	-0.07									-0.07	-1.05	NA	NA	-1.05	0.83	0.37	1.00	1.00	5	5_163	NA	NA	NA	NA	163	NA	NA
hsa-miR-301	-0.10	-0.10	0.00	-0.06									-0.06	-1.05	NA	NA	-1.05	0.84	0.38	1.00	1.00	5	5_164	NA	NA	NA	NA	164	NA	NA
hsa-miR-425-5p	-0.12	-0.11	0.02	-0.07									-0.07	-1.05	NA	NA	-1.05	0.84	0.35	1.00	1.00	5	5_165	NA	NA	NA	NA	165	NA	NA
kshv-miR-K12-10b	-0.04	-0.02	-0.16	-0.07									-0.07	-1.05	NA	NA	-1.05	0.85	0.34	1.00	1.00	5	5_166	NA	NA	NA	NA	166	NA	NA
NC2_00106057	0.03	-0.18	-0.13	-0.09									-0.09	-1.07	NA	NA	-1.07	0.85	0.27	1.00	1.00	5	5_167	NA	NA	NA	NA	167	NA	NA
hsa-miR-516-3p	0.00	-0.14	-0.06	-0.07									-0.07	-1.05	NA	NA	-1.05	0.86	0.33	1.00	1.00	5	5_168	NA	NA	NA	NA	168	NA	NA
hsa-miR-455	-0.04	-0.21	0.00	-0.08									-0.08	-1.06	NA	NA	-1.06	0.86	0.29	1.00	1.00	5	5_169	NA	NA	NA	NA	169	NA	NA
hsa-miR-125a	-0.18	0.03	-0.12	-0.09									-0.09	-1.07	NA	NA	-1.07	0.87	0.22	1.00	1.00	5	5_170	NA	NA	NA	NA	170	NA	NA
hsa-miR-362	-0.08	-0.15	0.00	-0.08									-0.08	-1.05	NA	NA	-1.05	0.87	0.32	1.00	1.00	5	5_171	NA	NA	NA	NA	171	NA	NA
hsa-miR-128b	-0.16	-0.14	0.04	-0.09									-0.09	-1.06	NA	NA	-1.06	0.87	0.25	1.00	1.00	5	5_172	NA	NA	NA	NA	172	NA	NA
ebv-miR-BHRF1-2*	-0.20	0.08	-0.21	-0.11									-0.11	-1.08	NA	NA	-1.08	0.87	0.13	1.00	1.00	5	5_173	NA	NA	NA	NA	173	NA	NA
hsa-miR-302a*	0.02	-0.20	-0.14	-0.11									-0.11	-1.08	NA	NA	-1.08	0.87	0.24	1.00	1.00	5	5_174	NA	NA	NA	NA	174	NA	NA
hsa-miR-524	0.05	-0.24	-0.33	-0.18									-0.18	-1.13	NA	NA	-1.13	0.89	0.08	1.00	1.00	5	5_175	NA	NA	NA	NA	175	NA	NA
hsa-miR-199b	-0.19	-0.03	-0.03	-0.08									-0.08	-1.06	NA	NA	-1.06	0.89	0.23	1.00	1.00	5	5_176	NA	NA	NA	NA	176	NA	NA
ebv-miR-BART3-5p	-0.40	0.09	-0.32	-0.21									-0.21	-1.16	NA	NA	-1.16	0.90	0.02	1.00	1.00	5	5_177	NA	NA	NA	NA	177	NA	NA
hsa-miR-574	-0.12	0.02	-0.38	-0.16									-0.16	-1.12	NA	NA	-1.12	0.90	0.10	1.00	1.00	5	5_178	NA	NA	NA	NA	178	NA	NA
hur_5	-0.21	-0.06	-0.01	-0.10									-0.10	-1.07	NA	NA	-1.07	0.90	0.18	1.00	1.00	5	5_179	NA	NA	NA	NA	179	NA	NA
hsa-miR-502	-0.05	-0.25	-0.03	-0.11									-0.11	-1.08	NA	NA	-1.08	0.90	0.22	1.00	1.00	5	5_180	NA	NA	NA	NA	180	NA	NA
hcmv-miR-US5-2	-0.14	-0.40	0.02	-0.17									-0.17	-1.13	NA	NA	-1.13	0.92	0.09	1.00	1.00	5	5_181	NA	NA	NA	NA	181	NA	NA
hsa-miR-628	-0.24	0.01	-0.19	-0.14									-0.14	-1.10	NA	NA	-1.10	0.92	0.10	1.00	1.00	5	5_182	NA	NA	NA	NA	182	NA	NA
ebv-miR-BART16	-0.09	-0.03	-0.38	-0.17									-0.17	-1.12	NA	NA	-1.12	0.92	0.11	1.00	1.00	5	5_183	NA	NA	NA	NA	183	NA	NA
hsa-miR-615	-0.25	-0.29	0.03	-0.17									-0.17	-1.12	NA	NA	-1.12	0.93	0.06	1.00	1.00	5	5_184	NA	NA	NA	NA	184	NA	NA

ID	log2ratio_Agilent_1	log2ratio_Agilent_2	log2ratio_Agilent_3	avg_log2ratio_Agilent	log2ratio_Exiqon_1	log2ratio_Exiqon_2	log2ratio_Exiqon_3	avg_log2ratio_Exiqon	log2ratio_Illumina_1	log2ratio_Illumina_2	log2ratio_Illumina_3	avg_log2ratio_Illumina	avg_log2ratio_all	avg_FoldChange_Agilent	avg_FoldChange_Exiqon	avg_FoldChange_Illumina	**avg_FoldChange_all**	p.value.up	p.value.down	**adjusted.p.value.up**	**adjusted.p.value.down**	Index	Index_Rank	RPrank.1	RPrank.2	RPrank.3	RPrank.4	RPrank.5	RPrank.6	RPrank.7
**hsa-miR-30a-3p**	-0.09	-0.13	-0.12	-0.11									-0.11	-1.08	NA	NA	**-1.08**	0.94	0.23	**1.00**	**1.00**	5	5_185	NA	NA	NA	NA	185	NA	NA
**hur_4**	-0.13	-0.16	-0.07	-0.12									-0.12	-1.09	NA	NA	**-1.09**	0.95	0.18	**1.00**	**1.00**	5	5_186	NA	NA	NA	NA	186	NA	NA
**hsa-miR-302b***	-0.05	-0.20	-0.17	-0.14									-0.14	-1.10	NA	NA	**-1.10**	0.95	0.14	**1.00**	**1.00**	5	5_187	NA	NA	NA	NA	187	NA	NA
**hsa-miR-501**	-0.07	-0.32	-0.10	-0.16									-0.16	-1.12	NA	NA	**-1.12**	0.95	0.10	**1.00**	**1.00**	5	5_188	NA	NA	NA	NA	188	NA	NA
**hsa-miR-374**	-0.21	-0.20	-0.02	-0.15									-0.15	-1.11	NA	NA	**-1.11**	0.96	0.10	**1.00**	**1.00**	5	5_189	NA	NA	NA	NA	189	NA	NA
**hsa-miR-488**	-0.15	-0.27	-0.03	-0.15									-0.15	-1.11	NA	NA	**-1.11**	0.96	0.11	**1.00**	**1.00**	5	5_190	NA	NA	NA	NA	190	NA	NA
**hsa-miR-34c**	-0.10	-0.36	-0.08	-0.18									-0.18	-1.13	NA	NA	**-1.13**	0.96	0.09	**1.00**	**1.00**	5	5_191	NA	NA	NA	NA	191	NA	NA
**hcmv-miR-UL36**	-0.31	-0.23	-0.02	-0.19									-0.19	-1.14	NA	NA	**-1.14**	0.96	0.05	**1.00**	**1.00**	5	5_192	NA	NA	NA	NA	192	NA	NA
**hsa-miR-129**	-0.32	-0.10	-0.09	-0.17									-0.17	-1.12	NA	NA	**-1.12**	0.96	0.06	**1.00**	**1.00**	5	5_193	NA	NA	NA	NA	193	NA	NA
**hsa-miR-371**	-0.03	-0.43	-0.39	-0.28									-0.28	-1.22	NA	NA	**-1.22**	0.98	0.01	**1.00**	**1.00**	5	5_194	NA	NA	NA	NA	194	NA	NA
**hsa-miR-520b**	-0.17	-0.09	-0.43	-0.23									-0.23	-1.17	NA	NA	**-1.17**	0.98	0.03	**1.00**	**1.00**	5	5_195	NA	NA	NA	NA	195	NA	NA
**ebv-miR-BART11-5p**	-0.20	-0.37	-0.06	-0.21									-0.21	-1.16	NA	NA	**-1.16**	0.98	0.04	**1.00**	**1.00**	5	5_196	NA	NA	NA	NA	196	NA	NA
**hsa-miR-29b-1***					0.49	0.72	0.70	0.64					0.64	NA	1.55	NA	**1.55**	0.00	1.00	**0.00**	**1.00**	6	6_1	NA	NA	NA	NA	NA	1	NA
**hsa-miR-222***					0.71	0.52	0.62	0.62					0.62	NA	1.53	NA	**1.53**	0.00	1.00	**0.00**	**1.00**	6	6_2	NA	NA	NA	NA	NA	2	NA
**miRPlus_17896**					0.47	0.61	0.36	0.48					0.48	NA	1.40	NA	**1.40**	0.00	1.00	**0.02**	**1.00**	6	6_3	NA	NA	NA	NA	NA	3	NA
**hsa-miR-31***					0.39	0.42	0.37	0.40					0.40	NA	1.32	NA	**1.32**	0.00	1.00	**0.07**	**1.00**	6	6_4	NA	NA	NA	NA	NA	4	NA
**miRPlus_17848**					0.38	0.30	0.28	0.32					0.32	NA	1.25	NA	**1.25**	0.00	1.00	**0.26**	**1.00**	6	6_5	NA	NA	NA	NA	NA	5	NA
**hsa-miR-29a***					0.23	0.26	0.25	0.25					0.25	NA	1.19	NA	**1.19**	0.00	1.00	**1.00**	**1.00**	6	6_6	NA	NA	NA	NA	NA	6	NA
**hsa-miR-145***					0.31	0.19	0.12	0.21					0.21	NA	1.16	NA	**1.16**	0.01	1.00	**1.00**	**1.00**	6	6_7	NA	NA	NA	NA	NA	7	NA
**miRPlus_17828**					0.20	0.17	0.19	0.19					0.19	NA	1.14	NA	**1.14**	0.01	1.00	**1.00**	**1.00**	6	6_8	NA	NA	NA	NA	NA	8	NA
**hsa-miR-27a***					0.26	0.20	0.14	0.20					0.20	NA	1.15	NA	**1.15**	0.01	1.00	**1.00**	**1.00**	6	6_9	NA	NA	NA	NA	NA	9	NA
**miRPlus_32953**					0.40	0.16	0.06	0.21					0.21	NA	1.15	NA	**1.15**	0.01	0.99	**1.00**	**1.00**	6	6_10	NA	NA	NA	NA	NA	10	NA
**hsa-miR-374a***					0.26	0.23	0.07	0.18					0.18	NA	1.14	NA	**1.14**	0.02	0.99	**1.00**	**1.00**	6	6_11	NA	NA	NA	NA	NA	11	NA
**hsa-miR-629***					0.09	0.12	0.27	0.16					0.16	NA	1.12	NA	**1.12**	0.03	0.98	**1.00**	**1.00**	6	6_12	NA	NA	NA	NA	NA	12	NA
**hsa-miR-502-5p**					0.28	0.05	0.14	0.15					0.15	NA	1.11	NA	**1.11**	0.04	0.99	**1.00**	**1.00**	6	6_13	NA	NA	NA	NA	NA	13	NA
**miRPlus_17827**					0.18	0.11	0.13	0.14					0.14	NA	1.10	NA	**1.10**	0.04	0.99	**1.00**	**1.00**	6	6_14	NA	NA	NA	NA	NA	14	NA
**hsa-miR-132***					0.18	0.09	0.15	0.14					0.14	NA	1.10	NA	**1.10**	0.04	0.99	**1.00**	**1.00**	6	6_15	NA	NA	NA	NA	NA	15	NA
**miRPlus_32900**					0.29	0.12	0.04	0.15					0.15	NA	1.11	NA	**1.11**	0.05	0.98	**1.00**	**1.00**	6	6_16	NA	NA	NA	NA	NA	16	NA
**hsa-let-7a***					0.21	0.07	0.13	0.14					0.14	NA	1.10	NA	**1.10**	0.05	0.99	**1.00**	**1.00**	6	6_17	NA	NA	NA	NA	NA	17	NA
**hsa-miR-30a***					0.36	0.06	0.05	0.16					0.16	NA	1.11	NA	**1.11**	0.06	0.97	**1.00**	**1.00**	6	6_18	NA	NA	NA	NA	NA	18	NA
**miRPlus_32902**					0.08	0.14	0.10	0.11					0.11	NA	1.08	NA	**1.08**	0.06	0.98	**1.00**	**1.00**	6	6_19	NA	NA	NA	NA	NA	19	NA
**miRPlus_17847**					0.26	0.03	0.11	0.13					0.13	NA	1.10	NA	**1.10**	0.06	0.97	**1.00**	**1.00**	6	6_20	NA	NA	NA	NA	NA	20	NA
**hsa-miR-17***					0.20	0.15	0.03	0.13					0.13	NA	1.09	NA	**1.09**	0.07	0.97	**1.00**	**1.00**	6	6_21	NA	NA	NA	NA	NA	21	NA
**miRPlus_28535**					0.13	0.07	0.15	0.11					0.11	NA	1.08	NA	**1.08**	0.07	0.98	**1.00**	**1.00**	6	6_22	NA	NA	NA	NA	NA	22	NA
**hsa-miR-20b***					0.03	0.08	0.16	0.09					0.09	NA	1.07	NA	**1.07**	0.09	0.95	**1.00**	**1.00**	6	6_23	NA	NA	NA	NA	NA	23	NA
**hsa-miR-181a-2***					0.16	0.10	0.06	0.11					0.11	NA	1.08	NA	**1.08**	0.11	0.98	**1.00**	**1.00**	6	6_24	NA	NA	NA	NA	NA	24	NA

ID	log2ratio_Agilent_1	log2ratio_Agilent_2	log2ratio_Agilent_3	avg_log2ratio_Agilent	log2ratio_Exiqon_1	log2ratio_Exiqon_2	log2ratio_Exiqon_3	avg_log2ratio_Exiqon	log2ratio_Illumina_1	log2ratio_Illumina_2	log2ratio_Illumina_3	avg_log2ratio_Illumina	avg_log2ratio_all	avg_FoldChange_Agilent	avg_FoldChange_Exiqon	avg_FoldChange_Illumina	**avg_FoldChange_all**	p.value.up	p.value.down	**adjusted.p.value.up**	**adjusted.p.value.down**	Index	Index_Rank	RPrank.1	RPrank.2	RPrank.3	RPrank.4	RPrank.5	RPrank.6	RPrank.7
**hsa-miR-525-3p**					-0.19	0.09	0.17	0.02					0.02	NA	1.02	NA	**1.02**	0.12	0.57	**1.00**	**1.00**	6	6_25	NA	NA	NA	NA	NA	25	NA
**miRPlus_17834**					0.23	0.03	0.07	0.11					0.11	NA	1.08	NA	**1.08**	0.12	0.96	**1.00**	**1.00**	6	6_26	NA	NA	NA	NA	NA	26	NA
**miRPlus_17861**					0.07	0.05	0.15	0.09					0.09	NA	1.06	NA	**1.06**	0.12	0.95	**1.00**	**1.00**	6	6_27	NA	NA	NA	NA	NA	27	NA
**hsa-miR-22***					0.03	0.10	0.10	0.08					0.08	NA	1.06	NA	**1.06**	0.13	0.94	**1.00**	**1.00**	6	6_28	NA	NA	NA	NA	NA	28	NA
**miRPlus_17941**					0.17	0.03	0.10	0.10					0.10	NA	1.07	NA	**1.07**	0.13	0.96	**1.00**	**1.00**	6	6_29	NA	NA	NA	NA	NA	29	NA
**miRPlus_17811**					0.01	0.11	0.09	0.07					0.07	NA	1.05	NA	**1.05**	0.14	0.91	**1.00**	**1.00**	6	6_30	NA	NA	NA	NA	NA	30	NA
**miRPlus_17920**					0.30	0.03	0.00	0.11					0.11	NA	1.08	NA	**1.08**	0.15	0.88	**1.00**	**1.00**	6	6_31	NA	NA	NA	NA	NA	31	NA
**hsa-miR-106b***					0.12	0.06	0.07	0.08					0.08	NA	1.06	NA	**1.06**	0.16	0.96	**1.00**	**1.00**	6	6_32	NA	NA	NA	NA	NA	32	NA
**miRPlus_11201**					0.12	0.03	0.10	0.08					0.08	NA	1.06	NA	**1.06**	0.16	0.95	**1.00**	**1.00**	6	6_33	NA	NA	NA	NA	NA	33	NA
**miRPlus_17956**					0.24	-0.05	0.08	0.09					0.09	NA	1.07	NA	**1.07**	0.17	0.80	**1.00**	**1.00**	6	6_34	NA	NA	NA	NA	NA	34	NA
**miRPlus_32832**					0.02	0.01	0.16	0.06					0.06	NA	1.05	NA	**1.05**	0.17	0.86	**1.00**	**1.00**	6	6_35	NA	NA	NA	NA	NA	35	NA
**miRPlus_17840**					0.01	0.03	0.16	0.07					0.07	NA	1.05	NA	**1.05**	0.18	0.86	**1.00**	**1.00**	6	6_36	NA	NA	NA	NA	NA	36	NA
**miRPlus_17945**					0.02	0.07	0.09	0.06					0.06	NA	1.04	NA	**1.04**	0.20	0.91	**1.00**	**1.00**	6	6_37	NA	NA	NA	NA	NA	37	NA
**hsa-miR-891a**					0.15	0.10	-0.02	0.08					0.08	NA	1.06	NA	**1.06**	0.20	0.85	**1.00**	**1.00**	6	6_38	NA	NA	NA	NA	NA	38	NA
**hsa-miR-34b***					0.04	0.13	0.02	0.06					0.06	NA	1.05	NA	**1.05**	0.21	0.88	**1.00**	**1.00**	6	6_39	NA	NA	NA	NA	NA	39	NA
**hsa-miR-519c-5p_MM1**					-0.08	0.09	0.09	0.03					0.03	NA	1.02	NA	**1.02**	0.23	0.74	**1.00**	**1.00**	6	6_40	NA	NA	NA	NA	NA	40	NA
**hsa-let-7d***					0.03	0.06	0.08	0.06					0.06	NA	1.04	NA	**1.04**	0.23	0.91	**1.00**	**1.00**	6	6_41	NA	NA	NA	NA	NA	41	NA
**hsa-miR-508-3p**					0.06	0.05	0.07	0.06					0.06	NA	1.04	NA	**1.04**	0.23	0.92	**1.00**	**1.00**	6	6_42	NA	NA	NA	NA	NA	42	NA
**hsa-miR-93***					0.24	-0.03	0.05	0.09					0.09	NA	1.06	NA	**1.06**	0.23	0.81	**1.00**	**1.00**	6	6_43	NA	NA	NA	NA	NA	43	NA
**hsa-miR-32***					0.13	0.04	0.03	0.07					0.07	NA	1.05	NA	**1.05**	0.25	0.92	**1.00**	**1.00**	6	6_44	NA	NA	NA	NA	NA	44	NA
**hsa_SNORD15A**					0.24	-0.02	0.03	0.09					0.09	NA	1.06	NA	**1.06**	0.25	0.84	**1.00**	**1.00**	6	6_45	NA	NA	NA	NA	NA	45	NA
**miRPlus_17868**					0.13	0.11	-0.09	0.05					0.05	NA	1.03	NA	**1.03**	0.26	0.65	**1.00**	**1.00**	6	6_46	NA	NA	NA	NA	NA	46	NA
**hsa-miR-488***					0.38	-0.15	-0.06	0.06					0.06	NA	1.04	NA	**1.04**	0.27	0.18	**1.00**	**1.00**	6	6_47	NA	NA	NA	NA	NA	47	NA
**hsa-miR-30e***					0.18	0.01	0.02	0.07					0.07	NA	1.05	NA	**1.05**	0.28	0.87	**1.00**	**1.00**	6	6_48	NA	NA	NA	NA	NA	48	NA
**hsa-miR-454***					-0.19	0.16	0.01	-0.01					-0.01	NA	-1.01	NA	**-1.01**	0.31	0.42	**1.00**	**1.00**	6	6_49	NA	NA	NA	NA	NA	49	NA
**hsa-miR-7-1***					0.10	0.03	0.04	0.06					0.06	NA	1.04	NA	**1.04**	0.32	0.89	**1.00**	**1.00**	6	6_50	NA	NA	NA	NA	NA	50	NA
**hsa-miR-33a***					0.15	-0.14	0.07	0.03					0.03	NA	1.02	NA	**1.02**	0.33	0.39	**1.00**	**1.00**	6	6_51	NA	NA	NA	NA	NA	51	NA
**hsa-miR-520d-3p**					0.17	0.00	0.02	0.06					0.06	NA	1.04	NA	**1.04**	0.34	0.84	**1.00**	**1.00**	6	6_52	NA	NA	NA	NA	NA	52	NA
**hsa-miR-493***					0.11	0.01	0.05	0.06					0.06	NA	1.04	NA	**1.04**	0.34	0.87	**1.00**	**1.00**	6	6_53	NA	NA	NA	NA	NA	53	NA
**miRPlus_17891**					0.06	0.03	0.06	0.05					0.05	NA	1.04	NA	**1.04**	0.35	0.87	**1.00**	**1.00**	6	6_54	NA	NA	NA	NA	NA	54	NA
**hsa-miR-500***					0.13	-0.12	0.08	0.03					0.03	NA	1.02	NA	**1.02**	0.35	0.48	**1.00**	**1.00**	6	6_55	NA	NA	NA	NA	NA	55	NA
**hsa-miR-525-5p**					-0.24	0.08	0.07	-0.03					-0.03	NA	-1.02	NA	**-1.02**	0.36	0.46	**1.00**	**1.00**	6	6_56	NA	NA	NA	NA	NA	56	NA
**miRPlus_17860**					0.30	-0.10	-0.07	0.04					0.04	NA	1.03	NA	**1.03**	0.36	0.28	**1.00**	**1.00**	6	6_57	NA	NA	NA	NA	NA	57	NA
**hsa-miR-99b***					0.17	-0.01	0.01	0.06					0.06	NA	1.04	NA	**1.04**	0.36	0.81	**1.00**	**1.00**	6	6_58	NA	NA	NA	NA	NA	58	NA
**hsa-miR-493**					-0.10	0.13	-0.01	0.01					0.01	NA	1.00	NA	**1.00**	0.36	0.54	**1.00**	**1.00**	6	6_59	NA	NA	NA	NA	NA	59	NA
**miRPlus_17812**					0.15	0.05	-0.08	0.04					0.04	NA	1.03	NA	**1.03**	0.36	0.65	**1.00**	**1.00**	6	6_60	NA	NA	NA	NA	NA	60	NA

**ID**	log2ratio_Agilent_1	log2ratio_Agilent_2	log2ratio_Agilent_3	avg_log2ratio_Agilent	log2ratio_Exiqon_1	log2ratio_Exiqon_2	log2ratio_Exiqon_3	avg_log2ratio_Exiqon	log2ratio_Illumina_1	log2ratio_Illumina_2	log2ratio_Illumina_3	avg_log2ratio_Illumina	avg_log2ratio_all	avg_FoldChange_Agilent	avg_FoldChange_Exiqon	avg_FoldChange_Illumina	**avg_FoldChange_all**	p.value.up	p.value.down	**adjusted.p.value.up**	**adjusted.p.value.down**	Index	Index_Rank	RPrank.1	RPrank.2	RPrank.3	RPrank.4	RPrank.5	RPrank.6	RPrank.7
**miRPlus_17821**					0.09	0.03	0.03	0.05					0.05	NA	1.04	NA	**1.04**	0.37	0.87	**1.00**	**1.00**	6	6_61	NA	NA	NA	NA	NA	61	NA
**miRPlus_30908**					0.02	0.02	0.07	0.04					0.04	NA	1.03	NA	**1.03**	0.38	0.83	**1.00**	**1.00**	6	6_62	NA	NA	NA	NA	NA	62	NA
**miRPlus_17900**					0.07	0.07	-0.02	0.04					0.04	NA	1.03	NA	**1.03**	0.38	0.78	**1.00**	**1.00**	6	6_63	NA	NA	NA	NA	NA	63	NA
**hsa-miR-96***					0.04	0.11	-0.07	0.03					0.03	NA	1.02	NA	**1.02**	0.38	0.61	**1.00**	**1.00**	6	6_64	NA	NA	NA	NA	NA	64	NA
**hsa-miR-125b-1***					0.23	-0.03	-0.01	0.06					0.06	NA	1.04	NA	**1.04**	0.39	0.67	**1.00**	**1.00**	6	6_65	NA	NA	NA	NA	NA	65	NA
**hsa-miR-378***					-0.07	0.05	0.06	0.01					0.01	NA	1.01	NA	**1.01**	0.40	0.71	**1.00**	**1.00**	6	6_66	NA	NA	NA	NA	NA	66	NA
**miRPlus_17833**					0.13	-0.04	0.05	0.04					0.04	NA	1.03	NA	**1.03**	0.41	0.72	**1.00**	**1.00**	6	6_67	NA	NA	NA	NA	NA	67	NA
**miRPlus_17897**					0.02	0.04	0.03	0.03					0.03	NA	1.02	NA	**1.02**	0.42	0.81	**1.00**	**1.00**	6	6_68	NA	NA	NA	NA	NA	68	NA
**hsa-let-7f-2***					0.11	0.00	0.02	0.04					0.04	NA	1.03	NA	**1.03**	0.44	0.81	**1.00**	**1.00**	6	6_69	NA	NA	NA	NA	NA	69	NA
**miRPlus_17930**					0.10	-0.03	0.04	0.04					0.04	NA	1.03	NA	**1.03**	0.46	0.75	**1.00**	**1.00**	6	6_70	NA	NA	NA	NA	NA	70	NA
**hsa-miR-520a-3p**					-0.03	0.10	-0.02	0.02					0.02	NA	1.01	NA	**1.01**	0.46	0.63	**1.00**	**1.00**	6	6_71	NA	NA	NA	NA	NA	71	NA
**hsa-miR-18b***					0.12	-0.02	0.01	0.04					0.04	NA	1.03	NA	**1.03**	0.46	0.77	**1.00**	**1.00**	6	6_72	NA	NA	NA	NA	NA	72	NA
**miRPlus_17880**					-0.16	0.13	-0.11	-0.04					-0.04	NA	-1.03	NA	**-1.03**	0.47	0.26	**1.00**	**1.00**	6	6_73	NA	NA	NA	NA	NA	73	NA
**hsa-let-7g***					-0.05	0.03	0.06	0.01					0.01	NA	1.01	NA	**1.01**	0.47	0.70	**1.00**	**1.00**	6	6_74	NA	NA	NA	NA	NA	74	NA
**hsa-miR-29c***					0.14	-0.08	0.03	0.03					0.03	NA	1.02	NA	**1.02**	0.48	0.58	**1.00**	**1.00**	6	6_75	NA	NA	NA	NA	NA	75	NA
**hsa-miR-548a-5p**					-0.04	0.10	-0.03	0.01					0.01	NA	1.01	NA	**1.01**	0.48	0.56	**1.00**	**1.00**	6	6_76	NA	NA	NA	NA	NA	76	NA
**hsa-miR-422a_MM2**					0.12	-0.06	0.04	0.03					0.03	NA	1.02	NA	**1.02**	0.48	0.65	**1.00**	**1.00**	6	6_77	NA	NA	NA	NA	NA	77	NA
**hsa-miR-34a***					0.11	0.02	-0.01	0.04					0.04	NA	1.03	NA	**1.03**	0.48	0.75	**1.00**	**1.00**	6	6_78	NA	NA	NA	NA	NA	78	NA
**hsa-miR-188-3p**					-0.10	-0.02	0.09	-0.01					-0.01	NA	-1.01	NA	**-1.01**	0.48	0.51	**1.00**	**1.00**	6	6_79	NA	NA	NA	NA	NA	79	NA
**hsa-miR-216a**					0.04	-0.01	0.04	0.02					0.02	NA	1.02	NA	**1.02**	0.49	0.78	**1.00**	**1.00**	6	6_80	NA	NA	NA	NA	NA	80	NA
**miRPlus_17858**					-0.17	0.01	0.09	-0.02					-0.02	NA	-1.02	NA	**-1.02**	0.49	0.47	**1.00**	**1.00**	6	6_81	NA	NA	NA	NA	NA	81	NA
**miRPlus_28454**					-0.01	0.06	0.00	0.02					0.02	NA	1.01	NA	**1.01**	0.50	0.71	**1.00**	**1.00**	6	6_82	NA	NA	NA	NA	NA	82	NA
**miRPlus_17912**					0.06	-0.07	0.06	0.02					0.02	NA	1.01	NA	**1.01**	0.50	0.61	**1.00**	**1.00**	6	6_83	NA	NA	NA	NA	NA	83	NA
**miRPlus_17653**					-0.01	-0.06	0.09	0.01					0.01	NA	1.00	NA	**1.00**	0.50	0.56	**1.00**	**1.00**	6	6_84	NA	NA	NA	NA	NA	84	NA
**hsa-miR-335***					0.11	0.02	-0.02	0.04					0.04	NA	1.03	NA	**1.03**	0.51	0.72	**1.00**	**1.00**	6	6_85	NA	NA	NA	NA	NA	85	NA
**hsa-miR-214***					0.14	-0.05	0.01	0.03					0.03	NA	1.02	NA	**1.02**	0.51	0.61	**1.00**	**1.00**	6	6_86	NA	NA	NA	NA	NA	86	NA
**miRPlus_17838**					0.11	0.03	-0.08	0.02					0.02	NA	1.02	NA	**1.02**	0.53	0.57	**1.00**	**1.00**	6	6_87	NA	NA	NA	NA	NA	87	NA
**hsa-miR-337-5p**					-0.01	0.03	0.03	0.02					0.02	NA	1.01	NA	**1.01**	0.54	0.72	**1.00**	**1.00**	6	6_88	NA	NA	NA	NA	NA	88	NA
**miRPlus_17865**					0.00	0.05	0.00	0.01					0.01	NA	1.01	NA	**1.01**	0.55	0.69	**1.00**	**1.00**	6	6_89	NA	NA	NA	NA	NA	89	NA
**hsa-miR-100***					-0.04	0.00	0.06	0.01					0.01	NA	1.00	NA	**1.00**	0.55	0.66	**1.00**	**1.00**	6	6_90	NA	NA	NA	NA	NA	90	NA
**miRPlus_17937**					0.11	-0.01	-0.02	0.03					0.03	NA	1.02	NA	**1.02**	0.56	0.67	**1.00**	**1.00**	6	6_91	NA	NA	NA	NA	NA	91	NA
**hsa-miR-192***					-0.04	-0.02	0.07	0.00					0.00	NA	1.00	NA	**1.00**	0.56	0.60	**1.00**	**1.00**	6	6_92	NA	NA	NA	NA	NA	92	NA
**hsa-miR-130b***					-0.11	0.07	-0.01	-0.02					-0.02	NA	-1.01	NA	**-1.01**	0.56	0.48	**1.00**	**1.00**	6	6_93	NA	NA	NA	NA	NA	93	NA
**miRPlus_17947**					-0.01	0.08	-0.10	-0.01					-0.01	NA	-1.01	NA	**-1.01**	0.57	0.43	**1.00**	**1.00**	6	6_94	NA	NA	NA	NA	NA	94	NA
**miRPlus_17819**					0.06	0.02	-0.01	0.02					0.02	NA	1.02	NA	**1.02**	0.58	0.71	**1.00**	**1.00**	6	6_95	NA	NA	NA	NA	NA	95	NA
**miRPlus_28534**					0.07	-0.02	0.02	0.02					0.02	NA	1.01	NA	**1.01**	0.58	0.70	**1.00**	**1.00**	6	6_96	NA	NA	NA	NA	NA	96	NA

ID	log2ratio_Agilent_1	log2ratio_Agilent_2	log2ratio_Agilent_3	avg_log2ratio_Agilent	log2ratio_Exiqon_1	log2ratio_Exiqon_2	log2ratio_Exiqon_3	avg_log2ratio_Exiqon	log2ratio_Illumina_1	log2ratio_Illumina_2	log2ratio_Illumina_3	avg_log2ratio_Illumina	avg_log2ratio_all	avg_FoldChange_Agilent	avg_FoldChange_Exiqon	avg_FoldChange_Illumina	**avg_FoldChange_all**	p.value.up	p.value.down	**adjusted.p.value.up**	**adjusted.p.value.down**	Index	Index_Rank	RPrank.1	RPrank.2	RPrank.3	RPrank.4	RPrank.5	RPrank.6	RPrank.7
**hsa-miR-297**					0.08	0.00	-0.01	0.02					0.02	NA	1.02	NA	**1.02**	0.58	0.69	**1.00**	**1.00**	6	6_97	NA	NA	NA	NA	NA	97	NA
**hsa-miR-19b-1***					-0.04	-0.04	0.07	0.00					0.00	NA	-1.00	NA	**-1.00**	0.59	0.52	**1.00**	**1.00**	6	6_98	NA	NA	NA	NA	NA	98	NA
**hsa_SNORD118**					0.04	0.04	-0.12	-0.01					-0.01	NA	-1.01	NA	**-1.01**	0.61	0.45	**1.00**	**1.00**	6	6_99	NA	NA	NA	NA	NA	99	NA
**miRPlus_30317**					-0.10	0.04	0.01	-0.02					-0.02	NA	-1.01	NA	**-1.01**	0.61	0.53	**1.00**	**1.00**	6	6_100	NA	NA	NA	NA	NA	100	NA
**hsa-miR-15a***					-0.10	0.04	0.01	-0.02					-0.02	NA	-1.01	NA	**-1.01**	0.61	0.53	**1.00**	**1.00**	6	6_101	NA	NA	NA	NA	NA	101	NA
**miRPlus_17957**					0.01	0.03	-0.03	0.00					0.00	NA	1.00	NA	**1.00**	0.62	0.60	**1.00**	**1.00**	6	6_102	NA	NA	NA	NA	NA	102	NA
**hsa-miR-143***					0.08	-0.09	0.03	0.01					0.01	NA	1.00	NA	**1.00**	0.63	0.51	**1.00**	**1.00**	6	6_103	NA	NA	NA	NA	NA	103	NA
**hsa-miR-101***					-0.09	0.02	0.03	-0.01					-0.01	NA	-1.01	NA	**-1.01**	0.63	0.56	**1.00**	**1.00**	6	6_104	NA	NA	NA	NA	NA	104	NA
**hsa-miR-616***					-0.11	0.04	0.01	-0.02					-0.02	NA	-1.02	NA	**-1.02**	0.63	0.50	**1.00**	**1.00**	6	6_105	NA	NA	NA	NA	NA	105	NA
**hsa-miR-491-3p**					0.03	-0.02	0.01	0.01					0.01	NA	1.01	NA	**1.01**	0.63	0.67	**1.00**	**1.00**	6	6_106	NA	NA	NA	NA	NA	106	NA
**hsa-miR-16-1***					0.03	0.03	-0.02	0.01					0.01	NA	1.01	NA	**1.01**	0.64	0.63	**1.00**	**1.00**	6	6_107	NA	NA	NA	NA	NA	107	NA
**miRPlus_29878**					0.01	-0.07	0.05	0.00					0.00	NA	-1.00	NA	**-1.00**	0.64	0.50	**1.00**	**1.00**	6	6_108	NA	NA	NA	NA	NA	108	NA
**hsa-let-7i***					0.04	-0.02	0.01	0.01					0.01	NA	1.01	NA	**1.01**	0.64	0.66	**1.00**	**1.00**	6	6_109	NA	NA	NA	NA	NA	109	NA
**hsa-miR-556-5p**					-0.19	0.02	0.04	-0.05					-0.05	NA	-1.03	NA	**-1.03**	0.65	0.41	**1.00**	**1.00**	6	6_110	NA	NA	NA	NA	NA	110	NA
**miRPlus_28790**					-0.06	0.05	-0.04	-0.02					-0.02	NA	-1.01	NA	**-1.01**	0.65	0.47	**1.00**	**1.00**	6	6_111	NA	NA	NA	NA	NA	111	NA
**miRPlus_17820**					-0.08	-0.01	0.04	-0.02					-0.02	NA	-1.01	NA	**-1.01**	0.65	0.55	**1.00**	**1.00**	6	6_112	NA	NA	NA	NA	NA	112	NA
**hsa-miR-30d***					0.08	-0.03	-0.01	0.02					0.02	NA	1.01	NA	**1.01**	0.65	0.59	**1.00**	**1.00**	6	6_113	NA	NA	NA	NA	NA	113	NA
**hsa-miR-19a***					0.10	-0.03	-0.03	0.01					0.01	NA	1.01	NA	**1.01**	0.67	0.53	**1.00**	**1.00**	6	6_114	NA	NA	NA	NA	NA	114	NA
**miRPlus_17932**					0.13	-0.09	-0.03	0.00					0.00	NA	1.00	NA	**1.00**	0.68	0.36	**1.00**	**1.00**	6	6_115	NA	NA	NA	NA	NA	115	NA
**hsa-miR-10a***					0.08	-0.01	-0.05	0.01					0.01	NA	1.00	NA	**1.00**	0.68	0.54	**1.00**	**1.00**	6	6_116	NA	NA	NA	NA	NA	116	NA
**miRPlus_31515**					-0.08	0.01	0.01	-0.02					-0.02	NA	-1.01	NA	**-1.01**	0.69	0.54	**1.00**	**1.00**	6	6_117	NA	NA	NA	NA	NA	117	NA
**miRPlus_17867**					-0.04	-0.04	0.04	-0.01					-0.01	NA	-1.01	NA	**-1.01**	0.70	0.49	**1.00**	**1.00**	6	6_118	NA	NA	NA	NA	NA	118	NA
**miRPlus_30209**					0.00	-0.01	0.01	0.00					0.00	NA	-1.00	NA	**-1.00**	0.70	0.60	**1.00**	**1.00**	6	6_119	NA	NA	NA	NA	NA	119	NA
**hsa_SNORD10**					-0.02	-0.10	0.05	-0.02					-0.02	NA	-1.02	NA	**-1.02**	0.71	0.36	**1.00**	**1.00**	6	6_120	NA	NA	NA	NA	NA	120	NA
**hsa_SNORD12**					-0.10	0.02	0.00	-0.03					-0.03	NA	-1.02	NA	**-1.02**	0.73	0.45	**1.00**	**1.00**	6	6_121	NA	NA	NA	NA	NA	121	NA
**miRPlus_17921**					0.15	-0.16	-0.24	-0.09					-0.09	NA	-1.06	NA	**-1.06**	0.73	0.05	**1.00**	**1.00**	6	6_122	NA	NA	NA	NA	NA	122	NA
**hsa-miR-425***					0.11	-0.07	-0.07	-0.01					-0.01	NA	-1.01	NA	**-1.01**	0.75	0.34	**1.00**	**1.00**	6	6_123	NA	NA	NA	NA	NA	123	NA
**hsa-miR-26a_MM1**					0.00	-0.02	0.00	-0.01					-0.01	NA	-1.00	NA	**-1.00**	0.75	0.54	**1.00**	**1.00**	6	6_124	NA	NA	NA	NA	NA	124	NA
**miRPlus_28350**					-0.02	-0.02	0.00	-0.01					-0.01	NA	-1.01	NA	**-1.01**	0.77	0.51	**1.00**	**1.00**	6	6_125	NA	NA	NA	NA	NA	125	NA
**hsa-miR-199a-5p**					0.00	0.00	-0.05	-0.02					-0.02	NA	-1.01	NA	**-1.01**	0.77	0.47	**1.00**	**1.00**	6	6_126	NA	NA	NA	NA	NA	126	NA
**hsa-miR-200c***					-0.05	-0.03	0.01	-0.02					-0.02	NA	-1.02	NA	**-1.02**	0.79	0.46	**1.00**	**1.00**	6	6_127	NA	NA	NA	NA	NA	127	NA
**hsa-miR-141***					-0.06	-0.01	-0.01	-0.03					-0.03	NA	-1.02	NA	**-1.02**	0.79	0.47	**1.00**	**1.00**	6	6_128	NA	NA	NA	NA	NA	128	NA
**hsa-miR-624***					0.02	-0.04	-0.02	-0.01					-0.01	NA	-1.01	NA	**-1.01**	0.80	0.46	**1.00**	**1.00**	6	6_129	NA	NA	NA	NA	NA	129	NA
**miRPlus_27869**					0.02	-0.02	-0.04	-0.02					-0.02	NA	-1.01	NA	**-1.01**	0.80	0.46	**1.00**	**1.00**	6	6_130	NA	NA	NA	NA	NA	130	NA
**miRPlus_30271**					0.09	-0.09	-0.07	-0.02					-0.02	NA	-1.02	NA	**-1.02**	0.80	0.27	**1.00**	**1.00**	6	6_131	NA	NA	NA	NA	NA	131	NA
**miRPlus_17824**					-0.42	-0.03	0.03	-0.14					-0.14	NA	-1.10	NA	**-1.10**	0.80	0.10	**1.00**	**1.00**	6	6_132	NA	NA	NA	NA	NA	132	NA

**ID**	log2ratio_Agilent_1	log2ratio_Agilent_2	log2ratio_Agilent_3	avg_log2ratio_Agilent	log2ratio_Exiqon_1	log2ratio_Exiqon_2	log2ratio_Exiqon_3	avg_log2ratio_Exiqon	log2ratio_Illumina_1	log2ratio_Illumina_2	log2ratio_Illumina_3	avg_log2ratio_Illumina	avg_log2ratio_all	avg_FoldChange_Agilent	avg_FoldChange_Exiqon	avg_FoldChange_Illumina	**avg_FoldChange_all**	p.value.up	p.value.down	**adjusted.p.value.up**	**adjusted.p.value.down**	Index	Index_Rank	RPrank.1	RPrank.2	RPrank.3	RPrank.4	RPrank.5	RPrank.6	RPrank.7
**miRPlus_17843**					-0.08	-0.02	0.01	-0.03					-0.03	NA	-1.02	NA	**-1.02**	0.80	0.43	**1.00**	**1.00**	6	6_133	NA	NA	NA	NA	NA	133	NA
**miRPlus_11239**					-0.03	0.01	-0.05	-0.03					-0.03	NA	-1.02	NA	**-1.02**	0.81	0.42	**1.00**	**1.00**	6	6_134	NA	NA	NA	NA	NA	134	NA
**miRPlus_27839**					0.08	-0.11	-0.06	-0.03					-0.03	NA	-1.02	NA	**-1.02**	0.82	0.23	**1.00**	**1.00**	6	6_135	NA	NA	NA	NA	NA	135	NA
**hsa-miR-15b***					-0.14	-0.03	0.02	-0.05					-0.05	NA	-1.04	NA	**-1.04**	0.82	0.32	**1.00**	**1.00**	6	6_136	NA	NA	NA	NA	NA	136	NA
**miRPlus_17926**					-0.04	-0.01	-0.04	-0.03					-0.03	NA	-1.02	NA	**-1.02**	0.82	0.42	**1.00**	**1.00**	6	6_137	NA	NA	NA	NA	NA	137	NA
**hsa-miR-499-3p**					-0.03	0.01	-0.09	-0.04					-0.04	NA	-1.03	NA	**-1.03**	0.83	0.33	**1.00**	**1.00**	6	6_138	NA	NA	NA	NA	NA	138	NA
**hsa-miR-380***					-0.19	0.02	-0.03	-0.07					-0.07	NA	-1.05	NA	**-1.05**	0.83	0.26	**1.00**	**1.00**	6	6_139	NA	NA	NA	NA	NA	139	NA
**hsa-miR-183***					0.02	-0.10	-0.01	-0.03					-0.03	NA	-1.02	NA	**-1.02**	0.84	0.31	**1.00**	**1.00**	6	6_140	NA	NA	NA	NA	NA	140	NA
**hsa-miR-524-3p**					-0.18	0.01	-0.02	-0.06					-0.06	NA	-1.04	NA	**-1.04**	0.84	0.28	**1.00**	**1.00**	6	6_141	NA	NA	NA	NA	NA	141	NA
**hsa-miR-185***					-0.13	0.03	-0.16	-0.09					-0.09	NA	-1.06	NA	**-1.06**	0.85	0.16	**1.00**	**1.00**	6	6_142	NA	NA	NA	NA	NA	142	NA
**hsa_SNORD14B**					0.03	-0.03	-0.11	-0.04					-0.04	NA	-1.03	NA	**-1.03**	0.85	0.28	**1.00**	**1.00**	6	6_143	NA	NA	NA	NA	NA	143	NA
**hsa-miR-550***					0.02	-0.12	-0.01	-0.04					-0.04	NA	-1.03	NA	**-1.03**	0.85	0.26	**1.00**	**1.00**	6	6_144	NA	NA	NA	NA	NA	144	NA
**miRPlus_17841**					-0.05	-0.03	-0.01	-0.03					-0.03	NA	-1.02	NA	**-1.02**	0.85	0.40	**1.00**	**1.00**	6	6_145	NA	NA	NA	NA	NA	145	NA
**miRPlus_17955**					0.04	-0.04	-0.17	-0.06					-0.06	NA	-1.04	NA	**-1.04**	0.85	0.19	**1.00**	**1.00**	6	6_146	NA	NA	NA	NA	NA	146	NA
**miRPlus_17869**					0.02	-0.03	-0.13	-0.05					-0.05	NA	-1.03	NA	**-1.03**	0.86	0.26	**1.00**	**1.00**	6	6_147	NA	NA	NA	NA	NA	147	NA
**hsa_SNORD4A**					0.05	-0.09	-0.09	-0.04					-0.04	NA	-1.03	NA	**-1.03**	0.87	0.20	**1.00**	**1.00**	6	6_148	NA	NA	NA	NA	NA	148	NA
**hsa-let-7e***					-0.10	-0.11	0.02	-0.06					-0.06	NA	-1.05	NA	**-1.05**	0.87	0.19	**1.00**	**1.00**	6	6_149	NA	NA	NA	NA	NA	149	NA
**hsa_SNORD6**					0.03	-0.06	-0.16	-0.06					-0.06	NA	-1.04	NA	**-1.04**	0.89	0.18	**1.00**	**1.00**	6	6_150	NA	NA	NA	NA	NA	150	NA
**hsa-miR-518d-3p**					-0.01	-0.05	-0.05	-0.04					-0.04	NA	-1.03	NA	**-1.03**	0.90	0.30	**1.00**	**1.00**	6	6_151	NA	NA	NA	NA	NA	151	NA
**hsa-miR-520a-5p**					-0.14	-0.13	0.01	-0.09					-0.09	NA	-1.06	NA	**-1.06**	0.90	0.14	**1.00**	**1.00**	6	6_152	NA	NA	NA	NA	NA	152	NA
**hsa-miR-589***					-0.02	-0.03	-0.08	-0.04					-0.04	NA	-1.03	NA	**-1.03**	0.91	0.26	**1.00**	**1.00**	6	6_153	NA	NA	NA	NA	NA	153	NA
**hsa_SNORD2**					0.01	-0.06	-0.14	-0.06					-0.06	NA	-1.04	NA	**-1.04**	0.91	0.19	**1.00**	**1.00**	6	6_154	NA	NA	NA	NA	NA	154	NA
**hsa-miR-516b**					0.01	-0.09	-0.08	-0.05					-0.05	NA	-1.04	NA	**-1.04**	0.91	0.21	**1.00**	**1.00**	6	6_155	NA	NA	NA	NA	NA	155	NA
**hsa-miR-33b***					-0.30	-0.01	-0.04	-0.12					-0.12	NA	-1.08	NA	**-1.08**	0.91	0.12	**1.00**	**1.00**	6	6_156	NA	NA	NA	NA	NA	156	NA
**miRPlus_17925**					-0.16	-0.14	0.01	-0.10					-0.10	NA	-1.07	NA	**-1.07**	0.93	0.10	**1.00**	**1.00**	6	6_157	NA	NA	NA	NA	NA	157	NA
**miRPlus_17892**					0.01	-0.13	-0.06	-0.06					-0.06	NA	-1.04	NA	**-1.04**	0.93	0.14	**1.00**	**1.00**	6	6_158	NA	NA	NA	NA	NA	158	NA
**hsa-miR-593***					-0.26	-0.02	-0.03	-0.11					-0.11	NA	-1.08	NA	**-1.08**	0.93	0.14	**1.00**	**1.00**	6	6_159	NA	NA	NA	NA	NA	159	NA
**hsa_SNORD13**					-0.08	-0.02	-0.16	-0.09					-0.09	NA	-1.06	NA	**-1.06**	0.93	0.15	**1.00**	**1.00**	6	6_160	NA	NA	NA	NA	NA	160	NA
**hsa-miR-99a***					-0.09	-0.13	-0.01	-0.08					-0.08	NA	-1.05	NA	**-1.05**	0.94	0.14	**1.00**	**1.00**	6	6_161	NA	NA	NA	NA	NA	161	NA
**hsa-miR-199b-5p**					-0.06	-0.09	-0.03	-0.06					-0.06	NA	-1.04	NA	**-1.04**	0.94	0.20	**1.00**	**1.00**	6	6_162	NA	NA	NA	NA	NA	162	NA
**hsa-miR-490-5p**					-0.06	-0.07	-0.05	-0.06					-0.06	NA	-1.04	NA	**-1.04**	0.94	0.21	**1.00**	**1.00**	6	6_163	NA	NA	NA	NA	NA	163	NA
**hsa-miR-654-3p**					-0.10	-0.04	-0.05	-0.07					-0.07	NA	-1.05	NA	**-1.05**	0.95	0.20	**1.00**	**1.00**	6	6_164	NA	NA	NA	NA	NA	164	NA
**hsa-miR-148a***					-0.17	-0.06	-0.02	-0.08					-0.08	NA	-1.06	NA	**-1.06**	0.95	0.17	**1.00**	**1.00**	6	6_165	NA	NA	NA	NA	NA	165	NA
**hsa-miR-200b***					-0.01	-0.07	-0.13	-0.07					-0.07	NA	-1.05	NA	**-1.05**	0.95	0.15	**1.00**	**1.00**	6	6_166	NA	NA	NA	NA	NA	166	NA
**hsa-miR-625***					-0.28	-0.04	-0.03	-0.12					-0.12	NA	-1.08	NA	**-1.08**	0.95	0.11	**1.00**	**1.00**	6	6_167	NA	NA	NA	NA	NA	167	NA
**hsa-miR-541***					-0.01	-0.15	-0.06	-0.07					-0.07	NA	-1.05	NA	**-1.05**	0.95	0.10	**1.00**	**1.00**	6	6_168	NA	NA	NA	NA	NA	168	NA

**ID**	log2ratio_Agilent_1	log2ratio_Agilent_2	log2ratio_Agilent_3	avg_log2ratio_Agilent	log2ratio_Exiqon_1	log2ratio_Exiqon_2	log2ratio_Exiqon_3	avg_log2ratio_Exiqon	log2ratio_Illumina_1	log2ratio_Illumina_2	log2ratio_Illumina_3	avg_log2ratio_Illumina	avg_log2ratio_all	avg_FoldChange_Agilent	avg_FoldChange_Exiqon	avg_FoldChange_Illumina	**avg_FoldChange_all**	p.value.up	p.value.down	**adjusted.p.value.up**	**adjusted.p.value.down**	Index	Index_Rank	RPrank.1	RPrank.2	RPrank.3	RPrank.4	RPrank.5	RPrank.6	RPrank.7
**miRPlus_17899**					-0.01	-0.12	-0.13	-0.08					-0.08	NA	-1.06	NA	**-1.06**	0.95	0.11	**1.00**	**1.00**	6	6_169	NA	NA	NA	NA	NA	169	NA
**miRPlus_17933**					-0.07	-0.13	-0.11	-0.10					-0.10	NA	-1.08	NA	**-1.08**	0.98	0.06	**1.00**	**1.00**	6	6_170	NA	NA	NA	NA	NA	170	NA
**miRPlus_28993**					-0.09	-0.09	-0.16	-0.12					-0.12	NA	-1.08	NA	**-1.08**	0.99	0.06	**1.00**	**1.00**	6	6_171	NA	NA	NA	NA	NA	171	NA
**miRPlus_17951**					-0.24	-0.08	-0.16	-0.16					-0.16	NA	-1.12	NA	**-1.12**	0.99	0.04	**1.00**	**1.00**	6	6_172	NA	NA	NA	NA	NA	172	NA
**miRPlus_27560**					-0.36	-0.05	-0.27	-0.22					-0.22	NA	-1.17	NA	**-1.17**	0.99	0.01	**1.00**	**1.00**	6	6_173	NA	NA	NA	NA	NA	173	NA
**U6-snRNA-2**					-0.33	-0.06	-0.19	-0.19					-0.19	NA	-1.14	NA	**-1.14**	0.99	0.02	**1.00**	**1.00**	6	6_174	NA	NA	NA	NA	NA	174	NA
**miRPlus_28232**					-0.25	-0.08	-0.21	-0.18					-0.18	NA	-1.14	NA	**-1.14**	0.99	0.02	**1.00**	**1.00**	6	6_175	NA	NA	NA	NA	NA	175	NA
**miRPlus_17832**					-0.25	-0.08	-0.29	-0.20					-0.20	NA	-1.15	NA	**-1.15**	1.00	0.01	**1.00**	**1.00**	6	6_176	NA	NA	NA	NA	NA	176	NA
**U6-snRNA-1**					-0.26	-0.12	-0.10	-0.16					-0.16	NA	-1.12	NA	**-1.12**	1.00	0.02	**1.00**	**1.00**	6	6_177	NA	NA	NA	NA	NA	177	NA
**miRPlus_28575**					-0.16	-0.21	-0.14	-0.17					-0.17	NA	-1.12	NA	**-1.12**	1.00	0.02	**1.00**	**1.00**	6	6_178	NA	NA	NA	NA	NA	178	NA
**miRPlus_27564**					-0.28	-0.10	-0.35	-0.25					-0.25	NA	-1.19	NA	**-1.19**	1.00	0.01	**1.00**	**1.00**	6	6_179	NA	NA	NA	NA	NA	179	NA
**miRPlus_17952**					-0.20	-0.22	-0.25	-0.22					-0.22	NA	-1.17	NA	**-1.17**	1.00	0.00	**1.00**	**1.00**	6	6_180	NA	NA	NA	NA	NA	180	NA
**hsa_SNORD3@**					-0.21	-0.16	-0.27	-0.21					-0.21	NA	-1.16	NA	**-1.16**	1.00	0.00	**1.00**	**1.00**	6	6_181	NA	NA	NA	NA	NA	181	NA
**miRPlus_28431**					-0.33	-0.24	-0.43	-0.33					-0.33	NA	-1.26	NA	**-1.26**	1.00	0.00	**1.00**	**0.30**	6	6_182	NA	NA	NA	NA	NA	182	NA
**hsa-miR-30b***					-0.34	-0.28	-0.36	-0.33					-0.33	NA	-1.25	NA	**-1.25**	1.00	0.00	**1.00**	**0.26**	6	6_183	NA	NA	NA	NA	NA	183	NA
**miRPlus_21472**					-0.71	-0.43	-0.68	-0.61					-0.61	NA	-1.52	NA	**-1.52**	1.00	0.00	**1.00**	**0.04**	6	6_184	NA	NA	NA	NA	NA	184	NA
**miRPlus_27561**					-0.83	-0.58	-0.82	-0.74					-0.74	NA	-1.68	NA	**-1.68**	1.00	0.00	**1.00**	**0.00**	6	6_185	NA	NA	NA	NA	NA	185	NA
**miRPlus_17890**					-1.23	-0.80	-1.12	-1.05					-1.05	NA	-2.07	NA	**-2.07**	1.00	0.00	**1.00**	**0.00**	6	6_186	NA	NA	NA	NA	NA	186	NA
**hsa-miR-516a-5p**									3.61			3.61	3.61	NA	NA	12.17	**12.17**	0.03	0.62	**1.00**	**1.00**	7	7_1	NA	NA	NA	NA	NA	NA	1
**hsa-miR-548k**									2.90		3.27	3.08	3.08	NA	NA	8.47	**8.47**	0.03	0.57	**1.00**	**1.00**	7	7_2.5	NA	NA	NA	NA	NA	NA	2.5
**hsa-miR-1255a**									4.08		2.07	3.07	3.07	NA	NA	8.42	**8.42**	0.03	0.57	**1.00**	**1.00**	7	7_2.5	NA	NA	NA	NA	NA	NA	2.5
**hsa-miR-1301**									4.32	-3.08	4.28	1.84	1.84	NA	NA	3.58	**3.58**	0.03	0.32	**1.00**	**1.00**	7	7_4	NA	NA	NA	NA	NA	NA	4
**hsa-miR-548e**									3.48	2.40	2.25	2.71	2.71	NA	NA	6.55	**6.55**	0.04	0.56	**1.00**	**1.00**	7	7_5	NA	NA	NA	NA	NA	NA	5
**hsa-miR-1285**									2.01	2.40		2.21	2.21	NA	NA	4.61	**4.61**	0.05	0.59	**1.00**	**1.00**	7	7_6	NA	NA	NA	NA	NA	NA	6
**hsa-miR-1255b**									2.84			2.84	2.84	NA	NA	7.17	**7.17**	0.07	0.61	**1.00**	**1.00**	7	7_7	NA	NA	NA	NA	NA	NA	7
**hsa-miR-128**									1.93	1.29	2.37	1.86	1.86	NA	NA	3.64	**3.64**	0.08	0.53	**1.00**	**1.00**	7	7_8	NA	NA	NA	NA	NA	NA	8
**hsa-miR-1277**									2.31			2.31	2.31	NA	NA	4.96	**4.96**	0.09	0.61	**1.00**	**1.00**	7	7_9	NA	NA	NA	NA	NA	NA	9
**hsa-miR-1287**									0.74		1.58	1.16	1.16	NA	NA	2.23	**2.23**	0.13	0.51	**1.00**	**1.00**	7	7_10	NA	NA	NA	NA	NA	NA	10
**hsa-miR-320a**									-0.96	2.62	-5.62	-1.32	-1.32	NA	NA	-2.50	**-2.50**	0.13	0.13	**1.00**	**1.00**	7	7_11	NA	NA	NA	NA	NA	NA	11
**hsa-miR-1207-5p**										1.12		1.12	1.12	NA	NA	2.17	**2.17**	0.16	0.47	**1.00**	**1.00**	7	7_12	NA	NA	NA	NA	NA	NA	12
**hsa-miR-1253**										1.02		1.02	1.02	NA	NA	2.02	**2.02**	0.17	0.46	**1.00**	**1.00**	7	7_13	NA	NA	NA	NA	NA	NA	13
**hsa-miR-1271**									-0.21	1.60		0.70	0.70	NA	NA	1.62	**1.62**	0.18	0.48	**1.00**	**1.00**	7	7_14	NA	NA	NA	NA	NA	NA	14
**hsa-miR-548n**									0.68			0.68	0.68	NA	NA	1.60	**1.60**	0.19	0.58	**1.00**	**1.00**	7	7_15	NA	NA	NA	NA	NA	NA	15
**hsa-miR-1261**									-0.31	0.35	1.83	0.63	0.63	NA	NA	1.54	**1.54**	0.20	0.41	**1.00**	**1.00**	7	7_16	NA	NA	NA	NA	NA	NA	16
**hsa-miR-450b-5p**											0.46	0.46	0.46	NA	NA	1.38	**1.38**	0.21	0.31	**1.00**	**1.00**	7	7_17.5	NA	NA	NA	NA	NA	NA	17.5
**hsa-miR-1278**									0.64			0.64	0.64	NA	NA	1.56	**1.56**	0.21	0.57	**1.00**	**1.00**	7	7_17.5	NA	NA	NA	NA	NA	NA	17.5

ID	log2ratio_Agilent_1	log2ratio_Agilent_2	log2ratio_Agilent_3	avg_log2ratio_Agilent	log2ratio_Exiqon_1	log2ratio_Exiqon_2	log2ratio_Exiqon_3	avg_log2ratio_Exiqon	log2ratio_Illumina_1	log2ratio_Illumina_2	log2ratio_Illumina_3	avg_log2ratio_Illumina	avg_log2ratio_all	avg_FoldChange_Agilent	avg_FoldChange_Exiqon	avg_FoldChange_Illumina	**avg_FoldChange_all**	p.value.up	p.value.down	**adjusted.p.value.up**	**adjusted.p.value.down**	Index	Index_Rank	RPrank.1	RPrank.2	RPrank.3	RPrank.4	RPrank.5	RPrank.6	RPrank.7
**hsa-miR-1259**									-0.64	1.37		0.36	0.36	NA	NA	1.29	**1.29**	0.22	0.45	**1.00**	**1.00**	7	7_19	NA	NA	NA	NA	NA	NA	19
**hsa-miR-1262**											0.29	0.29	0.29	NA	NA	1.23	**1.23**	0.22	0.30	**1.00**	**1.00**	7	7_20.5	NA	NA	NA	NA	NA	NA	20.5
**hsa-miR-1291**										0.81		0.81	0.81	NA	NA	1.75	**1.75**	0.22	0.41	**1.00**	**1.00**	7	7_20.5	NA	NA	NA	NA	NA	NA	20.5
**hsa-miR-320c**									-0.28	1.94	-3.23	-0.52	-0.52	NA	NA	-1.44	**-1.44**	0.23	0.21	**1.00**	**1.00**	7	7_22	NA	NA	NA	NA	NA	NA	22
**hsa-miR-140-3p**									0.55	0.01	0.72	0.43	0.43	NA	NA	1.34	**1.34**	0.25	0.39	**1.00**	**1.00**	7	7_23	NA	NA	NA	NA	NA	NA	23
**hsa-miR-296-3p**									1.19	0.20	-0.44	0.32	0.32	NA	NA	1.25	**1.25**	0.25	0.34	**1.00**	**1.00**	7	7_24	NA	NA	NA	NA	NA	NA	24
**hsa-miR-1308**									0.09	-0.30	0.80	0.20	0.20	NA	NA	1.15	**1.15**	0.25	0.37	**1.00**	**1.00**	7	7_25	NA	NA	NA	NA	NA	NA	25
**hsa-miR-320b**									-1.84	2.11	-5.12	-1.62	-1.62	NA	NA	-3.07	**-3.07**	0.25	0.14	**1.00**	**1.00**	7	7_26	NA	NA	NA	NA	NA	NA	26
**hsa-miR-548c-5p**									0.44			0.44	0.44	NA	NA	1.35	**1.35**	0.26	0.54	**1.00**	**1.00**	7	7_27	NA	NA	NA	NA	NA	NA	27
**hsa-miR-1250**									0.94		-1.91	-0.48	-0.48	NA	NA	-1.40	**-1.40**	0.27	0.22	**1.00**	**1.00**	7	7_28	NA	NA	NA	NA	NA	NA	28
**hsa-miR-219-1-3p**									0.27			0.27	0.27	NA	NA	1.20	**1.20**	0.28	0.53	**1.00**	**1.00**	7	7_29	NA	NA	NA	NA	NA	NA	29
**hsa-miR-720**									-2.05	0.89	0.77	-0.13	-0.13	NA	NA	-1.10	**-1.10**	0.28	0.32	**1.00**	**1.00**	7	7_30	NA	NA	NA	NA	NA	NA	30
**hsa-miR-320d**									-1.47	1.93	-2.17	-0.57	-0.57	NA	NA	-1.48	**-1.48**	0.28	0.20	**1.00**	**1.00**	7	7_31	NA	NA	NA	NA	NA	NA	31
**hsa-miR-933**										0.19		0.19	0.19	NA	NA	1.14	**1.14**	0.31	0.33	**1.00**	**1.00**	7	7_32	NA	NA	NA	NA	NA	NA	32
**hsa-miR-1275**									-1.08	0.77	0.01	-0.10	-0.10	NA	NA	-1.07	**-1.07**	0.32	0.32	**1.00**	**1.00**	7	7_33	NA	NA	NA	NA	NA	NA	33
**hsa-miR-873**									0.57	-1.04		-0.24	-0.24	NA	NA	-1.18	**-1.18**	0.32	0.34	**1.00**	**1.00**	7	7_34	NA	NA	NA	NA	NA	NA	34
**hsa-miR-219-2-3p**									0.03			0.03	0.03	NA	NA	1.02	**1.02**	0.33	0.49	**1.00**	**1.00**	7	7_35	NA	NA	NA	NA	NA	NA	35
**hsa-miR-923**									-0.86	0.97	-1.14	-0.34	-0.34	NA	NA	-1.27	**-1.27**	0.33	0.27	**1.00**	**1.00**	7	7_36	NA	NA	NA	NA	NA	NA	36
**hsa-miR-532-3p**									-0.06			-0.06	-0.06	NA	NA	-1.04	**-1.04**	0.35	0.47	**1.00**	**1.00**	7	7_37.5	NA	NA	NA	NA	NA	NA	37.5
**hsa-miR-576-5p**											-0.41	-0.41	-0.41	NA	NA	-1.33	**-1.33**	0.35	0.17	**1.00**	**1.00**	7	7_37.5	NA	NA	NA	NA	NA	NA	37.5
**hsa-miR-1274b**									-3.39	-0.81	1.24	-0.99	-0.99	NA	NA	-1.98	**-1.98**	0.35	0.18	**1.00**	**1.00**	7	7_39	NA	NA	NA	NA	NA	NA	39
**hsa-miR-1246**									-4.11	1.17	-1.48	-1.47	-1.47	NA	NA	-2.77	**-2.77**	0.37	0.11	**1.00**	**1.00**	7	7_40	NA	NA	NA	NA	NA	NA	40
**hsa-miR-1260**									-0.10	-0.81	-0.78	-0.56	-0.56	NA	NA	-1.48	**-1.48**	0.39	0.25	**1.00**	**1.00**	7	7_41	NA	NA	NA	NA	NA	NA	41
**hsa-miR-1266**									0.05	-1.05		-0.50	-0.50	NA	NA	-1.41	**-1.41**	0.39	0.29	**1.00**	**1.00**	7	7_42	NA	NA	NA	NA	NA	NA	42
**hsa-miR-941**									-3.33	-4.77	1.42	-2.22	-2.22	NA	NA	-4.67	**-4.67**	0.39	0.06	**1.00**	**1.00**	7	7_43	NA	NA	NA	NA	NA	NA	43
**hsa-miR-1307**									-5.01	-0.14	0.53	-1.54	-1.54	NA	NA	-2.91	**-2.91**	0.39	0.09	**1.00**	**1.00**	7	7_44	NA	NA	NA	NA	NA	NA	44
**hsa-miR-1290**									-2.39	0.29	-0.97	-1.02	-1.02	NA	NA	-2.03	**-2.03**	0.42	0.18	**1.00**	**1.00**	7	7_45	NA	NA	NA	NA	NA	NA	45
**hsa-miR-877**									-1.76	0.12	-1.78	-1.14	-1.14	NA	NA	-2.20	**-2.20**	0.44	0.18	**1.00**	**1.00**	7	7_46	NA	NA	NA	NA	NA	NA	46
**hsa-miR-139-3p**									-1.28	-0.37		-0.82	-0.82	NA	NA	-1.77	**-1.77**	0.45	0.27	**1.00**	**1.00**	7	7_47	NA	NA	NA	NA	NA	NA	47
**hsa-miR-1274a**									-2.91	-1.92	0.20	-1.54	-1.54	NA	NA	-2.91	**-2.91**	0.45	0.15	**1.00**	**1.00**	7	7_48	NA	NA	NA	NA	NA	NA	48
**hsa-miR-1268**									-1.68	-2.32	-0.39	-1.46	-1.46	NA	NA	-2.75	**-2.75**	0.45	0.18	**1.00**	**1.00**	7	7_49	NA	NA	NA	NA	NA	NA	49
**hsa-miR-1303**									-3.15	-0.85	-0.21	-1.40	-1.40	NA	NA	-2.64	**-2.64**	0.47	0.15	**1.00**	**1.00**	7	7_50	NA	NA	NA	NA	NA	NA	50
**hsa-miR-1254**									-2.10	-3.79	-0.07	-1.99	-1.99	NA	NA	-3.97	**-3.97**	0.48	0.10	**1.00**	**1.00**	7	7_51	NA	NA	NA	NA	NA	NA	51
**hsa-miR-1224-5p**										-1.46		-1.46	-1.46	NA	NA	-2.75	**-2.75**	0.49	0.14	**1.00**	**1.00**	7	7_52.5	NA	NA	NA	NA	NA	NA	52.5
**hsa-miR-1304**									-1.11			-1.11	-1.11	NA	NA	-2.16	**-2.16**	0.49	0.33	**1.00**	**1.00**	7	7_52.5	NA	NA	NA	NA	NA	NA	52.5
**hsa-miR-615-5p**									-4.55	-4.72	0.20	-3.02	-3.02	NA	NA	-8.14	**-8.14**	0.50	0.05	**1.00**	**1.00**	7	7_54	NA	NA	NA	NA	NA	NA	54

ID	log2ratio_Agilent_1	log2ratio_Agilent_2	log2ratio_Agilent_3	avg_log2ratio_Agilent	log2ratio_Exiqon_1	log2ratio_Exiqon_2	log2ratio_Exiqon_3	avg_log2ratio_Exiqon	log2ratio_Illumina_1	log2ratio_Illumina_2	log2ratio_Illumina_3	avg_log2ratio_Illumina	avg_log2ratio_all	avg_FoldChange_Agilent	avg_FoldChange_Exiqon	avg_FoldChange_Illumina	**avg_FoldChange_all**	p.value.up	p.value.down	**adjusted.p.value.up**	**adjusted.p.value.down**	Index	Index_Rank	RPrank.1	RPrank.2	RPrank.3	RPrank.4	RPrank.5	RPrank.6	RPrank.7
**hsa-miR-1273**									-1.18			-1.18	-1.18	NA	NA	-2.26	**-2.26**	0.50	0.31	**1.00**	**1.00**	7	7_55	NA	NA	NA	NA	NA	NA	55
**hsa-miR-935**										-2.35		-2.35	-2.35	NA	NA	-5.09	**-5.09**	0.53	0.09	**1.00**	**1.00**	7	7_56	NA	NA	NA	NA	NA	NA	56
**hsa-miR-874**										-3.17		-3.17	-3.17	NA	NA	-9.00	**-9.00**	0.56	0.04	**1.00**	**1.00**	7	7_57.5	NA	NA	NA	NA	NA	NA	57.5
**hsa-miR-1827**									-1.84			-1.84	-1.84	NA	NA	-3.57	**-3.57**	0.56	0.23	**1.00**	**1.00**	7	7_57.5	NA	NA	NA	NA	NA	NA	57.5
**hsa-miR-1180**									-2.52	-2.61		-2.57	-2.57	NA	NA	-5.93	**-5.93**	0.57	0.10	**1.00**	**1.00**	7	7_59	NA	NA	NA	NA	NA	NA	59
**hsa-miR-1826**									-2.09			-2.09	-2.09	NA	NA	-4.24	**-4.24**	0.58	0.17	**1.00**	**1.00**	7	7_60	NA	NA	NA	NA	NA	NA	60
**hsa-miR-1292**									-3.42			-3.42	-3.42	NA	NA	-10.69	**-10.69**	0.62	0.04	**1.00**	**1.00**	7	7_61	NA	NA	NA	NA	NA	NA	61
**hsa-miR-671-3p**														NA	NA	NA	**NA**	0.63	0.63	**1.00**	**1.00**	7	7_62	NA	NA	NA	NA	NA	NA	62
**hsa-miR-1276**														NA	NA	NA	**NA**	0.63	0.63	**1.00**	**1.00**	7	7_63	NA	NA	NA	NA	NA	NA	63
**hsa-miR-548o**														NA	NA	NA	**NA**	0.63	0.63	**1.00**	**1.00**	7	7_64	NA	NA	NA	NA	NA	NA	64
**hsa-miR-129-3p**														NA	NA	NA	**NA**	0.63	0.63	**1.00**	**1.00**	7	7_65	NA	NA	NA	NA	NA	NA	65
**hsa-miR-942**														NA	NA	NA	**NA**	0.63	0.63	**1.00**	**1.00**	7	7_66	NA	NA	NA	NA	NA	NA	66
**hsa-miR-548d-5p**														NA	NA	NA	**NA**	0.63	0.63	**1.00**	**1.00**	7	7_67	NA	NA	NA	NA	NA	NA	67
**hsa-miR-1286**														NA	NA	NA	**NA**	0.63	0.63	**1.00**	**1.00**	7	7_68	NA	NA	NA	NA	NA	NA	68
**hsa-miR-1283**														NA	NA	NA	**NA**	0.63	0.63	**1.00**	**1.00**	7	7_69	NA	NA	NA	NA	NA	NA	69
**hsa-miR-548j**														NA	NA	NA	**NA**	0.63	0.63	**1.00**	**1.00**	7	7_70	NA	NA	NA	NA	NA	NA	70
**hsa-miR-1258**														NA	NA	NA	**NA**	0.63	0.63	**1.00**	**1.00**	7	7_71	NA	NA	NA	NA	NA	NA	71
**hsa-miR-342-5p**														NA	NA	NA	**NA**	0.63	0.63	**1.00**	**1.00**	7	7_72	NA	NA	NA	NA	NA	NA	72
**hsa-miR-1293**														NA	NA	NA	**NA**	0.63	0.63	**1.00**	**1.00**	7	7_73	NA	NA	NA	NA	NA	NA	73
**hsa-miR-1296**														NA	NA	NA	**NA**	0.63	0.63	**1.00**	**1.00**	7	7_74	NA	NA	NA	NA	NA	NA	74
**hsa-miR-556-3p**														NA	NA	NA	**NA**	0.63	0.63	**1.00**	**1.00**	7	7_75	NA	NA	NA	NA	NA	NA	75
**hsa-miR-664**														NA	NA	NA	**NA**	0.63	0.63	**1.00**	**1.00**	7	7_76	NA	NA	NA	NA	NA	NA	76
**hsa-miR-1284**														NA	NA	NA	**NA**	0.63	0.63	**1.00**	**1.00**	7	7_77	NA	NA	NA	NA	NA	NA	77
**hsa-miR-1294**														NA	NA	NA	**NA**	0.63	0.63	**1.00**	**1.00**	7	7_78	NA	NA	NA	NA	NA	NA	78
**hsa-miR-1256**														NA	NA	NA	**NA**	0.63	0.63	**1.00**	**1.00**	7	7_79	NA	NA	NA	NA	NA	NA	79
**hsa-miR-548p**														NA	NA	NA	**NA**	0.63	0.63	**1.00**	**1.00**	7	7_80	NA	NA	NA	NA	NA	NA	80
**hsa-miR-1257**														NA	NA	NA	**NA**	0.63	0.63	**1.00**	**1.00**	7	7_81	NA	NA	NA	NA	NA	NA	81
**hsa-miR-1289**														NA	NA	NA	**NA**	0.63	0.63	**1.00**	**1.00**	7	7_82	NA	NA	NA	NA	NA	NA	82
**hsa-miR-1228**														NA	NA	NA	**NA**	0.63	0.63	**1.00**	**1.00**	7	7_83	NA	NA	NA	NA	NA	NA	83
**hsa-miR-548b-5p**														NA	NA	NA	**NA**	0.63	0.63	**1.00**	**1.00**	7	7_84	NA	NA	NA	NA	NA	NA	84
**hsa-miR-1299**														NA	NA	NA	**NA**	0.63	0.63	**1.00**	**1.00**	7	7_85	NA	NA	NA	NA	NA	NA	85
**hsa-miR-885-3p**														NA	NA	NA	**NA**	0.63	0.63	**1.00**	**1.00**	7	7_86	NA	NA	NA	NA	NA	NA	86
**hsa-miR-548l**														NA	NA	NA	**NA**	0.63	0.63	**1.00**	**1.00**	7	7_87	NA	NA	NA	NA	NA	NA	87
**hsa-miR-939**														NA	NA	NA	**NA**	0.63	0.63	**1.00**	**1.00**	7	7_88	NA	NA	NA	NA	NA	NA	88
**hsa-miR-937**														NA	NA	NA	**NA**	0.63	0.63	**1.00**	**1.00**	7	7_89	NA	NA	NA	NA	NA	NA	89
**hsa-miR-1323**														NA	NA	NA	**NA**	0.63	0.63	**1.00**	**1.00**	7	7_90	NA	NA	NA	NA	NA	NA	90

**ID**	log2ratio_Agilent_1	log2ratio_Agilent_2	log2ratio_Agilent_3	avg_log2ratio_Agilent	log2ratio_Exiqon_1	log2ratio_Exiqon_2	log2ratio_Exiqon_3	avg_log2ratio_Exiqon	log2ratio_Illumina_1	log2ratio_Illumina_2	log2ratio_Illumina_3	avg_log2ratio_Illumina	avg_log2ratio_all	avg_FoldChange_Agilent	avg_FoldChange_Exiqon	avg_FoldChange_Illumina	**avg_FoldChange_all**	p.value.up	p.value.down	**adjusted.p.value.up**	**adjusted.p.value.down**	Index	Index_Rank	RPrank.1	RPrank.2	RPrank.3	RPrank.4	RPrank.5	RPrank.6	RPrank.7
**hsa-miR-1272**														NA	NA	NA	**NA**	0.63	0.63	**1.00**	**1.00**	7	7_91	NA	NA	NA	NA	NA	NA	91
**hsa-miR-450b-3p**														NA	NA	NA	**NA**	0.63	0.63	**1.00**	**1.00**	7	7_92	NA	NA	NA	NA	NA	NA	92
**hsa-miR-298**														NA	NA	NA	**NA**	0.63	0.63	**1.00**	**1.00**	7	7_93	NA	NA	NA	NA	NA	NA	93
**hsa-miR-1226**														NA	NA	NA	**NA**	0.63	0.63	**1.00**	**1.00**	7	7_94	NA	NA	NA	NA	NA	NA	94
**hsa-miR-146b-3p**														NA	NA	NA	**NA**	0.63	0.63	**1.00**	**1.00**	7	7_95	NA	NA	NA	NA	NA	NA	95
**hsa-miR-1181**														NA	NA	NA	**NA**	0.63	0.63	**1.00**	**1.00**	7	7_96	NA	NA	NA	NA	NA	NA	96
**hsa-miR-1282**														NA	NA	NA	**NA**	0.63	0.63	**1.00**	**1.00**	7	7_97	NA	NA	NA	NA	NA	NA	97
**hsa-miR-220c**														NA	NA	NA	**NA**	0.63	0.63	**1.00**	**1.00**	7	7_98	NA	NA	NA	NA	NA	NA	98
**hsa-miR-1249**														NA	NA	NA	**NA**	0.63	0.63	**1.00**	**1.00**	7	7_99	NA	NA	NA	NA	NA	NA	99
**hsa-miR-1306**														NA	NA	NA	**NA**	0.63	0.63	**1.00**	**1.00**	7	7_100	NA	NA	NA	NA	NA	NA	100
**hsa-miR-1183**														NA	NA	NA	**NA**	0.63	0.63	**1.00**	**1.00**	7	7_101	NA	NA	NA	NA	NA	NA	101
**hsa-miR-1233**														NA	NA	NA	**NA**	0.63	0.63	**1.00**	**1.00**	7	7_102	NA	NA	NA	NA	NA	NA	102
**hsa-miR-1179**														NA	NA	NA	**NA**	0.63	0.63	**1.00**	**1.00**	7	7_103	NA	NA	NA	NA	NA	NA	103
**hsa-miR-1247**														NA	NA	NA	**NA**	0.63	0.63	**1.00**	**1.00**	7	7_104	NA	NA	NA	NA	NA	NA	104
**hsa-miR-1288**														NA	NA	NA	**NA**	0.63	0.63	**1.00**	**1.00**	7	7_105	NA	NA	NA	NA	NA	NA	105
**hsa-miR-1237**														NA	NA	NA	**NA**	0.63	0.63	**1.00**	**1.00**	7	7_106	NA	NA	NA	NA	NA	NA	106
**hsa-miR-1281**														NA	NA	NA	**NA**	0.63	0.63	**1.00**	**1.00**	7	7_107	NA	NA	NA	NA	NA	NA	107
**hsa-miR-548g**														NA	NA	NA	**NA**	0.63	0.63	**1.00**	**1.00**	7	7_108	NA	NA	NA	NA	NA	NA	108
**hsa-miR-1295**														NA	NA	NA	**NA**	0.63	0.63	**1.00**	**1.00**	7	7_109	NA	NA	NA	NA	NA	NA	109
**hsa-miR-1229**														NA	NA	NA	**NA**	0.63	0.63	**1.00**	**1.00**	7	7_110	NA	NA	NA	NA	NA	NA	110
**hsa-miR-1267**														NA	NA	NA	**NA**	0.63	0.63	**1.00**	**1.00**	7	7_111	NA	NA	NA	NA	NA	NA	111
**hsa-miR-1300**														NA	NA	NA	**NA**	0.63	0.63	**1.00**	**1.00**	7	7_112	NA	NA	NA	NA	NA	NA	112
**hsa-miR-34c-3p**														NA	NA	NA	**NA**	0.63	0.63	**1.00**	**1.00**	7	7_113	NA	NA	NA	NA	NA	NA	113
**hsa-miR-548h**														NA	NA	NA	**NA**	0.63	0.63	**1.00**	**1.00**	7	7_114	NA	NA	NA	NA	NA	NA	114
**hsa-miR-1269**														NA	NA	NA	**NA**	0.63	0.63	**1.00**	**1.00**	7	7_115	NA	NA	NA	NA	NA	NA	115
**hsa-miR-323-3p**														NA	NA	NA	**NA**	0.63	0.63	**1.00**	**1.00**	7	7_116	NA	NA	NA	NA	NA	NA	116
**hsa-miR-1185**														NA	NA	NA	**NA**	0.63	0.63	**1.00**	**1.00**	7	7_117	NA	NA	NA	NA	NA	NA	117
**hsa-miR-513a-3p**														NA	NA	NA	**NA**	0.63	0.63	**1.00**	**1.00**	7	7_118	NA	NA	NA	NA	NA	NA	118
**hsa-miR-1265**														NA	NA	NA	**NA**	0.63	0.63	**1.00**	**1.00**	7	7_119	NA	NA	NA	NA	NA	NA	119
**hsa-miR-1248**														NA	NA	NA	**NA**	0.63	0.63	**1.00**	**1.00**	7	7_120	NA	NA	NA	NA	NA	NA	120
**hsa-miR-708**														NA	NA	NA	**NA**	0.63	0.63	**1.00**	**1.00**	7	7_121	NA	NA	NA	NA	NA	NA	121
**hsa-miR-1245**														NA	NA	NA	**NA**	0.63	0.63	**1.00**	**1.00**	7	7_122	NA	NA	NA	NA	NA	NA	122
**hsa-miR-663b**														NA	NA	NA	**NA**	0.63	0.63	**1.00**	**1.00**	7	7_123	NA	NA	NA	NA	NA	NA	123
**hsa-miR-1231**														NA	NA	NA	**NA**	0.63	0.63	**1.00**	**1.00**	7	7_124	NA	NA	NA	NA	NA	NA	124
**hsa-miR-548i**														NA	NA	NA	**NA**	0.63	0.63	**1.00**	**1.00**	7	7_125	NA	NA	NA	NA	NA	NA	125
**hsa-miR-1200**														NA	NA	NA	**NA**	0.63	0.63	**1.00**	**1.00**	7	7_126	NA	NA	NA	NA	NA	NA	126

ID	log2ratio_Agilent_1	log2ratio_Agilent_2	log2ratio_Agilent_3	avg_log2ratio_Agilent	log2ratio_Exiqon_1	log2ratio_Exiqon_2	log2ratio_Exiqon_3	avg_log2ratio_Exiqon	log2ratio_Illumina_1	log2ratio_Illumina_2	log2ratio_Illumina_3	avg_log2ratio_Illumina	avg_log2ratio_all	avg_FoldChange_Agilent	avg_FoldChange_Exiqon	avg_FoldChange_Illumina	**avg_FoldChange_all**	p.value.up	p.value.down	**adjusted.p.value.up**	**adjusted.p.value.down**	Index	Index_Rank	RPrank.1	RPrank.2	RPrank.3	RPrank.4	RPrank.5	RPrank.6	RPrank.7
**hsa-miR-520c-3p**														NA	NA	NA	**NA**	0.63	0.63	**1.00**	**1.00**	7	7_127	NA	NA	NA	NA	NA	NA	127
**hsa-miR-1201**														NA	NA	NA	**NA**	0.63	0.63	**1.00**	**1.00**	7	7_128	NA	NA	NA	NA	NA	NA	128
**hsa-miR-1184**														NA	NA	NA	**NA**	0.63	0.63	**1.00**	**1.00**	7	7_129	NA	NA	NA	NA	NA	NA	129
**hsa-miR-199a-3p**														NA	NA	NA	**NA**	0.63	0.63	**1.00**	**1.00**	7	7_130	NA	NA	NA	NA	NA	NA	130
**hsa-miR-208b**														NA	NA	NA	**NA**	0.63	0.63	**1.00**	**1.00**	7	7_131	NA	NA	NA	NA	NA	NA	131
**hsa-miR-1203**														NA	NA	NA	**NA**	0.63	0.63	**1.00**	**1.00**	7	7_132	NA	NA	NA	NA	NA	NA	132
**hsa-miR-889**														NA	NA	NA	**NA**	0.63	0.63	**1.00**	**1.00**	7	7_133	NA	NA	NA	NA	NA	NA	133
**hsa-miR-1305**														NA	NA	NA	**NA**	0.63	0.63	**1.00**	**1.00**	7	7_134	NA	NA	NA	NA	NA	NA	134
**hsa-miR-1298**														NA	NA	NA	**NA**	0.63	0.63	**1.00**	**1.00**	7	7_135	NA	NA	NA	NA	NA	NA	135
**hsa-miR-1238**														NA	NA	NA	**NA**	0.63	0.63	**1.00**	**1.00**	7	7_136	NA	NA	NA	NA	NA	NA	136
**hsa-miR-1182**														NA	NA	NA	**NA**	0.63	0.63	**1.00**	**1.00**	7	7_137	NA	NA	NA	NA	NA	NA	137
**hsa-miR-1205**														NA	NA	NA	**NA**	0.63	0.63	**1.00**	**1.00**	7	7_138	NA	NA	NA	NA	NA	NA	138
**hsa-miR-1244**														NA	NA	NA	**NA**	0.63	0.63	**1.00**	**1.00**	7	7_139	NA	NA	NA	NA	NA	NA	139
**hsa-miR-1202**														NA	NA	NA	**NA**	0.63	0.63	**1.00**	**1.00**	7	7_140	NA	NA	NA	NA	NA	NA	140
**hsa-miR-1270**														NA	NA	NA	**NA**	0.63	0.63	**1.00**	**1.00**	7	7_141	NA	NA	NA	NA	NA	NA	141
**hsa-miR-208a**														NA	NA	NA	**NA**	0.63	0.63	**1.00**	**1.00**	7	7_142	NA	NA	NA	NA	NA	NA	142
**hsa-miR-944**														NA	NA	NA	**NA**	0.63	0.63	**1.00**	**1.00**	7	7_143	NA	NA	NA	NA	NA	NA	143
**hsa-miR-127-5p**														NA	NA	NA	**NA**	0.63	0.63	**1.00**	**1.00**	7	7_144	NA	NA	NA	NA	NA	NA	144
**hsa-miR-1825**														NA	NA	NA	**NA**	0.63	0.63	**1.00**	**1.00**	7	7_145	NA	NA	NA	NA	NA	NA	145
**hsa-miR-943**														NA	NA	NA	**NA**	0.63	0.63	**1.00**	**1.00**	7	7_146	NA	NA	NA	NA	NA	NA	146
**hsa-miR-1208**														NA	NA	NA	**NA**	0.63	0.63	**1.00**	**1.00**	7	7_147	NA	NA	NA	NA	NA	NA	147
