# Supplementary material for: Microarray and deep sequencing cross-platform analysis of the mirRNome and isomiR variation in response to epidermal growth factor
Source: BMC Genomics. 2013 Jun 1;14:371. doi: 10.1186/1471-2164-14-371 (PMC3680220; doi:10.1186/1471-2164-14-371)
Supplement: Additional file 9 — List of 234 genes predicted in common by TS5.0 miRvana 3.0 and PICTar to be targets of the 8 miRNAs found to be regulated by EGF in this work. [file 1471-2164-14-371-S9.pdf]

Common elements in "TS 5.0", "miRvana 3.0" and "PiCTar":

ABCB6
ADAMTS6
ADAMTS7
ANGPTL2
ANKHD1
ANKRD13B
ANP32A
APBB2
ARF4
ARFGEF1
ARID1A
ARID4A
ARL4C
ASPN
ASXL1
ATP2B2
BLCAP
BMF
BNC2
BOLL
BRD3
BTG2
CALU
CAMK1D
CAMK2D
CASKIN1
CCDC64
CCL1
CCNT2
CDKN1B
CDKN1C
CHD3
CHD7
CKAP4
CNN3
CNOT8
CNTFR
COL15A1
COL1A2
COL2A1
COL3A1
COL4A1
COL4A5
COL5A3
COL6A3
COL7A1
CORO7
CPSF6
CSDA

CTCF
CTNNBIP1
CYR61
DBT
DCP2
DCUN1D1
DEDD
DGKH
DMRT3
DNAJA2
DNMT3A
DNMT3B
DYNLT1
EIF4A2
EIF4E2
ELF2
ELOVL4
EML5
ENTPD7
FAM83A
FASLG
FGA
FKBP2
FMR1
FNDC3A
FOS
FOXA1
FOXJ2
FUSIP1
GAL3ST3
GARNL1
GLI3
GLIS2
GLTSCR1
GNAI2
GPR37
GTDC1
HAS3
HBEGF
HBP1
HECTD2
HELLS
HERPUD2
HIF3A
HIPK1
HMGA2
HOXC13
IFI30
IGF2BP2
INA

IPO11
IREB2
IRF2
IRX5
ISL1
ITGA6
JAG1
KBTBD2
KCNJ12
KCNK2
KCTD3
KHDRBS2
KHDRBS3
KIAA1267
KLF4
KRIT1
LASP1
LEMD3
LPL
LRRFIP1
LSM11
MAP4K5
MATN2
MBD2
MEIS1
MESDC1
MMP16
MYBL2
NASP
NAV3
NFIB
NID2
NIPBL
NLK
NPAS4
NR4A2
NTF3
NXT2
OLFM1
OLIG3
OSBPL5
OTUD4
PAIP2
PAK1
PAN3
PBX3
PC
PCDHA10
PCDHA2
PCDHA3

PCDHA4
PCSK6
PDCD4
PDE4D
PDE7B
PDGFC
PEA15
PELI1
PHF17
PHF20L1
PITPNM2
PITX2
PKP4
PLP2
PMP22
POLE4
POM121
PPIC
PPP1R3D
PPP2R2A
PPP6C
PTCH1
RAB2A
RAB30
RASA1
RB1
RBM24
RECK
RGS4
RLF
RMND5A
RNF122
RSBN1
RSBN1L
SESTD1
SETDB1
SFRS3
SH2D1A
SLC16A7
SMYD5
SNCA
SNCB
SOX2
SPG20
SPRY1
SPRY2
SSH2
STAG2
STRN4
SUV420H2

TACC1
TACC2
TAGAP
TCF7L1
TESK2
TFAP2C
TFEB
TGFBI
TIMM9
TJAP1
TLL1
TLN2
TMEFF1
TMPRSS11F
TNFRSF1A
TOX
TP53INP1
TRAF4
TRPS1
TSC22D3
TSPAN14
VDAC1
VGLL4
VPS26A
WDR47
WWP1
XKR6
YAP1
YWHAE
ZBTB5
ZFP36L1
ZFPM2
ZNF238
ZNF362
ZNF644
