# Supplementary material for: Microarray and deep sequencing cross-platform analysis of the mirRNome and isomiR variation in response to epidermal growth factor
Source: BMC Genomics. 2013 Jun 1;14:371. doi: 10.1186/1471-2164-14-371 (PMC3680220; doi:10.1186/1471-2164-14-371)
Supplement: Additional file 10 — Summary of isomiR counts for top EGF regulated miRNAs. [file 1471-2164-14-371-S10.pdf]

							Frequency						pvalue to be a real variant						Contribution to the total amount for the corresponding miRNA					
seq	len	mir	tr5	tr3	ad	mut	con1	con2	con3	egf1	egf2	egf3	zs_con1	zs_con2	zs_con3	zs_egf1	zs_egf2	zs_egf3	cont_con1	cont_con2	con_con3	con_egf1	con_egf2	con_egf3
TAACAGTCTACAGCCATGGTCG	22	hsa-miR-132	0	0	0	0	4	NA	NA	49	NA	68	0	NA	NA	0	NA	0	100	NA	NA	34.3	NA	17.7
AACAGTCTACAGCCATGGTCG	21	hsa-miR-132	tT	0	0	0	NA	NA	NA	10	NA	10	NA	NA	NA	0	NA	0	NA	NA	NA	7	NA	2.6
AACAGTCTACAGCCATGGTCGT	22	hsa-miR-132	tT	0	qT	0	NA	NA	NA	2	NA	17	NA	NA	NA	0	NA	0	NA	NA	NA	1.4	NA	4.4
ACAGTCTACAGCCATGGTCGT	21	hsa-miR-132	tTA	0	qT	0	NA	NA	NA	3	NA	6	NA	NA	NA	0	NA	0	NA	NA	NA	2.1	NA	1.6
ACAGTCTACAGCCATGGTCGTC	22	hsa-miR-132	tTA	0	qTC	0	NA	NA	NA	4	NA	7	NA	NA	NA	0	NA	0	NA	NA	NA	2.8	NA	1.8
ACAGTCTACAGCCATGGTCGTT	22	hsa-miR-132	tTA	0	qTT	0	NA	NA	NA	5	NA	81	NA	NA	NA	0	NA	0	NA	NA	NA	3.5	NA	21
TAACAGTCTACAGCCATGGTCGT	23	hsa-miR-132	0	0	qT	0	NA	NA	NA	61	NA	148	NA	NA	NA	0	NA	0	NA	NA	NA	42.7	NA	38.4
TAACAGTCTACAGCCATGGTCGTT	24	hsa-miR-132	0	0	qTT	0	NA	NA	NA	7	NA	16	NA	NA	NA	0	NA	0	NA	NA	NA	4.9	NA	4.2
TAACAGTCTACATCCATGGTCGT	23	hsa-miR-132	0	0	qT	13TG	NA	NA	NA	2	NA	2	NA	NA	NA	1	NA	0	NA	NA	NA	1.4	NA	0.5
ACAGGCTACAGCCATGGTCGTT	22	hsa-miR-132	tTA	0	qTT	7GT	NA	NA	NA	NA	NA	6	NA	NA	NA	NA	NA	0	NA	NA	NA	NA	NA	1.6
ACAGTCTACAGCCATGGTTGTT	22	hsa-miR-132	tTA	0	qTT	21TC	NA	NA	NA	NA	NA	2	NA	NA	NA	NA	NA	0	NA	NA	NA	NA	NA	0.5
TAACAGTCTACAGCCATGGGCG	22	hsa-miR-132	0	0	0	20GT	NA	NA	NA	NA	NA	3	NA	NA	NA	NA	NA	0	NA	NA	NA	NA	NA	0.8
TAACAGTCTACAGCCATGGGCGT	23	hsa-miR-132	0	0	qT	20GT	NA	NA	NA	NA	NA	4	NA	NA	NA	NA	NA	0	NA	NA	NA	NA	NA	1
TAACAGTCTACAGCCATGGTAG	22	hsa-miR-132	0	0	0	21AC	NA	NA	NA	NA	NA	2	NA	NA	NA	NA	NA	0	NA	NA	NA	NA	NA	0.5
TAACAGTCTACAGCCATGGTC	21	hsa-miR-132	0	tG	0	0	NA	NA	NA	NA	NA	2	NA	NA	NA	NA	NA	0	NA	NA	NA	NA	NA	0.5
TAACAGTCTACAGCCATGGTCGAA	24	hsa-miR-132	0	0	qAA	0	NA	NA	NA	NA	NA	2	NA	NA	NA	NA	NA	0	NA	NA	NA	NA	NA	0.5
TAACAGTCTACAGCCATGGTCT	22	hsa-miR-132	0	tG	qT	0	NA	NA	NA	NA	NA	3	NA	NA	NA	NA	NA	0	NA	NA	NA	NA	NA	0.8
TAACAGTCTACAGCCATGGTCTT	23	hsa-miR-132	0	tG	qTT	0	NA	NA	NA	NA	NA	2	NA	NA	NA	NA	NA	0	NA	NA	NA	NA	NA	0.5
TAACAGTCTACAGCCATGTTCGT	23	hsa-miR-132	0	0	qT	19TG	NA	NA	NA	NA	NA	2	NA	NA	NA	NA	NA	0	NA	NA	NA	NA	NA	0.5
TAACAGTCTACAGTCATGGTCGT	23	hsa-miR-132	0	0	qT	14TC	NA	NA	NA	NA	NA	2	NA	NA	NA	NA	NA	0	NA	NA	NA	NA	NA	0.5
AGCTACATTGGCTGCTGGGTTT	22	hsa-miR-221	0	tC	0	11GT	3	15	26	NA	52	363	1	0	0	NA	0	0	0.8	0.6	0.8	NA	0.6	0.4
AGCTACATTGTCTGCTGGGGTT	22	hsa-miR-221	0	tC	0	20GT	17	33	63	NA	84	602	0	0	0	NA	0	0	4.5	1.4	1.9	NA	1	0.7
AGCTACATTGTCTGCTGGGGTTC	23	hsa-miR-221	0	0	0	20GT	5	18	55	NA	79	410	0	0	0	NA	0	0	1.3	0.8	1.7	NA	0.9	0.5
AGCTACATTGTCTGCTGGGT	20	hsa-miR-221	0	tTTC	0	0	2	NA	NA	NA	NA	274	0	NA	NA	NA	NA	0	0.5	NA	NA	NA	NA	0.3
AGCTACATTGTCTGCTGGGTT	21	hsa-miR-221	0	tTC	0	0	10	48	61	NA	113	2457	0	0	0	NA	0	0	2.7	2	1.8	NA	1.4	2.8
AGCTACATTGTCTGCTGGGTTT	22	hsa-miR-221	0	tC	0	0	122	821	1023	NA	2876	32595	0	0	0	NA	0	0	32.6	34.8	30.8	NA	34.5	37.2
AGCTACATTGTCTGCTGGGTTTA	23	hsa-miR-221	0	tC	qA	0	8	62	74	NA	159	1608	0	0	0	NA	0	0	2.1	2.6	2.2	NA	1.9	1.8
AGCTACATTGTCTGCTGGGTTTC	23	hsa-miR-221	0	0	0	0	120	786	1090	NA	3327	29882	0	0	0	NA	0	0	32.1	33.3	32.8	NA	39.9	34.1
AGCTACATTGTCTGCTGGGTTTCA	24	hsa-miR-221	0	qA	0	0	4	29	42	NA	98	876	0	0	0	NA	0	0	1.1	1.2	1.3	NA	1.2	1
AGCTACATTGTCTGCTGGGTTTCT	24	hsa-miR-221	0	0	qT	0	17	35	57	NA	105	863	0	0	0	NA	0	0	4.5	1.5	1.7	NA	1.3	1
AGCTACATTGTCTGCTGGGTTTT	23	hsa-miR-221	0	tC	qT	0	31	200	213	NA	549	5494	0	0	0	NA	0	0	8.3	8.5	6.4	NA	6.6	6.3
AGCTACATTGTCTGCTGGGTTTTT	24	hsa-miR-221	0	tC	qTT	0	3	18	16	NA	17	298	0	0	0	NA	0	0	0.8	0.8	0.5	NA	0.2	0.3
AGCTACATTGTCTGCTGTGTTT	22	hsa-miR-221	0	tC	0	18TG	4	NA	12	NA	NA	572	0	NA	0	NA	NA	0	1.1	NA	0.4	NA	NA	0.7
AGCTACATTGTCTGCTGTGTTTC	23	hsa-miR-221	0	0	0	18TG	6	NA	9	NA	18	504	0	NA	0	NA	0	0	1.6	NA	0.3	NA	0.2	0.6
AGCTACATTGTCTGCTGTGTTTT	23	hsa-miR-221	0	tC	qT	18TG	2	NA	NA	NA	NA	204	0	NA	NA	NA	NA	0	0.5	NA	NA	NA	NA	0.2
AGCTACATTGTCTGCTTGGTT	21	hsa-miR-221	0	tTC	0	17TG	2	NA	NA	NA	NA	NA	0	NA	NA	NA	NA	NA	0.5	NA	NA	NA	NA	NA
AGCTACATTGTCTGCTTGGTTT	22	hsa-miR-221	0	tC	0	17TG	9	NA	NA	NA	22	924	0	NA	NA	NA	0	0	2.4	NA	NA	NA	0.3	1.1
AGCTACATTGTCTGCTTGGTTTC	23	hsa-miR-221	0	0	0	17TG	9	12	14	NA	23	909	0	0	0	NA	0	0	2.4	0.5	0.4	NA	0.3	1
AGATACATTGTCTGCTGGGTTT	22	hsa-miR-221	0	tC	0	3AC	NA	8	13	NA	29	177	NA	1	1	NA	1	1	NA	0.3	0.4	NA	0.3	0.2
AGCGACATTGTCTGCTGGGTTT	22	hsa-miR-221	0	tC	0	4GT	NA	12	19	NA	21	NA	NA	0	0	NA	0	NA	NA	0.5	0.6	NA	0.3	NA
AGCTACAGTGTCTGCTGGGTTT	22	hsa-miR-221	0	tC	0	8GT	NA	6	28	NA	23	NA	NA	0	0	NA	0	NA	NA	0.3	0.8	NA	0.3	NA
AGCTACAGTGTCTGCTGGGTTTC	23	hsa-miR-221	0	0	0	8GT	NA	9	26	NA	20	NA	NA	0	0	NA	0	NA	NA	0.4	0.8	NA	0.2	NA
AGCTACATTGGCTGCTGGGTTTC	23	hsa-miR-221	0	0	0	11GT	NA	11	28	NA	34	319	NA	0	0	NA	0	0	NA	0.5	0.8	NA	0.4	0.4
AGCTACATTGTATGCTGGGTTT	22	hsa-miR-221	0	tC	0	12AC	NA	8	NA	NA	16	209	NA	0	NA	NA	0	0	NA	0.3	NA	NA	0.2	0.2
AGCTACATTGTCTGCTGGGGTTT	23	hsa-miR-221	0	tC	qT	20GT	NA	18	21	NA	33	NA	NA	0	0	NA	0	NA	NA	0.8	0.6	NA	0.4	NA
AGCTACATTGTCTGCTGGGTTA	22	hsa-miR-221	0	tTC	qA	0	NA	15	20	NA	NA	539	NA	0	0	NA	NA	0	NA	0.6	0.6	NA	NA	0.6
AGCTACATTGTCTGCTGGGTTTAA	24	hsa-miR-221	0	tC	qAA	0	NA	6	NA	NA	NA	NA	NA	0	NA	NA	NA	NA	NA	0.3	NA	NA	NA	NA
AGCTACATTGTCTGCTGGGTTTTA	24	hsa-miR-221	0	tC	qTA	0	NA	5	NA	NA	NA	NA	NA	0	NA	NA	NA	NA	NA	0.2	NA	NA	NA	NA
AGCTACATTGTCTGGTGGGTTT	22	hsa-miR-221	0	tC	0	15GC	NA	16	17	NA	56	271	NA	0	0	NA	0	0	NA	0.7	0.5	NA	0.7	0.3
AGCTACATTGTCTGGTGGGTTTC	23	hsa-miR-221	0	0	0	15GC	NA	8	14	NA	17	NA	NA	0	0	NA	0	NA	NA	0.3	0.4	NA	0.2	NA
AGCTACATTGTCTGGTGGGTTTT	23	hsa-miR-221	0	tC	qT	15GC	NA	13	11	NA	27	NA	NA	0	0	NA	0	NA	NA	0.6	0.3	NA	0.3	NA
AGCTACATTGTCTGTTGGGTTT	22	hsa-miR-221	0	tC	0	15TC	NA	7	NA	NA	NA	NA	NA	0	NA	NA	NA	NA	NA	0.3	NA	NA	NA	NA
AGCTACATTGTCTGTTGGGTTTC	23	hsa-miR-221	0	0	0	15TC	NA	6	NA	NA	NA	NA	NA	0	NA	NA	NA	NA	NA	0.3	NA	NA	NA	NA
AGCTACATTGTGTGCTGGGTTT	22	hsa-miR-221	0	tC	0	12GC	NA	12	NA	NA	32	NA	NA	0	NA	NA	0	NA	NA	0.5	NA	NA	0.4	NA
AGCTACATTGTGTGCTGGGTTTC	23	hsa-miR-221	0	0	0	12GC	NA	6	NA	NA	NA	NA	NA	0	NA	NA	NA	NA	NA	0.3	NA	NA	NA	NA
AGCTACATTGTGTGCTGGGTTTT	23	hsa-miR-221	0	tC	qT	12GC	NA	7	NA	NA	16	NA	NA	0	NA	NA	0	NA	NA	0.3	NA	NA	0.2	NA
AGCGACATTGTCTGCTGGGTTTC	23	hsa-miR-221	0	0	0	4GT	NA	NA	14	NA	16	NA	NA	NA	0	NA	0	NA	NA	NA	0.4	NA	0.2	NA
AGCTACAGTGTCTGCTGGGTTTT	23	hsa-miR-221	0	tC	qT	8GT	NA	NA	11	NA	NA	NA	NA	NA	0	NA	NA	NA	NA	NA	0.3	NA	NA	NA
AGCTACATTGGCTGCTGGGTTTT	23	hsa-miR-221	0	tC	qT	11GT	NA	NA	9	NA	15	NA	NA	NA	0	NA	0	NA	NA	NA	0.3	NA	0.2	NA
AGCTACATTGTCTGCTGGGTTTAT	24	hsa-miR-221	0	tC	qAT	0	NA	NA	11	NA	NA	191	NA	NA	0	NA	NA	0	NA	NA	0.3	NA	NA	0.2
CGCTACATTGTCTGCTGGGTTT	22	hsa-miR-221	0	tC	0	1CA	NA	NA	47	NA	28	264	NA	NA	0	NA	0	0	NA	NA	1.4	NA	0.3	0.3
CGCTACATTGTCTGCTGGGTTTC	23	hsa-miR-221	0	0	0	1CA	NA	NA	44	5	39	244	NA	NA	0	0	0	0	NA	NA	1.3	4.4	0.5	0.3
ATCTACATTGTCTGCTGGGTT	21	hsa-miR-221	0	tTC	0	2TG	NA	NA	NA	3	NA	NA	NA	NA	NA	1	NA	NA	NA	NA	NA	2.7	NA	NA
ATCTACATTGTCTGCTGGGTTT	22	hsa-miR-221	0	tC	0	2TG	NA	NA	NA	27	NA	NA	NA	NA	NA	0	NA	NA	NA	NA	NA	23.9	NA	NA
ATCTACATTGTCTGCTGGGTTTC	23	hsa-miR-221	0	0	0	2TG	NA	NA	NA	34	NA	NA	NA	NA	NA	0	NA	NA	NA	NA	NA	30.1	NA	NA
ATCTACATTGTCTGCTGGGTTTCT	24	hsa-miR-221	0	0	qT	2TG	NA	NA	NA	7	NA	NA	NA	NA	NA	0	NA	NA	NA	NA	NA	6.2	NA	NA
ATCTACATTGTCTGCTGGGTTTT	23	hsa-miR-221	0	tC	qT	2TG	NA	NA	NA	3	NA	NA	NA	NA	NA	0	NA	NA	NA	NA	NA	2.7	NA	NA
CAGCTACATTGTCTGCTGGGTTTC	24	hsa-miR-221	qC	0	0	0	NA	NA	NA	2	NA	NA	NA	NA	NA	0	NA	NA	NA	NA	NA	1.8	NA	NA
GCTACATTGTCTGCTGGGTTT	21	hsa-miR-221	tA	tC	0	0	NA	NA	NA	7	NA	165	NA	NA	NA	0	NA	0	NA	NA	NA	6.2	NA	0.2
GCTACATTGTCTGCTGGGTTTC	22	hsa-miR-221	tA	0	0	0	NA	NA	NA	3	NA	NA	NA	NA	NA	0	NA	NA	NA	NA	NA	2.7	NA	NA
GCTACATTGTCTGCTGGGTTTT	22	hsa-miR-221	tA	tC	qT	0	NA	NA	NA	2	NA	NA	NA	NA	NA	0	NA	NA	NA	NA	NA	1.8	NA	NA
GCTACATTGTCTGCTTGGTTTC	22	hsa-miR-221	tA	0	0	17TG	NA	NA	NA	2	NA	NA	NA	NA	NA	0	NA	NA	NA	NA	NA	1.8	NA	NA
GGCTACATTGTCTGCTGGGTTT	22	hsa-miR-221	0	tC	0	1GA	NA	NA	NA	3	NA	NA	NA	NA	NA	0	NA	NA	NA	NA	NA	2.7	NA	NA
GGCTACATTGTCTGCTGGGTTTC	23	hsa-miR-221	0	0	0	1GA	NA	NA	NA	4	NA	NA	NA	NA	NA	0	NA	NA	NA	NA	NA	3.5	NA	NA
TGCTACATTGTCTGCTGGGTTT	22	hsa-miR-221	0	tC	0	1TA	NA	NA	NA	6	NA	NA	NA	NA	NA	0	NA	NA	NA	NA	NA	5.3	NA	NA
TGCTACATTGTCTGCTGGGTTTC	23	hsa-miR-221	0	0	0	1TA	NA	NA	NA	3	NA	NA	NA	NA	NA	0	NA	NA	NA	NA	NA	2.7	NA	NA
TGCTACATTGTCTGCTGGGTTTT	23	hsa-miR-221	0	tC	qT	1TA	NA	NA	NA	2	NA	NA	NA	NA	NA	0	NA	NA	NA	NA	NA	1.8	NA	NA
AGCTACATTGTCTGCTGGGTTTCAA	25	hsa-miR-221	0	qA	qA	0	NA	NA	NA	NA	NA	179	NA	NA	NA	NA	NA	0	NA	NA	NA	NA	NA	0.2
AGCTACATTGTCTGCTGGTTTT	22	hsa-miR-221	0	tC	0	19TG	NA	NA	NA	NA	NA	280	NA	NA	NA	NA	NA	0	NA	NA	NA	NA	NA	0.3
AGCTACATTGTCTGCTGGTTTTC	23	hsa-miR-221	0	0	0	19TG	NA	NA	NA	NA	NA	328	NA	NA	NA	NA	NA	0	NA	NA	NA	NA	NA	0.4
AGCTACATTGTCTGCTTGGTTTT	23	hsa-miR-221	0	tC	qT	17TG	NA	NA	NA	NA	NA	208	NA	NA	NA	NA	NA	0	NA	NA	NA	NA	NA	0.2

AGCTACAGCTGGCTACTGGG	20	hsa-miR-222	0	tT	0	8GT	2	NA	NA	NA	NA	NA	1	NA	NA	NA	NA	NA	0.9	NA	NA	NA	NA	NA
AGCTACAGCTGGCTACTGGGTCT	23	hsa-miR-222	0	qCT	0	8GT	3	6	11	28	11	44	0	0	0	0	0	0	1.4	1	1.1	0.3	0.6	0.2
AGCTACATCTGGCTACTGG	19	hsa-miR-222	0	tGT	0	0	2	4	NA	NA	NA	56	0	0	NA	NA	NA	0	0.9	0.6	NA	NA	NA	0.3
AGCTACATCTGGCTACTGGG	20	hsa-miR-222	0	tT	0	0	60	16	15	61	16	103	0	0	0	0	0	0	27.6	2.6	1.5	0.7	0.8	0.5
AGCTACATCTGGCTACTGGGT	21	hsa-miR-222	0	0	0	0	19	7	19	458	18	576	0	0	0	0	0	0	8.8	1.1	1.9	5.4	0.9	2.8
AGCTACATCTGGCTACTGGGTC	22	hsa-miR-222	0	qC	0	0	13	14	29	491	47	785	0	0	0	0	0	0	6	2.3	2.9	5.8	2.4	3.9
AGCTACATCTGGCTACTGGGTCA	23	hsa-miR-222	0	qC	qA	0	4	4	6	73	4	82	0	0	0	0	0	0	1.8	0.6	0.6	0.9	0.2	0.4
AGCTACATCTGGCTACTGGGTCT	23	hsa-miR-222	0	qCT	0	0	73	182	231	3690	384	5980	0	0	0	0	0	0	33.6	29.4	23.1	43.3	19.6	29.6
AGCTACATCTGGCTACTGGGTCTC	24	hsa-miR-222	0	qCTC	0	0	25	273	445	2327	1189	9252	0	0	0	0	0	0	11.5	44.2	44.5	27.3	60.6	45.7
AGCTACATCTGGCTACTGGGTCTT	24	hsa-miR-222	0	qCT	qT	0	3	48	63	351	60	884	0	0	0	0	0	0	1.4	7.8	6.3	4.1	3.1	4.4
AGCTACATCTGGCTACTGGT	20	hsa-miR-222	0	tGT	qT	0	3	NA	NA	NA	NA	NA	0	NA	NA	NA	NA	NA	1.4	NA	NA	NA	NA	NA
AGCTACATCTGGCTACTGGTT	21	hsa-miR-222	0	0	0	20TG	2	NA	NA	9	NA	NA	0	NA	NA	0	NA	NA	0.9	NA	NA	0.1	NA	NA
AGCTACATCTGGCTACTGGTTCT	23	hsa-miR-222	0	qCT	0	20TG	2	NA	4	136	NA	98	0	NA	0	0	NA	0	0.9	NA	0.4	1.6	NA	0.5
AGCTACATCTGGCTACTGTGTCT	23	hsa-miR-222	0	qCT	0	19TG	2	NA	NA	40	NA	46	0	NA	NA	0	NA	0	0.9	NA	NA	0.5	NA	0.2
AGCTACATCTTGCTACTGGG	20	hsa-miR-222	0	tT	0	11TG	2	NA	NA	NA	NA	NA	0	NA	NA	NA	NA	NA	0.9	NA	NA	NA	NA	NA
AGCTACATCTTGCTACTGGGT	21	hsa-miR-222	0	0	0	11TG	2	NA	NA	NA	NA	NA	0	NA	NA	NA	NA	NA	0.9	NA	NA	NA	NA	NA
AACTACATCTGGCTACTGGGTCTC	24	hsa-miR-222	0	qCTC	0	2AG	NA	2	NA	NA	NA	NA	NA	1	NA	NA	NA	NA	NA	0.3	NA	NA	NA	NA
AGATACATCTGGCTACTGGGTCTC	24	hsa-miR-222	0	qCTC	0	3AC	NA	4	6	NA	9	60	NA	0	0	NA	0	0	NA	0.6	0.6	NA	0.5	0.3
AGCGACATCTGGCTACTGGGTCT	23	hsa-miR-222	0	qCT	0	4GT	NA	3	7	NA	9	NA	NA	0	0	NA	0	NA	NA	0.5	0.7	NA	0.5	NA
AGCGACATCTGGCTACTGGGTCTC	24	hsa-miR-222	0	qCTC	0	4GT	NA	4	17	NA	14	58	NA	0	0	NA	0	0	NA	0.6	1.7	NA	0.7	0.3
AGCTACAGCTGGCTACTGGGTCTC	24	hsa-miR-222	0	qCTC	0	8GT	NA	7	20	22	26	94	NA	0	0	0	0	0	NA	1.1	2	0.3	1.3	0.5
AGCTACAGCTGGCTACTGGGTCTT	24	hsa-miR-222	0	qCT	qT	8GT	NA	2	3	NA	NA	NA	NA	0	0	NA	NA	NA	NA	0.3	0.3	NA	NA	NA
AGCTACATCTGGCTACAGGGTCTC	24	hsa-miR-222	0	qCTC	0	17AT	NA	2	NA	NA	NA	NA	NA	0	NA	NA	NA	NA	NA	0.3	NA	NA	NA	NA
AGCTACATCTGGCTACGGGGTCTC	24	hsa-miR-222	0	qCTC	0	17GT	NA	3	4	NA	NA	NA	NA	0	0	NA	NA	NA	NA	0.5	0.4	NA	NA	NA
AGCTACATCTGGCTACTGGGTCAT	24	hsa-miR-222	0	qC	qAT	0	NA	2	NA	12	NA	NA	NA	0	NA	0	NA	NA	NA	0.3	NA	0.1	NA	NA
AGCTACATCTGGCTACTGGGTCTA	24	hsa-miR-222	0	qCT	qA	0	NA	6	6	61	14	190	NA	0	0	0	0	0	NA	1	0.6	0.7	0.7	0.9
AGCTACATCTGGCTACTGGGTTT	23	hsa-miR-222	0	0	qTT	0	NA	3	9	51	4	90	NA	0	0	0	0	0	NA	0.5	0.9	0.6	0.2	0.4
AGCTACATCTGGCTACTGGTTCTC	24	hsa-miR-222	0	qCTC	0	20TG	NA	2	3	70	11	138	NA	0	0	0	0	0	NA	0.3	0.3	0.8	0.6	0.7
AGCTACATCTGGCTACTGGTTCTT	24	hsa-miR-222	0	qCT	qT	20TG	NA	2	NA	17	NA	NA	NA	0	NA	0	NA	NA	NA	0.3	NA	0.2	NA	NA
AGCTACATCTGGCTATTGGGTCTC	24	hsa-miR-222	0	qCTC	0	16TC	NA	4	NA	NA	NA	NA	NA	0	NA	NA	NA	NA	NA	0.6	NA	NA	NA	NA
AGCTACATCTGGCTATTGGGTTT	23	hsa-miR-222	0	0	qTT	16TC	NA	2	NA	NA	NA	NA	NA	0	NA	NA	NA	NA	NA	0.3	NA	NA	NA	NA
AGCTACATCTGGTTACTGGGTCT	23	hsa-miR-222	0	qCT	0	13TC	NA	2	NA	NA	5	NA	NA	0	NA	NA	0	NA	NA	0.3	NA	NA	0.3	NA
AGCTACATCTGGTTACTGGGTCTC	24	hsa-miR-222	0	qCTC	0	13TC	NA	6	4	NA	7	NA	NA	0	0	NA	0	NA	NA	1	0.4	NA	0.4	NA
AGCTACATCTGGTTACTGGGTCTT	24	hsa-miR-222	0	qCT	qT	13TC	NA	2	NA	NA	NA	NA	NA	0	NA	NA	NA	NA	NA	0.3	NA	NA	NA	NA
AGCTACATCTGTCTACTGGGTCTC	24	hsa-miR-222	0	qCTC	0	12TG	NA	2	3	16	9	45	NA	0	0	0	0	0	NA	0.3	0.3	0.2	0.5	0.2
AGCTTCATCTGGCTACTGGGTCT	23	hsa-miR-222	0	qCT	0	5TA	NA	2	NA	NA	NA	NA	NA	0	NA	NA	NA	NA	NA	0.3	NA	NA	NA	NA
GCTACATCTGGCTACTGGGTCTC	23	hsa-miR-222	tA	qCTC	0	0	NA	2	NA	NA	NA	40	NA	0	NA	NA	NA	0	NA	0.3	NA	NA	NA	0.2
AGATACATCTGGCTACTGGGTCT	23	hsa-miR-222	0	qCT	0	3AC	NA	NA	3	NA	5	NA	NA	NA	1	NA	1	NA	NA	NA	0.3	NA	0.3	NA
AGCTACATCTGGCTACTGCGTCTC	24	hsa-miR-222	0	qCTC	0	19CG	NA	NA	5	NA	NA	NA	NA	NA	0	NA	NA	NA	NA	NA	0.5	NA	NA	NA
AGCTACATCTGGCTACTGTGTCTC	24	hsa-miR-222	0	qCTC	0	19TG	NA	NA	5	36	5	88	NA	NA	0	0	0	0	NA	NA	0.5	0.4	0.3	0.4
AGCTACATCTGGCTACTTGGTCT	23	hsa-miR-222	0	qCT	0	18TG	NA	NA	7	57	NA	52	NA	NA	0	0	NA	0	NA	NA	0.7	0.7	NA	0.3
AGCTACATCTGGCTACTTGGTCTC	24	hsa-miR-222	0	qCTC	0	18TG	NA	NA	11	37	6	78	NA	NA	0	0	0	0	NA	NA	1.1	0.4	0.3	0.4
AGCTACATCTGGCTCCTGGGTCTC	24	hsa-miR-222	0	qCTC	0	15CA	NA	NA	3	NA	NA	73	NA	NA	0	NA	NA	0	NA	NA	0.3	NA	NA	0.4
AGCTACATCTTGCTACTGGGTCTC	24	hsa-miR-222	0	qCTC	0	11TG	NA	NA	3	11	NA	47	NA	NA	0	0	NA	0	NA	NA	0.3	0.1	NA	0.2
AGCTACCTCTGGCTACTGGGTCTC	24	hsa-miR-222	0	qCTC	0	7CA	NA	NA	3	NA	3	NA	NA	NA	0	NA	0	NA	NA	NA	0.3	NA	0.2	NA
CGCTACATCTGGCTACTGGGTCT	23	hsa-miR-222	0	qCT	0	1CA	NA	NA	11	NA	4	NA	NA	NA	0	NA	0	NA	NA	NA	1.1	NA	0.2	NA
CGCTACATCTGGCTACTGGGTCTC	24	hsa-miR-222	0	qCTC	0	1CA	NA	NA	15	NA	15	48	NA	NA	0	NA	0	0	NA	NA	1.5	NA	0.8	0.2
AGCTACATCTGGCTACTGGGTA	22	hsa-miR-222	0	0	qA	0	NA	NA	NA	10	NA	59	NA	NA	NA	0	NA	0	NA	NA	NA	0.1	NA	0.3
AGCTACATCTGGCTACTGGGTAA	23	hsa-miR-222	0	0	qAA	0	NA	NA	NA	15	NA	45	NA	NA	NA	0	NA	0	NA	NA	NA	0.2	NA	0.2
AGCTACATCTGGCTACTGGGTAT	23	hsa-miR-222	0	0	qAT	0	NA	NA	NA	29	NA	62	NA	NA	NA	0	NA	0	NA	NA	NA	0.3	NA	0.3
AGCTACATCTGGCTACTGGGTT	22	hsa-miR-222	0	0	qT	0	NA	NA	NA	34	NA	51	NA	NA	NA	0	NA	0	NA	NA	NA	0.4	NA	0.3
AGCTACATCTGTCTACTGGGTCT	23	hsa-miR-222	0	qCT	0	12TG	NA	NA	NA	24	3	NA	NA	NA	NA	0	0	NA	NA	NA	NA	0.3	0.2	NA
AGCTACATCTTGCTACTGGGTCT	23	hsa-miR-222	0	qCT	0	11TG	NA	NA	NA	16	NA	NA	NA	NA	NA	0	NA	NA	NA	NA	NA	0.2	NA	NA
AGCTACATTTGGCTACTGGGTCT	23	hsa-miR-222	0	qCT	0	9TC	NA	NA	NA	14	NA	NA	NA	NA	NA	0	NA	NA	NA	NA	NA	0.2	NA	NA
AGCTACATTTGGCTACTGGGTCTC	24	hsa-miR-222	0	qCTC	0	9TC	NA	NA	NA	10	6	NA	NA	NA	NA	0	0	NA	NA	NA	NA	0.1	0.3	NA
AGCTACATCTGGCTACTGTGTCTT	24	hsa-miR-222	0	qCT	qT	19TG	NA	NA	NA	NA	3	NA	NA	NA	NA	NA	0	NA	NA	NA	NA	NA	0.2	NA
AGCTACATCTGGCTATTGGGTCT	23	hsa-miR-222	0	qCT	0	16TC	NA	NA	NA	NA	5	NA	NA	NA	NA	NA	0	NA	NA	NA	NA	NA	0.3	NA
AGCTACATCTGGTTACTGGGTTT	23	hsa-miR-222	0	0	qTT	13TC	NA	NA	NA	NA	3	NA	NA	NA	NA	NA	0	NA	NA	NA	NA	NA	0.2	NA
AGCTATATCTGGCTACTGGGTCTC	24	hsa-miR-222	0	qCTC	0	6TC	NA	NA	NA	NA	3	NA	NA	NA	NA	NA	0	NA	NA	NA	NA	NA	0.2	NA
AGCTACATCTGGCTCCTGGGTCT	23	hsa-miR-222	0	qCT	0	15CA	NA	NA	NA	NA	NA	44	NA	NA	NA	NA	NA	0	NA	NA	NA	NA	NA	0.2
CTAGCACCATCTGAAATCGGT	21	hsa-miR-29a	qC	tTA	0	0	3	NA	NA	NA	NA	NA	0	NA	NA	NA	NA	NA	0.1	NA	NA	NA	NA	NA
CTAGCACCATCTGAAATCGGTT	22	hsa-miR-29a	qC	tA	0	0	43	11	7	758	11	341	0	0	0	0	0	0	1.3	1.2	0.8	1	0.3	0.4
CTAGCACCATCTGAAATCGGTTA	23	hsa-miR-29a	qC	0	0	0	60	7	13	2106	60	1297	0	0	0	0	0	0	1.8	0.8	1.5	2.9	1.5	1.6
CTAGCACCATCTGAAATCGGTTT	23	hsa-miR-29a	qC	tA	qT	0	10	2	3	303	10	182	0	0	0	0	0	0	0.3	0.2	0.3	0.4	0.2	0.2
CTAGCACCATCTGAAATCGTTTA	23	hsa-miR-29a	qC	0	0	19TG	5	NA	NA	NA	NA	NA	0	NA	NA	NA	NA	NA	0.1	NA	NA	NA	NA	NA
GCACCATCTGAAATCGGTTA	20	hsa-miR-29a	tTA	0	0	0	3	NA	NA	NA	NA	NA	0	NA	NA	NA	NA	NA	0.1	NA	NA	NA	NA	NA
TAGCAACATCTGAAATCGGTTA	22	hsa-miR-29a	0	0	0	6AC	3	NA	NA	NA	NA	172	0	NA	NA	NA	NA	0	0.1	NA	NA	NA	NA	0.2
TAGCACAATCTGAAATCGGTTA	22	hsa-miR-29a	0	0	0	7AC	6	6	7	NA	12	308	1	1	1	NA	0	0	0.2	0.6	0.8	NA	0.3	0.4
TAGCACCAGCTGAAATCGGTTA	22	hsa-miR-29a	0	0	0	9GT	5	NA	NA	56	5	NA	0	NA	NA	0	0	NA	0.1	NA	NA	0.1	0.1	NA
TAGCACCATCGGAAATCGGTTA	22	hsa-miR-29a	0	0	0	11GT	20	8	14	235	31	288	0	0	0	0	0	0	0.6	0.9	1.6	0.3	0.8	0.4
TAGCACCATCTGAAATCGG	19	hsa-miR-29a	0	tTTA	0	0	4	2	2	179	NA	174	0	0	0	0	NA	0	0.1	0.2	0.2	0.2	NA	0.2
TAGCACCATCTGAAATCGGCTA	22	hsa-miR-29a	0	0	0	20CT	5	NA	NA	86	NA	NA	0	NA	NA	0	NA	NA	0.1	NA	NA	0.1	NA	NA
TAGCACCATCTGAAATCGGGT	21	hsa-miR-29a	0	tA	0	20GT	4	NA	NA	NA	NA	NA	0	NA	NA	NA	NA	NA	0.1	NA	NA	NA	NA	NA
TAGCACCATCTGAAATCGGGTA	22	hsa-miR-29a	0	0	0	20GT	62	7	18	410	36	433	0	0	0	0	0	0	1.8	0.8	2	0.6	0.9	0.5
TAGCACCATCTGAAATCGGT	20	hsa-miR-29a	0	tTA	0	0	10	3	NA	199	4	181	0	0	NA	0	0	0	0.3	0.3	NA	0.3	0.1	0.2
TAGCACCATCTGAAATCGGTT	21	hsa-miR-29a	0	tA	0	0	192	27	32	2399	82	2133	0	0	0	0	0	0	5.7	2.9	3.6	3.3	2	2.6
TAGCACCATCTGAAATCGGTTA	22	hsa-miR-29a	0	0	0	0	2734	783	688	60713	3519	66383	0	0	0	0	0	0	80.5	84.7	77.7	82.7	86.6	82.1
TAGCACCATCTGAAATCGGTTAA	23	hsa-miR-29a	0	0	qA	0	32	15	3	696	16	553	0	0	0	0	0	0	0.9	1.6	0.3	0.9	0.4	0.7
TAGCACCATCTGAAATCGGTTAT	23	hsa-miR-29a	0	qT	0	0	22	6	6	590	11	386	0	0	0	0	0	0	0.6	0.6	0.7	0.8	0.3	0.5
TAGCACCATCTGAAATCGGTTT	22	hsa-miR-29a	0	tA	qT	0	30	12	6	494	15	669	0	0	0	0	0	0	0.9	1.3	0.7	0.7	0.4	0.8
TAGCACCATCTGAAATCGTTTA	22	hsa-miR-29a	0	0	0	19TG	37	3	NA	547	NA	327	0	0	NA	0	NA	0	1.1	0.3	NA	0.7	NA	0.4
TAGCACCATCTGAAATCTGTTA	22	hsa-miR-29a	0	0	0	18TG	32	NA	NA	355	NA	189	0	NA	NA	0	NA	0	0.9	NA	NA	0.5	NA	0.2
TAGCACCATCTGAACTCGGTTA	22	hsa-miR-29a	0	0	0	15CA	4	NA	5	NA	13	674	0	NA	0	NA	0	0	0.1	NA	0.6	NA	0.3	0.8

TAGCACCATCTGACATCGGTTA	22	hsa-miR-29a	0	0	0	14CA	3	NA	26	56	26	284	0	NA	0	0	0	0	0.1	NA	2.9	0.1	0.6	0.4
TAGCACCATCTGATATCGGTTA	22	hsa-miR-29a	0	0	0	14TA	6	3	4	156	5	NA	0	0	0	0	0	NA	0.2	0.3	0.5	0.2	0.1	NA
TAGCACCATCTGTAATCGGTTA	22	hsa-miR-29a	0	0	0	13TA	4	NA	NA	79	NA	NA	0	NA	NA	0	NA	NA	0.1	NA	NA	0.1	NA	NA
TAGCACCATCTTAAATCGGTTA	22	hsa-miR-29a	0	0	0	12TG	19	NA	NA	187	NA	139	0	NA	NA	0	NA	0	0.6	NA	NA	0.3	NA	0.2
TAGCACTATCTGAAATCGGTTA	22	hsa-miR-29a	0	0	0	7TC	4	NA	3	NA	7	153	0	NA	0	NA	0	0	0.1	NA	0.3	NA	0.2	0.2
TAGCATCATCTGAAATCGGTTA	22	hsa-miR-29a	0	0	0	6TC	3	NA	NA	NA	NA	241	0	NA	NA	NA	NA	0	0.1	NA	NA	NA	NA	0.3
TAGTACCATCTGAAATCGGTTA	22	hsa-miR-29a	0	0	0	4TC	4	NA	NA	92	4	186	0	NA	NA	0	0	0	0.1	NA	NA	0.1	0.1	0.2
TAGCACCATCTGAAATAGGTTA	22	hsa-miR-29a	0	0	0	17AC	NA	8	9	60	26	493	NA	0	0	0	0	0	NA	0.9	1	0.1	0.6	0.6
TAGCACCATCTGAAATCGGTTATAG	25	hsa-miR-29a	0	qT	qAG	0	NA	2	NA	NA	NA	NA	NA	0	NA	NA	NA	NA	NA	0.2	NA	NA	NA	NA
TAGCACCATCTGAAGTCGGTTA	22	hsa-miR-29a	0	0	0	15GA	NA	4	2	NA	4	NA	NA	0	0	NA	0	NA	NA	0.4	0.2	NA	0.1	NA
TAGCACCGTCTGAAATCGGTTA	22	hsa-miR-29a	0	0	0	8GA	NA	2	NA	NA	5	NA	NA	0	NA	NA	0	NA	NA	0.2	NA	NA	0.1	NA
TAGCAGCATCTGAAATCGGTTA	22	hsa-miR-29a	0	0	0	6GC	NA	2	NA	NA	NA	NA	NA	0	NA	NA	NA	NA	NA	0.2	NA	NA	NA	NA
TAGGACCATCTGAAATCGGTTA	22	hsa-miR-29a	0	0	0	4GC	NA	9	10	NA	31	311	NA	0	0	NA	0	0	NA	1	1.1	NA	0.8	0.4
TCGCACCATCTGAAATCGGTTA	22	hsa-miR-29a	0	0	0	2CA	NA	2	NA	NA	NA	NA	NA	0	NA	NA	NA	NA	NA	0.2	NA	NA	NA	NA
CAGCACCATCTGAAATCGGTTA	22	hsa-miR-29a	0	0	0	1CT	NA	NA	2	NA	NA	NA	NA	NA	0	NA	NA	NA	NA	NA	0.2	NA	NA	NA
CGAGCACCATCTGAAATCGGTTA	23	hsa-miR-29a	qC	0	0	1GT	NA	NA	2	NA	NA	NA	NA	NA	0	NA	NA	NA	NA	NA	0.2	NA	NA	NA
CTAGCACCATCGGAAATCGGTTA	23	hsa-miR-29a	qC	0	0	11GT	NA	NA	2	NA	NA	NA	NA	NA	0	NA	NA	NA	NA	NA	0.2	NA	NA	NA
CTAGCACCATCTGAAATCGGTTAT	24	hsa-miR-29a	qC	qT	0	0	NA	NA	2	113	NA	NA	NA	NA	0	0	NA	NA	NA	NA	0.2	0.2	NA	NA
TAGCACCATATGAAATCGGTTA	22	hsa-miR-29a	0	0	0	10AC	NA	NA	6	69	32	NA	NA	NA	0	0	0	NA	NA	NA	0.7	0.1	0.8	NA
TAGCACCATCTGAAATCGGGTT	22	hsa-miR-29a	0	tA	qT	20GT	NA	NA	2	NA	NA	NA	NA	NA	0	NA	NA	NA	NA	NA	0.2	NA	NA	NA
TAGCACCATCTGCAATCGGTTA	22	hsa-miR-29a	0	0	0	13CA	NA	NA	12	60	7	128	NA	NA	0	0	0	0	NA	NA	1.4	0.1	0.2	0.2
TAGAACCATCTGAAATCGGTTA	22	hsa-miR-29a	0	0	0	4AC	NA	NA	NA	64	12	360	NA	NA	NA	0	1	0	NA	NA	NA	0.1	0.3	0.4
TAGCACCATCTGAAATCGGTGA	22	hsa-miR-29a	0	0	0	21GT	NA	NA	NA	51	NA	NA	NA	NA	NA	0	NA	NA	NA	NA	NA	0.1	NA	NA
TAGCACCATCTGAAATCGGTTAAA	24	hsa-miR-29a	0	0	qAA	0	NA	NA	NA	112	NA	NA	NA	NA	NA	0	NA	NA	NA	NA	NA	0.2	NA	NA
TAGCACCATCTGAAATCGGTTC	22	hsa-miR-29a	0	tA	qC	0	NA	NA	NA	55	NA	174	NA	NA	NA	0	NA	0	NA	NA	NA	0.1	NA	0.2
TATCACCATCTGAAATCGGTTA	22	hsa-miR-29a	0	0	0	3TG	NA	NA	NA	66	NA	NA	NA	NA	NA	0	NA	NA	NA	NA	NA	0.1	NA	NA
CTAGCACCATCTGACATCGGTTA	23	hsa-miR-29a	qC	0	0	14CA	NA	NA	NA	NA	3	NA	NA	NA	NA	NA	0	NA	NA	NA	NA	NA	0.1	NA
TAGCACCATCTGAAATCGGTTATTG	25	hsa-miR-29a	0	qT	qTG	0	NA	NA	NA	NA	8	141	NA	NA	NA	NA	0	0	NA	NA	NA	NA	0.2	0.2
TAGCACCATCTGAAATGGGTTA	22	hsa-miR-29a	0	0	0	17GC	NA	NA	NA	NA	6	NA	NA	NA	NA	NA	0	NA	NA	NA	NA	NA	0.1	NA
TAGCACGATCTGAAATCGGTTA	22	hsa-miR-29a	0	0	0	7GC	NA	NA	NA	NA	3	NA	NA	NA	NA	NA	0	NA	NA	NA	NA	NA	0.1	NA
TGGCACCATCTGAAATCGGTTA	22	hsa-miR-29a	0	0	0	2GA	NA	NA	NA	NA	5	NA	NA	NA	NA	NA	0	NA	NA	NA	NA	NA	0.1	NA
AGCACCATCTGAAATCGGTTA	21	hsa-miR-29a	tT	0	0	0	NA	NA	NA	NA	NA	238	NA	NA	NA	NA	NA	0	NA	NA	NA	NA	NA	0.3
TAGCACCATCTGAAATTGGTTA	22	hsa-miR-29a	0	0	0	17TC	NA	NA	NA	NA	NA	129	NA	NA	NA	NA	NA	0	NA	NA	NA	NA	NA	0.2
TAGCACCATTTGAAATCAGGGTT	23	hsa-miR-29b	0	0	0	20GT	2	NA	NA	NA	NA	11	1	NA	NA	NA	NA	0	1.1	NA	NA	NA	NA	0.4
TAGCACCATTTGAAATCAGTGT	22	hsa-miR-29b	0	tT	0	0	18	4	3	58	28	172	0	0	0	0	0	0	10	6.6	2.8	5	5.7	6.2
TAGCACCATTTGAAATCAGTGTA	23	hsa-miR-29b	0	tT	qA	0	3	NA	NA	7	3	6	0	NA	NA	0	0	0	1.7	NA	NA	0.6	0.6	0.2
TAGCACCATTTGAAATCAGTGTT	23	hsa-miR-29b	0	0	0	0	148	53	94	929	411	2070	0	0	0	0	0	0	82.2	86.9	88.7	80.2	83	74.8
TAGCACCATTTGAAATCAGTGTTT	24	hsa-miR-29b	0	qT	0	0	4	2	2	68	12	108	0	0	0	0	0	0	2.2	3.3	1.9	5.9	2.4	3.9
TAGCACCATTTGAAATCATTGTT	23	hsa-miR-29b	0	0	0	19TG	3	NA	NA	2	NA	4	0	NA	NA	0	NA	0	1.7	NA	NA	0.2	NA	0.1
TAGCATCATTTGAAATCAGTGTT	23	hsa-miR-29b	0	0	0	6TC	2	NA	NA	4	NA	15	0	NA	NA	0	NA	0	1.1	NA	NA	0.3	NA	0.5
TCGCACCATTTGAAATCAGTGTT	23	hsa-miR-29b	0	0	0	2CA	NA	2	NA	NA	NA	5	NA	1	NA	NA	NA	0	NA	3.3	NA	NA	NA	0.2
TAGCACCATTGGAAATCAGTGTT	23	hsa-miR-29b	0	0	0	11GT	NA	NA	3	9	3	11	NA	NA	1	0	0	0	NA	NA	2.8	0.8	0.6	0.4
TAGCACCATTTGACATCAGTGTT	23	hsa-miR-29b	0	0	0	14CA	NA	NA	2	NA	2	12	NA	NA	0	NA	0	0	NA	NA	1.9	NA	0.4	0.4
TAGGACCATTTGAAATCAGTGTT	23	hsa-miR-29b	0	0	0	4GC	NA	NA	2	NA	7	23	NA	NA	0	NA	0	0	NA	NA	1.9	NA	1.4	0.8
AGCACCATTTGAAATCAGTGTT	22	hsa-miR-29b	tT	0	0	0	NA	NA	NA	4	NA	7	NA	NA	NA	0	NA	0	NA	NA	NA	0.3	NA	0.3
CAGCACCATTTGAAATCAGTGTT	23	hsa-miR-29b	0	0	0	1CT	NA	NA	NA	3	NA	NA	NA	NA	NA	1	NA	NA	NA	NA	NA	0.3	NA	NA
TAGCACAATTTGAAATCAGTGTT	23	hsa-miR-29b	0	0	0	7AC	NA	NA	NA	3	4	17	NA	NA	NA	0	1	0	NA	NA	NA	0.3	0.8	0.6
TAGCACCATGTGAAATCAGTGTT	23	hsa-miR-29b	0	0	0	10GT	NA	NA	NA	2	NA	NA	NA	NA	NA	0	NA	NA	NA	NA	NA	0.2	NA	NA
TAGCACCATTGGAAATCAGTGT	22	hsa-miR-29b	0	tT	0	11GT	NA	NA	NA	2	NA	NA	NA	NA	NA	0	NA	NA	NA	NA	NA	0.2	NA	NA
TAGCACCATTTGAAATCAGT	20	hsa-miR-29b	0	tGTT	0	0	NA	NA	NA	4	6	41	NA	NA	NA	0	0	0	NA	NA	NA	0.3	1.2	1.5
TAGCACCATTTGAAATCAGTG	21	hsa-miR-29b	0	tTT	0	0	NA	NA	NA	7	5	13	NA	NA	NA	0	0	0	NA	NA	NA	0.6	1	0.5
TAGCACCATTTGAAATCAGTGTTA	24	hsa-miR-29b	0	0	qA	0	NA	NA	NA	12	5	20	NA	NA	NA	0	0	0	NA	NA	NA	1	1	0.7
TAGCACCATTTGAAATCAGTGTTAA	25	hsa-miR-29b	0	0	qAA	0	NA	NA	NA	3	NA	7	NA	NA	NA	0	NA	0	NA	NA	NA	0.3	NA	0.3
TAGCACCATTTGAAATCAGTTTT	23	hsa-miR-29b	0	0	0	21TG	NA	NA	NA	9	NA	10	NA	NA	NA	0	NA	0	NA	NA	NA	0.8	NA	0.4
TAGCACCATTTGAAATTAGTGTT	23	hsa-miR-29b	0	0	0	17TC	NA	NA	NA	3	NA	NA	NA	NA	NA	0	NA	NA	NA	NA	NA	0.3	NA	NA
TAGCACCATTTGATATCAGTGTT	23	hsa-miR-29b	0	0	0	14TA	NA	NA	NA	5	2	NA	NA	NA	NA	0	0	NA	NA	NA	NA	0.4	0.4	NA
TAGCACCATTTGTAATCAGTGTT	23	hsa-miR-29b	0	0	0	13TA	NA	NA	NA	4	NA	NA	NA	NA	NA	0	NA	NA	NA	NA	NA	0.3	NA	NA
TAGCACCATTTTAAATCAGTGTT	23	hsa-miR-29b	0	0	0	12TG	NA	NA	NA	6	NA	10	NA	NA	NA	0	NA	0	NA	NA	NA	0.5	NA	0.4
TAGCACTATTTGAAATCAGTGTT	23	hsa-miR-29b	0	0	0	7TC	NA	NA	NA	9	NA	10	NA	NA	NA	0	NA	0	NA	NA	NA	0.8	NA	0.4
TAGCAGCATTTGAAATCAGTGTT	23	hsa-miR-29b	0	0	0	6GC	NA	NA	NA	2	NA	5	NA	NA	NA	0	NA	0	NA	NA	NA	0.2	NA	0.2
TAGCGCCATTTGAAATCAGTGTT	23	hsa-miR-29b	0	0	0	5GA	NA	NA	NA	2	NA	NA	NA	NA	NA	0	NA	NA	NA	NA	NA	0.2	NA	NA
TATCACCATTTGAAATCAGTGTT	23	hsa-miR-29b	0	0	0	3TG	NA	NA	NA	2	NA	NA	NA	NA	NA	0	NA	NA	NA	NA	NA	0.2	NA	NA
TAGCACCATTTGAAATAAGTGTT	23	hsa-miR-29b	0	0	0	17AC	NA	NA	NA	NA	2	22	NA	NA	NA	NA	0	0	NA	NA	NA	NA	0.4	0.8
TAGCACCGTTTGAAATCAGTGTT	23	hsa-miR-29b	0	0	0	8GA	NA	NA	NA	NA	3	NA	NA	NA	NA	NA	0	NA	NA	NA	NA	NA	0.6	NA
TGGCACCATTTGAAATCAGTGTT	23	hsa-miR-29b	0	0	0	2GA	NA	NA	NA	NA	2	NA	NA	NA	NA	NA	0	NA	NA	NA	NA	NA	0.4	NA
CTAGCACCATTTGAAATCAGTG	22	hsa-miR-29b	qC	tTT	0	0	NA	NA	NA	NA	NA	4	NA	NA	NA	NA	NA	0	NA	NA	NA	NA	NA	0.1
TAGAACCATTTGAAATCAGTGTT	23	hsa-miR-29b	0	0	0	4AC	NA	NA	NA	NA	NA	18	NA	NA	NA	NA	NA	0	NA	NA	NA	NA	NA	0.7
TAGCAACATTTGAAATCAGTGTT	23	hsa-miR-29b	0	0	0	6AC	NA	NA	NA	NA	NA	7	NA	NA	NA	NA	NA	0	NA	NA	NA	NA	NA	0.3
TAGCACCATTTGAAATCACTGTT	23	hsa-miR-29b	0	0	0	19CG	NA	NA	NA	NA	NA	6	NA	NA	NA	NA	NA	0	NA	NA	NA	NA	NA	0.2
TAGCACCATTTGAAATCAG	19	hsa-miR-29b	0	tTGTT	0	0	NA	NA	NA	NA	NA	11	NA	NA	NA	NA	NA	0	NA	NA	NA	NA	NA	0.4
TAGCACCATTTGAAATCAGTT	21	hsa-miR-29b	0	tGTT	qT	0	NA	NA	NA	NA	NA	4	NA	NA	NA	NA	NA	0	NA	NA	NA	NA	NA	0.1
TAGCACCATTTGAACTCAGTGTT	23	hsa-miR-29b	0	0	0	15CA	NA	NA	NA	NA	NA	29	NA	NA	NA	NA	NA	0	NA	NA	NA	NA	NA	1
TAGTACCATTTGAAATCAGTGTT	23	hsa-miR-29b	0	0	0	4TC	NA	NA	NA	NA	NA	5	NA	NA	NA	NA	NA	0	NA	NA	NA	NA	NA	0.2
