# Supplementary material for: Microarray and deep sequencing cross-platform analysis of the mirRNome and isomiR variation in response to epidermal growth factor
Source: BMC Genomics. 2013 Jun 1;14:371. doi: 10.1186/1471-2164-14-371 (PMC3680220; doi:10.1186/1471-2164-14-371)

sample	miRNA locus	count number						
		reference miRNA	5'-isomiR	3'-isomir	substitution	3'-addition	Total of counts annotating to	Total counts in each sample
C1	miR-29-b	148	0	25	7	3	183	4180443
C2	miR-29-b	53	0	6	2	0	61	7576291
C3	miR-29-b	94	0	5	7	0	106	8403655
EGF1	miR-29-b	929	4	146	58	22	1159	4460021
EGF2	miR-29-b	411	0	54	25	8	498	7156090
EGF3	miR-29-b	2081	21	367	279	48	2796	6024190
C1	miR-29-a	2736	129	325	179	80	3449	4180443
C2	miR-29-a	783	20	65	47	31	946	7576291
C3	miR-29-a	688	29	58	98	12	885	8403655
EGF1	miR-29-a	60889	3743	5926	4087	2293	76938	4460021
EGF2	miR-29-a	3519	84	155	245	62	4065	7156090
EGF3	miR-29-a	66396	2465	5703	6744	2886	84194	6024190
C1	miR-222	24	0	200	13	16	253	4180443
C2	miR-222	40	2	658	62	121	883	7576291
C3	miR-222	74	0	1044	186	173	1477	8403655
EGF1	miR-222	1883	22	8784	1182	1658	13529	4460021
EGF2	miR-222	1080	0	2077	231	259	3647	7156090
EGF3	miR-222	1246	91	21306	2818	3321	28782	6024190
C1	miR-221	120	0	200	40	61	421	4180443
C2	miR-221	786	4	1404	272	437	2903	7576291
C3	miR-221	1090	0	1838	610	553	4091	8403655
EGF1	miR-221	6717	21	9653	2726	2925	22042	4460021
EGF2	miR-221	3327	2	4451	929	1111	9820	7156090
EGF3	miR-221	29982	486	53822	12965	12080	109335	6024190
C1	miR-132	4	0	0	0	0	4	4180443
C2	miR-132	0	0	0	0	0	0	7576291
C3	miR-132	0	0	0	0	0	0	8403655
EGF1	miR-132	49	24	0	2	0	75	4460021
EGF2	miR-132	0	0	0	0	84	84	7156090
EGF3	miR-132	68	129	9	18	0	224	6024190
C1	miR-21	7510	207	24673	2787	302	35479	4180443
C2	miR-21	3392	168	13237	1613	3499	21909	7576291
C3	miR-21	3436	240	17679	2857	2584	26796	8403655
EGF1	miR-21	55184	2541	282898	26565	3860	371048	4460021
EGF2	miR-21	7461	450	57125	5235	50711	120982	7156090
EGF3	miR-21	47105	4103	253493	31004	9100	344805	6024190

Normalized frequencies (Per milion)						%, considering 100 the total of normalized sequences annotating to a l				
reference miRNA	5'-isomiR	3'-isomir	substitution	3'-addition	Normalized counts annotating to	reference miRNA	5'-isomiR	3'-isomir	substitution	3'-addition
35.40294653	0	5.980227454	1.674463687	0.717627295	43.77526497	80.9	0.0	13.7	3.8	1.6
6.995507432	0	0.791944238	0.263981413	0	8.051433082	86.9	0.0	9.8	3.3	0.0
11.18560912	0	0.594979208	0.832970892	0	12.61355922	88.7	0.0	4.7	6.6	0.0
208.2949834	0.896856764	32.73527187	13.00442307	4.9327122	259.8642473	80.2	0.3	12.6	5.0	1.9
57.43359852	0	7.546020243	3.49352789	1.117928925	69.59107557	82.5	0.0	10.8	5.0	1.6
345.4406319	3.485945828	60.92105329	46.31328029	7.967876179	464.1287874	74.4	0.8	13.1	10.0	1.7
654.4760926	30.85797366	77.74295691	42.81842857	19.13672785	825.0321796	79.3	3.7	9.4	5.2	2.3
103.348723	2.639814125	8.579395908	6.203563195	4.091711894	124.8632081	82.8	2.1	6.9	5.0	3.3
81.86913908	3.450879409	6.901758818	11.66159249	1.4279501	105.3113199	77.7	3.3	6.6	11.1	1.4
13652.17787	839.2337166	1328.693295	916.3633983	514.1231398	17250.59142	79.1	4.9	7.7	5.3	3.0
491.7489858	11.73825371	21.65987292	34.23657332	8.663949168	568.047635	86.6	2.1	3.8	6.0	1.5
11021.56472	409.1836413	946.6832885	1119.486603	479.0685553	13975.98681	78.9	2.9	6.8	8.0	3.4
5.741018356	0	47.84181963	3.109718276	3.827345571	60.51990184	9.5	0.0	79.1	5.1	6.3
5.279628251	0.263981413	86.84988473	8.183423789	15.97087546	116.5477936	4.5	0.2	74.5	7.0	13.7
8.805692285	0	124.2316587	22.13322655	20.58628061	175.7568582	5.0	0.0	70.7	12.6	11.7
422.1953215	4.9327122	1969.497453	265.0211737	371.7471285	3033.393789	13.9	0.2	64.9	8.7	12.3
150.9204049	0	290.2422971	32.28019771	36.19294894	509.6358486	29.6	0.0	57.0	6.3	7.1
206.8327858	15.10576526	3536.741039	467.7807307	551.2774331	4777.737754	4.3	0.3	74.0	9.8	11.5
28.70509178	0	47.84181963	9.568363927	14.59175499	100.7070303	28.5	0.0	47.5	9.5	14.5
103.7446951	0.527962825	185.3149516	35.90147211	57.67993864	383.1690203	27.1	0.1	48.4	9.4	15.1
129.7054674	0	218.714357	72.58746343	65.80470045	486.8119884	26.6	0.0	44.9	14.9	13.5
1506.04672	4.708498009	2164.339585	611.2078844	655.8265084	4942.129196	30.5	0.1	43.8	12.4	13.3
464.9186916	0.279482231	621.9877056	129.8194964	155.2523794	1372.257755	33.9	0.0	45.3	9.5	11.3
4976.934658	80.67474631	8934.313161	2152.156555	2005.248838	18149.32796	27.4	0.4	49.2	11.9	11.0
0.956836393	0	0	0	0	0.956836393	100.0	0.0	0.0	0.0	0.0
0	0	0	0	0	0	#¡DIV/0!	#¡DIV/0!	#¡DIV/0!	#¡DIV/0!	#¡DIV/0!
0	0	0	0	0	0	#¡DIV/0!	#¡DIV/0!	#¡DIV/0!	#¡DIV/0!	#¡DIV/0!
10.98649536	5.381140582	0	0.448428382	0	16.81606432	65.3	32.0	0.0	2.7	0.0
0	0	0	0	11.73825371	11.73825371	0.0	0.0	0.0	0.0	100.0
11.28782459	21.41366723	1.493976784	2.987953567	0	37.18342217	30.4	57.6	4.0	8.0	0.0
1796.460327	49.51628332	5902.006079	666.6757566	72.24114765	8486.899594	21.2	0.6	69.5	7.9	0.9
447.7124757	22.17443865	1747.160979	212.9010092	461.8354812	2891.784384	15.5	0.8	60.4	7.4	16.0
408.869712	28.55900201	2103.727485	339.9711197	307.4852549	3188.612574	12.8	0.9	66.0	10.7	9.6
12373.03591	569.7282591	63429.74618	5956.249982	865.466777	83194.22711	14.9	0.7	76.2	7.2	1.0
1042.608464	62.88350202	7982.711229	731.5447402	7086.411714	16906.15965	6.2	0.4	47.2	4.3	41.9
7819.308488	681.0874159	42079.18409	5146.584022	1510.576526	57236.74054	13.7	1.2	73.5	9.0	2.6

		AVERAGE					STDEVP				
		reference miRNA	5'-isomiR	3'-isomir	substitution	3'-addition	reference miRNA	5'-isomiR	3'-isomir	substitution	3'-addition
C	miR-29b	85.5	0.0	9.4	4.6	0.5	4.087921379	0	4.487682882	1.783050738	0.946475851
EGF	miR-29b	79.0	0.4	12.2	6.7	1.7	4.165191916	0.375946715	1.194780185	2.867330639	0.147319496
C	miR-29a	79.9	3.0	7.6	7.1	2.3	2.571103306	0.837711311	1.573028453	3.462611704	0.960513334
EGF	miR-29a	81.5	3.3	6.1	6.4	2.6	4.371466733	1.433319646	2.031173453	1.397781866	0.994768799
C	miR-222	6.3	0.1	74.8	8.3	10.6	2.733394592	0.130770163	4.188629353	3.876471405	3.817789449
EGF	miR-222	16.0	0.2	65.3	8.3	10.3	12.76443086	0.158106535	8.543398468	1.771745187	2.791537253
C	miR-221	27.4	0.0	46.9	11.3	14.4	0.973338593	0.079552224	1.788267393	3.161894874	0.776926224
EGF	miR-221	30.6	0.2	46.1	11.2	11.9	3.230470168	0.226372914	2.80124323	1.552382185	1.21333823
C	miR-21	16.5	0.7	65.3	8.6	8.8	4.262772051	0.156897442	4.598320092	1.779938845	7.593105794
EGF	miR-21	11.6	0.7	65.7	6.8	15.2	4.715508991	0.412744818	16.0290343	2.350128659	23.15193719

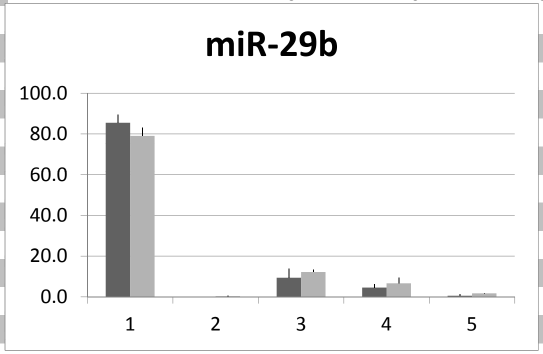

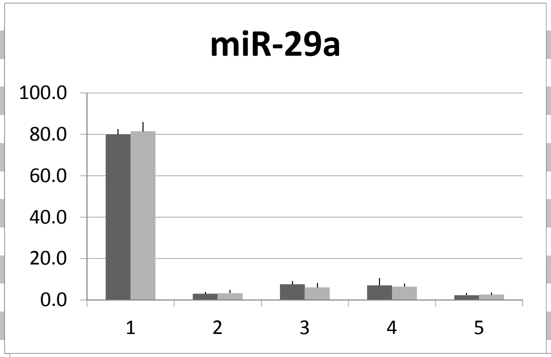

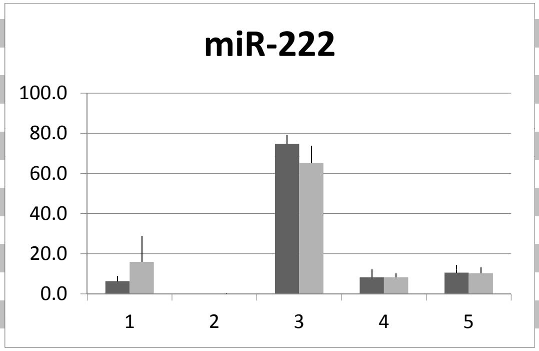

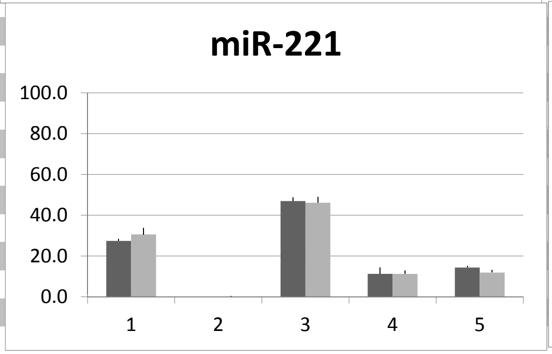

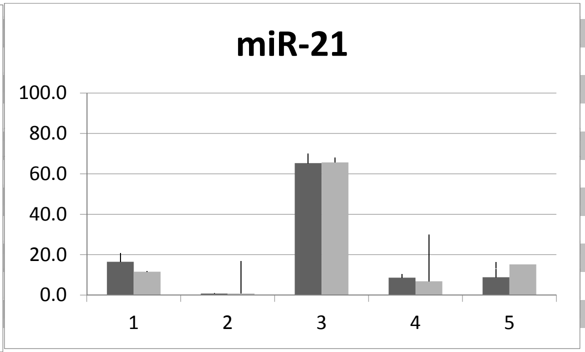

Supplement: Additional file 11 — Listing of isomiR sequence variants and their respective read counts for top EGF regulated miRNAs. [file 1471-2164-14-371-S11.pdf]
